# RNA Expression Profile and Potential Biomarkers in Patients With Spinocerebellar Ataxia Type 3 From Mainland China

**DOI:** 10.3389/fgene.2019.00566

**Published:** 2019-06-13

**Authors:** Tianjiao Li, Xiaocan Hou, Zhao Chen, Yun Peng, Puzhi Wang, Yue Xie, Lang He, Hongyu Yuan, Huirong Peng, Rong Qiu, Kun Xia, Beisha Tang, Hong Jiang

**Affiliations:** ^1^Department of Neurology, Xiangya Hospital, Central South University, Changsha, China; ^2^School of Information Science and Engineering, Central South University, Changsha, China; ^3^Medical Genetics Research Center, Central South University, Changsha, China; ^4^National Clinical Research Center for Geriatric Diseases, Xiangya Hospital, Central South University, Changsha, China; ^5^Key Laboratory of Hunan Province in Neurodegenerative Disorders, Central South University, Changsha, China

**Keywords:** Spinocerebellar Ataxia Type 3, Machado–Joseph disease, long non-coding RNAs, expression profile, biomarker

## Abstract

Long non-coding RNAs (lncRNAs) play an important role in growth, development, and reproduction and undoubtedly contribute to the pathogenesis and progression of diseases. Emerging evidence suggests the involvement of lncRNAs as regulatory factors in pathological conditions, including some neurodegenerative diseases. Spinocerebellar Ataxia Type 3/Machado–Joseph Disease (SCA3/MJD) has a prominent prevalence in China. Because the role of lncRNAs in SCA3/MJD pathogenesis has not yet been investigated, we conducted a pilot study to investigate the expression profile of lncRNAs by high-throughput sequencing in 12 patients and 12 healthy individuals. The sequencing analysis detected 5,540 known and 2,759 novel lncRNAs. Six lncRNAs were confirmed to be differentially expressed in peripheral blood mononuclear cells between SCA3/MJD patients and healthy individuals and were further validated in cerebellar tissue. Based on these results, NONHSAT022144.2 and NONHSAT165686.1 may be involved in the pathogenesis of SCA3/MJD and may be potential biomarkers for SCA3/MJD. Together with NONHSAT022144.2 and NONHSAT165686.1, the other four novel lncRNAs increase our understanding of lncRNA expression profile.

## Introduction

Long non-coding RNAs (lncRNAs) play an essential role in regulating the expression of genes involved in almost all metabolic pathways and other biological macromolecules at both the transcriptional and post-transcriptional levels. lncRNAs also play a role in the regulation of growth, development, and reproduction (Wapinski and Chang, 2011; Khorkova et al., 2015; Lardenoije et al., 2015; Engreitz et al., 2016). A growing body of evidence suggests that lncRNAs are involved in the pathogenesis and progression of some neurodegenerative diseases, such as Alzheimer’s Disease (AD) and Huntington’s disease (HD) (Lin et al., 2014; Tan et al., 2014; Salta and De Strooper, 2017; Sunwoo et al., 2017; Zhou et al., 2018).

Alteration in the expression levels of some lncRNAs contributes to the pathogenesis of neurodegenerative diseases (Lin et al., 2014; Roberts et al., 2014; Tan et al., 2014; Zhang et al., 2016; Salta and De Strooper, 2017; Sunwoo et al., 2017; Gagliardi et al., 2018). For example, lnc-sca7 (*ATXN7L3B*) is highly conserved in the central nervous system of human and adult mice and transcriptionally regulates the expression of *ATXN7*, which is the pathogenic gene of Spinocerebellar Ataxia Type 7 (SCA7). Indeed, knockout of lnc-sca7 in N2a cells leads to a significant decrease in *ATXN7* expression at the translational level. Accordingly, overexpression of lnc-sca7 significantly enhances transcription of *ATXN7* (Tan et al., 2014; Salta and De Strooper, 2017). A more detailed study showed that the lncRNA TUNA has elevated expression in the thalamus and striatum. Gene expression analysis in brain tissue of 44 HD patients and 36 healthy individuals confirmed that TUNA expression in the caudate nucleus might be involved in the pathophysiology of HD (Lin et al., 2014; Salta and De Strooper, 2017). Additionally, the lncRNA NEAT1 is associated with the damage mechanism of HD (Sunwoo et al., 2017).

Polyglutamine disease (PolyQ disease) is a type of disease caused by dynamic mutation of a trinucleotide sequence; PolyQ diseases include spinocerebellar ataxia (SCA) types 1, 2, 3, 6, 7, and 17; dentatorubral–pallidoluysian atrophy (DRPLA); spinal and bulbar muscular atrophy (SBMA), and HD (Matos et al., 2011). Spinocerebellar Ataxia Type 3/Machado–Joseph Disease, one of the PolyQ diseases similar to SCA7 and HD (Wang et al., 2015), is the most common SCA subtype in mainland China (Chen et al., 2018). It is mainly caused by the abnormal expansion of the trinucleotide sequence CAG in the ATXN3 gene and has a broad age of onset ranging from 4- to over 70-years-old (Peng et al., 2014; Wang et al., 2015; Chen Z. et al., 2016; Chen et al., 2017). Although various pathogenic hypotheses have been proposed and supported experimentally for SCA3/MJD, no effective strategies are available for surveillance, delay progression, or treatment of the disease (Costa Mdo and Paulson, 2012). Given that some lncRNAs are involved in SCA7 and HD pathogenesis (Lin et al., 2014; Roberts et al., 2014; Tan et al., 2014; Zhang et al., 2016; Salta and De Strooper, 2017; Sunwoo et al., 2017; Gagliardi et al., 2018) and that we previously detected abnormal expression of three lncRNAs in the SCA3/MJD mouse model (Vidal et al., 2000), we speculated that some lncRNAs also contribute to SCA3/MJD pathogenesis. Largely because of their accessibility and ease of operation, peripheral blood mononuclear cells (PBMCs) are widely used to establish suitable biomarkers for neurodegenerative and other diseases (Coppola et al., 2011; Zhang et al., 2016; Gagliardi et al., 2018). To investigate the possible mechanisms of lncRNAs in SCA3/MJD pathogenesis and to explore potential SCA3/MJD biomarkers, we isolated PBMCs from patients and healthy individuals for high-throughput sequencing. Through bioinformatics analysis, we identified 5,540 known and 2,759 novel lncRNAs in patients and normal individuals. Validation including expression analysis in human brains enabled us to target six lncRNAs. This provides useful information for understanding the pathogenesis of SCA3/MJD and enabling new treatment strategy options.

## Materials and Methods

### Sample Collection and PBMC Isolation

All participants, including the 12 patients and 12 healthy individuals from Xiangya Hospital of Central South University, were required to sign consent forms prior to collection of their blood samples. The subjects included six males and six females ranging in age from 18- to 62-years-old. All of the patients met the clinical manifestations of SCA3/MJD, had been diagnosed with SCA3/MJD by neurologists and geneticists, and were confirmed to not have any other known brain or neurological disorder. By matching age and gender, we found suitable healthy individuals from the physical examination center at Xiangya Hospital. Ten milliliters of peripheral blood was collected for isolation of PBMCs. The blood samples collected from participants were processed within 2 h to isolate PBMCs using lymphocyte solution (Ficoll–Hypaque) based on the standard protocol instructions.

### RNA Extraction and High-Throughput Sequencing

TRIzol^TM^ reagent (Invitrogen, Carlsbad, CA, United States) was added to isolated PBMCs for total RNA extraction, and genomic DNA was removed with the RNeasy kit (Qiagen, Hilden, Germany). Ribosomal RNA removal was conducted using a biotin-labeled specific probe from the Ribo-Zero^TM^ rRNA Removal Kit (Epicentre)^®^ prior to RNA library preparation. The first strand cDNA was then synthesized using random primers and reverse transcriptase in the TruSeq^®^ Stranded kit (Illumina) followed by synthesis of the double-stranded cDNA using DNA polymerase I and RNaseH. The adaptor-ligated cDNA fragments were amplified to generate the final cDNA library followed by library purification. High-throughput sequencing was carried out by BGI Genomics with the Illumina Hi-seq X-Ten platform.

High-throughput sequencing obtained raw reads as data. Subsequently, we used the short reads alignment tool SOAP^1^ to compare reads to the ribosomal database and filtered data by removing the ribosomal reads on the alignment, which allows for up to five mismatches. Then, reads containing only adapter dimers or reads with 10% or more N or low-quality bases were filtered out. After that, we obtained the clean reads. Clean reads were aligned to the reference genome with the HISAT software^2^ and assembled with StringTie^3^. The Hg19 genome assembly version was used for alignment and assembly. By aligning all reads to the genome and constructing all possible transcripts in each alignment region, only the transcripts with fragments per kilobase of transcript per million reads mapped (FPKM) values ≥ 0.5, coverage values > 1, and length values > 200 bp were retained for further quantitative analysis. To compare the expression levels between samples, it was necessary to standardize the expression level of the gene. Therefore, we aligned the clean reads to the reference sequence with Bowtie2^4^, and expression levels of genes and transcripts were calculated by using RSEM^5^. The standardized method of RSEM is FPKM. Its calculation method is FPKM(A) = 10^6^*C*/(*NL*/10^3^), where FPKM(A) is the expression level of gene A, *C* is the number of fragments that are uniquely aligned to gene A, *N* is the total fragment number that is aligned to the reference gene, and *L* is the number of bases of the gene A coding region. We needed to predict whether transcripts had coding ability to determine if they were lncRNAs. Three prediction programs – CPC^6^, txCdsPredict^7^, and CNCI^8^ – were used to score the transcripts’ coding ability to distinguish mRNA and lncRNA by different score ranges. The thresholds in the CPC and CNCI software were set to 0, and mRNAs had scores greater than 0, while lncRNAs had scores less than 0. The txCdspredict threshold was set to 500, and mRNA transcripts had scores over 500, lncRNAs had scores while less than 500. Additionally, transcripts were compared to the protein database Pfam^9^ to classify coding RNAs (mRNAs) and lncRNAs. We considered transcripts lncRNAs only if they met the criteria of at least three of the four software and databases, and then we analyzed their predicted target gene functional annotation. Moreover, we considered a fold change ≥ 2.00 and false discovery rate ≤ 0.001 significant in DEGseq (Wang et al., 2010) to compare lncRNA expression differences the SCA3/MJD and control group All data alignments, assembly and analysis were conducted by BGI Genomics.

### Target Gene Prediction

We determined the Spearman and Pearson correlation coefficients to determine lncRNA targets; only mRNAs with a Spearman correlation ≥ 0.6 and Pearson correlation ≥ 0.6 were considered target genes of a given lncRNA. When the lncRNA overlapped with the target gene, we classified the lncRNAs and their target mRNAs based on their positional relationship.

### Gene Function Annotation

We analyzed differentially expressed genes (DEGs) and their target genes with the GO database^10^ to explore possible functional enrichment analyses of DEGs and identify functional modules enriched in DEGs. To determine the pathways that DEGs and their target genes are concentrated in, we performed enrichment analysis using the KEGG database^11^.

### Quantitative Real-Time PCR Verification in PBMCs and Human Brain Tissue

Reverse transcription was carried out with the Goldenstar^TM^ RT6 cDNA Synthesis Kit (Beijing TsingKe Biotech Co. Ltd., China). Based on the analysis of the high-throughput sequencing, 20 lncRNAs were selected for quantitative real-time PCR (qRT-PCR) to confirm their differential expression between the case and control groups. Further validation was performed in 28 SCA3/MJD patients and 28 healthy individuals as mentioned in the section “Materials and Methods.” Primers for RT-PCR listed in Supplementary Table S1 were designed by Primer 5, and the qRT-PCR was performed per the instructions of the 2 × T5 Fast qPCR Mix (SYBR Green I) Kit. We used GAPDH as an internal control and the 2^–ΔΔCt^ method to calculate the quantitative expression of lncRNAs. The Wilcoxon rank-sum test was used to compare statistical significance in the expression levels of DEGs between groups, and *p*-values < 0.05 were considered statistically significant. For further validation, statistically significant lncRNAs were validated by qRT-PCR in cerebellar tissue from a SCA3/MJD patient and a healthy individual.

## Results

### Expression Profiles of lncRNAs and mRNAs

A total of 124,394 transcripts was detected by high-throughput sequencing, including 15,926 novel lncRNAs, 13,651 novel mRNAs, 61,335 known lncRNAs, and 33,482 known mRNAs. After data filtering and DEG analysis with DEGseq, we identified 5,540 known lncRNAs and 2,759 novel lncRNAs with differential expression. Additionally, we identified 4,701 known mRNAs and 2,517 novel mRNAs.

### Summary of the *Cis*- and *Trans*-Regulation and Classification of the Overlap

Through target gene prediction analyses, we predicted the possible regulatory patterns of lncRNAs. All of the positional information and the complementarity between mRNAs and lncRNAs are shown in Supplementary Material II, and we indicate how lncRNAs may act on their target genes. The overlap is divided into 10 classes (Table 1), including Lnc-Overlap-mRNA with 812 pairs, Lnc-AntiOverlap-mRNA with 204 pairs, Lnc-Complete In-mRNAExon with 153 pairs, Lnc-AntiComplete In-mRNAExon with eight pairs, mRNA-CompleteIn-LncExon with 47 pairs, and mRNA-AntiCompleteIn-LncExon with 2 pairs. Lnc-CompleteIn-mRNA Intron with 235 pairs, Lnc-AntiComplete In-mRNA Intron with 64 pairs, mRNA-Complete In-LncIntron with 19 pairs, and mRNA-AntiComplete In-LncIntron with 18 pairs. As listed in Table 2, we predicted the target genes for 5 of 20 tested lncRNAs in this study.

**TABLE 1:** The classification of overlap.

**Overlap class**	**LncRNA**	**mRNA**	**Pair**
	**number**	**number**	**number**
Lnc-Overlap-mRNA	624	664	812
Lnc-AntiOverlap-mRNA	188	189	204
Lnc-CompleteIn-mRNAExon	154	156	159
Lnc-AntiCompleteIn-mRNAExon	8	8	8
mRNA-CompleteIn-LncExon	42	46	47
mRNA-AntiCompleteIn-LncExon	2	2	2
Lnc-CompleteIn-mRNAIntron	212	213	235
Lnc-AntiCompleteIn-mRNAIntron	63	56	64
mRNA-CompleteIn-LncIntron	14	16	19
mRNA-AntiCompleteIn-LncIntron	15	10	18

*Lnc-Overlap-mRNA means lncRNA on the same strand as mRNA, and lncRNA has an overlap with mRNA. Lnc-AntiOverlap-mRNA means that lncRNA and mRNA are on different strands, and there is an overlap between lncRNA and mRNA. Lnc-CompleteIn-mRNAExon means that lncRNA and mRNA are on the same strand, and the lncRNA completely falls in the Exon region of mRNA. Lnc-AntiCompleteIn-mRNAExon means lncRNA and mRNA are on different strands, and the lncRNA completely falls in the Exon region of mRNA. mRNA-CompleteIn-LncExon means that lncRNA and mRNA are on the same strand, and the mRNA completely falls in the Exon region of lncRNA. mRNA-AntiCompleteIn-LncExon means that lncRNA and mRNA are on different strands, and mRNA completely falls in the Exon region of lncRNA. Lnc-CompleteIn-mRNAIntron means that lncRNA and mRNA are on the same strand, and the lncRNA completely falls in the Intron region of mRNA. Lnc-AntiCompleteIn-mRNAIntron means lncRNA and mRNA are on different strands, and the lncRNA completely falls in the Intron region of mRNA. mRNA-CompleteIn-LncIntron means that lncRNA and mRNA are on the same strand, and mRNA completely falls in the Intron region of lncRNA. mRNA-AntiCompleteIn-LncIntron means that lncRNA and mRNA are on different strands, and mRNA completely falls in the Intron region of lncRNA.*

**TABLE 2:** The classification of overlap of DEGs.

**LncRNA ID**	**Target mRNA**	**Overlap class**	**The value of**	**The value of**
			**Pearson correlation**	**Spearman correlation**
LTCONS_00174904	MTCONS_00174903	Lnc-CompleteIn-mRNAIntron	0.9031	0.7902
LTCONS_00175021	MTCONS_00174902	NA	0.8225	0.8433
LTCONS_00051791	MTCONS_00051780	Lnc-Overlap-mRNA	0.9743	0.7005
NONHSAT007220.2	NM_001184763	Lnc-CompleteIn-mRNAExon	0.6652	0.7045
NONHSAT177312.1	NM_001291470	NA	0.8275	0.6707

*The details of overlap class included in Table 1, and NA means the lncRNA and their target gene are on different chromosomes.*

### Gene Function Annotation

**FIGURE 1:**
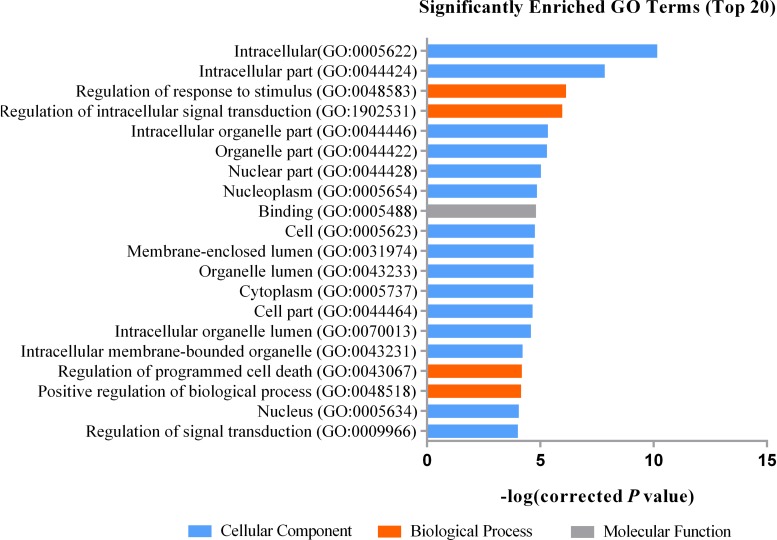
The top 20 significantly enriched GO terms for DEGs. The top 20 GO items are Intracellular (GO:0005622), Intracellular part (GO:0044424), Regulation of response to stimulus (GO:0048583), Regulation of intracellular signal transduction (GO:1902531), Intracellular organelle part (GO:0044446), Organelle part (GO:0044422), Nuclear part (GO:0044428), Nucleoplasm (GO:0005654), Binding (GO:0005488), Cell (GO:0005623), Membrane-enclosed lumen (GO:0031974), Organelle lumen (GO:0043233), Cytoplasm (GO:0005737), Cell part (GO:0044464), Intracellular organelle lumen (GO:0070013), Intracellular membrane-bounded organelle (GO:0043231), Regulation of programmed cell death (GO:0043067), Positive regulation of biological process (GO:0048518), Nucleus (GO:0005634), Regulation of signal transduction (GO:0009966), they are mainly enriched in cellular components.

**FIGURE 2:**
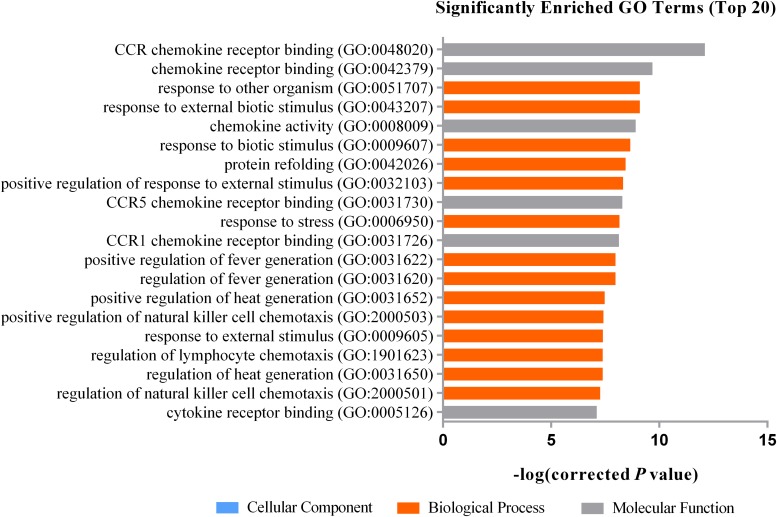
The top 20 significantly enriched GO terms for the target gene of DEGs. Top 20 GO terms consist of biological processes and molecular functions, they are CCR chemokine receptor binding (GO:0048020), chemokine receptor binding (GO:0042379), response to external biotic stimulus (GO:0043207), response to other organism (GO:0051707), chemokine activity (GO:0008009), response to biotic stimulus (GO:0009607), protein refolding (GO:0042026), positive regulation of response to external stimulus (GO:0032103), CCR5 chemokine receptor binding (GO:0031730), response to stress (GO:0006950), CCR1 chemokine receptor binding (GO:0031726), regulation of fever generation (GO:0031620), positive regulation of fever generation (GO:0031622), positive regulation of heat generation (GO:0031652), positive regulation of natural killer cell chemotaxis (GO:2000503), response to external stimulus (GO:0009605), regulation of heat generation (GO:0031650), regulation of lymphocyte chemotaxis (GO:1901623), regulation of natural killer cell chemotaxis (GO:2000501), cytokine receptor binding (GO:0005126).

**FIGURE 3:**
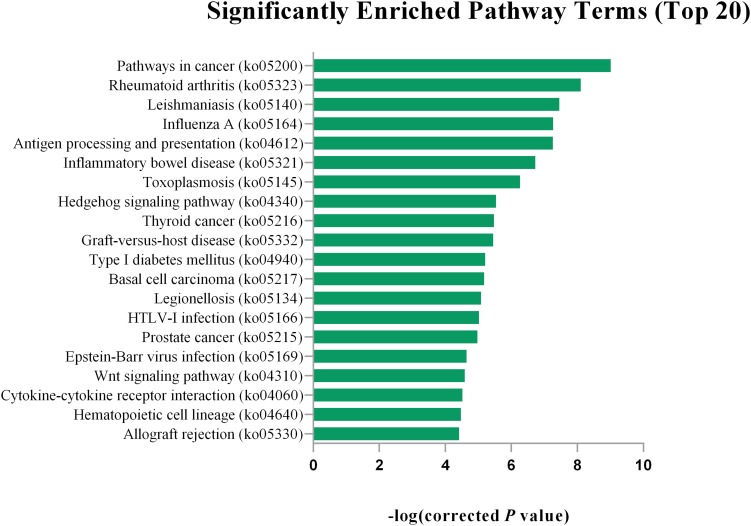
The top 20 significantly enriched pathway terms for DEGs. The top 20 KEGG pathway terms are Pathways in cancer (ko05200), Rheumatoid arthritis (ko05323), Leishmaniasis (ko05140), Influenza A (ko05164),Influenza A (ko05164), Antigen processing and presentation (ko04612), Inflammatory bowel disease (ko05321), Toxoplasmosis (ko05145), Hedgehog signaling pathway (ko04340), Thyroid cancer (ko05216), Graft-versus-host disease (ko05332), Type I diabetes mellitus (ko04940), Basal cell carcinoma (ko05217), Legionellosis (ko05134), HTLV-I infection (ko05166), Prostate cancer (ko05215), Epstein-Barr virus infection (ko05169), Wnt signaling pathway (ko04310), Cytokine-cytokine receptor interaction (ko04060), Hematopoietic cell lineage (ko04640), Allograft rejection (ko05330).

**FIGURE 4:**
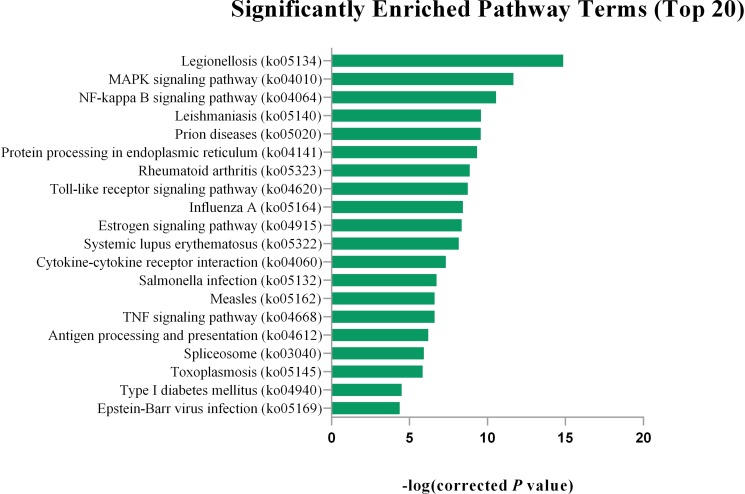
The top 20 significantly enriched pathway terms for the target gene of DEGs. The top 20 KEGG pathway terms are Legionellosis (ko05134), MAPK signaling pathway (ko04010), NF-kappa B signaling pathway (ko04064), Leishmaniasis (ko05140), Prion diseases (ko05020), Protein processing in endoplasmic reticulum (ko04141), Rheumatoid arthritis (ko05323), Toll-like receptor signaling pathway (ko04620), Influenza A (ko05164), Estrogen signaling pathway (ko04915), Systemic lupus erythematosus (ko05322), Cytokine-cytokine receptor interaction (ko04060), Salmonella infection (ko05132), Measles (ko05162), TNF signaling pathway (ko04668), Antigen processing and presentation (ko04612), Spliceosome (ko03040)Toxoplasmosis (ko05145), Type I diabetes mellitus (ko04940), Epstein-Barr virus infection (ko05169).

Gene Ontology (GO) analysis showed that DEGs were significantly enriched in certain functional terms, and the top 20 GO terms are listed in Figure 1. Similarly, we performed GO analysis of target genes of DEGs and listed significant enriched GO terms in Figure 2. KEGG analysis indicated that DEGs and their target genes were enriched in different pathways. We listed the top 20 terms in Figures 3, 4.

### Validation of lncRNAs

**FIGURE 5:**
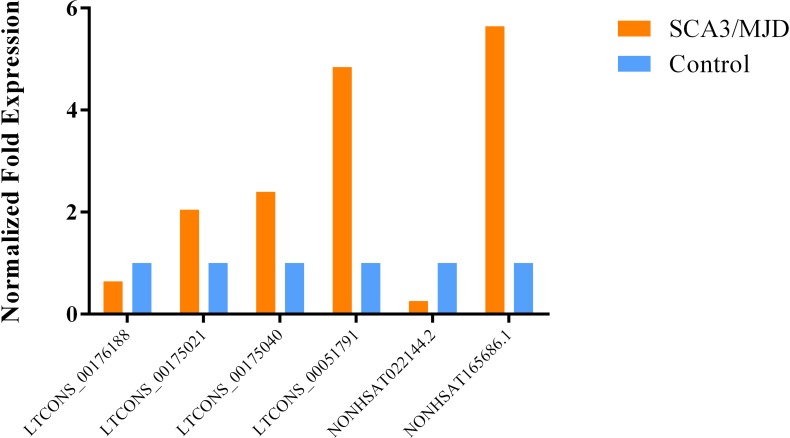
The expression level of statistically significant DEGs in PBMCs. The expression levels of NONHSAT165686.1, LTCONS_00175040, LTCONS_00175021, and LTCONS_00051791 are up-regulated.Compared with the control group, the expression level of NONHSAT165686.1 is about 5.6 times (*p* = 0.036), the expression of LTCONS_00051791 is about 4.8 times (*p* = 0.018), and LTCONS_00175021 and LTCONS_00175040 are both up more than twice (*p* = 0.027 of LTCONS_00175021 and *p* = 0.032 of LTCONS_00175040). Contrary to this, both known lncRNA NONHSAT022144.2 and novel lncRNA LTCONS_00176188 are down-regulated, their expression levels are 0.259 times (*p* = 0.000) and 0.640 times (*p* = 0.043) that of the control group, respectively.

**FIGURE 6:**
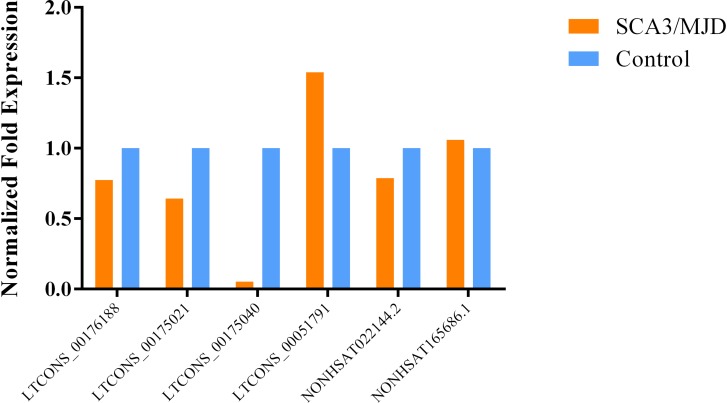
The expression level of statistically significant DEGs in cerebellum tissue. The expression levels of LTCONS_00176188 and NONHSAT022144.2 decreased by about a quarter in the cerebellar tissues of SCA3/MJD patients, while the expression levels of LTCONS_00175021 decreased by about a third in the cerebellar tissues of SCA3/MJD patients, Surprisingly, the expression of LTCONS_00175040 is only 5% of the control. Among the up-regulated genes, the expression level of NONHSAT165686.1 was only 5% higher than that of the control group, while the expression level of LTCONS_00051791 was 1.5 times that of the control group.

Among the 20 lncRNAs we selected, we verified that 6 lncRNAs are statistically different in PBMCs. The NONHSAT165686.1, LTCONS_00051791, LTCONS_00175021, and LTCONS_00175040 lncRNAs were up-regulated by 5.6, 4.8, 2.0, and 2.4-fold, respectively. There were also two down-regulated lncRNAs. The expression of NONHSAT022144.2 in the SCA3/MJD group was approximately one-quarter of the control group, while the expression of LTCONS_00176188 in the SCA3/MJD group was approximately two-thirds of the control group (Figure 5). All six lncRNAs were verified in cerebellum tissue (Figure 6). The dysregulation of these four lncRNAs, NONHSAT165686.1, LTCONS_00051791, NONHSAT022144.2, and LTCONS_00176188, in cerebellum tissue was consistent with the results in PBMCs. In contrast, the expression trends for LTCONS_00175021 and LTCONS_00175040 in cerebellum tissue differed from those in PBMCs.

## Discussion

To explore possible biomarkers for SCA3/MJD, we performed high-throughput sequencing of 12 SCA3/MJD patients and 12 healthy individuals. We found 3,812 known lncRNAs and 2,300 novel lncRNAs that were up-regulated in the SCA3/MJD group compared to the control group, while the remainder of the lncRNAs was down-regulated. We analyzed 20 lncRNAs with qRT- PCR, including 8 known lncRNAs and 12 novel lncRNAs. The results suggested that six of them were significantly differentially expressed in the two groups, including two known and four novel lncRNAs.

According to the NONCODE database^12^, NONHSAT022144.2 is located on chr11: 65499058–65506444 from database hg38, is most highly expressed in the heart (FPKM = 202.808) and second most in the brain (FPKM = 152.041). According to the NCBI database^13^, NONHSAT022144.2 aligns to the three transcripts of *MALAT1*, which are NR_002819.4, NR_144567.1, and NR_144568. The mature *MALAT1* transcript is not stabilized by a poly(A) tail but instead has a 3′-triple helical structure. The *MALAT1* gene is associated with cancer metastasis, cell migration, and cell cycle regulation. Studies have confirmed that *MALAT1* inhibits miR-101 expression (Li et al., 2017), and miR-101 regulates apoptosis, cellular stress, metastasis, autophagy, and tumor growth (Assali et al., 2018). In the SAMP8 mice which are an AD animal model, miR-101 is an important node that regulates the expression of gene networks in the brain (Cheng et al., 2013). Additionally, *MALAT1* plays an important role in the differentiation of N2a cells, and knockdown of *MALAT1* may lead to neurite outgrowth and cell death through the ERK/MAPK signaling pathway (Chen L. et al., 2016). This evidence indicates that *MALAT1* is important in the development, growth, differentiation, and function of the nervous system, and SCA3/MJD is closely related to the functional regression of the nervous system. Notably, *MALAT1* is also known as *NEAT2* (nuclear paraspeckle assembly transcript 2), and its family member *NEAT1* resists neuronal damage and contributes to neuroprotection in patients with HD. This suggests that *NEAT1* is a potential target for therapeutic treatment (Sunwoo et al., 2017). It is well-known that the pathogenesis of HD and SCA3/MJD, which are both PolyQ diseases, is similar. This may be evidence that NONHSAT022144.2 could be a potential therapeutic molecule for SCA3/MJD. Additionally, we found that the expression level of this lncRNA was significantly decreased in SCA3/MJD patients relative to healthy individuals, suggesting its potential as a biomarker and therapeutic molecule.

NONHSAT165686.1 is located on Chr13:48233221–48261860, as described in the hg38 and NONCODE database^14^, and it aligns to the *ITM2B*, NM_021999.4 transcript. To our knowledge, mutation of this gene leads to two autosomal dominant neurodegenerative diseases: familial British dementia (FBD) and familial Danish dementia (FDD). FBD is characterized by progressive dementia, cerebellar ataxia, and spasticity and is partially similar to SCA3/MJD (Vidal et al., 1999). Coincidentally, FDD shares similar symptoms such as progressive ataxia (Vidal et al., 2000). FBD also shares certain similarities with SCA3/MJD pathogenesis. Mitochondrial dysfunction has been implicated in the pathogenesis of cerebral amyloidosis such as FBD (Todd et al., 2014). Similarly, mitochondrial dysfunction is involved in the pathogenesis of SCA3/MJD, and ataxin-3 proteolysis produces toxic fragments, leading to mitochondrial defects (Harmuth et al., 2018). Expression of *ITM2B* induces apoptosis and is associated with loss of mitochondrial membrane potential, release of cytochrome c, and induction of apoptosis by a caspase-dependent mitochondrial pathway (Fleischer et al., 2002). Our results show that the expression of NONHSAT165686.1 is 5.6-fold higher in SCA3/MJD than the control group. This suggests that NONHSAT165686.1 might be associated with the pathogenesis and clinical features of SCA3/MJD.

Although only two human cerebellar tissues were used for validation, it seems that these lncRNAs are differentially expressed in cerebellar tissue between SCA3/MJD patients and healthy individuals. More importantly, we detected a significant decrease in LTCONS_00175040 expression in cerebellar tissue of SCA3/MJD patients relative to the healthy individual. We only used two human samples for validation due to the limitation of human brain sample collection. Even though we cannot exclude the possibility that the difference between these two human brain samples (the SCA3/MJD patients and the healthy individuals) was caused by individual variation, this result at least confirms the expression of these lncRNAs in the cerebellum. Thus, further validation using cerebellar tissues from more patients and healthy individuals is indispensable for concluding that LTCONS_00175040 is an important molecule. Additionally, more than 10 other lncRNAs have been identified near the LTCONS_00175040 lncRNA locus, indicating that this is a lncRNA-enriched region. Furthermore, four novel lncRNAs contribute to the expansion and improvement of the lncRNA expression profile.

In summary, our study may open a new angle for dissecting SCA3/MJD pathogenesis based on lncRNA analysis. By further exploring potential functions and pathways in which the lncRNAs are involved, we anticipate that lncRNAs such as NONHSAT022144.2 and NONHSAT165686.1 will be biomarkers or even potential therapeutic targets for SCA3/MJD treatment.

## Ethics Statement

The study was approved by the Ethics Committee of Xiangya Hospital of Central South University in China (equivalent to an Institutional Review Board), and written informed consent was obtained from all of the patients.

## Author Contributions

All the authors contributed to the experimental design and sample collection. TL completed the statistical analysis and wrote the manuscript.

## Conflict of Interest Statement

The authors declare that the research was conducted in the absence of any commercial or financial relationships that could be construed as a potential conflict of interest. The handling Editor declared a past collaboration with several of the authors HJ and BT.

## Footnotes

1 https://github.com/BGI-flexlab/SOAPnuke

2 http://www.ccb.jhu.edu/software/hisat

3 http://ccb.jhu.edu/software/stringtie

4 http://bowtie-bio.sourceforge.net/bowtie2/index.shtml

5 http://deweylab.biostat.wisc.edu/rsem

6 http://CPC.cbi.pku.edu.cn

7 http://hgdownload.soe.ucsc.edu/admin/jksrc.zip

8 https://github.com/www-bioinfo-org/CNCI

9 http://pfam.xfam.org/

10 http://www.geneontology.org/

11 https://www.kegg.jp/

12 https://www.bioinfo.org/NONCODE2016/

13 https://www.ncbi.nlm.nih.gov/gene/378938

14 http://www.noncode.org/show_rna.php?id=NONHSAT165686&version=1&utd=1#
