# Supplementary material for: RNA Expression Profile and Potential Biomarkers in Patients With Spinocerebellar Ataxia Type 3 From Mainland China
Source: Front Genet. 2019 Jun 13;10:566. doi: 10.3389/fgene.2019.00566 (PMC6584761; doi:10.3389/fgene.2019.00566)
Supplement: Supplementary file 1 [file Data_Sheet_1.PDF]

lncRNA class	Overlap class	lncRNA	mRNA	lncRNA chr	lncRNA start	lncRNA end	mRNA chr	mRNA start	mRNA end
tran	NA	LTCONS_0000024	NM_006983	chr1	1630032	1633812	chr1	1567560	1570030
cis_mRNA dw20k	NA	LTCONS_00000246	NM_001199787	chr1	1647920	1650769	chr1	1656277	1677438
tran	NA	LTCONS_0000049	MTCONS_0020881	chr1	6707579	6708407	chrX	148558521	148586884
cis_mRNA dw20k	NA	LTCONS_00000697	NM_013319	chr1	11354926	11359596	chr1	11333255	11348491
cis_mRNA_overlap	Lnc-AntiOverlap-mRNA	LTCONS_00000932	MTCONS_00009845	chr1	16933805	16953640	chr1	16888603	16940100
tran	NA	LTCONS_0000112	MTCONS_0000984	chr1	21766543	21793805	chr1	16888603	16940100
cis_mRNA dw20k	NA	LTCONS_00003619	NM_006820	chr1	79121316	79129763	chr1	79086067	79111830
cis_mRNA overlap	Lnc-Overlap-mRNA	LTCONS_00003619	MTCONS_00003615	chr1	79121316	79129763	chr1	79086067	79129763
cis_mRNA overlap	Lnc-Overlap-mRNA	LTCONS_00003619	NM_006417	chr1	79121316	79129763	chr1	79115477	79129763
cis_mRNA dw20k	NA	LTCONS_00003620	NM_006820	chr1	79125438	79126057	chr1	79086067	79111830
cis_mRNA overlap	Lnc-CompleteIn-	LTCONS_00003620	MTCONS_00003615	chr1	79125438	79126057	chr1	79086067	79129763
cis_mRNA overlap	Lnc-CompleteIn-	LTCONS_00003620	NM_006417	chr1	79125438	79126057	chr1	79115477	79129763
cis_mRNA overlap	other	LTCONS_00005287	NM_018379	chr1	150968886	150969301	chr1	150969301	150980854
cis_mRNA overlap	Lnc-Overlap-mRNA	LTCONS_00005296	MTCONS_00005304	chr1	151012478	151098021	chr1	151043080	151098021
cis_mRNA_overlap	mRNA-CompleteIn-LncIntron	LTCONS_00005296	NM_144618	chr1	151012478	151098021	chr1	151043080	151091007
cis_mRNA_overlap	Lnc-AntiOverlap-mRNA	LTCONS_00005692	NM_001282858	chr1	155714539	155720107	chr1	155719449	155827086

cis_mRNA_overlap	Lnc-AntiOverlap-mRNA	LTCONS_00005904	MTCONS_00014282	chr1	159823844	159856513	chr1	159808672	159825137
cis_mRNA_up10k	NA	LTCONS_00005911	NM_001277224	chr1	159893594	159895241	chr1	159887897	159893507
cis_mRNA_overlap	Lnc-Overlap-mRNA	LTCONS_00005970	MTCONS_00005969	chr1	160708847	160724608	chr1	160708847	160724608
cis_mRNA_overlap	Lnc-Overlap-mRNA	LTCONS_00006249	MTCONS_00006251	chr1	167190066	167268119	chr1	167190066	167350237
cis_mRNA_dw20k	NA	LTCONS_00006632	NM_016545	chr1	181063036	181063722	chr1	181057638	181059979
cis_mRNA_overlap	mRNA-CompleteIn-LncIntron	LTCONS_00006795	NM_022375	chr1	186342598	186394348	chr1	186369704	186370587
cis_mRNA_dw20k	NA	LTCONS_00007034	NM_002838	chr1	198730169	198734393	chr1	198608098	198726605
cis_mRNA_overlap	Lnc-Overlap-mRNA	LTCONS_00007422	MTCONS_00007423	chr1	207494817	207534311	chr1	207494817	207534311
cis_mRNA_overlap	Lnc-Overlap-mRNA	LTCONS_00007422	NM_001300902	chr1	207494817	207534311	chr1	207494817	207514111
cis_mRNA_overlap	Lnc-Overlap-mRNA	LTCONS_00007542	MTCONS_00007545	chr1	209923828	209930068	chr1	209929377	209955668
cis_mRNA_overlap	Lnc-CompleteIn-	LTCONS_00008888	MTCONS_00008890	chr1	14407	29370	chr1	14407	29370
cis_mRNA_overlap	Lnc-AntiOverlap-mRNA	LTCONS_00009120	MTCONS_00000238	chr1	1565857	1567512	chr1	1567010	1570030
cis_mRNA_overlap	Lnc-Overlap-mRNA	LTCONS_00009135	NM_001290264	chr1	1592939	1603430	chr1	1592939	1624243
cis_mRNA_overlap	Lnc-Overlap-mRNA	LTCONS_00009151	MTCONS_00009146	chr1	1649498	1656004	chr1	1633532	1656004
cis_mRNA_overlap	Lnc-AntiOverlap-mRNA	LTCONS_00009238	MTCONS_00000303	chr1	2478907	2489070	chr1	2487804	2490838

cis_mRNA up10k	NA	LTCONS_00009238	MTCONS_00000310	chr1	2478907	2489070	chr1	2497849	2522908
cis_mRNA overlap	Lnc-Overlap-mRNA	LTCONS_00009492	NM_006786	chr1	7903912	7913551	chr1	7907672	7913104
cis_mRNA overlap	Lnc-Overlap-mRNA	LTCONS_00009495	NM_021995	chr1	7907672	7973340	chr1	7907672	7913551
cis_mRNA overlap	Lnc-Overlap-mRNA	LTCONS_00009519	MTCONS_00009530	chr1	8411138	8484665	chr1	8476833	8586311
cis_mRNA _overlap	Lnc-AntiCompleteIn-mRNAExon	LTCONS_00009679	MTCONS_00000758	chr1	12208910	12213411	chr1	12185419	12213947
cis_mRNA _overlap	Lnc-AntiOverlap-mRNA	LTCONS_00010110	NM_044472	chr1	22390699	22485051	chr1	22379120	22417296
cis_mRNA _overlap	Lnc-AntiOverlap-mRNA	LTCONS_00010295	MTCONS_00001332	chr1	25629051	25664726	chr1	25598893	25688852
cis_mRNA up10k	NA	LTCONS_00010301	NM_014313	chr1	25657572	25664735	chr1	25664789	25688852
cis_mRNA _overlap	Lnc-AntiCompleteIn-mRNAIntron	LTCONS_00010361	NM_001243564	chr1	26780140	26794028	chr1	26758773	26797795
cis_mRNA dw20k	NA	LTCONS_00010461	NM_178191	chr1	28566020	28573670	chr1	28562602	28564616
cis_mRNA dw20k	NA	LTCONS_00010465	NM_178191	chr1	28569158	28573670	chr1	28562602	28564616
cis_mRNA dw20k	NA	LTCONS_00010629	MTCONS_00001751	chr1	32679885	32681517	chr1	32645345	32663886
cis_mRNA dw20k	NA	LTCONS_00010629	NM_019118	chr1	32679885	32681517	chr1	32681798	32687972
cis_mRNA overlap	Lnc-CompleteIn-	LTCONS_00010660	NM_001308139	chr1	33086817	33116191	chr1	33086817	33116191
cis_mRNA overlap	Lnc-Overlap-mRNA	LTCONS_00010748	MTCONS_00010742	chr1	35481363	35497569	chr1	35451767	35497569

cis_mRNA_overlap	mRNA-CompleteIn-LncExon	LTCONS_00010748	MTCONS_00010747	chr1	35481363	35497569	chr1	35481363	35497569
cis_mRNA dw20k	NA	LTCONS_00010847	NM_013285	chr1	38014208	38019945	chr1	38032413	38061586
tran	NA	LTCONS_0001100	MTCONS_0019989	chr1	40952240	40974344	chr9	35084238	35097583
tran	NA	LTCONS_0001100	NM_153498	chr1	40952240	40974344	chr10	12391542	12877545
cis_mRNA overlap	Lnc-Overlap-mRNA	LTCONS_00011064	MTCONS_00011063	chr1	42054000	42127875	chr1	42017421	42130116
cis_mRNA up10k	NA	LTCONS_00011220	NM_001013632	chr1	45273191	45274156	chr1	45271582	45272957
cis_mRNA up10k	NA	LTCONS_00011716	NM_002228	chr1	59250082	59250680	chr1	59246463	59249785
cis_mRNA overlap	Lnc-Overlap-mRNA	LTCONS_00011988	MTCONS_00011994	chr1	71528974	71535915	chr1	71534099	71546972
cis_mRNA_overlap	mRNA-CompleteIn-LncExon	LTCONS_00012592	NM_001993	chr1	94994732	95007413	chr1	94994732	95007413
cis_mRNA overlap	Lnc-Overlap-mRNA	LTCONS_00012993	MTCONS_00012994	chr1	111738227	111743325	chr1	111738227	111747160
cis_mRNA overlap	Lnc-Overlap-mRNA	LTCONS_00012993	NM_024901	chr1	111738227	111743325	chr1	111728591	111743325
cis_mRNA overlap	Lnc-Overlap-mRNA	LTCONS_00013556	MTCONS_00013557	chr1	150532678	150545373	chr1	150532678	150552214
cis_mRNA dw20k	NA	LTCONS_00013565	NM_207044	chr1	150577184	150588776	chr1	150595754	150602098
cis_mRNA overlap	Lnc-Overlap-mRNA	LTCONS_00013613	NM_001038707	chr1	151023447	151043079	chr1	151023447	151032125
cis_mRNA up10k	NA	LTCONS_00013613	NM_144618	chr1	151023447	151043079	chr1	151043080	151091007
cis_mRNA_overlap	mRNA-CompleteIn-LncExon	LTCONS_00014152	NM_001161442	chr1	156774583	156788750	chr1	156776035	156786640
cis_mRNA dw20k	NA	LTCONS_00014159	NM_005973	chr1	156782521	156785530	chr1	156737274	156770609

tran	NA	LTCONS_0001425	MTCONS_0009079	chr1	158856378	158925522	chr19	20946830	20993757
cis_mRNA dw20k	NA	LTCONS_00015048	NM_001200050	chr1	182806324	182811675	chr1	182758584	182799519
cis_mRNA up10k	NA	LTCONS_00015533	NM_002646	chr1	204463189	204465254	chr1	204391758	204459474
cis_mRNA dw20k	NA	LTCONS_00016303	MTCONS_00016306	chr1	226029673	226029970	chr1	226033233	226070420
cis_mRNA dw20k	NA	LTCONS_00016382	NM_024319	chr1	228265571	228270212	chr1	228288428	228291163
cis_mRNA overlap	Lnc-CompleteIn-	LTCONS_00016638	NM_001077397	chr1	234743709	234744092	chr1	234740015	234745271
cis_mRNA _overlap	Lnc-AntiOverlap-mRNA	LTCONS_00016773	MTCONS_00008564	chr1	237947743	237963221	chr1	237946596	237997288
cis_mRNA dw20k	NA	LTCONS_00017183	MTCONS_00000758	chr1	12220501	12225556	chr1	12185419	12213947
cis_mRNA dw20k	NA	LTCONS_00017184	MTCONS_00000758	chr1	12225672	12226751	chr1	12185419	12213947
cis_mRNA dw20k	NA	LTCONS_00017251	NM_000478	chr1	21909218	21910583	chr1	21835851	21904905
cis_mRNA up10k	NA	LTCONS_00017634	NM_001006605	chr1	93427459	93428659	chr1	93307717	93427079
cis_mRNA up10k	NA	LTCONS_00017731	NM_000757	chr1	110448449	110450008	chr1	110453233	110473616
cis_mRNA up10k	NA	LTCONS_00017764	NM_001142782	chr1	113933138	113933405	chr1	113933475	114228545
cis_mRNA dw20k	NA	LTCONS_00017773	MTCONS_00004877	chr1	116615697	116616384	chr1	116519119	116612675
cis_mRNA up10k	NA	LTCONS_00017866	NM_012437	chr1	153621431	153623500	chr1	153631130	153634328
cis_mRNA dw20k	NA	LTCONS_00017885	MTCONS_00014113	chr1	156427898	156428731	chr1	156432660	156471490
cis_mRNA dw20k	NA	LTCONS_00017917	MTCONS_00005899	chr1	159788789	159790126	chr1	159770250	159786047

cis_mRNA dw20k	NA	LTCONS_00017917	MTCONS_00005900	chr1	159788789	159790126	chr1	159770301	159786047
cis_mRNA dw20k	NA	LTCONS_00018208	NM_002393	chr1	204537250	204538992	chr1	204485507	204527248
cis_mRNA dw20k	NA	LTCONS_00018236	NM_014002	chr1	206674808	206675889	chr1	206643586	206670223
cis_mRNA up10k	NA	LTCONS_00019275	NM_182543	chr10	18940572	18966940	chr10	18834136	18940566
cis_mRNA overlap	Lnc-Overlap-mRNA	LTCONS_00019677	NM_001174094	chr10	31608101	31645095	chr10	31610064	31818742
cis_mRNA _overlap	Lnc-AntiOverlap-mRNA	LTCONS_00020811	MTCONS_00025259	chr10	75242348	75268422	chr10	75196186	75255282
cis_mRNA overlap	Lnc-Overlap-mRNA	LTCONS_00021114	MTCONS_00021111	chr10	81107600	81115089	chr10	81107220	81112483
cis_mRNA up10k	NA	LTCONS_00021407	MTCONS_00021409	chr10	90520415	90520842	chr10	90521163	90528160
cis_mRNA dw20k	NA	LTCONS_00021670	NM_001776	chr10	97642500	97654726	chr10	97515673	97637023
cis_mRNA overlap	Lnc-Overlap-mRNA	LTCONS_00021678	MTCONS_00021676	chr10	97803159	97820625	chr10	97803159	97820625
cis_mRNA overlap	Lnc-Overlap-mRNA	LTCONS_00022008	MTCONS_00022007	chr10	104153867	104162286	chr10	104153867	104162286
cis_mRNA _overlap	mRNA-CompleteIn-LncExon	LTCONS_00023075	NM_014468	chr10	135049407	135055434	chr10	135051408	135055434
cis_mRNA overlap	Lnc-Overlap-mRNA	LTCONS_00023162	MTCONS_00023158	chr10	1256198	1282545	chr10	1223253	1282544
cis_mRNA overlap	Lnc-Overlap-mRNA	LTCONS_00023168	MTCONS_00023158	chr10	1262598	1282583	chr10	1223253	1282544
cis_mRNA _overlap	Lnc-AntiOverlap-mRNA	LTCONS_00023534	MTCONS_00018996	chr10	8095610	8100256	chr10	8096667	8117311
cis_mRNA up10k	NA	LTCONS_00023844	NM_182543	chr10	18946403	18948245	chr10	18834136	18940566

tran	NA	LTCONS_0002503	MTCONS_0003253	chr10	69524382	69525325	chr11	123986111	124017618
cis_mRNA overlap	Lnc-Overlap-mRNA	LTCONS_00025251	NM_001024593	chr10	75178239	75188417	chr10	75183337	75193319
cis_mRNA overlap	Lnc-Overlap-mRNA	LTCONS_00025271	NM_001144000	chr10	75430506	75441654	chr10	75434033	75457554
cis_mRNA dw20k	NA	LTCONS_00025992	NM_012083	chr10	99077065	99078992	chr10	99092254	99094458
cis_mRNA up10k	NA	LTCONS_00025992	MTCONS_00021731	chr10	99077065	99078992	chr10	99079022	99082661
cis_mRNA up10k	NA	LTCONS_00025992	NM_005479	chr10	99077065	99078992	chr10	99079022	99081672
tran	NA	LTCONS_0002599	MTCONS_0007231	chr10	99077065	99078992	chr16	3292028	3306627
tran	NA	LTCONS_0002599	NM_001031701	chr10	99077065	99078992	chr12	104166081	104234975
tran	NA	LTCONS_0002599	NM_153713	chr10	99077065	99078992	chr1	145477066	145501668
tran	NA	LTCONS_0002599	NM_173462	chr10	99077065	99078992	chr14	73704205	73741347
cis_mRNA dw20k	NA	LTCONS_00026411	NM_005962	chr10	112052798	112064707	chr10	111985762	112047123
cis_mRNA overlap	Lnc-CompleteIn-	LTCONS_00026690	MTCONS_00026686	chr10	121348910	121354188	chr10	121332978	121356541
cis_mRNA _overlap	Lnc-AntiOverlap-mRNA	LTCONS_00026695	NM_014937	chr10	121430230	121486368	chr10	121485559	121588662
cis_mRNA overlap	Lnc-CompleteIn-	LTCONS_00026829	NM_001270765	chr10	125842659	125851940	chr10	125779169	125853123
cis_mRNA up10k	NA	LTCONS_00027238	MTCONS_00023292	chr10	3827633	3830102	chr10	3818188	3827473
cis_mRNA up10k	NA	LTCONS_00027238	MTCONS_00023293	chr10	3827633	3830102	chr10	3818188	3827473
cis_mRNA up10k	NA	LTCONS_00027519	NM_174890	chr10	46173126	46173758	chr10	46110949	46168180
cis_mRNA dw20k	NA	LTCONS_00027875	MTCONS_00022336	chr10	114930079	114932444	chr10	114710009	114915765
cis_mRNA dw20k	NA	LTCONS_00027875	NM_001146283	chr10	114930079	114932444	chr10	114710009	114927436
tran	NA	LTCONS_0002796	MTCONS_0007775	chr10	126480616	126481454	chr17	42084349	42088557

cis_mRNA_up10k	NA	LTCONS_00028049	MTCONS_00028059	chr11	287007	288987	chr11	289418	295688
cis_mRNA_up10k	NA	LTCONS_00028052	MTCONS_00028059	chr11	287265	288987	chr11	289418	295688
cis_mRNA_up10k	NA	LTCONS_00028052	MTCONS_00028060	chr11	287265	288987	chr11	289418	295688
cis_mRNA_overlap	Lnc-AntiOverlap-mRNA	LTCONS_00028146	MTCONS_00033061	chr11	777489	786722	chr11	766694	777502
cis_mRNA_dw20k	NA	LTCONS_00028338	NM_005969	chr11	2920951	2946476	chr11	2965660	3013607
cis_mRNA_overlap	mRNA-CompleteIn-LncExon	LTCONS_00028338	NM_002555	chr11	2920951	2946476	chr11	2923512	2946476
cis_mRNA_overlap	Lnc-Overlap-mRNA	LTCONS_00028428	MTCONS_00028423	chr11	4415042	4543398	chr11	4415035	4503536
cis_mRNA_overlap	Lnc-AntiOverlap-mRNA	LTCONS_00028614	NM_001286095	chr11	8985991	9000526	chr11	8968748	8986558
tran	NA	LTCONS_0002861	NM_001127395	chr11	8985991	9000526	chr2	208473839	208490055
tran	NA	LTCONS_0002861	NM_152384	chr11	8985991	9000526	chr2	170336006	170363165
cis_mRNA_dw20k	NA	LTCONS_00030134	MTCONS_00030122	chr11	61739648	61740189	chr11	61717302	61732844
cis_mRNA_up10k	NA	LTCONS_00030134	NM_002032	chr11	61739648	61740189	chr11	61731757	61735132
cis_mRNA_overlap	Lnc-AntiOverlap-mRNA	LTCONS_00030378	NM_006795	chr11	64627812	64641896	chr11	64620199	64646240
cis_mRNA_dw20k	NA	LTCONS_00030626	MTCONS_00030615	chr11	66414555	66432457	chr11	66384053	66408651
cis_mRNA_dw20k	NA	LTCONS_00030626	NM_002896	chr11	66414555	66432457	chr11	66406088	66413944
cis_mRNA_dw20k	NA	LTCONS_00030626	MTCONS_00030620	chr11	66414555	66432457	chr11	66391219	66405613

cis_mRNA dw20k	NA	LTCONS_00030626	NM_001286135	chr11	66414555	66432457	chr11	66432465	66445392
cis_mRNA up10k	NA	LTCONS_00030679	NM_207354	chr11	67056047	67056467	chr11	67056762	67069955
cis_mRNA overlap	Lnc-Overlap-mRNA	LTCONS_00031409	MTCONS_00031413	chr11	82868137	82896835	chr11	82869401	82896835
cis_mRNA dw20k	NA	LTCONS_00032310	MTCONS_00032307	chr11	118876666	118886502	chr11	118868843	118873672
cis_mRNA overlap	Lnc-Overlap-mRNA	LTCONS_00032773	MTCONS_00032771	chr11	129726741	129733498	chr11	129685741	129733498
cis_mRNA _overlap	mRNA-CompleteIn-LncExon	LTCONS_00033202	NM_001142946	chr11	2307213	2323143	chr11	2317507	2323143
cis_mRNA up10k	NA	LTCONS_00033202	MTCONS_00028314	chr11	2307213	2323143	chr11	2323243	2349904
cis_mRNA up10k	NA	LTCONS_00033202	MTCONS_00028315	chr11	2307213	2323143	chr11	2323243	2349904
cis_mRNA up10k	NA	LTCONS_00033202	NM_139022	chr11	2307213	2323143	chr11	2323243	2339430
cis_mRNA up10k	NA	LTCONS_00033205	MTCONS_00028314	chr11	2307213	2323143	chr11	2323243	2349904
cis_mRNA dw20k	NA	LTCONS_00033444	MTCONS_00033445	chr11	6726265	6730727	chr11	6731362	6767699
cis_mRNA _overlap	Lnc-AntiOverlap-mRNA	LTCONS_00034046	NM_181706	chr11	31453946	31531184	chr11	31391377	31454382
cis_mRNA dw20k	NA	LTCONS_00034216	NM_004620	chr11	36498312	36503302	chr11	36505317	36531863
cis_mRNA _overlap	Lnc-AntiOverlap-mRNA	LTCONS_00035513	MTCONS_00030900	chr11	70230722	70244594	chr11	70243820	70282690
cis_mRNA up10k	NA	LTCONS_00035513	NM_005231	chr11	70230722	70244594	chr11	70244612	70282690
cis_mRNA up10k	NA	LTCONS_00035513	NM_138565	chr11	70230722	70244594	chr11	70244612	70282690

cis_mRNA overlap	Lnc-Overlap-mRNA	LTCONS_00036206	MTCONS_00036199	chr11	94919929	94964246	chr11	94898677	94964246
cis_mRNA _overlap	mRNA-CompleteIn-LncExon	LTCONS_00036227	MTCONS_00036225	chr11	95502106	95523610	chr11	95502106	95522954
cis_mRNA dw20k	NA	LTCONS_00037490	NM_006074	chr11	5738880	5740283	chr11	5710817	5732093
cis_mRNA dw20k	NA	LTCONS_00037526	NM_001282666	chr11	12288090	12290090	chr11	12183626	12285337
cis_mRNA dw20k	NA	LTCONS_00037527	MTCONS_00028745	chr11	12290518	12292486	chr11	12132123	12287278
cis_mRNA dw20k	NA	LTCONS_00037616	MTCONS_00029405	chr11	36489154	36491497	chr11	36397948	36486754
cis_mRNA dw20k	NA	LTCONS_00037740	NM_005125	chr11	66377149	66377608	chr11	66360690	66373490
cis_mRNA dw20k	NA	LTCONS_00037741	MTCONS_00030672	chr11	67031466	67033845	chr11	67007506	67025550
cis_mRNA up10k	NA	LTCONS_00037751	MTCONS_00035417	chr11	67981452	67983462	chr11	67922330	67981326
cis_mRNA overlap	Lnc-CompleteIn-	LTCONS_00038721	MTCONS_00038714	chr12	9265134	9265957	chr12	9142221	9368017
cis_mRNA overlap	Lnc-CompleteIn-	LTCONS_00038722	MTCONS_00038714	chr12	9268557	9270256	chr12	9142221	9368017
cis_mRNA dw20k	NA	LTCONS_00038799	MTCONS_00038790	chr12	9852353	9854788	chr12	9822304	9852151
cis_mRNA overlap	Lnc-Overlap-mRNA	LTCONS_00038817	MTCONS_00038825	chr12	10163226	10170548	chr12	10163226	10172432
cis_mRNA overlap	Lnc-Overlap-mRNA	LTCONS_00038817	NM_001129998	chr12	10163226	10170548	chr12	10163226	10171399
cis_mRNA overlap	Lnc-Overlap-mRNA	LTCONS_00038844	NM_002262	chr12	10457050	10475046	chr12	10460417	10469850
cis_mRNA overlap	Lnc-Overlap-mRNA	LTCONS_00038844	NM_007334	chr12	10457050	10475046	chr12	10460417	10469850

cis_mRNA_overlap	Lnc-AntiOverlap-mRNA	LTCONS_00038861	MTCONS_00043740	chr12	10574587	10594568	chr12	10564914	10588592
cis_mRNA_overlap	Lnc-AntiOverlap-mRNA	LTCONS_00038861	MTCONS_00043741	chr12	10574587	10594568	chr12	10564914	10588592
cis_mRNA_overlap	mRNA-AntiCompleteIn-LncIntron	LTCONS_00038861	MTCONS_00043743	chr12	10574587	10594568	chr12	10583205	10588592
cis_mRNA_overlap	mRNA-AntiCompleteIn-LncIntron	LTCONS_00038861	MTCONS_00043745	chr12	10574587	10594568	chr12	10583205	10588596
cis_mRNA up10k	NA	LTCONS_00038861	NM_002261	chr12	10574587	10594568	chr12	10564914	10573194
cis_mRNA dw20k	NA	LTCONS_00039473	NM_018169	chr12	32149201	32160369	chr12	32112353	32146041
cis_mRNA dw20k	NA	LTCONS_00040005	NM_030809	chr12	51442082	51454207	chr12	51454988	51477454
cis_mRNA overlap	Lnc-Overlap-mRNA	LTCONS_00040005	NM_015416	chr12	51442082	51454207	chr12	51442082	51454207
cis_mRNA overlap	Lnc-CompleteIn-	LTCONS_00040091	NM_001202234	chr12	52438768	52441658	chr12	52431499	52453291
cis_mRNA up10k	NA	LTCONS_00040091	MTCONS_00040096	chr12	52438768	52441658	chr12	52443418	52453677
cis_mRNA up10k	NA	LTCONS_00040091	MTCONS_00040100	chr12	52438768	52441658	chr12	52445186	52453677
cis_mRNA up10k	NA	LTCONS_00040091	NM_002135	chr12	52438768	52441658	chr12	52445186	52453291
cis_mRNA up10k	NA	LTCONS_00040197	MTCONS_00044976	chr12	53894705	53900215	chr12	53874276	53893444
cis_mRNA up10k	NA	LTCONS_00040197	NM_001193511	chr12	53894705	53900215	chr12	53874276	53893444
cis_mRNA dw20k	NA	LTCONS_00040280	NM_005337	chr12	54940863	54952677	chr12	54891495	54936899

cis_mRNA overlap	Lnc-Overlap-mRNA	LTCONS_00041068	MTCONS_00041066	chr12	77235530	77248757	chr12	77157854	77248757
cis_mRNA overlap	Lnc-Overlap-mRNA	LTCONS_00041891	NM_013300	chr12	110906232	110929321	chr12	110906232	110928192
cis_mRNA overlap	Lnc-CompleteIn-	LTCONS_00041997	MTCONS_00041994	chr12	113593221	113593656	chr12	113587663	113599052
cis_mRNA overlap	Lnc-Overlap-mRNA	LTCONS_00043421	NM_032489	chr12	6747238	6748507	chr12	6747242	6756580
cis_mRNA overlap	Lnc-Overlap-mRNA	LTCONS_00043670	NM_005127	chr12	10007086	10010075	chr12	10004968	10022458
cis_mRNA dw20k	NA	LTCONS_00043736	MTCONS_00043740	chr12	10524952	10563454	chr12	10564914	10588592
cis_mRNA dw20k	NA	LTCONS_00043736	MTCONS_00043741	chr12	10524952	10563454	chr12	10564914	10588592
cis_mRNA dw20k	NA	LTCONS_00043736	NM_002261	chr12	10524952	10563454	chr12	10564914	10573194
cis_mRNA dw20k	NA	LTCONS_00043736	MTCONS_00043745	chr12	10524952	10563454	chr12	10583205	10588596
cis_mRNA dw20k	NA	LTCONS_00043736	NM_007333	chr12	10524952	10563454	chr12	10568183	10573194
cis_mRNA overlap	Lnc-Overlap-mRNA	LTCONS_00043736	NM_013431	chr12	10524952	10563454	chr12	10559983	10562356
cis_mRNA overlap	Lnc-Overlap-mRNA	LTCONS_00043756	MTCONS_00043760	chr12	10740298	10752434	chr12	10747602	10752434
cis_mRNA_overlap	mRNA-CompleteIn-LncIntron	LTCONS_00043785	NM_023920	chr12	11038086	11320809	chr12	11060525	11062161
cis_mRNA_overlap	mRNA-CompleteIn-LncIntron	LTCONS_00043785	NM_176887	chr12	11038086	11320809	chr12	11213964	11214893
cis_mRNA_overlap	mRNA-CompleteIn-LncIntron	LTCONS_00043785	NM_176888	chr12	11038086	11320809	chr12	11174218	11175219

cis_mRNA_overlap	mRNA-CompleteIn-LncIntron	LTCONS_00043785	NM_176889	chr12	11038086	11320809	chr12	11148561	11150474
cis_mRNA_overlap	mRNA-CompleteIn-LncIntron	LTCONS_00043792	NM_176887	chr12	11124630	11278217	chr12	11213964	11214893
cis_mRNA_overlap	mRNA-CompleteIn-LncIntron	LTCONS_00043802	NM_176887	chr12	11207703	11243586	chr12	11213964	11214893
cis_mRNA_overlap	Lnc-AntiCompleteIn-mRNAIntron	LTCONS_00044552	NM_138371	chr12	47514534	47540775	chr12	47473386	47630446
cis_mRNA_overlap	mRNA-CompleteIn-LncExon	LTCONS_00044658	MTCONS_00044659	chr12	49358798	49380857	chr12	49358798	49380857
cis_mRNA_up10k	NA	LTCONS_00044662	NM_003394	chr12	49365860	49366708	chr12	49359123	49365641
cis_mRNA_overlap	Lnc-Overlap-mRNA	LTCONS_00044808	NM_002702	chr12	51590327	51606256	chr12	51580719	51591950
cis_mRNA_up10k	NA	LTCONS_00044980	NM_001193511	chr12	53900238	53902034	chr12	53874276	53893444
cis_mRNA_overlap	Lnc-AntiOverlap-mRNA	LTCONS_00045429	NM_006313	chr12	62639633	62656200	chr12	62654121	62803501
cis_mRNA_overlap	Lnc-CompleteIn-	LTCONS_00045791	NM_001286548	chr12	75757010	75759343	chr12	75671001	75784702
cis_mRNA_up10k	NA	LTCONS_00047070	MTCONS_00042350	chr12	122233165	122240510	chr12	122241894	122271045
cis_mRNA_overlap	mRNA-CompleteIn-LncExon	LTCONS_00047141	MTCONS_00047139	chr12	122989190	123011560	chr12	122989190	123011560
cis_mRNA_dw20k	NA	LTCONS_00047756	MTCONS_00038714	chr12	9378517	9383821	chr12	9142221	9368017
cis_mRNA_up10k	NA	LTCONS_00048459	NM_207311	chr12	120426894	120427508	chr12	120427648	120532299

tran	NA	LTCONS_0004892	MTCONS_0009079	chr13	30221056	30230021	chr19	20946830	20993757
cis_mRNA overlap	Lnc-Overlap-mRNA	LTCONS_00049642	NM_030911	chr13	49822047	49867622	chr13	49822047	49867622
cis_mRNA_overlap	Lnc-AntiOverlap-mRNA	LTCONS_00050642	NM_001105515	chr13	95945017	95963239	chr13	95748025	95953700
cis_mRNA overlap	Lnc-Overlap-mRNA	LTCONS_00051171	NM_001008895	chr13	113862507	113884538	chr13	113863819	113919392
cis_mRNA overlap	Lnc-Overlap-mRNA	LTCONS_00051171	NM_003589	chr13	113862507	113884538	chr13	113863086	113919392
cis_mRNA overlap	Lnc-Overlap-mRNA	LTCONS_00051784	MTCONS_00051780	chr13	31709111	31736525	chr13	31709111	31726774
cis_mRNA overlap	Lnc-Overlap-mRNA	LTCONS_00051784	MTCONS_00051785	chr13	31709111	31736525	chr13	31709111	31736525
cis_mRNA overlap	Lnc-Overlap-mRNA	LTCONS_00051784	MTCONS_00051786	chr13	31709111	31736525	chr13	31709111	31736525
cis_mRNA overlap	Lnc-Overlap-mRNA	LTCONS_00051784	MTCONS_00051790	chr13	31709111	31736525	chr13	31716655	31736525
cis_mRNA overlap	Lnc-Overlap-mRNA	LTCONS_00051791	MTCONS_00051780	chr13	31723572	31736525	chr13	31709111	31726774
cis_mRNA overlap	Lnc-Overlap-mRNA	LTCONS_00051791	MTCONS_00051785	chr13	31723572	31736525	chr13	31709111	31736525
cis_mRNA overlap	Lnc-Overlap-mRNA	LTCONS_00051791	MTCONS_00051786	chr13	31723572	31736525	chr13	31709111	31736525
cis_mRNA overlap	Lnc-Overlap-mRNA	LTCONS_00051791	MTCONS_00051790	chr13	31723572	31736525	chr13	31716655	31736525
cis_mRNA dw20k	NA	LTCONS_00052072	NM_014252	chr13	41396432	41495916	chr13	41363547	41386596
cis_mRNA dw20k	NA	LTCONS_00052389	NM_000321	chr13	49063099	49067303	chr13	48877883	49056026
cis_mRNA overlap	Lnc-Overlap-mRNA	LTCONS_00052536	NM_024705	chr13	52368015	52399363	chr13	52342129	52378298
cis_mRNA up10k	NA	LTCONS_00053838	MTCONS_00051785	chr13	31736701	31737692	chr13	31709111	31736525

cis_mRNA up10k	NA	LTCONS_00053838	MTCONS_00051786	chr13	31736701	31737692	chr13	31709111	31736525
cis_mRNA up10k	NA	LTCONS_00053838	MTCONS_00051790	chr13	31736701	31737692	chr13	31716655	31736525
cis_mRNA up10k	NA	LTCONS_00053838	MTCONS_00051780	chr13	31736701	31737692	chr13	31709111	31726774
cis_mRNA overlap	Lnc-CompleteIn-	LTCONS_00054649	MTCONS_00054648	chr14	22996331	22996697	chr14	22994433	23021103
cis_mRNA up10k	NA	LTCONS_00054649	MTCONS_00054650	chr14	22996331	22996697	chr14	22997994	23021103
cis_mRNA up10k	NA	LTCONS_00054649	MTCONS_00054652	chr14	22996331	22996697	chr14	23000289	23021103
cis_mRNA overlap	Lnc-CompleteIn-	LTCONS_00054754	MTCONS_00054751	chr14	24025191	24037282	chr14	24025191	24037282
cis_mRNA _overlap	Lnc-AntiCompleteIn-mRNAIntron	LTCONS_00055463	NM_016350	chr14	51278940	51284569	chr14	51192547	51297839
cis_mRNA dw20k	NA	LTCONS_00055615	NM_080867	chr14	55518362	55525265	chr14	55493844	55516206
cis_mRNA overlap	Lnc-Overlap-mRNA	LTCONS_00055615	NM_144578	chr14	55518362	55525265	chr14	55518362	55536912
cis_mRNA up10k	NA	LTCONS_00055934	NM_145112	chr14	65574253	65585684	chr14	65541842	65569262
cis_mRNA up10k	NA	LTCONS_00055934	NM_145113	chr14	65574253	65585684	chr14	65541842	65569262
cis_mRNA dw20k	NA	LTCONS_00055959	MTCONS_00055956	chr14	66215897	66217566	chr14	65879541	66213294
cis_mRNA dw20k	NA	LTCONS_00056081	NM_001039465	chr14	70238832	70242553	chr14	70233829	70238722
cis_mRNA overlap	Lnc-Overlap-mRNA	LTCONS_00056198	MTCONS_00056195	chr14	73566695	73588076	chr14	73525221	73588076
cis_mRNA overlap	Lnc-Overlap-mRNA	LTCONS_00057104	MTCONS_00057106	chr14	99947739	99959747	chr14	99947739	99977852
cis_mRNA up10k	NA	LTCONS_00057925	NM_001199864	chr14	23765130	23769136	chr14	23775971	23795394

cis_mRNA up10k	NA	LTCONS_00058931	MTCONS_00055738	chr14	58732083	58764855	chr14	58765222	58840451
cis_mRNA up10k	NA	LTCONS_00058933	MTCONS_00055738	chr14	58732083	58764855	chr14	58765222	58840451
cis_mRNA dw20k	NA	LTCONS_00059220	NM_182526	chr14	67933695	67934779	chr14	67936983	67982021
cis_mRNA up10k	NA	LTCONS_00059573	NM_024496	chr14	77498344	77501609	chr14	77490886	77495042
tran	NA	LTCONS_0005961	MTCONS_0009437	chr14	78123872	78124683	chr19	11487649	11495018
tran	NA	LTCONS_0005961	NM_001080825	chr14	78123872	78124683	chr12	122150658	122219974
tran	NA	LTCONS_0005961	NM_001163446	chr14	78123872	78124683	chr7	129932974	129964020
tran	NA	LTCONS_0005961	NM_005511	chr14	78123872	78124683	chr9	5890909	5909822
cis_mRNA overlap	Lnc-Overlap-mRNA	LTCONS_00060179	NM_001144995	chr14	99977603	99983058	chr14	99977603	100070727
cis_mRNA overlap	Lnc-Overlap-mRNA	LTCONS_00060376	NM_001100119	chr14	104161517	104166977	chr14	104163954	104181823
cis_mRNA overlap	Lnc-Overlap-mRNA	LTCONS_00060463	NM_001278695	chr14	105522969	105533770	chr14	105515726	105531887
cis_mRNA overlap	Lnc-CompleteIn-	LTCONS_00060518	MTCONS_00060513	chr14	106173801	106174901	chr14	106134592	106209253
cis_mRNA up10k	NA	LTCONS_00060529	MTCONS_00060528	chr14	106322224	106328007	chr14	106318066	106322110
cis_mRNA overlap	Lnc-CompleteIn-	LTCONS_00060598	MTCONS_00060524	chr14	106829588	106830329	chr14	106235440	106878313
cis_mRNA overlap	Lnc-Overlap-mRNA	LTCONS_00060604	MTCONS_00060606	chr14	106899262	106926745	chr14	106925902	106926751
cis_mRNA overlap	Lnc-Overlap-mRNA	LTCONS_00060629	MTCONS_00060631	chr14	107122312	107131561	chr14	107131036	107131584
cis_mRNA dw20k	NA	LTCONS_00060907	MTCONS_00055600	chr14	55263401	55264695	chr14	55076321	55260326
cis_mRNA dw20k	NA	LTCONS_00060925	NM_018229	chr14	57759998	57766027	chr14	57735606	57756797
cis_mRNA dw20k	NA	LTCONS_00061014	NM_000021	chr14	73695438	73696319	chr14	73603143	73690399

cis_mRNA_up10k	NA	LTCONS_00061026	MTCONS_00056319	chr14	75741716	75743182	chr14	75745481	75748937
cis_mRNA_up10k	NA	LTCONS_00061026	NM_005252	chr14	75741716	75743182	chr14	75745481	75748937
cis_mRNA_dw20k	NA	LTCONS_00061027	MTCONS_00056319	chr14	75754158	75755900	chr14	75745481	75748937
cis_mRNA_dw20k	NA	LTCONS_00061028	MTCONS_00056319	chr14	75765958	75766727	chr14	75745481	75748937
cis_mRNA_dw20k	NA	LTCONS_00061137	NM_001127700	chr14	94833907	94835862	chr14	94843084	94857029
cis_mRNA_dw20k	NA	LTCONS_00061224	NM_002719	chr14	102399072	102401961	chr14	102276140	102394328
cis_mRNA_overlap	Lnc-CompleteIn-	LTCONS_00062472	NM_012388	chr15	45879574	45901914	chr15	45879321	45901914
cis_mRNA_overlap	mRNA-CompleteIn-LncExon	LTCONS_00062726	NM_004855	chr15	55611130	55647846	chr15	55611133	55647846
cis_mRNA_overlap	Lnc-Overlap-mRNA	LTCONS_00063020	MTCONS_00063019	chr15	64443916	64449680	chr15	64443916	64449680
cis_mRNA_overlap	Lnc-Overlap-mRNA	LTCONS_00063048	NM_182703	chr15	65238543	65243251	chr15	65204101	65251041
cis_mRNA_dw20k	NA	LTCONS_00063653	NM_006791	chr15	79190707	79214091	chr15	79165123	79190081
cis_mRNA_dw20k	NA	LTCONS_00063653	NM_206839	chr15	79190707	79214091	chr15	79165123	79190081
cis_mRNA_up10k	NA	LTCONS_00064294	NM_001042572	chr15	93425937	93441977	chr15	93443551	93492463
cis_mRNA_overlap	Lnc-CompleteIn-	LTCONS_00064311	NM_001042572	chr15	93445821	93446721	chr15	93443551	93492463
cis_mRNA_overlap	Lnc-Overlap-mRNA	LTCONS_00064366	MTCONS_00064368	chr15	94773053	94838699	chr15	94774641	94890951
cis_mRNA_overlap	Lnc-Overlap-mRNA	LTCONS_00064366	MTCONS_00064372	chr15	94773053	94838699	chr15	94774787	95017210
cis_mRNA_up10k	NA	LTCONS_00064366	NM_018349	chr15	94773053	94838699	chr15	94841430	95027181

cis_mRNA overlap	Lnc-Overlap-mRNA	LTCONS_00064373	MTCONS_00064368	chr15	94774794	94786459	chr15	94774641	94890951
cis_mRNA overlap	Lnc-Overlap-mRNA	LTCONS_00064373	MTCONS_00064372	chr15	94774794	94786459	chr15	94774787	95017210
cis_mRNA overlap	Lnc-Overlap-mRNA	LTCONS_00064556	MTCONS_00064558	chr15	100106133	100203718	chr15	100106133	100240191
cis_mRNA overlap	Lnc-Overlap-mRNA	LTCONS_00064818	MTCONS_00064809	chr15	23338853	23378259	chr15	23281071	23378259
cis_mRNA up10k	NA	LTCONS_00064989	MTCONS_00064985	chr15	30714727	30723938	chr15	30692491	30712666
cis_mRNA up10k	NA	LTCONS_00065055	MTCONS_00065051	chr15	32756939	32758703	chr15	32733822	32754940
cis_mRNA overlap	Lnc-Overlap-mRNA	LTCONS_00065056	MTCONS_00065057	chr15	32782432	32828839	chr15	32784941	32792310
cis_mRNA overlap	Lnc-Overlap-mRNA	LTCONS_00065086	NM_001103184	chr15	33354616	33384905	chr15	33057745	33360233
cis_mRNA up10k	NA	LTCONS_00065320	NM_003246	chr15	39872621	39873146	chr15	39873280	39891122
cis_mRNA _overlap	Lnc-AntiOverlap-mRNA	LTCONS_00065545	NM_001199877	chr15	44086372	44092593	chr15	44069294	44088287
cis_mRNA dw20k	NA	LTCONS_00065562	MTCONS_00065573	chr15	44825691	44836438	chr15	44854894	45003532
tran	NA	LTCONS_0006556	NM_001318152	chr15	44825691	44836438	chr22	19420462	19423601
cis_mRNA dw20k	NA	LTCONS_00066181	NM_001195059	chr15	65167854	65172533	chr15	65134082	65160201
cis_mRNA _overlap	mRNA-CompleteIn-LncExon	LTCONS_00066417	MTCONS_00066409	chr15	70340130	70390256	chr15	70340130	70390256
cis_mRNA _overlap	Lnc-AntiOverlap-mRNA	LTCONS_00067063	MTCONS_00063894	chr15	85184726	85197574	chr15	85174682	85186244
cis_mRNA _overlap	Lnc-AntiCompleteIn-mRNAIntron	LTCONS_00067205	MTCONS_00064010	chr15	89179624	89181900	chr15	89178868	89199575

cis_mRNA up10k	NA	LTCONS_00067205	NM_001303236	chr15	89179624	89181900	chr15	89181974	89199575
cis_mRNA dw20k	NA	LTCONS_00067312	MTCONS_00064212	chr15	91474148	91475799	chr15	91445209	91465815
cis_mRNA _overlap	mRNA-CompleteIn-LncExon	LTCONS_00067312	NM_001286451	chr15	91474148	91475799	chr15	91474148	91475799
cis_mRNA _overlap	Lnc-AntiCompleteIn-mRNAIntron	LTCONS_00067381	NM_001271	chr15	93503445	93521366	chr15	93443551	93571237
tran	NA	LTCONS_0006782	MTCONS_0006648	chr15	22587257	22587915	chr15	72491337	72526033
tran	NA	LTCONS_0006782	MTCONS_0006354	chr15	22587257	22587915	chr15	76196200	76229582
tran	NA	LTCONS_0006782	MTCONS_0000960	chr15	22587257	22587915	chr1	10513711	10532613
tran	NA	LTCONS_0006782	MTCONS_0003061	chr15	22587257	22587915	chr11	66384053	66387692
tran	NA	LTCONS_0006782	MTCONS_0003070	chr15	22587257	22587915	chr11	67155110	67165883
tran	NA	LTCONS_0006782	MTCONS_0003253	chr15	22587257	22587915	chr11	123986111	124017618
tran	NA	LTCONS_0006782	MTCONS_0003307	chr15	22587257	22587915	chr11	799179	809935
tran	NA	LTCONS_0006782	MTCONS_0004464	chr15	22587257	22587915	chr12	49100394	49110781
tran	NA	LTCONS_0006782	MTCONS_0007314	chr15	22587257	22587915	chr16	30351916	30366682
tran	NA	LTCONS_0006782	MTCONS_0008412	chr15	22587257	22587915	chr17	79504395	79519433
tran	NA	LTCONS_0006782	MTCONS_0009341	chr15	22587257	22587915	chr19	58281020	58302958
tran	NA	LTCONS_0006782	MTCONS_0012476	chr15	22587257	22587915	chr22	21271714	21308037
tran	NA	LTCONS_0006782	MTCONS_0012798	chr15	22587257	22587915	chr22	39526779	39548538
tran	NA	LTCONS_0006782	MTCONS_0013155	chr15	22587257	22587915	chr3	101568358	101575244
tran	NA	LTCONS_0006782	MTCONS_0014142	chr15	22587257	22587915	chr4	2965230	2972037
tran	NA	LTCONS_0006782	MTCONS_0017990	chr15	22587257	22587915	chr7	142985308	143004789
tran	NA	LTCONS_0006782	MTCONS_0019001	chr15	22587257	22587915	chr8	145582217	145588184
tran	NA	LTCONS_0006782	MTCONS_0019074	chr15	22587257	22587915	chr8	23286665	23315244
tran	NA	LTCONS_0006782	NM_001024215	chr15	22587257	22587915	chr1	16090994	16101715
tran	NA	LTCONS_0006782	NM_001135993	chr15	22587257	22587915	chr18	21594384	21715574
tran	NA	LTCONS_0006782	NM_001277333	chr15	22587257	22587915	chr18	12093848	12129748
tran	NA	LTCONS_0006782	NM_002098	chr15	22587257	22587915	chr6	42151022	42162694
tran	NA	LTCONS_0006782	NM_004932	chr15	22587257	22587915	chr5	31193762	31329253
tran	NA	LTCONS_0006782	NM_005223	chr15	22587257	22587915	chr16	3702940	3708096
tran	NA	LTCONS_0006782	NM_005652	chr15	22587257	22587915	chr16	69389464	69419891

tran	NA	LTCONS_0006782	NM_013432	chr15	22587257	22587915	chr8	145654163	145669812
tran	NA	LTCONS_0006782	NM_014468	chr15	22587257	22587915	chr10	135051408	135055434
tran	NA	LTCONS_0006782	NM_014668	chr15	22587257	22587915	chr2	11674242	11782912
tran	NA	LTCONS_0006782	NM_015458	chr15	22587257	22587915	chr8	11142000	11185654
tran	NA	LTCONS_0006782	NM_016823	chr15	22587257	22587915	chr17	1324647	1359561
tran	NA	LTCONS_0006782	NM_017420	chr15	22587257	22587915	chr14	61176256	61190852
tran	NA	LTCONS_0006782	NM_020705	chr15	22587257	22587915	chr16	2525147	2555734
tran	NA	LTCONS_0006782	NM_022098	chr15	22587257	22587915	chr22	41253085	41328823
tran	NA	LTCONS_0006782	NM_024874	chr15	22587257	22587915	chr1	35899091	36023037
tran	NA	LTCONS_0006782	NM_024963	chr15	22587257	22587915	chr7	5515428	5553399
tran	NA	LTCONS_0006782	NM_030626	chr15	22587257	22587915	chr10	134145614	134195010
tran	NA	LTCONS_0006782	NM_032329	chr15	22587257	22587915	chr2	242641456	242668896
tran	NA	LTCONS_0006782	NM_153211	chr15	22587257	22587915	chr18	21572737	21715574
cis_mRNA_dw20k	NA	LTCONS_00068013	MTCONS_00063014	chr15	64441640	64442603	chr15	64388083	64436433
cis_mRNA_up10k	NA	LTCONS_00068103	NM_000745	chr15	78850780	78853113	chr15	78857862	78887611
cis_mRNA_up10k	NA	LTCONS_00068293	MTCONS_00071952	chr16	686392	688596	chr16	684427	686366
cis_mRNA_up10k	NA	LTCONS_00068295	NM_032366	chr16	691840	698476	chr16	684427	686366
cis_mRNA_up10k	NA	LTCONS_00068650	MTCONS_00072316	chr16	3313743	3325807	chr16	3292028	3306627
cis_mRNA_overlap	Lnc-Overlap-mRNA	LTCONS_00068673	NM_001317094	chr16	3503074	3538503	chr16	3507985	3536963
cis_mRNA_overlap	Lnc-AntiOverlap-mRNA	LTCONS_00068695	NM_016292	chr16	3661676	3708780	chr16	3708038	3767598
cis_mRNA_overlap	Lnc-Overlap-mRNA	LTCONS_00068695	NM_005223	chr16	3661676	3708780	chr16	3702940	3708096
cis_mRNA_up10k	NA	LTCONS_00068695	NM_032444	chr16	3661676	3708780	chr16	3631184	3661585
cis_mRNA_overlap	Lnc-Overlap-mRNA	LTCONS_00068696	NM_005223	chr16	3661676	3708780	chr16	3702940	3708096

cis_mRNA overlap	Lnc-Overlap-mRNA	LTCONS_00069305	MTCONS_00069299	chr16	24562135	24569789	chr16	24550908	24584183
tran	NA	LTCONS_0006948	NM_006985	chr16	29063228	29063552	chr16	15031300	15045931
cis_mRNA overlap	Lnc-Overlap-mRNA	LTCONS_00069611	NM_001193333	chr16	30196727	30197920	chr16	30194731	30200397
cis_mRNA overlap	Lnc-Overlap-mRNA	LTCONS_00069611	NM_007074	chr16	30196727	30197920	chr16	30194731	30200397
cis_mRNA dw20k	NA	LTCONS_00069845	NM_003414	chr16	31934157	31935670	chr16	31885079	31928629
cis_mRNA dw20k	NA	LTCONS_00070401	NM_005953	chr16	56659585	56670494	chr16	56642478	56643409
cis_mRNA dw20k	NA	LTCONS_00070401	MTCONS_00070398	chr16	56659585	56670494	chr16	56651375	56653250
cis_mRNA overlap	Lnc-Overlap-mRNA	LTCONS_00070401	NM_175617	chr16	56659585	56670494	chr16	56659585	56661025
cis_mRNA _overlap	mRNA-CompleteIn-LncIntron	LTCONS_00070401	MTCONS_00070402	chr16	56659585	56670494	chr16	56666534	56667898
cis_mRNA up10k	NA	LTCONS_00070401	NM_005946	chr16	56659585	56670494	chr16	56672578	56674000
cis_mRNA dw20k	NA	LTCONS_00070412	NM_001301267	chr16	56691855	56698877	chr16	56700647	56701977
cis_mRNA dw20k	NA	LTCONS_00070412	NM_005950	chr16	56691855	56698877	chr16	56700647	56701977
cis_mRNA dw20k	NA	LTCONS_00070412	NM_005946	chr16	56691855	56698877	chr16	56672578	56674000
cis_mRNA _overlap	mRNA-CompleteIn-LncExon	LTCONS_00070412	NM_001301272	chr16	56691855	56698877	chr16	56691855	56693215
cis_mRNA _overlap	mRNA-CompleteIn-LncExon	LTCONS_00070412	NM_005949	chr16	56691855	56698877	chr16	56691855	56693215
cis_mRNA up10k	NA	LTCONS_00070412	NM_005951	chr16	56691855	56698877	chr16	56703726	56705041
tran	NA	LTCONS_0007041	MTCONS_0007040	chr16	56691855	56698877	chr16	56666534	56667898

tran	NA	LTCONS_0007041	NM_176870	chr16	56691855	56698877 chr16	56666534	56667898
cis_mRNA dw20k	NA	LTCONS_00071231	NM_014940	chr16	77234711	77236298 chr16	77224816	77233543
cis_mRNA up10k	NA	LTCONS_00071622	MTCONS_00071629	chr16	87985038	87989956 chr16	87990130	88110924
cis_mRNA up10k	NA	LTCONS_00071764	NM_001256183	chr16	89557059	89567264 chr16	89334029	89556969
cis_mRNA dw20k	NA	LTCONS_00071903	NM_016310	chr16	75501	87037 chr16	96979	103632
cis_mRNA overlap	Lnc-CompleteIn-	LTCONS_00072775	MTCONS_00072773	chr16	21429554	21434384 chr16	21412820	21531830
cis_mRNA _overlap	Lnc-AntiOverlap-mRNA	LTCONS_00072833	MTCONS_00069227	chr16	22430090	22449153 chr16	22448001	22547841
cis_mRNA _overlap	Lnc-AntiOverlap-mRNA	LTCONS_00073228	MTCONS_00069784	chr16	31360768	31366378 chr16	31360768	31394318
cis_mRNA _overlap	mRNA-CompleteIn-LncExon	LTCONS_00073587	NM_022476	chr16	53524952	53537216 chr16	53524952	53537216
cis_mRNA dw20k	NA	LTCONS_00073700	NM_001301272	chr16	56700647	56701977 chr16	56691855	56693215
cis_mRNA dw20k	NA	LTCONS_00073700	NM_005949	chr16	56700647	56701977 chr16	56691855	56693215
cis_mRNA _overlap	mRNA-CompleteIn-LncExon	LTCONS_00073700	NM_001301267	chr16	56700647	56701977 chr16	56700647	56701977
cis_mRNA _overlap	mRNA-CompleteIn-LncExon	LTCONS_00073700	NM_005950	chr16	56700647	56701977 chr16	56700647	56701977
cis_mRNA up10k	NA	LTCONS_00073700	NM_005951	chr16	56700647	56701977 chr16	56703726	56705041
tran	NA	LTCONS_0007370	NM_005952	chr16	56700647	56701977 chr16	56716382	56718108
tran	NA	LTCONS_0007370	NM_005946	chr16	56700647	56701977 chr16	56672578	56674000
tran	NA	LTCONS_0007370	NM_175617	chr16	56700647	56701977 chr16	56659585	56661025

tran	NA	LTCONS_0007370	MTCONS_0007039	chr16	56700647	56701977	chr16	56651375	56653250
tran	NA	LTCONS_0007370	NM_005953	chr16	56700647	56701977	chr16	56642478	56643409
cis_mRNA up10k	NA	LTCONS_00073714	MTCONS_00070445	chr16	57018374	57023381	chr16	57023408	57117436
cis_mRNA overlap	Lnc-CompleteIn-	LTCONS_00073763	NM_001896	chr16	58193039	58197777	chr16	58191812	58231782
cis_mRNA overlap	Lnc-Overlap-mRNA	LTCONS_00074904	NM_001256183	chr16	89367274	89556969	chr16	89334029	89556969
cis_mRNA overlap	Lnc-Overlap-mRNA	LTCONS_00074907	NM_013275	chr16	89480058	89488989	chr16	89334029	89556969
cis_mRNA dw20k	NA	LTCONS_00075029	NM_014117	chr16	9215801	9217975	chr16	9185537	9213555
cis_mRNA dw20k	NA	LTCONS_00075030	NM_014117	chr16	9218688	9220997	chr16	9185537	9213555
cis_mRNA dw20k	NA	LTCONS_00075262	NM_018380	chr16	68038652	68039907	chr16	68055177	68057770
cis_mRNA _overlap	Lnc-AntiCompleteIn-mRNAIntron	LTCONS_00075361	MTCONS_00071493	chr16	85600831	85611224	chr16	85587006	85710419
cis_mRNA dw20k	NA	LTCONS_00075480	NM_022463	chr17	699305	702253	chr17	702553	882998
tran	NA	LTCONS_0007550	MTCONS_0009437	chr17	1366963	1377720	chr19	11487649	11495018
cis_mRNA _overlap	Lnc-AntiCompleteIn-mRNAIntron	LTCONS_00075518	NM_152346	chr17	1529755	1530998	chr17	1472548	1532180
cis_mRNA up10k	NA	LTCONS_00075971	MTCONS_00075973	chr17	7739163	7741542	chr17	7741764	7759952
cis_mRNA up10k	NA	LTCONS_00075971	MTCONS_00075976	chr17	7739163	7741542	chr17	7745623	7758118
cis_mRNA dw20k	NA	LTCONS_00076414	MTCONS_00076409	chr17	17712456	17714789	chr17	17584747	17707708
tran	NA	LTCONS_0007661	NM_001130111	chr17	20717933	20748002	chr19	1876975	1885518
cis_mRNA dw20k	NA	LTCONS_00076822	NM_014680	chr17	26926063	26933928	chr17	26941458	26972177

cis_mRNA_overlap	Lnc-AntiCompleteIn-mRNAIntron	LTCONS_00076911	NM_033389	chr17	28060757	28119763	chr17	27952957	28257058
cis_mRNA_overlap	Lnc-Overlap-mRNA	LTCONS_00076919	MTCONS_00076920	chr17	28443075	28466081	chr17	28443435	28515889
cis_mRNA_dw20k	NA	LTCONS_00077242	NM_002988	chr17	34400226	34418184	chr17	34391632	34398841
tran	NA	LTCONS_0007724	MTCONS_0008167	chr17	34415634	34418184	chr17	34522269	34625731
cis_mRNA_dw20k	NA	LTCONS_00077265	NM_001291472	chr17	34443216	34465758	chr17	34431220	34433014
cis_mRNA_overlap	Lnc-AntiOverlap-mRNA	LTCONS_00077276	MTCONS_00081678	chr17	34519990	34625580	chr17	34522269	34625731
tran	NA	LTCONS_0007728	NM_001291470	chr17	34639388	34641846	chr17	34431220	34433014
tran	NA	LTCONS_0007728	NM_001291474	chr17	34639388	34641846	chr17	34431220	34433014
tran	NA	LTCONS_0007728	NM_002984	chr17	34639388	34641846	chr17	34431220	34433014
tran	NA	LTCONS_0007728	NM_001291470	chr17	34639804	34641846	chr17	34431220	34433014
tran	NA	LTCONS_0007728	NM_002984	chr17	34639804	34641846	chr17	34431220	34433014
tran	NA	LTCONS_0007769	NM_015688	chr17	41283757	41284566	chr4	17633709	17783135
tran	NA	LTCONS_0007782	MTCONS_0004045	chr17	43116142	43117828	chr12	56915609	56989980
tran	NA	LTCONS_0007782	MTCONS_0009566	chr17	43116142	43117828	chr19	37128283	37157755
cis_mRNA_dw20k	NA	LTCONS_00077970	NM_004287	chr17	45023670	45031416	chr17	45000486	45018733
cis_mRNA_overlap	Lnc-CompleteIn-	LTCONS_00077970	MTCONS_00077969	chr17	45023670	45031416	chr17	45000486	45046518
cis_mRNA_up10k	NA	LTCONS_00078498	MTCONS_00082934	chr17	56410003	56431088	chr17	56378588	56406152
cis_mRNA_up10k	NA	LTCONS_00078498	MTCONS_00082938	chr17	56410003	56431088	chr17	56378588	56406152
cis_mRNA_overlap	Lnc-AntiCompleteIn-mRNAIntron	LTCONS_00078665	MTCONS_00083151	chr17	61791491	61805664	chr17	61780192	61820272
cis_mRNA_overlap	Lnc-Overlap-mRNA	LTCONS_00078697	NM_001291316	chr17	62461569	62471974	chr17	62461569	62464760

cis_mRNA overlap	Lnc-Overlap-mRNA	LTCONS_00078702	MTCONS_00078709	chr17	62502836	62512536	chr17	62502854	62534069
cis_mRNA up10k	NA	LTCONS_00078702	MTCONS_00083225	chr17	62502836	62512536	chr17	62494372	62502639
cis_mRNA overlap	Lnc-Overlap-mRNA	LTCONS_00078705	MTCONS_00078709	chr17	62502854	62512536	chr17	62502854	62534069
cis_mRNA overlap	Lnc-Overlap-mRNA	LTCONS_00078705	NM_138363	chr17	62502854	62512536	chr17	62502854	62534069
cis_mRNA up10k	NA	LTCONS_00078705	MTCONS_00083225	chr17	62502854	62512536	chr17	62494372	62502639
cis_mRNA dw20k	NA	LTCONS_00079325	MTCONS_00083846	chr17	74727079	74729963	chr17	74730197	74733493
cis_mRNA dw20k	NA	LTCONS_00079768	NM_001206950	chr17	80197531	80203467	chr17	80186282	80197375
cis_mRNA _overlap	Lnc-AntiOverlap-mRNA	LTCONS_00079768	MTCONS_00084231	chr17	80197531	80203467	chr17	80200537	80231594
cis_mRNA up10k	NA	LTCONS_00080375	NM_174893	chr17	6902367	6915668	chr17	6918056	6920843
cis_mRNA dw20k	NA	LTCONS_00080467	NM_004860	chr17	7484424	7489002	chr17	7494548	7518215
cis_mRNA dw20k	NA	LTCONS_00080467	NM_015670	chr17	7484424	7489002	chr17	7465309	7475287
cis_mRNA up10k	NA	LTCONS_00080535	MTCONS_00080525	chr17	8057961	8068086	chr17	8043788	8056490
cis_mRNA dw20k	NA	LTCONS_00080805	MTCONS_00080809	chr17	15414130	15433332	chr17	15440294	15466945
cis_mRNA dw20k	NA	LTCONS_00080805	NM_001135036	chr17	15414130	15433332	chr17	15440294	15466945
cis_mRNA dw20k	NA	LTCONS_00080851	MTCONS_00080855	chr17	15912939	15919834	chr17	15932738	16118874
cis_mRNA dw20k	NA	LTCONS_00080852	MTCONS_00080855	chr17	15920006	15930689	chr17	15932738	16118874
cis_mRNA dw20k	NA	LTCONS_00080873	MTCONS_00076279	chr17	16294860	16320003	chr17	16284604	16287220

tran	NA	LTCONS_0008103	NM_001288648	chr17	18561661	18585714	chr17	15602891	15624100
cis_mRNA overlap	Lnc-CompleteIn-	LTCONS_00081683	MTCONS_00081678	chr17	34596385	34598975	chr17	34522269	34625731
cis_mRNA up10k	NA	LTCONS_00081689	MTCONS_00081678	chr17	34626193	34628984	chr17	34522269	34625731
cis_mRNA up10k	NA	LTCONS_00081689	MTCONS_00081687	chr17	34626193	34628984	chr17	34623842	34625731
cis_mRNA overlap	Lnc-Overlap-mRNA	LTCONS_00082271	MTCONS_00082272	chr17	42109260	42118573	chr17	42109260	42144987
cis_mRNA dw20k	NA	LTCONS_00082501	MTCONS_00077969	chr17	45060155	45177663	chr17	45000486	45046518
cis_mRNA up10k	NA	LTCONS_00082945	MTCONS_00082936	chr17	56408245	56409869	chr17	56378588	56406152
cis_mRNA overlap	Lnc-CompleteIn-	LTCONS_00082974	NM_182620	chr17	57187308	57232800	chr17	57187308	57232800
cis_mRNA dw20k	NA	LTCONS_00083840	NM_003016	chr17	74714525	74718518	chr17	74730197	74733493
cis_mRNA overlap	Lnc-Overlap-mRNA	LTCONS_00083840	MTCONS_00083841	chr17	74714525	74718518	chr17	74714525	74722881
cis_mRNA overlap	Lnc-Overlap-mRNA	LTCONS_00083840	NM_001081461	chr17	74714525	74718518	chr17	74708914	74722881
cis_mRNA overlap	Lnc-Overlap-mRNA	LTCONS_00083840	NM_015167	chr17	74714525	74718518	chr17	74714525	74722881
cis_mRNA up10k	NA	LTCONS_00084334	MTCONS_00080397	chr17	7018513	7019646	chr17	7004641	7018355
cis_mRNA dw20k	NA	LTCONS_00084467	NM_015626	chr17	25649924	25652260	chr17	25621106	25640645
cis_mRNA dw20k	NA	LTCONS_00084468	NM_015626	chr17	25655748	25656483	chr17	25621106	25640645
cis_mRNA up10k	NA	LTCONS_00084494	NM_000286	chr17	33905715	33908233	chr17	33901814	33905656
cis_mRNA dw20k	NA	LTCONS_00084500	NM_001291470	chr17	34435284	34436237	chr17	34431220	34433014
cis_mRNA dw20k	NA	LTCONS_00084500	NM_001291474	chr17	34435284	34436237	chr17	34431220	34433014

cis_mRNA dw20k	NA	LTCONS_00084593	NM_005831	chr17	46946096	46948645	chr17	46908350	46942607
cis_mRNA up10k	NA	LTCONS_00084660	NM_005450	chr17	54670076	54670672	chr17	54671060	54672951
cis_mRNA up10k	NA	LTCONS_00084790	NM_052916	chr17	74236828	74237701	chr17	74138534	74236390
tran	NA	LTCONS_0008479	MTCONS_0014142	chr17	74519332	74520270	chr4	2965230	2972037
cis_mRNA up10k	NA	LTCONS_00084852	NM_004309	chr17	79830763	79831456	chr17	79825595	79829282
cis_mRNA overlap	Lnc-Overlap-mRNA	LTCONS_00085026	NM_170695	chr18	3449411	3468946	chr18	3451403	3458406
cis_mRNA overlap	Lnc-Overlap-mRNA	LTCONS_00085549	NM_001135993	chr18	21572737	21600262	chr18	21594384	21715574
cis_mRNA overlap	Lnc-Overlap-mRNA	LTCONS_00085549	NM_001243425	chr18	21572737	21600262	chr18	21594384	21600262
cis_mRNA overlap	Lnc-Overlap-mRNA	LTCONS_00085549	NM_153211	chr18	21572737	21600262	chr18	21572737	21715574
cis_mRNA overlap	Lnc-Overlap-mRNA	LTCONS_00086468	NM_020854	chr18	59969124	59972647	chr18	59854524	59974355
cis_mRNA overlap	Lnc-Overlap-mRNA	LTCONS_00086923	NM_172389	chr18	77283930	77289325	chr18	77160274	77289323
cis_mRNA _overlap	Lnc-AntiCompleteIn-mRNAIntron	LTCONS_00087526	NM_153211	chr18	21573362	21574941	chr18	21572737	21715574
cis_mRNA up10k	NA	LTCONS_00088086	NM_005904	chr18	46477146	46478412	chr18	46446223	46477081
cis_mRNA overlap	Lnc-Overlap-mRNA	LTCONS_00089357	MTCONS_00089360	chr19	1248552	1259142	chr19	1250821	1259142
cis_mRNA overlap	Lnc-Overlap-mRNA	LTCONS_00089372	NM_001300815	chr19	1273610	1274808	chr19	1269265	1274808
cis_mRNA dw20k	NA	LTCONS_00089436	MTCONS_00089430	chr19	1986973	1990369	chr19	1940871	1982748
cis_mRNA overlap	Lnc-Overlap-mRNA	LTCONS_00089548	MTCONS_00089543	chr19	3539332	3557567	chr19	3506295	3544097

cis_mRNA dw20k	NA	LTCONS_00090166	NM_001136501	chr19	12191070	12192573	chr19	12175514	12188631
cis_mRNA up10k	NA	LTCONS_00090202	NM_002229	chr19	12898010	12902111	chr19	12902310	12904125
cis_mRNA dw20k	NA	LTCONS_00090273	MTCONS_00090270	chr19	14191231	14199539	chr19	14183821	14185874
tran	NA	LTCONS_0009074	MTCONS_0009079	chr19	19835564	19839058	chr19	20946830	20993757
tran	NA	LTCONS_0009074	NM_003708	chr19	19835564	19839058	chr12	57345215	57353158
cis_mRNA overlap	Lnc-CompleteIn-	LTCONS_00090832	NM_145326	chr19	21583003	21587689	chr19	21579921	21591601
tran	NA	LTCONS_0009085	MTCONS_0009310	chr19	21769177	21905511	chr19	55417508	55445875
cis_mRNA overlap	Lnc-Overlap-mRNA	LTCONS_00091353	NM_001260489	chr19	35718342	35756026	chr19	35739559	35758867
cis_mRNA up10k	NA	LTCONS_00091404	NM_005306	chr19	35937061	35939688	chr19	35940617	35942669
cis_mRNA up10k	NA	LTCONS_00091778	NM_003407	chr19	39893338	39897272	chr19	39897487	39900052
cis_mRNA up10k	NA	LTCONS_00091778	MTCONS_00091781	chr19	39893338	39897272	chr19	39900474	39919055
cis_mRNA overlap	Lnc-Overlap-mRNA	LTCONS_00092697	MTCONS_00092701	chr19	51714343	51743274	chr19	51728335	51743274
cis_mRNA overlap	Lnc-Overlap-mRNA	LTCONS_00092697	NM_001772	chr19	51714343	51743274	chr19	51728335	51743274
tran	NA	LTCONS_0009285	NM_138374	chr19	53450320	53454191	chr19	53837002	53858122
tran	NA	LTCONS_0009285	MTCONS_0009694	chr19	53450320	53454191	chr19	53379425	53400919
tran	NA	LTCONS_0009285	NM_207333	chr19	53450320	53454191	chr19	53379425	53394599
cis_mRNA overlap	Lnc-Overlap-mRNA	LTCONS_00092924	NM_001317119	chr19	54057753	54060200	chr19	54058561	54083523
cis_mRNA _overlap	Lnc-AntiCompleteIn-mRNAIntron	LTCONS_00093004	MTCONS_00097085	chr19	54879277	54888761	chr19	54739078	55142756
cis_mRNA overlap	Lnc-Overlap-mRNA	LTCONS_00093034	NM_001278319	chr19	55105041	55111185	chr19	55105662	55111185

cis_mRNA_overlap	Lnc-AntiOverlap-mRNA	LTCONS_00093124	MTCONS_00097186	chr19	55550250	55555780	chr19	55555612	55575467
cis_mRNA_overlap	Lnc-Overlap-mRNA	LTCONS_00093736	NM_031918	chr19	1852337	1855315	chr19	1852398	1863564
cis_mRNA_overlap	Lnc-Overlap-mRNA	LTCONS_00094273	MTCONS_00094269	chr19	9868151	9896824	chr19	9861856	9896840
cis_mRNA_up10k	NA	LTCONS_00094488	NM_002229	chr19	12896014	12902151	chr19	12902310	12904125
cis_mRNA_dw20k	NA	LTCONS_00094545	NM_023072	chr19	13943077	13953702	chr19	13906274	13943044
cis_mRNA_up10k	NA	LTCONS_00094573	MTCONS_00090291	chr19	14416270	14481462	chr19	14490857	14519537
cis_mRNA_overlap	Lnc-AntiOverlap-mRNA	LTCONS_00094577	MTCONS_00090291	chr19	14492354	14494090	chr19	14490857	14519537
cis_mRNA_overlap	mRNA-CompleteIn-LncExon	LTCONS_00094594	NM_001300914	chr19	14625576	14635483	chr19	14625576	14628674
cis_mRNA_overlap	mRNA-CompleteIn-LncExon	LTCONS_00094594	NM_006145	chr19	14625576	14635483	chr19	14625576	14629371
cis_mRNA_dw20k	NA	LTCONS_00094731	MTCONS_00094733	chr19	16481918	16485730	chr19	16493366	16582823
cis_mRNA_up10k	NA	LTCONS_00095851	NM_003407	chr19	39893338	39897272	chr19	39897487	39900052
cis_mRNA_up10k	NA	LTCONS_00095851	MTCONS_00091781	chr19	39893338	39897272	chr19	39900474	39919055
cis_mRNA_up10k	NA	LTCONS_00095880	MTCONS_00095877	chr19	40450118	40459497	chr19	40402137	40440534
cis_mRNA_overlap	Lnc-AntiOverlap-mRNA	LTCONS_00095998	NM_002483	chr19	42260865	42263437	chr19	42259428	42276113
cis_mRNA_dw20k	NA	LTCONS_00096596	MTCONS_00096601	chr19	50356144	50363771	chr19	50363999	50370822

cis_mRNA overlap	Lnc-Overlap-mRNA	LTCONS_00096611	NM_001098632	chr19	50374368	50380644	chr19	50372290	50380644
cis_mRNA up10k	NA	LTCONS_00096611	NM_024682	chr19	50374368	50380644	chr19	50380682	50392007
cis_mRNA dw20k	NA	LTCONS_00097502	NM_014963	chr19	1101000	1103832	chr19	1107633	1174282
cis_mRNA up10k	NA	LTCONS_00097507	NM_003200	chr19	1652764	1653474	chr19	1609289	1652328
cis_mRNA up10k	NA	LTCONS_00097594	NM_001300925	chr19	16771219	16771852	chr19	16763167	16770968
cis_mRNA up10k	NA	LTCONS_00097715	MTCONS_00095578	chr19	36400569	36401003	chr19	36377315	36394757
cis_mRNA dw20k	NA	LTCONS_00097739	MTCONS_00091935	chr19	42104901	42105767	chr19	42082270	42093220
tran	NA	LTCONS_0009781	MTCONS_0009606	chr19	58203697	58204697	chr19	43084271	43099082
tran	NA	LTCONS_0009870	MTCONS_0009079	chr2	24757411	24792862	chr19	20946830	20993757
cis_mRNA dw20k	NA	LTCONS_00098851	NM_144631	chr2	27579896	27583123	chr2	27600098	27603611
cis_mRNA _overlap	Lnc-AntiOverlap-mRNA	LTCONS_00098851	NM_001035521	chr2	27579896	27583123	chr2	27548716	27579901
cis_mRNA _overlap	Lnc-AntiOverlap-mRNA	LTCONS_00099242	MTCONS_00107582	chr2	37826300	37870742	chr2	37869025	37899738
cis_mRNA _overlap	Lnc-AntiCompleteIn-mRNAIntron	LTCONS_00099249	MTCONS_00107582	chr2	37880962	37891266	chr2	37869025	37899738
cis_mRNA _overlap	Lnc-AntiCompleteIn-mRNAIntron	LTCONS_00099319	NM_198963	chr2	39037899	39040716	chr2	39024873	39103021
cis_mRNA dw20k	NA	LTCONS_00099513	NM_001033557	chr2	44464233	44468231	chr2	44395942	44458612
cis_mRNA up10k	NA	LTCONS_00099759	NM_014614	chr2	54197979	54278897	chr2	54091204	54197977

cis_mRNA overlap	Lnc-Overlap-mRNA	LTCONS_00099801	NM_001177413	chr2	55459541	55462989	chr2	55459814	55462989
cis_mRNA overlap	Lnc-CompleteIn-	LTCONS_00100444	NM_080916	chr2	74153953	74186088	chr2	74153953	74186088
cis_mRNA dw20k	NA	LTCONS_00100807	NM_172213	chr2	87035562	87037461	chr2	87042460	87089047
cis_mRNA overlap	Lnc-Overlap-mRNA	LTCONS_00100811	MTCONS_00100810	chr2	87174966	87191139	chr2	87173309	87239734
cis_mRNA up10k	NA	LTCONS_00100811	MTCONS_00100812	chr2	87174966	87191139	chr2	87191384	87239734
cis_mRNA up10k	NA	LTCONS_00100811	MTCONS_00100813	chr2	87174966	87191139	chr2	87197915	87239734
tran	NA	LTCONS_0010081	MTCONS_0011013	chr2	87174966	87191139	chr2	111320022	111334696
tran	NA	LTCONS_0010094	MTCONS_0010936	chr2	90077726	90078316	chr2	89156608	89442664
tran	NA	LTCONS_0010094	MTCONS_0010936	chr2	90077726	90078316	chr2	89156608	89442642
cis_mRNA overlap	Lnc-CompleteIn-	LTCONS_00101762	MTCONS_00101763	chr2	113032498	113077605	chr2	113032498	113077605
cis_mRNA up10k	NA	LTCONS_00101817	MTCONS_00110239	chr2	113594500	113596284	chr2	113587337	113594356
cis_mRNA up10k	NA	LTCONS_00101817	MTCONS_00110242	chr2	113594500	113596284	chr2	113587337	113594356
tran	NA	LTCONS_0010194	MTCONS_0000889	chr2	114341211	114356658	chr1	14407	29370
tran	NA	LTCONS_0010194	MTCONS_0006467	chr2	114341211	114356658	chr15	102501807	102516761
tran	NA	LTCONS_0010194	MTCONS_0006467	chr2	114349409	114356658	chr15	102501807	102516761
tran	NA	LTCONS_0010194	MTCONS_0009355	chr2	114349409	114356658	chr19	60024	70966
tran	NA	LTCONS_0010194	NM_182905	chr2	114349409	114356658	chr9	14511	29739
cis_mRNA overlap	Lnc-Overlap-mRNA	LTCONS_00102047	NM_016133	chr2	118857981	118868484	chr2	118846050	118867597
cis_mRNA _overlap	Lnc-AntiOverlap-mRNA	LTCONS_00103248	MTCONS_00111174	chr2	157189338	157191034	chr2	157180944	157190970
cis_mRNA overlap	Lnc-Overlap-mRNA	LTCONS_00103893	MTCONS_00103945	chr2	179387554	179493498	chr2	179405852	179408159

cis_mRNA_overlap	mRNA-CompleteIn-LncIntron	LTCONS_00103896	MTCONS_00103945	chr2	179387742	179480329	chr2	179405852	179408159
cis_mRNA_overlap	Lnc-Overlap-mRNA	LTCONS_00104298	MTCONS_00104296	chr2	192542798	192553248	chr2	192542798	192553248
cis_mRNA_overlap	Lnc-Overlap-mRNA	LTCONS_00104298	NM_001031716	chr2	192542798	192553248	chr2	192542861	192553248
cis_mRNA_overlap	mRNA-AntiCompleteIn-LncIntron	LTCONS_00104440	MTCONS_00112426	chr2	198340954	198367400	chr2	198351308	198364347
cis_mRNA_overlap	mRNA-AntiCompleteIn-LncIntron	LTCONS_00104440	MTCONS_00112427	chr2	198340954	198367400	chr2	198351308	198364640
cis_mRNA_overlap	mRNA-AntiCompleteIn-LncIntron	LTCONS_00104440	MTCONS_00112431	chr2	198340954	198367400	chr2	198357761	198364640
cis_mRNA_dw20k	NA	LTCONS_00104564	MTCONS_00104552	chr2	202041926	202047255	chr2	201983269	202037411
cis_mRNA_overlap	Lnc-Overlap-mRNA	LTCONS_00105114	MTCONS_00105111	chr2	219228155	219237940	chr2	219221579	219237940
cis_mRNA_overlap	Lnc-Overlap-mRNA	LTCONS_00105860	MTCONS_00105864	chr2	237994084	238007489	chr2	237994084	238016285
cis_mRNA_overlap	Lnc-Overlap-mRNA	LTCONS_00105861	MTCONS_00105864	chr2	237994084	238007489	chr2	237994084	238016285
cis_mRNA_dw20k	NA	LTCONS_00105898	NM_001137552	chr2	238674659	238690290	chr2	238600807	238674558
cis_mRNA_dw20k	NA	LTCONS_00106044	MTCONS_00106039	chr2	241981087	241986304	chr2	241938230	241974838
tran	NA	LTCONS_0010607	NM_001145204	chr2	242295664	242337183	chr16	12995477	13334273
cis_mRNA_overlap	mRNA-CompleteIn-LncExon	LTCONS_00106167	NM_001159597	chr2	218136	264547	chr2	218136	264097
cis_mRNA_dw20k	NA	LTCONS_00106572	NM_001256477	chr2	6967510	6980595	chr2	6990781	7005950

cis_mRNA dw20k	NA	LTCONS_00106572	NM_207315	chr2	6967510	6980595	chr2	6988440	7005950
cis_mRNA dw20k	NA	LTCONS_00106572	NM_001256478	chr2	6967510	6980595	chr2	6980684	7005950
cis_mRNA dw20k	NA	LTCONS_00106576	NM_001256477	chr2	6969265	6980595	chr2	6990781	7005950
cis_mRNA dw20k	NA	LTCONS_00106576	NM_207315	chr2	6969265	6980595	chr2	6988440	7005950
cis_mRNA overlap	Lnc-Overlap-mRNA	LTCONS_00106736	MTCONS_00106731	chr2	9079964	9099901	chr2	8996701	9127052
cis_mRNA _overlap	mRNA-CompleteIn-LncExon	LTCONS_00107697	NM_005633	chr2	39208690	39349017	chr2	39208690	39347604
cis_mRNA up10k	NA	LTCONS_00108030	MTCONS_00099674	chr2	48537377	48541736	chr2	48541795	48606434
cis_mRNA dw20k	NA	LTCONS_00108082	MTCONS_00108089	chr2	54091003	54095179	chr2	54102550	54197977
cis_mRNA up10k	NA	LTCONS_00108485	MTCONS_00100110	chr2	65273704	65283317	chr2	65283495	65314142
cis_mRNA overlap	Lnc-Overlap-mRNA	LTCONS_00109266	NM_001164732	chr2	86437768	86444492	chr2	86441120	86564777
cis_mRNA _overlap	mRNA-CompleteIn-LncExon	LTCONS_00109279	NM_001198951	chr2	86830135	86851000	chr2	86830516	86851000
cis_mRNA _overlap	mRNA-CompleteIn-LncExon	LTCONS_00109279	NM_005667	chr2	86830135	86851000	chr2	86830516	86851000
cis_mRNA overlap	Lnc-Overlap-mRNA	LTCONS_00109286	NM_001198952	chr2	86845909	86851639	chr2	86843540	86851000
cis_mRNA overlap	Lnc-Overlap-mRNA	LTCONS_00109286	NM_005667	chr2	86845909	86851639	chr2	86830516	86851000
cis_mRNA dw20k	NA	LTCONS_00109290	NM_004931	chr2	87042460	87055209	chr2	87068689	87089047
cis_mRNA overlap	Lnc-Overlap-mRNA	LTCONS_00109290	NM_172213	chr2	87042460	87055209	chr2	87042460	87089047

cis_mRNA_overlap	Lnc-AntiOverlap-mRNA	LTCONS_00109636	NM_017623	chr2	97492718	97507216	chr2	97481991	97501121
cis_mRNA_overlap	Lnc-Overlap-mRNA	LTCONS_00109636	NM_144994	chr2	97492718	97507216	chr2	97503651	97509758
cis_mRNA_dw20k	NA	LTCONS_00109729	NM_001134225	chr2	99215786	99224981	chr2	99061321	99207496
cis_mRNA_dw20k	NA	LTCONS_00109833	MTCONS_00109834	chr2	101887694	101891543	chr2	101892063	101925178
cis_mRNA_overlap	Lnc-Overlap-mRNA	LTCONS_00110736	MTCONS_00110737	chr2	132226784	132228175	chr2	132226784	132230090
cis_mRNA_overlap	Lnc-AntiOverlap-mRNA	LTCONS_00112199	NM_018471	chr2	187169913	187350886	chr2	187350885	187374087
tran	NA	LTCONS_0011237	MTCONS_0009079	chr2	196602427	196610967	chr19	20946830	20993757
cis_mRNA_dw20k	NA	LTCONS_00112530	NM_153689	chr2	200793070	200820459	chr2	200775979	200792996
cis_mRNA_dw20k	NA	LTCONS_00113178	NM_005687	chr2	223426717	223427185	chr2	223436162	223521074
cis_mRNA_up10k	NA	LTCONS_00114008	NM_004040	chr2	20636726	20640405	chr2	20646832	20649204
cis_mRNA_dw20k	NA	LTCONS_00114052	NM_001127401	chr2	30391829	30392548	chr2	30369750	30383399
cis_mRNA_dw20k	NA	LTCONS_00114079	NM_016252	chr2	32846858	32848838	chr2	32582096	32843965
cis_mRNA_dw20k	NA	LTCONS_00114128	NM_001033557	chr2	44468642	44470759	chr2	44395942	44458612
cis_mRNA_dw20k	NA	LTCONS_00114288	NM_152792	chr2	70172398	70174882	chr2	70187223	70189397
cis_mRNA_dw20k	NA	LTCONS_00114288	MTCONS_00100314	chr2	70172398	70174882	chr2	70142173	70170076
cis_mRNA_dw20k	NA	LTCONS_00114487	NM_145686	chr2	102516084	102519558	chr2	102314165	102511152
cis_mRNA_up10k	NA	LTCONS_00115093	NM_001127257	chr2	196513952	196516553	chr2	196521532	196602426

cis_mRNA dw20k	NA	LTCONS_00115276	MTCONS_00105432	chr2	228439262	228440366	chr2	228336888	228425938
cis_mRNA dw20k	NA	LTCONS_00115276	NM_004504	chr2	228439262	228440366	chr2	228336888	228425938
cis_mRNA _overlap	Lnc-AntiOverlap-mRNA	LTCONS_00115540	NM_001039508	chr20	1584670	1616564	chr20	1609798	1638425
cis_mRNA dw20k	NA	LTCONS_00116557	NM_080476	chr20	33146501	33148149	chr20	33148346	33265089
cis_mRNA overlap	Lnc-CompleteIn-	LTCONS_00116822	MTCONS_00116811	chr20	39732610	39734500	chr20	39657462	39753126
cis_mRNA overlap	Lnc-CompleteIn-	LTCONS_00116822	MTCONS_00116821	chr20	39732610	39734500	chr20	39723776	39753126
cis_mRNA dw20k	NA	LTCONS_00116987	NM_002638	chr20	43808589	43821571	chr20	43803540	43805185
cis_mRNA dw20k	NA	LTCONS_00117068	MTCONS_00119862	chr20	44560243	44576662	chr20	44577292	44600417
cis_mRNA dw20k	NA	LTCONS_00118005	NM_015894	chr20	62258393	62260943	chr20	62271058	62284963
cis_mRNA dw20k	NA	LTCONS_00118042	NM_181485	chr20	62371211	62378647	chr20	62339380	62367494
cis_mRNA overlap	Lnc-Overlap-mRNA	LTCONS_00118273	NM_022760	chr20	2815961	2821348	chr20	2815961	2821348
cis_mRNA overlap	Lnc-Overlap-mRNA	LTCONS_00119503	NM_004902	chr20	34328133	34330258	chr20	34291531	34330258
cis_mRNA dw20k	NA	LTCONS_00120006	NM_001316	chr20	47716433	47724463	chr20	47662783	47713497
cis_mRNA overlap	Lnc-Overlap-mRNA	LTCONS_00120139	MTCONS_00120142	chr20	50003494	50021219	chr20	50003494	50159258
cis_mRNA up10k	NA	LTCONS_00120432	MTCONS_00120424	chr20	58494959	58508457	chr20	58438612	58493593
cis_mRNA _overlap	mRNA-AntiCompleteIn-LncIntron	LTCONS_00121331	NM_001256579	chr21	15903689	16177028	chr21	15963713	16015428

cis_mRNA_overlap	Lnc-Overlap-mRNA	LTCONS_00123442	NM_001270402	chr21	35868395	35884611	chr21	35818986	35883637
cis_mRNA_dw20k	NA	LTCONS_00123675	MTCONS_00123679	chr21	40547372	40553360	chr21	40557404	40685712
cis_mRNA_up10k	NA	LTCONS_00124143	MTCONS_00122682	chr21	47738574	47743813	chr21	47744036	47865682
cis_mRNA_up10k	NA	LTCONS_00124284	NM_001270402	chr21	35885707	35886611	chr21	35818986	35883637
cis_mRNA_dw20k	NA	LTCONS_00124292	MTCONS_00122089	chr21	38892071	38897311	chr21	38735312	38887679
cis_mRNA_overlap	mRNA-AntiCompleteIn-LncExon	LTCONS_00125422	NM_001079539	chr22	29168662	29205275	chr22	29190548	29196560
cis_mRNA_dw20k	NA	LTCONS_00126247	NM_032311	chr22	42902245	42972131	chr22	42979727	43010968
cis_mRNA_up10k	NA	LTCONS_00126662	NM_004377	chr22	51022806	51038760	chr22	51007290	51016894
cis_mRNA_dw20k	NA	LTCONS_00126815	NM_001184781	chr22	19018596	19022477	chr22	19023795	19109967
cis_mRNA_dw20k	NA	LTCONS_00126815	NM_005137	chr22	19018596	19022477	chr22	19023795	19109967
cis_mRNA_overlap	Lnc-AntiOverlap-mRNA	LTCONS_00127117	MTCONS_00125011	chr22	23161672	23248974	chr22	23165261	23248991
cis_mRNA_overlap	mRNA-CompleteIn-LncExon	LTCONS_00127204	NM_213720	chr22	24107993	24110426	chr22	24108021	24110159
cis_mRNA_up10k	NA	LTCONS_00127204	NM_005940	chr22	24107993	24110426	chr22	24115006	24126503
cis_mRNA_overlap	Lnc-AntiOverlap-mRNA	LTCONS_00127238	MTCONS_00125117	chr22	24376133	24384311	chr22	24373095	24376211
cis_mRNA_overlap	mRNA-CompleteIn-LncExon	LTCONS_00127238	NM_000853	chr22	24376133	24384311	chr22	24376133	24384311

cis_mRNA_overlap	Lnc-Overlap-mRNA	LTCONS_00127457	MTCONS_00127458	chr22	29655844	29663972	chr22	29655844	29663986
cis_mRNA_overlap	mRNA-CompleteIn-LncExon	LTCONS_00128093	NM_052945	chr22	42314915	42323501	chr22	42321036	42322821
cis_mRNA_up10k	NA	LTCONS_00128093	MTCONS_00128089	chr22	42314915	42323501	chr22	42305212	42311493
cis_mRNA_overlap	Lnc-Overlap-mRNA	LTCONS_00128458	MTCONS_00128461	chr22	50683613	50689834	chr22	50683613	50689834
cis_mRNA_up10k	NA	LTCONS_00128458	MTCONS_00128455	chr22	50683613	50689834	chr22	50656118	50683400
cis_mRNA_overlap	mRNA-CompleteIn-LncExon	LTCONS_00128497	NM_001014440	chr22	50968838	50971008	chr22	50968838	50971008
cis_mRNA_up10k	NA	LTCONS_00128497	NM_001113756	chr22	50968838	50971008	chr22	50964182	50968514
cis_mRNA_overlap	mRNA-CompleteIn-LncExon	LTCONS_00128500	NM_001014440	chr22	50968838	50973982	chr22	50968838	50971008
cis_mRNA_up10k	NA	LTCONS_00128500	NM_001113756	chr22	50968838	50973982	chr22	50964182	50968514
cis_mRNA_up10k	NA	LTCONS_00128567	MTCONS_00124549	chr22	18892804	18893683	chr22	18893736	18899601
cis_mRNA_overlap	Lnc-CompleteIn-	LTCONS_00128884	MTCONS_00128880	chr3	5028728	5028949	chr3	5021097	5044868
cis_mRNA_overlap	Lnc-AntiCompleteIn-mRNAIntron	LTCONS_00129298	NM_015199	chr3	15784209	15784989	chr3	15708743	15901053
cis_mRNA_dw20k	NA	LTCONS_00129320	MTCONS_00129311	chr3	16364968	16368282	chr3	16306667	16364867
cis_mRNA_dw20k	NA	LTCONS_00129320	MTCONS_00129312	chr3	16364968	16368282	chr3	16306667	16364867
cis_mRNA_dw20k	NA	LTCONS_00129320	MTCONS_00129313	chr3	16364968	16368282	chr3	16306667	16364867

cis_mRNA dw20k	NA	LTCONS_00129320	NM_138381	chr3	16364968	16368282	chr3	16306667	16347594
cis_mRNA _overlap	mRNA-CompleteIn-LncExon	LTCONS_00130297	MTCONS_00130296	chr3	48282596	48312479	chr3	48282596	48312479
cis_mRNA overlap	Lnc-Overlap-mRNA	LTCONS_00130819	MTCONS_00130825	chr3	63850012	63888823	chr3	63850233	63993755
cis_mRNA overlap	Lnc-Overlap-mRNA	LTCONS_00131461	NM_001271142	chr3	98482174	98501592	chr3	98451080	98514689
cis_mRNA overlap	Lnc-Overlap-mRNA	LTCONS_00131462	MTCONS_00131460	chr3	98482174	98514792	chr3	98482158	98514792
cis_mRNA dw20k	NA	LTCONS_00131534	NM_017819	chr3	101291844	101313310	chr3	101280680	101285290
cis_mRNA overlap	Lnc-Overlap-mRNA	LTCONS_00131534	NM_020357	chr3	101291844	101313310	chr3	101292938	101313310
cis_mRNA _overlap	Lnc-AntiOverlap-mRNA	LTCONS_00131540	MTCONS_00137139	chr3	101405489	101421908	chr3	101399934	101410311
cis_mRNA dw20k	NA	LTCONS_00131566	MTCONS_00131557	chr3	101591270	101623453	chr3	101568358	101579869
cis_mRNA dw20k	NA	LTCONS_00131566	MTCONS_00131558	chr3	101591270	101623453	chr3	101568358	101579869
cis_mRNA dw20k	NA	LTCONS_00131566	MTCONS_00131559	chr3	101591270	101623453	chr3	101568358	101579869
cis_mRNA _overlap	mRNA-CompleteIn-LncExon	LTCONS_00132124	NM_032839	chr3	122512733	122599986	chr3	122513901	122599986
cis_mRNA up10k	NA	LTCONS_00132124	NM_024610	chr3	122512733	122599986	chr3	122458844	122512666
cis_mRNA dw20k	NA	LTCONS_00133673	NM_006218	chr3	178955889	178957540	chr3	178866311	178952497
cis_mRNA overlap	Lnc-CompleteIn-	LTCONS_00134333	NM_130834	chr3	193337485	193342262	chr3	193310933	193415600

cis_mRNA_overlap	Lnc-AntiOverlap-mRNA	LTCONS_00134857	MTCONS_00128880	chr3	5026876	5067640	chr3	5021097	5044868
cis_mRNA_overlap	Lnc-AntiOverlap-mRNA	LTCONS_00134857	MTCONS_00128881	chr3	5026876	5067640	chr3	5021097	5044868
cis_mRNA_up10k	NA	LTCONS_00135616	NM_001008392	chr3	37844866	37903271	chr3	37903669	38025960
tran	NA	LTCONS_0013672	MTCONS_0009427	chr3	69031048	69043430	chr19	9920943	9929779
cis_mRNA_overlap	Lnc-Overlap-mRNA	LTCONS_00137537	MTCONS_00137523	chr3	114720618	114866127	chr3	114033347	114819227
cis_mRNA_overlap	Lnc-Overlap-mRNA	LTCONS_00137537	MTCONS_00137524	chr3	114720618	114866127	chr3	114033347	114866127
cis_mRNA_overlap	Lnc-Overlap-mRNA	LTCONS_00137596	NM_018266	chr3	119166694	119182529	chr3	119147807	119182529
cis_mRNA_dw20k	NA	LTCONS_00137605	MTCONS_00132023	chr3	119355402	119357848	chr3	119316695	119348658
cis_mRNA_overlap	Lnc-Overlap-mRNA	LTCONS_00137999	NM_001276270	chr3	129149787	129159022	chr3	129149787	129159022
cis_mRNA_overlap	Lnc-Overlap-mRNA	LTCONS_00138906	NM_178824	chr3	167366148	167375559	chr3	167196473	167371289
cis_mRNA_overlap	Lnc-Overlap-mRNA	LTCONS_00138953	MTCONS_00138955	chr3	169805368	169830154	chr3	169805368	169899537
cis_mRNA_overlap	mRNA-CompleteIn-LncExon	LTCONS_00138968	MTCONS_00138969	chr3	170582665	170588045	chr3	170582665	170588045
cis_mRNA_overlap	mRNA-CompleteIn-LncExon	LTCONS_00138968	NM_001099645	chr3	170582665	170588045	chr3	170582665	170588045
cis_mRNA_overlap	Lnc-Overlap-mRNA	LTCONS_00139147	NM_152240	chr3	178735011	178791010	chr3	178735013	178789656
cis_mRNA_dw20k	NA	LTCONS_00139192	MTCONS_00133745	chr3	180701498	180707562	chr3	180630234	180686700
cis_mRNA_up10k	NA	LTCONS_00139928	NM_001312673	chr3	196017981	196045202	chr3	195964616	196014623

cis_mRNA up10k	NA	LTCONS_00140289	NM_014240	chr3	45633710	45635566	chr3	45636323	45722755
cis_mRNA dw20k	NA	LTCONS_00140323	MTCONS_00130471	chr3	50625522	50631752	chr3	50606325	50622421
cis_mRNA dw20k	NA	LTCONS_00140507	NM_000097	chr3	98281536	98284751	chr3	98298290	98312455
cis_mRNA dw20k	NA	LTCONS_00140514	MTCONS_00131558	chr3	101586033	101586693	chr3	101568358	101579869
cis_mRNA dw20k	NA	LTCONS_00140830	NM_007287	chr3	154905118	154906692	chr3	154797705	154901518
cis_mRNA up10k	NA	LTCONS_00140962	NM_001190942	chr3	172242496	172243202	chr3	172223298	172241297
cis_mRNA up10k	NA	LTCONS_00141096	MTCONS_00134375	chr3	193852143	193853639	chr3	193853931	193856401
cis_mRNA up10k	NA	LTCONS_00141096	NM_005524	chr3	193852143	193853639	chr3	193853931	193856401
cis_mRNA overlap	Lnc-Overlap-mRNA	LTCONS_00141188	MTCONS_00141183	chr4	523435	533710	chr4	492989	533710
cis_mRNA up10k	NA	LTCONS_00141338	NM_012318	chr4	1864443	1870412	chr4	1813206	1857974
cis_mRNA up10k	NA	LTCONS_00141408	NM_001120	chr4	2936626	2943919	chr4	2932288	2935964
tran	NA	LTCONS_0014152	NM_020702	chr4	4551805	4554923	chr9	34366664	34376894
cis_mRNA overlap	Lnc-Overlap-mRNA	LTCONS_00141579	MTCONS_00141580	chr4	6675478	6677814	chr4	6675678	6677814
cis_mRNA overlap	Lnc-Overlap-mRNA	LTCONS_00141579	MTCONS_00141581	chr4	6675478	6677814	chr4	6675684	6677814
cis_mRNA _overlap	mRNA-CompleteIn-LncExon	LTCONS_00142074	NM_015874	chr4	26319393	26436752	chr4	26322429	26436752
cis_mRNA up10k	NA	LTCONS_00142335	NM_003359	chr4	39529351	39552545	chr4	39500376	39529218
cis_mRNA overlap	Lnc-Overlap-mRNA	LTCONS_00142479	MTCONS_00142486	chr4	44680433	44695964	chr4	44680433	44702697

cis_mRNA overlap	Lnc-Overlap-mRNA	LTCONS_00143365	MTCONS_00143348	chr4	88050934	88062206	chr4	87856154	88062206
cis_mRNA up10k	NA	LTCONS_00143754	NM_016269	chr4	109092927	109177992	chr4	108968701	109090112
cis_mRNA up10k	NA	LTCONS_00143845	NM_052864	chr4	113207162	113209409	chr4	113196782	113207059
cis_mRNA _overlap	Lnc-AntiCompleteIn-mRNAIntron	LTCONS_00144544	NM_001101669	chr4	143484095	143543002	chr4	142949182	143767604
cis_mRNA _overlap	Lnc-AntiCompleteIn-mRNAIntron	LTCONS_00144545	NM_001101669	chr4	143486778	143509625	chr4	142949182	143767604
cis_mRNA _overlap	Lnc-AntiCompleteIn-mRNAIntron	LTCONS_00144547	NM_001101669	chr4	143486778	143543002	chr4	142949182	143767604
cis_mRNA up10k	NA	LTCONS_00144548	MTCONS_00148629	chr4	143486778	143584931	chr4	142942517	143481957
cis_mRNA dw20k	NA	LTCONS_00144810	MTCONS_00144801	chr4	154633731	154654236	chr4	154605073	154627412
cis_mRNA up10k	NA	LTCONS_00145004	MTCONS_00145006	chr4	159119167	159124030	chr4	159130912	159176439
cis_mRNA up10k	NA	LTCONS_00145820	MTCONS_00141183	chr4	477198	492960	chr4	492989	533710
cis_mRNA up10k	NA	LTCONS_00145821	MTCONS_00141183	chr4	485872	492960	chr4	492989	533710
cis_mRNA _overlap	Lnc-AntiOverlap-mRNA	LTCONS_00146191	MTCONS_00141612	chr4	7646762	7655864	chr4	7651065	7744564
tran	NA	LTCONS_0014729	MTCONS_0009079	chr4	75114439	75196399	chr19	20946830	20993757
cis_mRNA dw20k	NA	LTCONS_00148540	NM_006874	chr4	139967637	139972462	chr4	139978871	140005568
cis_mRNA overlap	Lnc-CompleteIn-	LTCONS_00148634	NM_001101669	chr4	143494000	143496249	chr4	142949182	143767604
cis_mRNA overlap	other	LTCONS_00148939	NM_152680	chr4	153545402	153547266	chr4	153547266	153601317

cis_mRNA overlap	Lnc-Overlap-mRNA	LTCONS_00149081	MTCONS_00149076	chr4	164064474	164088347	chr4	164049820	164088347
cis_mRNA dw20k	NA	LTCONS_00150030	NM_018126	chr4	41966165	41968961	chr4	41937137	41962824
cis_mRNA dw20k	NA	LTCONS_00150082	NM_032313	chr4	57809284	57810449	chr4	57829510	57843826
cis_mRNA dw20k	NA	LTCONS_00152375	NM_001243953	chr5	61684892	61686705	chr5	61601989	61683011
cis_mRNA dw20k	NA	LTCONS_00152375	NM_004520	chr5	61684892	61686705	chr5	61601989	61683011
cis_mRNA overlap	Lnc-CompleteIn-	LTCONS_00152450	MTCONS_00152446	chr5	65449871	65479444	chr5	65440046	65479444
cis_mRNA overlap	Lnc-Overlap-mRNA	LTCONS_00152450	NM_001270492	chr5	65449871	65479444	chr5	65440046	65479444
cis_mRNA overlap	Lnc-Overlap-mRNA	LTCONS_00152450	NM_001270493	chr5	65449871	65479444	chr5	65440046	65453569
cis_mRNA overlap	Lnc-Overlap-mRNA	LTCONS_00152451	NM_139168	chr5	65454288	65463201	chr5	65440663	65479444
cis_mRNA _overlap	Lnc-AntiCompleteIn-mRNAExon	LTCONS_00152627	MTCONS_00157857	chr5	69404135	69406240	chr5	69387093	69586514
cis_mRNA overlap	Lnc-Overlap-mRNA	LTCONS_00153146	MTCONS_00153141	chr5	86674976	86690080	chr5	86563828	86690080
cis_mRNA _overlap	Lnc-AntiOverlap-mRNA	LTCONS_00153164	NM_153354	chr5	87564667	87732860	chr5	87491023	87564696
cis_mRNA dw20k	NA	LTCONS_00153564	MTCONS_00153561	chr5	102541667	102549081	chr5	102455958	102541531
cis_mRNA overlap	Lnc-Overlap-mRNA	LTCONS_00153736	MTCONS_00153739	chr5	112312407	112320362	chr5	112312407	112357892
cis_mRNA overlap	Lnc-Overlap-mRNA	LTCONS_00154142	NM_020240	chr5	130588733	130730382	chr5	130599702	130730382
cis_mRNA overlap	Lnc-CompleteIn-	LTCONS_00154174	MTCONS_00154171	chr5	131631112	131635226	chr5	131630088	131680244

cis_mRNA dw20k	NA	LTCONS_00154185	NM_003060	chr5	131746569	131805830	chr5	131705401	131731306
cis_mRNA dw20k	NA	LTCONS_00154197	NM_002188	chr5	132009678	132018370	chr5	131993865	131996801
cis_mRNA _overlap	mRNA-CompleteIn-LncExon	LTCONS_00154249	NM_003202	chr5	133425573	133492072	chr5	133450402	133483920
cis_mRNA overlap	Lnc-Overlap-mRNA	LTCONS_00154264	NM_001134851	chr5	133445487	133492072	chr5	133451298	133483920
cis_mRNA _overlap	mRNA-CompleteIn-LncExon	LTCONS_00154268	NM_001134851	chr5	133445487	133492072	chr5	133451298	133483920
cis_mRNA _overlap	Lnc-AntiOverlap-mRNA	LTCONS_00154757	MTCONS_00159674	chr5	141016517	141020631	chr5	141018594	141031041
cis_mRNA overlap	Lnc-Overlap-mRNA	LTCONS_00154948	MTCONS_00154952	chr5	147763498	147778152	chr5	147763498	147822399
cis_mRNA overlap	Lnc-Overlap-mRNA	LTCONS_00154948	MTCONS_00154953	chr5	147763498	147778152	chr5	147763498	147822399
cis_mRNA overlap	Lnc-Overlap-mRNA	LTCONS_00154948	MTCONS_00154954	chr5	147763498	147778152	chr5	147763498	147822399
cis_mRNA up10k	NA	LTCONS_00155623	MTCONS_00160420	chr5	172198313	172205905	chr5	172195093	172198203
cis_mRNA up10k	NA	LTCONS_00155623	NM_004417	chr5	172198313	172205905	chr5	172195093	172198203
cis_mRNA up10k	NA	LTCONS_00155624	MTCONS_00160420	chr5	172198313	172209631	chr5	172195093	172198203
cis_mRNA up10k	NA	LTCONS_00155624	NM_004417	chr5	172198313	172209631	chr5	172195093	172198203
cis_mRNA up10k	NA	LTCONS_00155828	NM_014901	chr5	175965536	175974398	chr5	175953700	175964421
cis_mRNA up10k	NA	LTCONS_00155878	NM_001004106	chr5	176852117	176853603	chr5	176853687	176869850

cis_mRNA_overlap	Lnc-AntiOverlap-mRNA	LTCONS_00156035	NM_014275	chr5	179229895	179243800	chr5	179224598	179233952
cis_mRNA_overlap	Lnc-AntiOverlap-mRNA	LTCONS_00157030	MTCONS_00151803	chr5	34900635	34929848	chr5	34915820	34959069
cis_mRNA_dw20k	NA	LTCONS_00158827	NM_001744	chr5	110831733	110848295	chr5	110559947	110820748
cis_mRNA_overlap	Lnc-Overlap-mRNA	LTCONS_00158827	NM_139164	chr5	110831733	110848295	chr5	110831733	110848295
cis_mRNA_overlap	mRNA-CompleteIn-LncExon	LTCONS_00158833	NM_139164	chr5	110831733	110850566	chr5	110831733	110848295
cis_mRNA_overlap	Lnc-Overlap-mRNA	LTCONS_00159122	NM_001164479	chr5	126371326	126386436	chr5	126378250	126409184
cis_mRNA_overlap	Lnc-Overlap-mRNA	LTCONS_00159323	NM_001300818	chr5	132354722	132362296	chr5	132332678	132362296
cis_mRNA_up10k	NA	LTCONS_00159344	NM_003202	chr5	133425573	133442073	chr5	133450402	133483920
cis_mRNA_overlap	Lnc-Overlap-mRNA	LTCONS_00159575	MTCONS_00159574	chr5	139937853	139944189	chr5	139929652	139944255
cis_mRNA_overlap	Lnc-Overlap-mRNA	LTCONS_00160340	MTCONS_00160342	chr5	169675088	169680425	chr5	169675088	169724822
cis_mRNA_overlap	Lnc-Overlap-mRNA	LTCONS_00160576	NM_014901	chr5	175953356	175964421	chr5	175953700	175964421
cis_mRNA_dw20k	NA	LTCONS_00160678	MTCONS_00160685	chr5	177138118	177145570	chr5	177150147	177194185
cis_mRNA_up10k	NA	LTCONS_00160905	NM_001267556	chr5	264477	264888	chr5	271736	315089
tran	NA	LTCONS_0016090	MTCONS_0013990	chr5	264477	264888	chr3	195684922	195717189
cis_mRNA_up10k	NA	LTCONS_00161244	NM_014324	chr5	34008326	34009189	chr5	33987091	34008220
cis_mRNA_dw20k	NA	LTCONS_00161455	NM_152687	chr5	57794845	57796357	chr5	57787262	57792185

cis_mRNA dw20k	NA	LTCONS_00161456	NM_152687	chr5	57802126	57805001	chr5	57787262	57792185
cis_mRNA dw20k	NA	LTCONS_00161999	NM_000024	chr5	148213672	148221341	chr5	148206156	148208197
cis_mRNA _overlap	Lnc-AntiCompleteIn-mRNAIntron	LTCONS_00161999	MTCONS_00154964	chr5	148213672	148221341	chr5	148206156	148244494
cis_mRNA dw20k	NA	LTCONS_00162000	NM_000024	chr5	148221648	148222547	chr5	148206156	148208197
cis_mRNA _overlap	Lnc-AntiCompleteIn-mRNAIntron	LTCONS_00162000	MTCONS_00154964	chr5	148221648	148222547	chr5	148206156	148244494
cis_mRNA dw20k	NA	LTCONS_00162072	NM_001130864	chr5	159497119	159499761	chr5	159518347	159546452
cis_mRNA up10k	NA	LTCONS_00162147	MTCONS_00160606	chr5	176740355	176742835	chr5	176728191	176730745
cis_mRNA up10k	NA	LTCONS_00162169	MTCONS_00160829	chr5	180244208	180245176	chr5	180217541	180242621
cis_mRNA up10k	NA	LTCONS_00162170	MTCONS_00160829	chr5	180247119	180249406	chr5	180217541	180242621
cis_mRNA overlap	Lnc-Overlap-mRNA	LTCONS_00163077	NM_001100829	chr6	11537082	11583757	chr6	11538460	11583757
cis_mRNA overlap	Lnc-Overlap-mRNA	LTCONS_00163103	MTCONS_00163098	chr6	12012152	12098550	chr6	12002325	12166086
cis_mRNA overlap	Lnc-CompleteIn-	LTCONS_00163179	NM_004233	chr6	14117865	14137148	chr6	14117865	14137148
cis_mRNA overlap	Lnc-Overlap-mRNA	LTCONS_00163179	NM_001040280	chr6	14117865	14137148	chr6	14117865	14137148
cis_mRNA dw20k	NA	LTCONS_00163282	MTCONS_00169213	chr6	17607458	17611950	chr6	17615266	17707101
cis_mRNA dw20k	NA	LTCONS_00164526	NM_001315	chr6	36093406	36096345	chr6	35995454	36079013
cis_mRNA up10k	NA	LTCONS_00164734	NM_001271807	chr6	41122194	41193203	chr6	41116999	41122087

cis_mRNA overlap	Lnc-Overlap-mRNA	LTCONS_00164921	NM_001025368	chr6	43739722	43754223	chr6	43737946	43754223
cis_mRNA overlap	Lnc-Overlap-mRNA	LTCONS_00164921	NM_001171626	chr6	43739722	43754223	chr6	43737946	43754223
cis_mRNA overlap	Lnc-Overlap-mRNA	LTCONS_00164921	NM_001317010	chr6	43739722	43754223	chr6	43737946	43754223
cis_mRNA overlap	Lnc-CompleteIn-	LTCONS_00165407	NM_001160130	chr6	73347039	73367769	chr6	73331571	73908573
cis_mRNA dw20k	NA	LTCONS_00165429	NM_012123	chr6	74216688	74220359	chr6	74171454	74211179
cis_mRNA dw20k	NA	LTCONS_00165429	NM_133645	chr6	74216688	74220359	chr6	74171454	74211179
cis_mRNA dw20k	NA	LTCONS_00166810	NM_001139510	chr6	127587827	127609705	chr6	127609857	127663552
cis_mRNA overlap	Lnc-Overlap-mRNA	LTCONS_00167149	MTCONS_00167150	chr6	138191562	138201729	chr6	138191562	138204451
cis_mRNA overlap	Lnc-Overlap-mRNA	LTCONS_00167149	MTCONS_00167153	chr6	138191562	138201729	chr6	138191565	138204451
cis_mRNA overlap	Lnc-Overlap-mRNA	LTCONS_00167149	NM_001270507	chr6	138191562	138201729	chr6	138188325	138204451
cis_mRNA overlap	Lnc-Overlap-mRNA	LTCONS_00167149	NM_006290	chr6	138191562	138201729	chr6	138188325	138204451
cis_mRNA overlap	Lnc-Overlap-mRNA	LTCONS_00167152	MTCONS_00167150	chr6	138191565	138201729	chr6	138191562	138204451
cis_mRNA overlap	Lnc-Overlap-mRNA	LTCONS_00167152	NM_001270507	chr6	138191565	138201729	chr6	138188325	138204451
cis_mRNA overlap	Lnc-Overlap-mRNA	LTCONS_00167152	NM_001270508	chr6	138191565	138201729	chr6	138188325	138204451
cis_mRNA overlap	Lnc-Overlap-mRNA	LTCONS_00167152	NM_006290	chr6	138191565	138201729	chr6	138188325	138204451
cis_mRNA _overlap	Lnc-AntiOverlap-mRNA	LTCONS_00167289	MTCONS_00172815	chr6	143267665	143277784	chr6	143072604	143270664
cis_mRNA overlap	Lnc-Overlap-mRNA	LTCONS_00167345	MTCONS_00167346	chr6	144471654	144475038	chr6	144471654	144513076

cis_mRNA_overlap	Lnc-Overlap-mRNA	LTCONS_00167764	NM_001242395	chr6	159069717	159085278	chr6	159071046	159185908
cis_mRNA_up10k	NA	LTCONS_00167776	NM_001302839	chr6	159288104	159290743	chr6	159290899	159331385
cis_mRNA_overlap	Lnc-AntiCompleteIn-mRNAIntron	LTCONS_00167801	NM_001278733	chr6	159460342	159461754	chr6	159455501	159463385
cis_mRNA_overlap	Lnc-AntiOverlap-mRNA	LTCONS_00168177	MTCONS_00173633	chr6	170053074	170110335	chr6	170106484	170124106
cis_mRNA_overlap	Lnc-Overlap-mRNA	LTCONS_00169008	MTCONS_00169004	chr6	11343561	11382581	chr6	11183531	11382581
cis_mRNA_overlap	Lnc-AntiOverlap-mRNA	LTCONS_00169031	MTCONS_00163106	chr6	12042453	12093616	chr6	12012900	12166086
tran	NA	LTCONS_0016905	MTCONS_0010113	chr6	13276248	13277101	chr2	97778343	97930725
tran	NA	LTCONS_0016905	MTCONS_0011940	chr6	13276248	13277101	chr20	33302578	33413452
tran	NA	LTCONS_0016905	NM_000440	chr6	13276248	13277101	chr5	149237519	149324356
tran	NA	LTCONS_0016905	NM_001205319	chr6	13276248	13277101	chr17	702553	767351
tran	NA	LTCONS_0016905	NM_001652	chr6	13276248	13277101	chr12	50366620	50370922
tran	NA	LTCONS_0016905	NM_005026	chr6	13276248	13277101	chr1	9711790	9789172
tran	NA	LTCONS_0016905	NM_014293	chr6	13276248	13277101	chr22	39214456	39240017
tran	NA	LTCONS_0016905	NM_022463	chr6	13276248	13277101	chr17	702553	882998
tran	NA	LTCONS_0016905	NM_173641	chr6	13276248	13277101	chr1	38225945	38230824
cis_mRNA_overlap	Lnc-Overlap-mRNA	LTCONS_00169863	NM_002117	chr6	31236526	31237627	chr6	31236526	31239913
cis_mRNA_up10k	NA	LTCONS_00170268	NM_005643	chr6	34855949	34856968	chr6	34845555	34855848
cis_mRNA_overlap	mRNA-CompleteIn-LncExon	LTCONS_00170506	NM_145063	chr6	41032627	41040281	chr6	41034531	41040188
cis_mRNA_dw20k	NA	LTCONS_00170529	NM_001242590	chr6	41235277	41242641	chr6	41242999	41254457
cis_mRNA_dw20k	NA	LTCONS_00170529	NM_018643	chr6	41235277	41242641	chr6	41242999	41254457

cis_mRNA_overlap	Lnc-Overlap-mRNA	LTCONS_00170606	MTCONS_00170598	chr6	42325058	42419907	chr6	42192511	42419701
cis_mRNA_overlap	Lnc-Overlap-mRNA	LTCONS_00172320	MTCONS_00172318	chr6	128232711	128239776	chr6	128029339	128239776
cis_mRNA_overlap	Lnc-Overlap-mRNA	LTCONS_00172478	NM_005627	chr6	134494922	134496034	chr6	134490384	134496034
cis_mRNA_overlap	Lnc-Overlap-mRNA	LTCONS_00172542	MTCONS_00172540	chr6	135814876	135818905	chr6	135714266	135818903
cis_mRNA_overlap	Lnc-AntiOverlap-mRNA	LTCONS_00172680	MTCONS_00167150	chr6	138175261	138199568	chr6	138191562	138204451
cis_mRNA_overlap	Lnc-AntiOverlap-mRNA	LTCONS_00172680	NM_001270507	chr6	138175261	138199568	chr6	138188325	138204451
cis_mRNA_overlap	Lnc-AntiOverlap-mRNA	LTCONS_00172680	NM_001270508	chr6	138175261	138199568	chr6	138188325	138204451
cis_mRNA_overlap	Lnc-AntiOverlap-mRNA	LTCONS_00172680	NM_006290	chr6	138175261	138199568	chr6	138188325	138204451
cis_mRNA_overlap	Lnc-Overlap-mRNA	LTCONS_00173469	NM_016098	chr6	166778408	166796504	chr6	166778408	166796501
cis_mRNA_overlap	Lnc-Overlap-mRNA	LTCONS_00173629	NM_182552	chr6	170071388	170102159	chr6	169857303	170102159
cis_mRNA_dw20k	NA	LTCONS_00173800	NM_001040280	chr6	14139458	14140469	chr6	14117865	14137148
cis_mRNA_dw20k	NA	LTCONS_00173801	NM_001040280	chr6	14140657	14144455	chr6	14117865	14137148
cis_mRNA_dw20k	NA	LTCONS_00173801	NM_004233	chr6	14140657	14144455	chr6	14117865	14137148
cis_mRNA_dw20k	NA	LTCONS_00173862	NM_018473	chr6	24721530	24723135	chr6	24667263	24705295
cis_mRNA_dw20k	NA	LTCONS_00173870	NM_003524	chr6	26254574	26256922	chr6	26251879	26252303

cis_mRNA up10k	NA	LTCONS_00173923	NM_002123	chr6	32635173	32636181	chr6	32627241	32634466
cis_mRNA dw20k	NA	LTCONS_00173929	NM_005104	chr6	32952020	32954625	chr6	32936437	32949282
tran	NA	LTCONS_0017432	MTCONS_0001507	chr6	95227988	95228771	chr1	183595328	183605076
tran	NA	LTCONS_0017432	NM_005717	chr6	95227988	95228771	chr1	183595328	183605076
tran	NA	LTCONS_0017454	MTCONS_0009427	chr6	123996072	124000107	chr19	9920943	9929779
tran	NA	LTCONS_0017454	NM_001242575	chr6	123996072	124000107	chr2	214141277	214148929
cis_mRNA up10k	NA	LTCONS_00174748	NM_016020	chr6	155635763	155638210	chr6	155577264	155635617
cis_mRNA overlap	Lnc-CompleteIn-	LTCONS_00174904	MTCONS_00174903	chr6_apd_hap	3101122	3101734	chr6_apd_hap	3098016	3113026
cis_mRNA up10k	NA	LTCONS_00174904	MTCONS_00174905	chr6_apd_hap	3101122	3101734	chr6_apd_hap	3110206	3122953
tran	NA	LTCONS_00174904	MTCONS_00175297	chr6_apd_hap	3101122	3101734	chr6_qbl_hap	3076875	3080274
cis_mRNA_overlap	Lnc-AntiOverlap-mRNA	LTCONS_00174915	MTCONS_00174903	chr6_apd_hap1	3099538	3107063	chr6_apd_hap1	3098016	3113026
cis_mRNA up10k	NA	LTCONS_00174915	MTCONS_00174905	chr6_apd_hap	3099538	3107063	chr6_apd_hap	3110206	3122953
tran	NA	LTCONS_00174915	MTCONS_00175049	chr6_apd_hap	3099538	3107063	chr6_dbb_hap	3068866	3071307
tran	NA	LTCONS_00174915	MTCONS_00175299	chr6_apd_hap	3099538	3107063	chr6_qbl_hap	3078132	3091932
cis_mRNA overlap	Lnc-CompleteIn-	LTCONS_00174961	MTCONS_00174959	chr6_cox_hap	4489269	4491883	chr6_cox_hap	4487902	4502069
tran	NA	LTCONS_00174981	MTCONS_00175051	chr6_cox_hap	3292111	3306149	chr6_dbb_hap	3079475	3093125
tran	NA	LTCONS_00174982	MTCONS_00175049	chr6_cox_hap	3292111	3307845	chr6_dbb_hap	3068866	3071307
tran	NA	LTCONS_00175018	MTCONS_00163846	chr6_dbb_hap	1149538	1207092	chr6	29855917	29858839
tran	NA	LTCONS_00175018	MTCONS_00174902	chr6_dbb_hap	1149538	1207092	chr6_apd_hap	1150151	1153525

tran	NA	LTCONS_00175018	NM_001242758	chr6_dbb_hap	1149538	1207092	chr6	29910247	29913661
tran	NA	LTCONS_00175018	NM_002116	chr6_dbb_hap	1149538	1207092	chr6	29910247	29913661
tran	NA	LTCONS_00175019	MTCONS_00163846	chr6_dbb_hap	1151445	1152969	chr6	29855917	29858839
tran	NA	LTCONS_00175021	MTCONS_00174902	chr6_dbb_hap	1153053	1207092	chr6_apd_hap	1150151	1153525
tran	NA	LTCONS_00175021	MTCONS_00175194	chr6_dbb_hap	1153053	1207092	chr6_mcf_hap	1292291	1295619
tran	NA	LTCONS_00175021	NM_001242758	chr6_dbb_hap	1153053	1207092	chr6	29910247	29913661
tran	NA	LTCONS_00175021	NM_002116	chr6_dbb_hap	1153053	1207092	chr6	29910247	29913661
tran	NA	LTCONS_00175058	MTCONS_00164268	chr6_dbb_hap	3892081	3895208	chr6	32605183	32614759
tran	NA	LTCONS_00175058	MTCONS_00175212	chr6_dbb_hap	3892081	3895208	chr6_mcf_hap	3943999	3950241
tran	NA	LTCONS_00175066	MTCONS_00175377	chr6_dbb_hap	991865	993977	chr6_ssto_hap	1239669	1243999
tran	NA	LTCONS_00175075	NM_002341	chr6_dbb_hap	2834360	2835316	chr6	31548336	31550202
tran	NA	LTCONS_00175075	NM_009588	chr6_dbb_hap	2834360	2835316	chr6	31548336	31550202
tran	NA	LTCONS_00175189	NM_005104	chr6_mann_hap4	4393514	4393904	chr6	32936437	32949282
cis_mRNA dw20k	NA	LTCONS_00175215	MTCONS_00175213	chr6_mcf_hap	4181697	4184397	chr6_mcf_hap	4158810	4165465
cis_mRNA dw20k	NA	LTCONS_00175215	MTCONS_00175214	chr6_mcf_hap	4181697	4184397	chr6_mcf_hap	4158837	4165465
cis_mRNA dw20k	NA	LTCONS_00175255	MTCONS_00175193	chr6_mcf_hap	1295993	1296616	chr6_mcf_hap	1292291	1295619
cis_mRNA dw20k	NA	LTCONS_00175255	MTCONS_00175194	chr6_mcf_hap	1295993	1296616	chr6_mcf_hap	1292291	1295619

cis_mRNA_dw20k	NA	LTCONS_00175263	MTCONS_00175211	chr6_mcf_hap	3020593	3021040	chr6_mcf_hap	3016031	3017560
tran	NA	LTCONS_00175289	NM_205838	chr6_qbl_hap	2847640	2850328	chr6	31553956	31556686
tran	NA	LTCONS_00175291	NM_205838	chr6_qbl_hap	2847642	2850328	chr6	31553956	31556686
cis_mRNA_overlap	Lnc-AntiOverlap-mRNA	LTCONS_00175335	MTCONS_00175298	chr6_qbl_hap6	3076730	3080274	chr6_qbl_hap6	3076875	3091932
cis_mRNA_dw20k	NA	LTCONS_00175347	MTCONS_00175348	chr6_qbl_hap	4393588	4394829	chr6_qbl_hap	4394960	4396592
tran	NA	LTCONS_00175349	MTCONS_00175250	chr6_qbl_hap	4464082	4464933	chr6_mcf_hap	4705624	4710738
cis_mRNA_up10k	NA	LTCONS_00175360	MTCONS_00175296	chr6_qbl_hap	2974627	2975183	chr6_qbl_hap	2984784	2985374
cis_mRNA_dw20k	NA	LTCONS_00175372	MTCONS_00175396	chr6_ssto_hap	1028255	1030195	chr6_ssto_hap	1044456	1054653
cis_mRNA_dw20k	NA	LTCONS_00175374	MTCONS_00175396	chr6_ssto_hap	1032124	1034726	chr6_ssto_hap	1044456	1054653
cis_mRNA_dw20k	NA	LTCONS_00175444	MTCONS_00175385	chr6_ssto_hap	4063442	4063867	chr6_ssto_hap	4038602	4044437
tran	NA	LTCONS_00175447	NM_000593	chr6_ssto_hap	4243594	4245411	chr6	32812986	32821748
cis_mRNA_overlap	mRNA-CompleteIn-LncExon	LTCONS_00175883	MTCONS_00175886	chr7	7222246	7288280	chr7	7222246	7288280
cis_mRNA_overlap	Lnc-Overlap-mRNA	LTCONS_00176518	NM_001011666	chr7	28725598	28796001	chr7	28725598	28865511
cis_mRNA_overlap	Lnc-Overlap-mRNA	LTCONS_00176827	MTCONS_00176828	chr7	37723355	37842404	chr7	37723355	37940002
cis_mRNA_overlap	Lnc-Overlap-mRNA	LTCONS_00176827	MTCONS_00176829	chr7	37723355	37842404	chr7	37723368	37810320
cis_mRNA_up10k	NA	LTCONS_00176990	NM_001284315	chr7	44078648	44084089	chr7	44084239	44101315

cis_mRNA_overlap	Lnc-AntiOverlap-mRNA	LTCONS_00177887	MTCONS_00182637	chr7	74306474	74322330	chr7	74293720	74306717
cis_mRNA_overlap	Lnc-Overlap-mRNA	LTCONS_00177918	MTCONS_00177909	chr7	74988462	75027869	chr7	74988446	75006876
tran	NA	LTCONS_0017791	MTCONS_0018254	chr7	74988462	75027869	chr7	72440552	72476431
tran	NA	LTCONS_0017798	NM_001242713	chr7	76178628	76251680	chr7	102004308	102021080
cis_mRNA_dw20k	NA	LTCONS_00178046	NM_032936	chr7	77411258	77412338	chr7	77423045	77427747
cis_mRNA_dw20k	NA	LTCONS_00178046	NM_198467	chr7	77411258	77412338	chr7	77325743	77409120
cis_mRNA_dw20k	NA	LTCONS_00178057	MTCONS_00178049	chr7	77577222	77586821	chr7	77428109	77576648
cis_mRNA_overlap	mRNA-CompleteIn-LncExon	LTCONS_00178268	NM_001042717	chr7	89975979	90020769	chr7	89975979	90020769
tran	NA	LTCONS_0017827	MTCONS_0018041	chr7	89985215	89989807	chr7	155437203	155480457
cis_mRNA_up10k	NA	LTCONS_00178649	NM_178238	chr7	99933702	99949523	chr7	99955626	99965454
cis_mRNA_overlap	Lnc-AntiCompleteIn-mRNAIntron	LTCONS_00180064	MTCONS_00184666	chr7	149250175	149255693	chr7	149239848	149321905
tran	NA	LTCONS_0018006	NM_001127395	chr7	149250175	149255693	chr2	208473839	208490055
cis_mRNA_overlap	Lnc-Overlap-mRNA	LTCONS_00181340	NM_002137	chr7	26229556	26240413	chr7	26229556	26240413
cis_mRNA_dw20k	NA	LTCONS_00181714	NM_001003799	chr7	38279170	38289151	chr7	38299244	38313248
cis_mRNA_dw20k	NA	LTCONS_00181714	NM_032016	chr7	38279170	38289151	chr7	38217808	38270272
cis_mRNA_overlap	Lnc-Overlap-mRNA	LTCONS_00181720	NM_001003806	chr7	38279170	38359476	chr7	38299244	38313248
tran	NA	LTCONS_0018186	MTCONS_0018340	chr7	44022202	44025081	chr7	102178366	102283238
tran	NA	LTCONS_0018186	MTCONS_0018341	chr7	44022202	44025081	chr7	102178366	102283238
tran	NA	LTCONS_0018186	MTCONS_0018341	chr7	44022202	44025081	chr7	102277195	102312176
tran	NA	LTCONS_0018244	MTCONS_0009418	chr7	66146357	66147040	chr19	8554940	8567538

cis_mRNA up10k	NA	LTCONS_00183067	NM_004126	chr7	93543040	93544400	chr7	93551016	93555826
cis_mRNA overlap	Lnc-Overlap-mRNA	LTCONS_00183177	MTCONS_00183167	chr7	97578031	97601656	chr7	97481429	97601656
cis_mRNA dw20k	NA	LTCONS_00183229	NM_001039178	chr7	99046080	99046926	chr7	99055784	99063824
cis_mRNA _overlap	Lnc-AntiCompleteIn-mRNAExon	LTCONS_00183301	MTCONS_00178662	chr7	99968521	99969887	chr7	99949941	99969887
cis_mRNA _overlap	Lnc-AntiOverlap-mRNA	LTCONS_00183394	NM_001242713	chr7	102019618	102021008	chr7	102004308	102021080
cis_mRNA dw20k	NA	LTCONS_00183411	NM_006989	chr7	102184824	102213068	chr7	102220093	102257205
cis_mRNA overlap	Lnc-CompleteIn-	LTCONS_00183411	MTCONS_00183409	chr7	102184824	102213068	chr7	102178366	102283238
cis_mRNA overlap	Lnc-CompleteIn-	LTCONS_00183411	MTCONS_00183410	chr7	102184824	102213068	chr7	102178366	102283238
cis_mRNA overlap	Lnc-Overlap-mRNA	LTCONS_00183411	MTCONS_00183401	chr7	102184824	102213068	chr7	102113548	102213068
cis_mRNA up10k	NA	LTCONS_00183411	MTCONS_00183406	chr7	102184824	102213068	chr7	102178366	102184560
tran	NA	LTCONS_0018341	MTCONS_0018341	chr7	102184824	102213068	chr7	102277195	102312176
tran	NA	LTCONS_0018341	MTCONS_0018341	chr7	102184824	102213068	chr7	102307093	102312176
tran	NA	LTCONS_0018352	NM_001313692	chr7	104585097	104596178	chr21	35747779	35761452
cis_mRNA _overlap	Lnc-AntiOverlap-mRNA	LTCONS_00184594	NM_005435	chr7	144021788	144052811	chr7	144052489	144077725
cis_mRNA overlap	Lnc-Overlap-mRNA	LTCONS_00184628	MTCONS_00184630	chr7	148504464	148515915	chr7	148504464	148581441
cis_mRNA overlap	Lnc-Overlap-mRNA	LTCONS_00184628	MTCONS_00184632	chr7	148504464	148515915	chr7	148504464	148581441
cis_mRNA overlap	Lnc-Overlap-mRNA	LTCONS_00184628	NM_152998	chr7	148504464	148515915	chr7	148504464	148581441

cis_mRNA_overlap	Lnc-AntiOverlap-mRNA	LTCONS_00184687	MTCONS_00180095	chr7	149560505	149606784	chr7	149535509	149564627
cis_mRNA_overlap	Lnc-AntiOverlap-mRNA	LTCONS_00184687	NM_145230	chr7	149560505	149606784	chr7	149570057	149577801
cis_mRNA dw20k	NA	LTCONS_00184734	NM_001101312	chr7	150468099	150478201	chr7	150488376	150497621
cis_mRNA dw20k	NA	LTCONS_00184734	NM_001101314	chr7	150468099	150478201	chr7	150488376	150497621
cis_mRNA dw20k	NA	LTCONS_00184734	NM_014020	chr7	150468099	150478201	chr7	150488376	150498448
cis_mRNA dw20k	NA	LTCONS_00185339	NM_031267	chr7	40146286	40149139	chr7	39989959	40136733
cis_mRNA dw20k	NA	LTCONS_00185420	MTCONS_00177187	chr7	50476815	50479592	chr7	50343679	50472798
cis_mRNA dw20k	NA	LTCONS_00185498	NM_153033	chr7	66110232	66112443	chr7	66093868	66108216
cis_mRNA up10k	NA	LTCONS_00185528	MTCONS_00182654	chr7	74589791	74597928	chr7	74572392	74587785
cis_mRNA dw20k	NA	LTCONS_00185535	NM_001540	chr7	75947827	75950708	chr7	75931875	75933614
cis_mRNA up10k	NA	LTCONS_00185692	MTCONS_00183642	chr7	108097214	108099321	chr7	107788071	108096841
cis_mRNA dw20k	NA	LTCONS_00185811	NM_014390	chr7	127736816	127744967	chr7	127292202	127732659
cis_mRNA dw20k	NA	LTCONS_00185912	NM_001199692	chr7	150780922	150781248	chr7	150755299	150773614
cis_mRNA dw20k	NA	LTCONS_00186083	MTCONS_00186080	chr8	2102699	2153054	chr8	2066817	2093394
cis_mRNA dw20k	NA	LTCONS_00186083	NM_003970	chr8	2102699	2153054	chr8	1993158	2093380
cis_mRNA up10k	NA	LTCONS_00186747	NM_001018003	chr8	22402188	22415147	chr8	22423179	22433008

cis_mRNA overlap	Lnc-CompleteIn-	LTCONS_00186843	MTCONS_00186842	chr8	24185665	24187084	chr8	24182496	24196013
cis_mRNA up10k	NA	LTCONS_00187309	MTCONS_00187313	chr8	38758753	38766135	chr8	38770940	38831459
cis_mRNA overlap	Lnc-Overlap-mRNA	LTCONS_00187319	NM_003816	chr8	38942956	38962779	chr8	38854505	38962779
cis_mRNA overlap	Lnc-CompleteIn-	LTCONS_00188259	NM_016010	chr8	79615521	79622755	chr8	79578282	79631997
cis_mRNA overlap	Lnc-CompleteIn-	LTCONS_00188260	NM_016010	chr8	79622871	79627146	chr8	79578282	79631997
cis_mRNA overlap	Lnc-Overlap-mRNA	LTCONS_00189887	NM_001271156	chr8	144328991	144337650	chr8	144328991	144335761
cis_mRNA overlap	Lnc-Overlap-mRNA	LTCONS_00189887	NM_173832	chr8	144328991	144337650	chr8	144329098	144344875
cis_mRNA dw20k	NA	LTCONS_00190042	NM_145754	chr8	145702816	145721942	chr8	145691720	145699499
cis_mRNA up10k	NA	LTCONS_00190042	NM_032902	chr8	145702816	145721942	chr8	145722109	145727504
cis_mRNA dw20k	NA	LTCONS_00191343	NM_005195	chr8	48648560	48649296	chr8	48649476	48650726
tran	NA	LTCONS_0019209	MTCONS_0009079	chr8	79341527	79357856	chr19	20946830	20993757
cis_mRNA _overlap	mRNA-CompleteIn-LncExon	LTCONS_00192570	NM_000989	chr8	99053938	99057818	chr8	99053938	99057818
cis_mRNA overlap	Lnc-CompleteIn-	LTCONS_00193009	NM_001101676	chr8	119335914	119344415	chr8	119201695	119634184
cis_mRNA up10k	NA	LTCONS_00194202	NM_005195	chr8	48650940	48652004	chr8	48649476	48650726
cis_mRNA dw20k	NA	LTCONS_00194473	MTCONS_00188303	chr8	81442123	81443154	chr8	81398448	81441828
cis_mRNA up10k	NA	LTCONS_00194710	NM_000127	chr8	119124255	119125373	chr8	118811604	119124058
cis_mRNA overlap	Lnc-Overlap-mRNA	LTCONS_00196191	MTCONS_00196190	chr9	42671876	42691349	chr9	42669291	42714626
tran	NA	LTCONS_0019619	MTCONS_0019912	chr9	42671876	42691349	chr9	119766	179075

cis_mRNA overlap	Lnc-Overlap-mRNA	LTCONS_00196941	MTCONS_00196939	chr9	91933388	91947419	chr9	91926110	91974578
cis_mRNA _overlap	Lnc-AntiCompleteIn-mRNAIntron	LTCONS_00198564	NM_015033	chr9	132692640	132694142	chr9	132649466	132805473
cis_mRNA up10k	NA	LTCONS_00198907	MTCONS_00202628	chr9	139440664	139444346	chr9	139387379	139440257
cis_mRNA up10k	NA	LTCONS_00198907	NM_017617	chr9	139440664	139444346	chr9	139388885	139440238
cis_mRNA _overlap	mRNA-CompleteIn-LncExon	LTCONS_00198969	MTCONS_00198971	chr9	139866682	139876194	chr9	139869303	139876194
cis_mRNA dw20k	NA	LTCONS_00199026	NM_001128228	chr9	140083054	140084822	chr9	140086069	140095163
cis_mRNA dw20k	NA	LTCONS_00199062	MTCONS_00202781	chr9	140317847	140328858	chr9	140342023	140353786
cis_mRNA overlap	Lnc-Overlap-mRNA	LTCONS_00199103	NM_182905	chr9	14474	27640	chr9	14511	29739
cis_mRNA overlap	Lnc-CompleteIn-	LTCONS_00199236	MTCONS_00199229	chr9	3512630	3516647	chr9	3230727	3526001
cis_mRNA up10k	NA	LTCONS_00199236	MTCONS_00199228	chr9	3512630	3516647	chr9	3230727	3504659
cis_mRNA up10k	NA	LTCONS_00199944	MTCONS_00199922	chr9	35690737	35691408	chr9	35681990	35690053
tran	NA	LTCONS_0020027	MTCONS_0019635	chr9	70181647	70218729	chr9	70856861	70914951
tran	NA	LTCONS_0020027	MTCONS_0019912	chr9	70181647	70218729	chr9	119766	179075
tran	NA	LTCONS_0020027	MTCONS_0020025	chr9	70181647	70218729	chr9	69204457	69262556
cis_mRNA overlap	Lnc-Overlap-mRNA	LTCONS_00200434	NM_001102420	chr9	74966341	74979160	chr9	74966341	74980163
cis_mRNA _overlap	mRNA-CompleteIn-LncExon	LTCONS_00200442	NM_001102420	chr9	74966341	74980163	chr9	74966341	74980163
cis_mRNA overlap	Lnc-Overlap-mRNA	LTCONS_00200650	MTCONS_00200649	chr9	86532985	86536380	chr9	86505833	86536444

cis_mRNA up10k	NA	LTCONS_00200983	NM_001286723	chr9	96208781	96213900	chr9	96213978	96294871
cis_mRNA up10k	NA	LTCONS_00201323	NM_173199	chr9	102347480	102584088	chr9	102584137	102596341
cis_mRNA dw20k	NA	LTCONS_00201822	MTCONS_00197946	chr9	123940415	123964365	chr9	123837247	123939886
cis_mRNA dw20k	NA	LTCONS_00201822	MTCONS_00197949	chr9	123940415	123964365	chr9	123884060	123939886
cis_mRNA overlap	Lnc-Overlap-mRNA	LTCONS_00202736	MTCONS_00202737	chr9	140069236	140083057	chr9	140069236	140083057
cis_mRNA dw20k	NA	LTCONS_00202842	NM_004972	chr9	5138870	5140512	chr9	4985245	5128183
tran	NA	LTCONS_00203285	MTCONS_00174905	chr9	94877785	94878731	chr6_apd_hap	3110206	3122953
cis_mRNA up10k	NA	LTCONS_00203519	NM_033387	chr9	134153999	134157308	chr9	134133465	134151906
cis_mRNA dw20k	NA	LTCONS_00203582	MTCONS_00202792	chr9	140445618	140446142	chr9	140449359	140473387
tran	NA	LTCONS_0020361	NM_001190470	chrM	1825	2954	chr5	79945819	79946854
tran	NA	LTCONS_0020361	NM_001190470	chrM	2447	2897	chr5	79945819	79946854
cis_mRNA_overlap	Lnc-Overlap-mRNA	LTCONS_00203743	MTCONS_00203742	chrUn_gl000222	11347	23750	chrUn_gl000222	10800	19288
cis_mRNA dw20k	NA	LTCONS_00205193	NM_000291	chrX	77382553	77388838	chrX	77359666	77382324
cis_mRNA overlap	Lnc-Overlap-mRNA	LTCONS_00205688	NM_001257230	chrX	110924346	110933623	chrX	110924346	111003875
cis_mRNA overlap	Lnc-Overlap-mRNA	LTCONS_00206334	NM_001171907	chrX	148622519	148629876	chrX	148622519	148629876
cis_mRNA overlap	Lnc-Overlap-mRNA	LTCONS_00206681	MTCONS_00206690	chrX	3735563	3855896	chrX	3739722	3820041
cis_mRNA overlap	Lnc-Overlap-mRNA	LTCONS_00206692	MTCONS_00206680	chrX	3771051	3838787	chrX	3735563	3820041
cis_mRNA overlap	Lnc-Overlap-mRNA	LTCONS_00206692	MTCONS_00206690	chrX	3771051	3838787	chrX	3739722	3820041

cis_mRNA overlap	Lnc-Overlap-mRNA	LTCONS_00206693	MTCONS_00206690	chrX	3771051	3855490	chrX	3739722	3820041
cis_mRNA overlap	Lnc-Overlap-mRNA	LTCONS_00207120	NM_001034853	chrX	38166392	38186788	chrX	38143702	38186788
cis_mRNA overlap	Lnc-Overlap-mRNA	LTCONS_00209006	NM_001110792	chrX	153354496	153363188	chrX	153295686	153363188
cis_mRNA overlap	Lnc-Overlap-mRNA	LTCONS_00209006	NM_004992	chrX	153354496	153363188	chrX	153287264	153363188
cis_mRNA up10k	NA	LTCONS_00209006	MTCONS_00208996	chrX	153354496	153363188	chrX	153287264	153354096
cis_mRNA up10k	NA	LTCONS_00209272	NM_021083	chrX	37541493	37542947	chrX	37545133	37591383
tran	NA	LTCONS_0020956	MTCONS_0011100	chrX	106612945	106614557	chr2	148687966	148778463
tran	NA	LTCONS_0020956	NM_020297	chrX	106612945	106614557	chr12	21950324	22089628
cis_mRNA dw20k	NA	LTCONS_00209770	NM_001171132	chrX	153719800	153720429	chrX	153734490	153744566
cis_mRNA overlap	Lnc-Overlap-mRNA	LTCONS_00209977	MTCONS_00209974	chrY	15864978	16002295	chrY	15863379	15945236
tran	NA	LTCONS_0020997	NM_000216	chrY	16020047	16028067	chrX	8496915	8700227
cis_mRNA dw20k	NA	LTCONS_00210553	NM_004202	chrY	15820781	15821684	chrY	15815447	15817902
tran	NA	LTCONS_0021055	NM_000216	chrY	16030551	16031220	chrX	8496915	8700227
cis_mRNA dw20k	NA	LTCONS_00210583	MTCONS_00210024	chrY	21768208	21769029	chrY	21729199	21756123
cis_mRNA dw20k	NA	LTCONS_00210583	MTCONS_00210028	chrY	21768208	21769029	chrY	21729199	21756123
cis_mRNA dw20k	NA	LTCONS_00210583	MTCONS_00210038	chrY	21768208	21769029	chrY	21757066	21767704
tran	NA	NONHSAT000027.	MTCONS_0003295	chr1	134773	140566	chr11	126987	139612
cis_mRNA dw20k	NA	NONHSAT000295.2	MTCONS_00009093	chr1	1309818	1310539	chr1	1321091	1334718
cis_mRNA overlap	Lnc-Overlap-mRNA	NONHSAT000295.2	NM_001127229	chr1	1309818	1310539	chr1	1309110	1310580
cis_mRNA up10k	NA	NONHSAT000313.2	MTCONS_00009093	chr1	1342695	1345800	chr1	1321091	1334718

cis_mRNA up10k	NA	NONHSAT000323.2	NM_001145210	chr1	1365629	1368382	chr1	1353800	1356824
cis_mRNA dw20k	NA	NONHSAT000875.2	NM_001001998	chr1	11120263	11122696	chr1	11126670	11159967
cis_mRNA dw20k	NA	NONHSAT000909.2	NM_198545	chr1	11782187	11785914	chr1	11751781	11780336
cis_mRNA dw20k	NA	NONHSAT001116.2	MTCONS_00009826	chr1	16787443	16794976	chr1	16799390	16825752
cis_mRNA overlap	Lnc-Overlap-mRNA	NONHSAT001122.2	MTCONS_00009826	chr1	16799390	16802560	chr1	16799390	16825752
cis_mRNA overlap	Lnc-Overlap-mRNA	NONHSAT001128.2	MTCONS_00009826	chr1	16822681	16825749	chr1	16799390	16825752
tran	NA	NONHSAT001188.	NM_001412	chr1	17011093	17013295	chrX	20142636	20159966
cis_mRNA overlap	Lnc-Overlap-mRNA	NONHSAT001510.2	MTCONS_00010137	chr1	23607802	23613245	chr1	23607802	23670857
cis_mRNA dw20k	NA	NONHSAT001652.2	NM_001251977	chr1	24865207	24865735	chr1	24829387	24863510
cis_mRNA dw20k	NA	NONHSAT001652.2	NM_013441	chr1	24865207	24865735	chr1	24829387	24863510
cis_mRNA dw20k	NA	NONHSAT001732.2	NM_015627	chr1	25911711	25917708	chr1	25870076	25895377
cis_mRNA dw20k	NA	NONHSAT002060.2	NM_006762	chr1	31196182	31197911	chr1	31205315	31230683
cis_mRNA dw20k	NA	NONHSAT002390.2	NM_001317122	chr1	36391433	36395210	chr1	36348796	36389899
cis_mRNA dw20k	NA	NONHSAT002390.2	NM_012199	chr1	36391433	36395210	chr1	36348796	36389899
cis_mRNA up10k	NA	NONHSAT002484.2	MTCONS_00010870	chr1	38326369	38327252	chr1	38310245	38325292
cis_mRNA overlap	Lnc-Overlap-mRNA	NONHSAT002636.2	NM_022733	chr1	40879649	40881041	chr1	40839378	40888998
cis_mRNA dw20k	NA	NONHSAT002679.2	NM_001905	chr1	41480262	41509562	chr1	41444971	41478237
cis_mRNA dw20k	NA	NONHSAT002828.2	NM_001136215	chr1	44404330	44406129	chr1	44398992	44402912

cis_mRNA dw20k	NA	NONHSAT002838.2	MTCONS_00002343	chr1	44438106	44440475	chr1	44412478	44433694
cis_mRNA dw20k	NA	NONHSAT002908.2	MTCONS_00002445	chr1	45282505	45283445	chr1	45264998	45271667
cis_mRNA dw20k	NA	NONHSAT002908.2	MTCONS_00002447	chr1	45282505	45283445	chr1	45264998	45271667
cis_mRNA _overlap	Lnc-AntiOverlap-mRNA	NONHSAT003193.2	NM_001297663	chr1	51795326	51800996	chr1	51752930	51796236
cis_mRNA overlap	Lnc-CompleteIn-	NONHSAT003695.2	NM_002633	chr1	64121414	64121716	chr1	64058947	64125917
cis_mRNA up10k	NA	NONHSAT004368.2	NM_032270	chr1	90094731	90097764	chr1	90098644	90185094
tran	NA	NONHSAT004648.	MTCONS_0009079	chr1	95628775	95699538	chr19	20946830	20993757
cis_mRNA overlap	Lnc-Overlap-mRNA	NONHSAT004982.2	MTCONS_00004420	chr1	108815761	108880009	chr1	108815761	108881772
cis_mRNA dw20k	NA	NONHSAT005088.2	NM_033088	chr1	110600962	110605177	chr1	110577223	110597263
cis_mRNA _overlap	Lnc-AntiOverlap-mRNA	NONHSAT005152.2	NM_018372	chr1	111486089	111495470	chr1	111489812	111506566
cis_mRNA up10k	NA	NONHSAT005180.2	NM_002557	chr1	111973668	111975690	chr1	111956937	111970399
cis_mRNA dw20k	NA	NONHSAT005456.2	NM_001767	chr1	117317726	117318245	chr1	117297086	117311851
cis_mRNA dw20k	NA	NONHSAT005482.2	NM_006699	chr1	118069961	118071491	chr1	117910085	118068320
cis_mRNA dw20k	NA	NONHSAT005609.2	MTCONS_00005012	chr1	121133256	121134581	chr1	121107167	121130831
tran	NA	NONHSAT005833.	MTCONS_0000984	chr1	148342064	148343810	chr1	16888603	16940100
cis_mRNA overlap	Lnc-CompleteIn-	NONHSAT005839.2	NM_001278267	chr1	148250267	148251686	chr1	144168359	148590603
cis_mRNA overlap	Lnc-CompleteIn-	NONHSAT006062.2	NM_001037675	chr1	147594624	147596613	chr1	144823812	148346929

cis_mRNA overlap	Lnc-CompleteIn-	NONHSAT006062.2	NM_001278267	chr1	147594624	147596613	chr1	144168359	148590603
cis_mRNA overlap	Lnc-CompleteIn-	NONHSAT006062.2	NM_015383	chr1	147594624	147596613	chr1	144159762	148024925
cis_mRNA overlap	Lnc-Overlap-mRNA	NONHSAT006062.2	NM_001101663	chr1	147594624	147596613	chr1	147574323	147608092
tran	NA	NONHSAT006062.	MTCONS_0000984	chr1	147594624	147596613	chr1	16888603	16940100
cis_mRNA up10k	NA	NONHSAT006140.2	NM_173638	chr1	148598398	148599531	chr1	148560846	148596266
cis_mRNA overlap	Lnc-CompleteIn-	NONHSAT006177.2	MTCONS_00005125	chr1	149239868	149265510	chr1	149239868	149265510
cis_mRNA dw20k	NA	NONHSAT006252.2	NM_001040874	chr1	149802876	149804109	chr1	149813785	149814318
cis_mRNA up10k	NA	NONHSAT006252.2	NM_001034077	chr1	149802876	149804109	chr1	149804221	149804616
cis_mRNA up10k	NA	NONHSAT006252.2	NM_003548	chr1	149802876	149804109	chr1	149804221	149804616
cis_mRNA up10k	NA	NONHSAT006253.2	NM_001040874	chr1	149814608	149815100	chr1	149813785	149814318
cis_mRNA up10k	NA	NONHSAT006253.2	NM_003516	chr1	149814608	149815100	chr1	149822628	149823161
tran	NA	NONHSAT006253.	MTCONS_0001348	chr1	149814608	149815100	chr1	149369374	149400546
tran	NA	NONHSAT006253.	NM_003522	chr1	149814608	149815100	chr6	26199787	26200216
tran	NA	NONHSAT006253.	NM_003523	chr1	149814608	149815100	chr6	26184024	26184458
tran	NA	NONHSAT006253.	NM_003524	chr1	149814608	149815100	chr6	26251879	26252303
tran	NA	NONHSAT006253.	NM_003525	chr1	149814608	149815100	chr6	26273204	26273640
tran	NA	NONHSAT006253.	NM_003526	chr1	149814608	149815100	chr6	26123695	26124132
tran	NA	NONHSAT006253.	NM_021062	chr1	149814608	149815100	chr6	26043455	26043885
cis_mRNA dw20k	NA	NONHSAT006254.2	MTCONS_00013505	chr1	149816065	149820591	chr1	149828245	149832807
tran	NA	NONHSAT006457.	MTCONS_0000984	chr1	151967007	152015250	chr1	16888603	16940100
cis_mRNA overlap	Lnc-Overlap-mRNA	NONHSAT006571.2	NM_001030	chr1	153963239	153964632	chr1	153963239	153964631
cis_mRNA up10k	NA	NONHSAT006652.2	NM_138300	chr1	154934301	154935099	chr1	154929502	154934258

cis_mRNA overlap	Lnc-CompleteIn-	NONHSAT006771.2	NM_152280	chr1	155839565	155841887	chr1	155829260	155854990
cis_mRNA up10k	NA	NONHSAT006882.2	MTCONS_00014113	chr1	156471576	156478185	chr1	156432660	156471490
tran	NA	NONHSAT006985.	NM_004879	chr1	158423988	158425063	chr11	125439283	125454584
cis_mRNA dw20k	NA	NONHSAT007028.2	NM_001277224	chr1	159879884	159880344	chr1	159887897	159893507
cis_mRNA dw20k	NA	NONHSAT007037.2	NM_145167	chr1	159994743	159996584	chr1	159997462	160001783
cis_mRNA overlap	Lnc-Overlap-mRNA	NONHSAT007187.2	MTCONS_00006064	chr1	161551129	161559487	chr1	161519677	161575814
cis_mRNA overlap	Lnc-Overlap-mRNA	NONHSAT007188.2	NM_201563	chr1	161551129	161559676	chr1	161551129	161571010
cis_mRNA overlap	Lnc-Overlap-mRNA	NONHSAT007189.2	NM_201563	chr1	161551136	161561081	chr1	161551129	161571010
cis_mRNA overlap	Lnc-Overlap-mRNA	NONHSAT007190.2	MTCONS_00006061	chr1	161561940	161575452	chr1	161519667	161566519
cis_mRNA overlap	Lnc-Overlap-mRNA	NONHSAT007190.2	NM_201563	chr1	161561940	161575452	chr1	161551129	161571010
cis_mRNA dw20k	NA	NONHSAT007191.2	MTCONS_00006061	chr1	161569361	161574927	chr1	161519667	161566519
cis_mRNA dw20k	NA	NONHSAT007204.2	MTCONS_00006101	chr1	161735115	161736017	chr1	161719558	161726954
cis_mRNA overlap	Lnc-CompleteIn-	NONHSAT007220.2	NM_001184763	chr1	162495162	162499391	chr1	162466964	162499419
cis_mRNA dw20k	NA	NONHSAT007232.2	NM_003115	chr1	162579813	162580921	chr1	162531296	162569633
cis_mRNA overlap	Lnc-CompleteIn-	NONHSAT007429.2	MTCONS_00006280	chr1	168218462	168220378	chr1	168195255	168232401
cis_mRNA up10k	NA	NONHSAT007688.2	NM_001122770	chr1	173833622	173836168	chr1	173837493	173855774
tran	NA	NONHSAT007690.	MTCONS_0018086	chr1	173834093	173837127	chr7	5659672	5821361
cis_mRNA dw20k	NA	NONHSAT007744.2	MTCONS_00006466	chr1	174966616	174969328	chr1	174128552	174964445

cis_mRNA up10k	NA	NONHSAT008235.2	MTCONS_00006591	chr1	179922319	179923575	chr1	179923908	180084015
cis_mRNA dw20k	NA	NONHSAT008282.2	NM_016545	chr1	181060245	181062035	chr1	181057638	181059979
cis_mRNA dw20k	NA	NONHSAT008823.2	MTCONS_00007176	chr1	202518232	202523878	chr1	202317830	202499036
cis_mRNA overlap	Lnc-Overlap-mRNA	NONHSAT008955.2	NM_002393	chr1	204507003	204512059	chr1	204485507	204527248
cis_mRNA dw20k	NA	NONHSAT009067.2	NM_014002	chr1	206677281	206677789	chr1	206643586	206670223
cis_mRNA up10k	NA	NONHSAT009281.2	MTCONS_00007599	chr1	211431307	211432191	chr1	211432708	211489725
cis_mRNA overlap	Lnc-CompleteIn-	NONHSAT009285.2	MTCONS_00007600	chr1	211477892	211479780	chr1	211432708	211489725
cis_mRNA overlap	Lnc-Overlap-mRNA	NONHSAT009860.2	MTCONS_00016306	chr1	226047363	226053911	chr1	226033233	226070420
cis_mRNA overlap	Lnc-Overlap-mRNA	NONHSAT009860.2	NM_014698	chr1	226047363	226053911	chr1	226033233	226070420
cis_mRNA overlap	Lnc-CompleteIn-	NONHSAT009902.2	MTCONS_00016347	chr1	226920314	226922612	chr1	226894984	226927067
cis_mRNA up10k	NA	NONHSAT009946.2	MTCONS_00016372	chr1	227931532	227934892	chr1	227916314	227923112
cis_mRNA up10k	NA	NONHSAT009965.2	NM_024319	chr1	228295468	228296717	chr1	228288428	228291163
cis_mRNA overlap	Lnc-CompleteIn-	NONHSAT010086.2	MTCONS_00016461	chr1	229589677	229591617	chr1	229577044	229644088
cis_mRNA dw20k	NA	NONHSAT010428.2	NM_000254	chr1	237070071	237072046	chr1	236958581	237067281
tran	NA	NONHSAT010583.	MTCONS_0015617	chr1	243219612	243265046	chr5	180750476	180755196
cis_mRNA dw20k	NA	NONHSAT010618.2	NM_006642	chr1	243665066	243666895	chr1	243419307	243663393
cis_mRNA overlap	Lnc-Overlap-mRNA	NONHSAT010657.2	MTCONS_00016966	chr1	245003608	245010690	chr1	245003545	245027840
tran	NA	NONHSAT010791.	MTCONS_0003277	chr1	247803351	247827145	chr11	129685741	129733498

cis_mRNA overlap	Lnc-CompleteIn-	NONHSAT010902.2	NM_014023	chr10	1147572	1149291	chr10	1102776	1178237
cis_mRNA overlap	Lnc-CompleteIn-	NONHSAT010921.2	MTCONS_00023158	chr10	1259747	1260413	chr10	1223253	1282544
cis_mRNA overlap	Lnc-CompleteIn-	NONHSAT010924.2	MTCONS_00023158	chr10	1259983	1260562	chr10	1223253	1282544
cis_mRNA overlap	Lnc-CompleteIn-	NONHSAT011406.2	MTCONS_00019072	chr10	11219703	11221256	chr10	11206993	11378672
cis_mRNA dw20k	NA	NONHSAT011652.2	MTCONS_00019277	chr10	18967049	18970567	chr10	18948313	18966940
cis_mRNA dw20k	NA	NONHSAT011652.2	NM_178815	chr10	18967049	18970567	chr10	18948313	18966940
cis_mRNA overlap	Lnc-CompleteIn-	NONHSAT011826.2	NM_019043	chr10	26823287	26824417	chr10	26727266	26856732
cis_mRNA overlap	Lnc-CompleteIn-	NONHSAT011908.2	NM_001256410	chr10	27810962	27811539	chr10	27793103	27831166
cis_mRNA overlap	Lnc-CompleteIn-	NONHSAT011908.2	NM_021252	chr10	27810962	27811539	chr10	27793103	27831166
tran	NA	NONHSAT012891.	NM_001007094	chr10	43016235	43048270	chr10	38383264	38412278
cis_mRNA _overlap	Lnc-AntiCompleteIn-mRNAIntron	NONHSAT013359.2	NM_001290117	chr10	51592090	51594455	chr10	51384299	51734610
cis_mRNA dw20k	NA	NONHSAT013371.2	NM_001077665	chr10	51780942	51827563	chr10	51748078	51770259
cis_mRNA up10k	NA	NONHSAT013371.2	MTCONS_00020277	chr10	51780942	51827563	chr10	51827648	51893269
cis_mRNA up10k	NA	NONHSAT013371.2	NM_001005751	chr10	51780942	51827563	chr10	51827648	51893269
cis_mRNA up10k	NA	NONHSAT013406.2	NM_006258	chr10	52822339	52828313	chr10	52834234	54058110
cis_mRNA overlap	Lnc-CompleteIn-	NONHSAT013486.2	NM_152230	chr10	59960595	59961847	chr10	59951278	60027694
cis_mRNA overlap	Lnc-CompleteIn-	NONHSAT013724.2	MTCONS_00025069	chr10	70266399	70277099	chr10	70236960	70277099

cis_mRNA dw20k	NA	NONHSAT013828.2	NM_001142648	chr10	71897737	71906496	chr10	71909961	71930285
tran	NA	NONHSAT013946.	NM_001967	chr10	74959584	74960983	chr3	186501361	186507685
cis_mRNA dw20k	NA	NONHSAT015230.2	NM_001278407	chr10	81903200	81907928	chr10	81914880	81965433
cis_mRNA up10k	NA	NONHSAT015262.2	NM_207372	chr10	82289325	82293237	chr10	82297658	82406316
cis_mRNA up10k	NA	NONHSAT015375.2	NM_015045	chr10	88281750	88284806	chr10	88195013	88281541
tran	NA	NONHSAT015402.	MTCONS_0009437	chr10	88965591	89102315	chr19	11487649	11495018
tran	NA	NONHSAT015421.	MTCONS_0011059	chr10	89102168	89103331	chr2	128619205	128643937
tran	NA	NONHSAT015444.	MTCONS_0008799	chr10	89369920	89419760	chr18	44179670	44236996
cis_mRNA_overlap	Lnc-AntiCompleteIn-mRNAIntron	NONHSAT015489.2	NM_000043	chr10	90751179	90752732	chr10	90750316	90776818
cis_mRNA dw20k	NA	NONHSAT015843.2	NM_015652	chr10	98752207	98755716	chr10	98741041	98745585
tran	NA	NONHSAT016130.	MTCONS_0009682	chr10	104213597	104216049	chr19	52370214	52408305
cis_mRNA dw20k	NA	NONHSAT016379.2	NM_007373	chr10	112778764	112782745	chr10	112679301	112773425
cis_mRNA overlap	Lnc-CompleteIn-	NONHSAT016421.2	NM_001198526	chr10	114747235	114747624	chr10	114710009	114927436
cis_mRNA overlap	Lnc-CompleteIn-	NONHSAT016422.2	MTCONS_00022336	chr10	114843553	114846535	chr10	114710009	114915765
cis_mRNA overlap	Lnc-Overlap-mRNA	NONHSAT016441.2	NM_198514	chr10	115671515	115676949	chr10	115614391	115672265
cis_mRNA_overlap	Lnc-AntiOverlap-mRNA	NONHSAT016513.2	NM_003054	chr10	118999111	119001434	chr10	119000584	119038941
cis_mRNA dw20k	NA	NONHSAT016573.2	NM_001134672	chr10	120065401	120067578	chr10	120068572	120101839
cis_mRNA up10k	NA	NONHSAT016640.2	MTCONS_00026686	chr10	121356543	121359514	chr10	121332978	121356541
cis_mRNA dw20k	NA	NONHSAT016711.2	NM_017615	chr10	123687706	123705250	chr10	123716603	123734743

cis_mRNA dw20k	NA	NONHSAT016827.2	NM_022126	chr10	126307653	126311871	chr10	126150341	126302710
cis_mRNA up10k	NA	NONHSAT017220.2	MTCONS_00028059	chr11	287441	288726	chr11	289418	295688
cis_mRNA up10k	NA	NONHSAT017220.2	MTCONS_00028060	chr11	287441	288726	chr11	289418	295688
cis_mRNA up10k	NA	NONHSAT017221.2	MTCONS_00028060	chr11	287702	288773	chr11	289418	295688
cis_mRNA up10k	NA	NONHSAT017222.2	MTCONS_00028059	chr11	288038	288987	chr11	289418	295688
cis_mRNA up10k	NA	NONHSAT017222.2	MTCONS_00028060	chr11	288038	288987	chr11	289418	295688
cis_mRNA dw20k	NA	NONHSAT017317.2	MTCONS_00033075	chr11	777489	786722	chr11	799179	809935
cis_mRNA overlap	Lnc-CompleteIn-	NONHSAT017332.2	NM_001004	chr11	809936	812876	chr11	809936	812876
cis_mRNA overlap	Lnc-Overlap-mRNA	NONHSAT017339.2	MTCONS_00028159	chr11	822016	823572	chr11	818901	823813
cis_mRNA overlap	Lnc-CompleteIn-	NONHSAT017381.2	NM_002457	chr11	1096364	1097364	chr11	1074875	1104416
cis_mRNA dw20k	NA	NONHSAT017537.2	MTCONS_00028342	chr11	2965661	2973999	chr11	2923512	2946476
cis_mRNA up10k	NA	NONHSAT017628.2	NM_003156	chr11	3875842	3876629	chr11	3876933	4114440
cis_mRNA up10k	NA	NONHSAT017730.2	NM_144666	chr11	6509416	6510607	chr11	6518526	6593254
cis_mRNA dw20k	NA	NONHSAT017735.2	NM_000391	chr11	6624872	6625650	chr11	6633997	6640692
cis_mRNA dw20k	NA	NONHSAT017741.2	NM_006284	chr11	6630110	6630872	chr11	6632048	6633475
cis_mRNA_overlap	Lnc-AntiOverlap-mRNA	NONHSAT017896.2	NM_001286095	chr11	8986222	8997830	chr11	8968748	8986558

cis_mRNA_overlap	Lnc-AntiOverlap-mRNA	NONHSAT017896.2	NM_020644	chr11	8986222	8997830	chr11	8968748	8986320
tran	NA	NONHSAT017916.	NM_018457	chr11	9332831	9333236	chr12	53835433	53840427
cis_mRNA up10k	NA	NONHSAT017959.2	NM_001124	chr11	10323789	10326390	chr11	10326527	10328949
cis_mRNA dw20k	NA	NONHSAT018043.2	NM_001282668	chr11	12237841	12241075	chr11	12132123	12229916
cis_mRNA dw20k	NA	NONHSAT018228.2	MTCONS_00028885	chr11	17402196	17405078	chr11	17372656	17398868
cis_mRNA overlap	Lnc-Overlap-mRNA	NONHSAT018588.2	NM_152316	chr11	30358557	30359978	chr11	30344646	30359770
cis_mRNA overlap	Lnc-Overlap-mRNA	NONHSAT018589.2	NM_152316	chr11	30358557	30359978	chr11	30344646	30359770
cis_mRNA dw20k	NA	NONHSAT018688.2	NM_018393	chr11	33097696	33101000	chr11	33060963	33095109
cis_mRNA dw20k	NA	NONHSAT018796.2	NM_017583	chr11	35835882	35839557	chr11	35684300	35832603
tran	NA	NONHSAT021174.	MTCONS_0017024	chr11	46450163	46450609	chr6	34254973	34393902
cis_mRNA up10k	NA	NONHSAT021236.2	NM_001080547	chr11	47404699	47417191	chr11	47376409	47400127
cis_mRNA up10k	NA	NONHSAT021236.2	NM_003120	chr11	47404699	47417191	chr11	47376409	47400127
cis_mRNA overlap	Lnc-CompleteIn-	NONHSAT021271.2	MTCONS_00034510	chr11	47738779	47746103	chr11	47738062	47789030
tran	NA	NONHSAT021466.	NM_001967	chr11	58009515	58010569	chr3	186501361	186507685
cis_mRNA up10k	NA	NONHSAT022268.2	MTCONS_00030555	chr11	66012267	66015172	chr11	66023374	66035332
cis_mRNA up10k	NA	NONHSAT022331.2	NM_006328	chr11	66381671	66383832	chr11	66384053	66397397
cis_mRNA dw20k	NA	NONHSAT022416.2	MTCONS_00030701	chr11	67172850	67174446	chr11	67155110	67165883
cis_mRNA overlap	Lnc-Overlap-mRNA	NONHSAT022416.2	NM_198517	chr11	67172850	67174446	chr11	67171384	67177561

cis_mRNA dw20k	NA	NONHSAT022500.2	NM_006019	chr11	67819100	67821228	chr11	67806462	67818366
cis_mRNA dw20k	NA	NONHSAT022500.2	NM_006053	chr11	67819100	67821228	chr11	67810447	67818366
cis_mRNA dw20k	NA	NONHSAT022502.2	NM_006053	chr11	67820290	67821229	chr11	67810447	67818366
cis_mRNA _overlap	Lnc-AntiOverlap-mRNA	NONHSAT022744.2	MTCONS_00035553	chr11	71725337	71731956	chr11	71713910	71752196
cis_mRNA up10k	NA	NONHSAT022801.2	MTCONS_00031029	chr11	72523491	72525129	chr11	72525451	72547790
cis_mRNA dw20k	NA	NONHSAT022919.2	MTCONS_00035675	chr11	74541627	74543555	chr11	74551951	74660232
cis_mRNA dw20k	NA	NONHSAT022919.2	MTCONS_00035676	chr11	74541627	74543555	chr11	74551951	74660232
cis_mRNA dw20k	NA	NONHSAT022919.2	MTCONS_00035677	chr11	74541627	74543555	chr11	74551951	74660232
cis_mRNA dw20k	NA	NONHSAT023292.2	NM_024678	chr11	78135028	78140882	chr11	78147007	78285909
cis_mRNA up10k	NA	NONHSAT023374.2	MTCONS_00035912	chr11	82783164	82784236	chr11	82684175	82782965
cis_mRNA dw20k	NA	NONHSAT023390.2	MTCONS_00031411	chr11	82896837	82905290	chr11	82868137	82896835
cis_mRNA dw20k	NA	NONHSAT023583.2	NM_012124	chr11	89927674	89931288	chr11	89933598	89956532
cis_mRNA dw20k	NA	NONHSAT023765.2	NM_144665	chr11	94883703	94892312	chr11	94898677	94964246
cis_mRNA overlap	Lnc-CompleteIn-	NONHSAT024263.2	MTCONS_00036632	chr11	113691578	113692607	chr11	113668597	113746292
cis_mRNA dw20k	NA	NONHSAT024388.2	MTCONS_00032192	chr11	117070040	117075503	chr11	117049418	117068161
cis_mRNA dw20k	NA	NONHSAT024388.2	NM_001040455	chr11	117070040	117075503	chr11	117049939	117068161
cis_mRNA overlap	Lnc-Overlap-mRNA	NONHSAT024388.2	NM_001001522	chr11	117070040	117075503	chr11	117070040	117075508

tran	NA	NONHSAT024395.	MTCONS_0004877	chr11	117080080	117080943	chr13	24824591	24881212
cis_mRNA overlap	Lnc-Overlap-mRNA	NONHSAT024459.2	NM_000732	chr11	118209789	118213459	chr11	118209789	118213459
cis_mRNA overlap	Lnc-Overlap-mRNA	NONHSAT024459.2	NM_001040651	chr11	118209789	118213459	chr11	118209789	118213459
cis_mRNA dw20k	NA	NONHSAT024523.2	MTCONS_00036873	chr11	118756550	118758451	chr11	118766850	118781613
cis_mRNA dw20k	NA	NONHSAT024523.2	MTCONS_00036877	chr11	118756550	118758451	chr11	118766850	118796280
cis_mRNA dw20k	NA	NONHSAT024893.2	NM_138961	chr11	124609829	124617102	chr11	124623019	124632223
cis_mRNA _overlap	mRNA-CompleteIn-LncExon	NONHSAT024893.2	NM_001126181	chr11	124609829	124617102	chr11	124609829	124617102
cis_mRNA up10k	NA	NONHSAT024901.2	MTCONS_00037108	chr11	124670301	124679484	chr11	124636394	124670300
cis_mRNA up10k	NA	NONHSAT024901.2	NM_001308027	chr11	124670301	124679484	chr11	124636394	124670300
cis_mRNA _overlap	Lnc-AntiOverlap-mRNA	NONHSAT025023.2	NM_014026	chr11	126210853	126225482	chr11	126173647	126215648
cis_mRNA overlap	Lnc-CompleteIn-	NONHSAT025074.2	NM_002017	chr11	128659852	128662730	chr11	128563811	128683162
cis_mRNA dw20k	NA	NONHSAT025075.2	NM_001271010	chr11	128691118	128691548	chr11	128563811	128683162
cis_mRNA overlap	Lnc-Overlap-mRNA	NONHSAT025080.2	NM_001256088	chr11	128769460	128776126	chr11	128769460	128775964
cis_mRNA overlap	Lnc-Overlap-mRNA	NONHSAT025113.2	MTCONS_00032781	chr11	129939943	129941235	chr11	129939716	130014706
cis_mRNA overlap	Lnc-Overlap-mRNA	NONHSAT025114.2	MTCONS_00032781	chr11	129939943	129941235	chr11	129939716	130014706
cis_mRNA dw20k	NA	NONHSAT025149.2	NM_014758	chr11	130736149	130740142	chr11	130745766	130786382
cis_mRNA dw20k	NA	NONHSAT025239.2	MTCONS_00037412	chr11	134020575	134021747	chr11	134022337	134095089

cis_mRNA dw20k	NA	NONHSAT025325.2	NM_001042603	chr12	383120	386289	chr12	389223	498621
cis_mRNA up10k	NA	NONHSAT025475.2	MTCONS_00038292	chr12	2901746	2904044	chr12	2904108	2914587
cis_mRNA dw20k	NA	NONHSAT025501.2	NM_201441	chr12	3150603	3154116	chr12	3068478	3149842
cis_mRNA up10k	NA	NONHSAT026095.2	NM_014865	chr12	6602959	6603267	chr12	6603298	6641132
cis_mRNA dw20k	NA	NONHSAT026106.2	NM_001258310	chr12	6645713	6646179	chr12	6666036	6677498
cis_mRNA dw20k	NA	NONHSAT026202.2	NM_002831	chr12	7076500	7076769	chr12	7060434	7070479
cis_mRNA up10k	NA	NONHSAT026230.2	NM_014718	chr12	7260904	7274447	chr12	7282967	7311530
cis_mRNA up10k	NA	NONHSAT026240.2	MTCONS_00038589	chr12	7319270	7341665	chr12	7341681	7364079
cis_mRNA up10k	NA	NONHSAT026240.2	NM_001131025	chr12	7319270	7341665	chr12	7342282	7364079
cis_mRNA dw20k	NA	NONHSAT026265.2	MTCONS_00043516	chr12	7878128	7879277	chr12	7881241	7900308
cis_mRNA dw20k	NA	NONHSAT026265.2	NM_130441	chr12	7878128	7879277	chr12	7882011	7902069
tran	NA	NONHSAT026317.	MTCONS_0007428	chr12	8388478	8390769	chr16	74357188	74402046
tran	NA	NONHSAT026344.	NM_003168	chr12	8664970	8665322	chr17	56422536	56429599
cis_mRNA overlap	Lnc-CompleteIn-	NONHSAT026384.2	MTCONS_00038714	chr12	9217781	9219303	chr12	9142221	9368017
cis_mRNA overlap	Lnc-CompleteIn-	NONHSAT026390.2	MTCONS_00038714	chr12	9287680	9288187	chr12	9142221	9368017
cis_mRNA overlap	Lnc-Overlap-mRNA	NONHSAT026890.2	MTCONS_00043760	chr12	10741078	10752434	chr12	10747602	10752434
tran	NA	NONHSAT026913.	NM_176890	chr12	11117024	11117951	chr12	11138512	11139511
tran	NA	NONHSAT026913.	NM_176889	chr12	11117024	11117951	chr12	11148561	11150474
tran	NA	NONHSAT026913.	NM_176887	chr12	11117024	11117951	chr12	11213964	11214893
cis_mRNA dw20k	NA	NONHSAT026917.2	NM_176887	chr12	11200931	11201855	chr12	11213964	11214893

tran	NA	NONHSAT026917.	NM_176888	chr12	11200931	11201855	chr12	11174218	11175219
cis_mRNA dw20k	NA	NONHSAT026918. 2	NM_176884	chr12	11229368	11231770	chr12	11243886	11244912
cis_mRNA overlap	Lnc-CompleteIn-	NONHSAT026925. 2	NM_001271592	chr12	11323780	11325723	chr12	11323780	11328619
cis_mRNA overlap	Lnc-CompleteIn-	NONHSAT026975. 2	MTCONS_0004384 8	chr12	12627157	12629731	chr12	12626216	12715558
cis_mRNA overlap	Lnc-CompleteIn-	NONHSAT026976. 2	MTCONS_0004384 8	chr12	12627157	12629731	chr12	12626216	12715558
tran	NA	NONHSAT027028.	NM_019860	chr12	13153354	13157764	chr10	92500576	92617671
cis_mRNA overlap	Lnc-CompleteIn-	NONHSAT027320. 2	MTCONS_0004403 7	chr12	24430184	24715427	chr12	23685231	24715525
cis_mRNA overlap	Lnc-Overlap-mRNA	NONHSAT027320. 2	MTCONS_0004403 6	chr12	24430184	24715427	chr12	23685231	24715525
cis_mRNA overlap	Lnc-Overlap-mRNA	NONHSAT027320. 2	MTCONS_0004404 0	chr12	24430184	24715427	chr12	23685231	24715525
cis_mRNA dw20k	NA	NONHSAT027600. 2	NM_018169	chr12	32154358	32155125	chr12	32112353	32146041
cis_mRNA dw20k	NA	NONHSAT027801. 2	NM_031292	chr12	44112796	44117803	chr12	44122412	44152620
cis_mRNA _overlap	Lnc-AntiOverlap-mRNA	NONHSAT027898. 2	NM_138371	chr12	47599681	47610191	chr12	47473386	47630446
cis_mRNA _overlap	Lnc-AntiCompleteIn -mRNAIntron	NONHSAT027901. 2	MTCONS_0003976 5	chr12	47599682	47601754	chr12	47473386	47630942
cis_mRNA _overlap	Lnc-AntiCompleteIn -mRNAIntron	NONHSAT027901. 2	NM_138371	chr12	47599682	47601754	chr12	47473386	47630446
cis_mRNA _overlap	Lnc-AntiOverlap-mRNA	NONHSAT027905. 2	MTCONS_0003976 6	chr12	47599685	47610032	chr12	47604580	47630942
cis_mRNA _overlap	Lnc-AntiCompleteIn -mRNAIntron	NONHSAT027908. 2	MTCONS_0003976 5	chr12	47602203	47610226	chr12	47473386	47630942

tran	NA	NONHSAT028044.	NM_001007074	chr12	49297208	49297892	chr3	12876444	12881949
tran	NA	NONHSAT028155.	MTCONS_0017304	chr12	50305763	50314301	chr6	150117711	150185480
cis_mRNA overlap	Lnc-CompleteIn-	NONHSAT028182.2	MTCONS_00044756	chr12	50579363	50585146	chr12	50569563	50616488
cis_mRNA up10k	NA	NONHSAT028320.2	MTCONS_00040096	chr12	52430963	52437695	chr12	52443418	52453677
cis_mRNA up10k	NA	NONHSAT028320.2	MTCONS_00040100	chr12	52430963	52437695	chr12	52445186	52453677
cis_mRNA overlap	Lnc-Overlap-mRNA	NONHSAT028755.2	MTCONS_00040410	chr12	56510374	56511615	chr12	56510374	56516280
cis_mRNA overlap	Lnc-Overlap-mRNA	NONHSAT029118.2	NM_014254	chr12	64173897	64195983	chr12	64173583	64203338
cis_mRNA dw20k	NA	NONHSAT029263.2	NM_006482	chr12	68072174	68078832	chr12	68042512	68056444
cis_mRNA up10k	NA	NONHSAT029293.2	NM_020128	chr12	68726299	68729466	chr12	68718785	68726161
cis_mRNA _overlap	Lnc-AntiOverlap-mRNA	NONHSAT029665.2	MTCONS_00045862	chr12	80328681	80334448	chr12	80165069	80328978
cis_mRNA dw20k	NA	NONHSAT029682.2	NM_004664	chr12	81186299	81189685	chr12	81191171	81331694
cis_mRNA _overlap	Lnc-AntiOverlap-mRNA	NONHSAT030138.2	NM_001032284	chr12	98906751	98910200	chr12	98909351	98944157
cis_mRNA _overlap	Lnc-AntiOverlap-mRNA	NONHSAT030138.2	NM_001307975	chr12	98906751	98910200	chr12	98909351	98944157
cis_mRNA overlap	Lnc-Overlap-mRNA	NONHSAT030480.2	NM_152772	chr12	106697271	106705010	chr12	106696569	106740792
cis_mRNA dw20k	NA	NONHSAT030590.2	NM_001001655	chr12	109510482	109525751	chr12	109525993	109531293
cis_mRNA up10k	NA	NONHSAT031446.2	MTCONS_00042350	chr12	122233165	122240870	chr12	122241894	122271045
cis_mRNA dw20k	NA	NONHSAT031449.2	NM_001080825	chr12	122233178	122237538	chr12	122150658	122219974

cis_mRNA dw20k	NA	NONHSAT031462.2	NM_001080825	chr12	122235738	122241674	chr12	122150658	122219974
cis_mRNA up10k	NA	NONHSAT031462.2	NM_019034	chr12	122235738	122241674	chr12	122215660	122231594
cis_mRNA dw20k	NA	NONHSAT031554.2	NM_001304833	chr12	123465263	123467461	chr12	123459750	123464588
cis_mRNA overlap	Lnc-Overlap-mRNA	NONHSAT031554.2	NM_001002251	chr12	123465263	123467461	chr12	123464607	123467460
cis_mRNA overlap	Lnc-Overlap-mRNA	NONHSAT031554.2	NM_001278380	chr12	123465263	123467461	chr12	123464607	123467460
cis_mRNA dw20k	NA	NONHSAT031708.2	NM_080626	chr12	125514067	125515683	chr12	125478194	125510349
cis_mRNA overlap	Lnc-Overlap-mRNA	NONHSAT031991.2	NM_001300797	chr12	131356617	131360824	chr12	131356539	131362223
tran	NA	NONHSAT032261.	MTCONS_0005133	chr13	19793206	19801573	chr13	20248902	20357159
tran	NA	NONHSAT032261.	MTCONS_0009079	chr13	19793206	19801573	chr19	20946830	20993757
cis_mRNA dw20k	NA	NONHSAT032281.2	MTCONS_00051335	chr13	20241349	20244877	chr13	20248902	20357159
cis_mRNA _overlap	Lnc-AntiOverlap-mRNA	NONHSAT032281.2	NM_017520	chr13	20241349	20244877	chr13	20207788	20247599
cis_mRNA dw20k	NA	NONHSAT032286.2	NM_017520	chr13	20248902	20251990	chr13	20207788	20247599
cis_mRNA overlap	Lnc-CompleteIn-	NONHSAT032378.2	MTCONS_00051415	chr13	21946716	21948651	chr13	21946710	22033509
tran	NA	NONHSAT033104.	MTCONS_0020841	chr13	33084429	33085024	chrX	118722300	118729852
cis_mRNA overlap	Lnc-Overlap-mRNA	NONHSAT033109.2	MTCONS_00051833	chr13	33107682	33109994	chr13	33091030	33113022
cis_mRNA overlap	Lnc-Overlap-mRNA	NONHSAT033109.2	NM_014887	chr13	33107682	33109994	chr13	33091030	33112932
cis_mRNA dw20k	NA	NONHSAT033213.2	NM_000538	chr13	37417670	37419983	chr13	37393339	37403740
cis_mRNA overlap	Lnc-Overlap-mRNA	NONHSAT033912.2	MTCONS_00049752	chr13	52027277	52030240	chr13	52027277	52037666

cis_mRNA up10k	NA	NONHSAT033912.2	MTCONS_00052513	chr13	52027277	52030240	chr13	51922771	52027275
cis_mRNA up10k	NA	NONHSAT033912.2	NM_012141	chr13	52027277	52030240	chr13	51935701	52027275
cis_mRNA overlap	Lnc-Overlap-mRNA	NONHSAT033913.2	MTCONS_00049752	chr13	52027477	52028966	chr13	52027277	52037666
cis_mRNA overlap	Lnc-CompleteIn-	NONHSAT034093.2	NM_001258367	chr13	60411809	60413392	chr13	60239723	60738119
cis_mRNA up10k	NA	NONHSAT034902.2	NM_177967	chr13	99834096	99852978	chr13	99853163	100038753
cis_mRNA dw20k	NA	NONHSAT035213.2	MTCONS_00051066	chr13	111944867	111947538	chr13	111806006	111938268
cis_mRNA overlap	Lnc-CompleteIn-	NONHSAT035586.2	NM_000270	chr14	20944648	20945246	chr14	20937538	20946165
tran	NA	NONHSAT035616.	NM_002934	chr14	21387500	21388335	chr14	21423630	21424594
cis_mRNA overlap	Lnc-CompleteIn-	NONHSAT035793.2	MTCONS_00054650	chr14	23007853	23008077	chr14	22997994	23021103
cis_mRNA dw20k	NA	NONHSAT035875.2	NM_001282322	chr14	23763061	23764935	chr14	23707127	23743494
cis_mRNA overlap	Lnc-CompleteIn-	NONHSAT036628.2	MTCONS_00058519	chr14	45407755	45409773	chr14	45406840	45431179
cis_mRNA up10k	NA	NONHSAT036748.2	NM_004713	chr14	50320336	50320632	chr14	50250532	50319791
cis_mRNA up10k	NA	NONHSAT036752.2	NM_004713	chr14	50329254	50329627	chr14	50250532	50319791
cis_mRNA dw20k	NA	NONHSAT036882.2	NM_000953	chr14	52753177	52754692	chr14	52734431	52743442
cis_mRNA overlap	Lnc-Overlap-mRNA	NONHSAT036882.2	MTCONS_00055521	chr14	52753177	52754692	chr14	52734431	52795322
tran	NA	NONHSAT037079.	MTCONS_0009079	chr14	57533726	57579378	chr19	20946830	20993757
cis_mRNA overlap	Lnc-CompleteIn-	NONHSAT037151.2	NM_001244193	chr14	58987941	58988706	chr14	58906399	59003931
cis_mRNA overlap	Lnc-Overlap-mRNA	NONHSAT037278.2	MTCONS_00055855	chr14	62193139	62194367	chr14	62162119	62214977

cis_mRNA overlap	Lnc-CompleteIn-	NONHSAT037360.2	NM_002028	chr14	65453948	65455990	chr14	65453507	65529370
cis_mRNA _overlap	Lnc-AntiOverlap-mRNA	NONHSAT037537.2	MTCONS_00056071	chr14	70233000	70234430	chr14	70233804	70238722
cis_mRNA overlap	Lnc-Overlap-mRNA	NONHSAT037539.2	MTCONS_00056071	chr14	70233834	70238721	chr14	70233804	70238722
cis_mRNA overlap	Lnc-Overlap-mRNA	NONHSAT037539.2	NM_006925	chr14	70233834	70238721	chr14	70233804	70238722
cis_mRNA _overlap	Lnc-AntiOverlap-mRNA	NONHSAT037719.2	NM_006821	chr14	74034942	74036537	chr14	74035763	74042362
cis_mRNA overlap	Lnc-CompleteIn-	NONHSAT039178.2	MTCONS_00059815	chr14	89756549	89757938	chr14	89622516	90085494
cis_mRNA up10k	NA	NONHSAT039495.2	NM_001098725	chr14	96181817	96223993	chr14	96176304	96180533
cis_mRNA up10k	NA	NONHSAT039495.2	NM_021966	chr14	96181817	96223993	chr14	96176304	96180533
cis_mRNA dw20k	NA	NONHSAT039622.2	MTCONS_00057106	chr14	99978838	99979913	chr14	99947739	99977852
cis_mRNA overlap	Lnc-Overlap-mRNA	NONHSAT039659.2	MTCONS_00057133	chr14	100608256	100610572	chr14	100531751	100610573
cis_mRNA overlap	Lnc-CompleteIn-	NONHSAT039689.2	NM_001039355	chr14	100770007	100771198	chr14	100757448	100772884
cis_mRNA overlap	Lnc-CompleteIn-	NONHSAT039689.2	NM_001291813	chr14	100770007	100771198	chr14	100757448	100772884
cis_mRNA overlap	Lnc-CompleteIn-	NONHSAT039689.2	NM_152333	chr14	100770007	100771198	chr14	100757448	100772884
cis_mRNA dw20k	NA	NONHSAT040074.2	MTCONS_00060307	chr14	102543520	102543810	chr14	102547075	102553222
cis_mRNA overlap	Lnc-Overlap-mRNA	NONHSAT040076.2	MTCONS_00060307	chr14	102551317	102552044	chr14	102547075	102553222
cis_mRNA dw20k	NA	NONHSAT040138.2	MTCONS_00057380	chr14	103605080	103606351	chr14	103593698	103603776

cis_mRNA dw20k	NA	NONHSAT040197.2	MTCONS_00060377	chr14	104160897	104161507	chr14	104161517	104179894
cis_mRNA dw20k	NA	NONHSAT040197.2	NM_005552	chr14	104160897	104161507	chr14	104095525	104152568
cis_mRNA overlap	Lnc-CompleteIn-	NONHSAT040197.2	NM_001130107	chr14	104160897	104161507	chr14	104095525	104167888
cis_mRNA dw20k	NA	NONHSAT040199.2	NM_005552	chr14	104162690	104163500	chr14	104095525	104152568
cis_mRNA up10k	NA	NONHSAT040317.2	NM_013345	chr14	105532038	105540262	chr14	105515726	105531887
cis_mRNA _overlap	mRNA-CompleteIn-LncIntron	NONHSAT040426.2	MTCONS_00060555	chr14	106330426	107218968	chr14	106493944	106494780
cis_mRNA _overlap	mRNA-CompleteIn-LncIntron	NONHSAT040426.2	MTCONS_00060559	chr14	106330426	107218968	chr14	106551751	106553042
cis_mRNA _overlap	mRNA-CompleteIn-LncIntron	NONHSAT040426.2	MTCONS_00060561	chr14	106330426	107218968	chr14	106573192	106573951
tran	NA	NONHSAT040639.	MTCONS_0006480	chr15	20667631	20711424	chr15	23281071	23378259
cis_mRNA overlap	Lnc-Overlap-mRNA	NONHSAT040801.2	MTCONS_00064788	chr15	23190859	23208357	chr15	23181307	23208357
tran	NA	NONHSAT041129.	MTCONS_0009079	chr15	25500726	25502174	chr19	20946830	20993757
cis_mRNA overlap	Lnc-Overlap-mRNA	NONHSAT041265.2	MTCONS_00061714	chr15	28986271	29000413	chr15	28982558	29005800
tran	NA	NONHSAT041265.	NM_001080435	chr15	28986271	29000413	chr15	83478380	83503613
cis_mRNA overlap	Lnc-CompleteIn-	NONHSAT041417.2	NM_014967	chr15	31218717	31220610	chr15	31196076	31235310
cis_mRNA dw20k	NA	NONHSAT041492.2	NM_001277308	chr15	32702782	32727193	chr15	32734115	32747835
cis_mRNA overlap	Lnc-Overlap-mRNA	NONHSAT041492.2	MTCONS_00065040	chr15	32702782	32727193	chr15	32681724	32727434
cis_mRNA up10k	NA	NONHSAT041777.2	NM_001013703	chr15	40213271	40218081	chr15	40226325	40327797

cis_mRNA dw20k	NA	NONHSAT041787.2	NM_001013703	chr15	40328505	40331296	chr15	40226325	40327797
cis_mRNA up10k	NA	NONHSAT041802.2	NM_001309434	chr15	40338162	40358063	chr15	40327891	40331403
tran	NA	NONHSAT041924.	MTCONS_0017304	chr15	41576206	41580469	chr6	150117711	150185480
cis_mRNA overlap	Lnc-CompleteIn-	NONHSAT041962.2	MTCONS_00062284	chr15	42100589	42102741	chr15	42065307	42120059
tran	NA	NONHSAT042222.	MTCONS_0003070	chr15	45444862	45459724	chr11	67155110	67165883
cis_mRNA dw20k	NA	NONHSAT042255.2	NM_032413	chr15	45742448	45771942	chr15	45722727	45725647
cis_mRNA up10k	NA	NONHSAT042443.2	NM_007347	chr15	51199113	51200792	chr15	51200869	51298097
cis_mRNA up10k	NA	NONHSAT043921.2	NM_006578	chr15	52472223	52498076	chr15	52413123	52472162
cis_mRNA up10k	NA	NONHSAT043922.2	NM_006578	chr15	52472414	52498071	chr15	52413123	52472162
cis_mRNA dw20k	NA	NONHSAT044134.2	MTCONS_00065990	chr15	59152982	59154096	chr15	59171244	59225875
cis_mRNA overlap	Lnc-CompleteIn-	NONHSAT044188.2	MTCONS_00066014	chr15	59955064	59961189	chr15	59951345	59981733
cis_mRNA up10k	NA	NONHSAT044188.2	NM_004492	chr15	59955064	59961189	chr15	59930261	59949737
cis_mRNA dw20k	NA	NONHSAT046604.2	NM_031301	chr15	63610925	63618606	chr15	63569749	63601325
cis_mRNA dw20k	NA	NONHSAT046951.2	MTCONS_00063204	chr15	69750861	69754145	chr15	69706585	69740766
cis_mRNA _overlap	Lnc-AntiOverlap-mRNA	NONHSAT047181.2	NM_005576	chr15	74209809	74220301	chr15	74218799	74244482
cis_mRNA _overlap	Lnc-AntiOverlap-mRNA	NONHSAT047342.2	NM_015477	chr15	75660496	75661925	chr15	75661720	75743926
tran	NA	NONHSAT047368.	NM_021130	chr15	76039110	76039604	chr7	44836235	44842722
cis_mRNA overlap	Lnc-Overlap-mRNA	NONHSAT047781.2	NM_001291420	chr15	83087381	83102960	chr15	83099513	83108111

cis_mRNA dw20k	NA	NONHSAT047870.2	MTCONS_00063822	chr15	83511246	83514392	chr15	83478380	83503613
tran	NA	NONHSAT047967.	NM_001291420	chr15	85047738	85060078	chr15	83099513	83108111
cis_mRNA dw20k	NA	NONHSAT047990.2	NM_181877	chr15	85173314	85174690	chr15	85144249	85166947
cis_mRNA_overlap	Lnc-AntiOverlap-mRNA	NONHSAT047990.2	MTCONS_00063894	chr15	85173314	85174690	chr15	85174682	85186244
cis_mRNA_overlap	Lnc-AntiOverlap-mRNA	NONHSAT047999.2	MTCONS_00063894	chr15	85177461	85188902	chr15	85174682	85186244
cis_mRNA overlap	Lnc-Overlap-mRNA	NONHSAT048002.2	MTCONS_00063894	chr15	85177512	85182793	chr15	85174682	85186244
cis_mRNA_overlap	Lnc-AntiOverlap-mRNA	NONHSAT048193.2	MTCONS_00064010	chr15	89181898	89182861	chr15	89178868	89199575
cis_mRNA_overlap	Lnc-AntiOverlap-mRNA	NONHSAT048193.2	NM_001303236	chr15	89181898	89182861	chr15	89181974	89199575
tran	NA	NONHSAT048463.	MTCONS_0003070	chr15	91565849	91573936	chr11	67155110	67165883
cis_mRNA up10k	NA	NONHSAT049679.2	NM_001271	chr15	93430986	93441302	chr15	93443551	93571237
tran	NA	NONHSAT050728.	MTCONS_0009079	chr15	97839111	97959054	chr19	20946830	20993757
tran	NA	NONHSAT051129.	MTCONS_0016852	chr15	101389988	101396267	chr6	2887500	2903546
tran	NA	NONHSAT051182.	MTCONS_0006354	chr15	101874642	101877633	chr15	76196200	76229582
cis_mRNA_overlap	Lnc-AntiOverlap-mRNA	NONHSAT051184.2	NM_138319	chr15	102030123	102031685	chr15	101844133	102030187
cis_mRNA up10k	NA	NONHSAT051185.2	NM_138319	chr15	102031215	102032412	chr15	101844133	102030187
cis_mRNA up10k	NA	NONHSAT051185.2	NM_138323	chr15	102031215	102032412	chr15	101886947	102030187
tran	NA	NONHSAT051726.	MTCONS_0000889	chr16	64088	68422	chr1	14407	29370
cis_mRNA up10k	NA	NONHSAT051779.2	MTCONS_00068273	chr16	572483	577855	chr16	577856	604636

cis_mRNA up10k	NA	NONHSAT051816.2	MTCONS_00071952	chr16	695208	699002	chr16	684427	686366
cis_mRNA dw20k	NA	NONHSAT051856.2	NM_001031737	chr16	767619	769654	chr16	772582	776473
cis_mRNA up10k	NA	NONHSAT051899.2	NM_014587	chr16	1025761	1031590	chr16	1031808	1036979
cis_mRNA up10k	NA	NONHSAT051902.2	NM_014587	chr16	1025761	1031318	chr16	1031808	1036979
cis_mRNA up10k	NA	NONHSAT051904.2	NM_014587	chr16	1025780	1030677	chr16	1031808	1036979
cis_mRNA dw20k	NA	NONHSAT051908.2	NM_014587	chr16	1041085	1043057	chr16	1031808	1036979
cis_mRNA overlap	Lnc-Overlap-mRNA	NONHSAT052328.2	NM_014238	chr17	25950561	25953461	chr17	25799036	25950718
cis_mRNA overlap	Lnc-Overlap-mRNA	NONHSAT052329.2	NM_014238	chr17	25950561	25953461	chr17	25799036	25950718
cis_mRNA dw20k	NA	NONHSAT052672.2	MTCONS_00081349	chr17	27253293	27271767	chr17	27281947	27333458
cis_mRNA up10k	NA	NONHSAT052678.2	NM_001033561	chr17	27280048	27281946	chr17	27232271	27278508
cis_mRNA overlap	Lnc-CompleteIn-	NONHSAT052728.2	MTCONS_00081370	chr17	27971814	27972865	chr17	27951695	28088450
cis_mRNA up10k	NA	NONHSAT052840.2	NM_024683	chr17	29234392	29237871	chr17	29226001	29233286
cis_mRNA dw20k	NA	NONHSAT053044.2	NM_013975	chr17	33332107	33336762	chr17	33307517	33332088
cis_mRNA_overlap	Lnc-AntiOverlap-mRNA	NONHSAT053044.2	NM_001017368	chr17	33332107	33336762	chr17	33336131	33390759
cis_mRNA overlap	Lnc-Overlap-mRNA	NONHSAT053093.2	MTCONS_00081573	chr17	33733967	33734837	chr17	33733703	33760195
cis_mRNA dw20k	NA	NONHSAT053099.2	NM_001195790	chr17	33797088	33797647	chr17	33801942	33814758
cis_mRNA overlap	Lnc-Overlap-mRNA	NONHSAT053196.2	MTCONS_00081678	chr17	34623842	34625720	chr17	34522269	34625731

tran	NA	NONHSAT053196.	NM_002983	chr17	34623842	34625720	chr17	34415602	34417506
tran	NA	NONHSAT053197.	NM_001291469	chr17	34640056	34641835	chr17	34431220	34433014
tran	NA	NONHSAT053197.	NM_001291470	chr17	34640056	34641835	chr17	34431220	34433014
tran	NA	NONHSAT053197.	NM_001291474	chr17	34640056	34641835	chr17	34431220	34433014
tran	NA	NONHSAT053207.	NM_001123391	chr17	34803416	34805455	chr17	36284005	36295098
tran	NA	NONHSAT053207.	MTCONS_0008177	chr17	34803416	34805455	chr17	36340447	36413315
cis_mRNA up10k	NA	NONHSAT053517. 2	MTCONS_0007750 8	chr17	38275466	38277989	chr17	38278790	38293044
cis_mRNA up10k	NA	NONHSAT053517. 2	NM_001012241	chr17	38275466	38277989	chr17	38278790	38293044
cis_mRNA _overlap	Lnc-AntiOverlap-mRNA	NONHSAT053726. 2	NM_000413	chr17	40704456	40706766	chr17	40703984	40707232
cis_mRNA dw20k	NA	NONHSAT053905. 2	MTCONS_0008229 2	chr17	42253342	42264085	chr17	42269173	42278853
cis_mRNA dw20k	NA	NONHSAT053905. 2	NM_001098833	chr17	42253342	42264085	chr17	42269173	42275529
cis_mRNA dw20k	NA	NONHSAT053962. 2	NM_144609	chr17	42751056	42753625	chr17	42754805	42767165
cis_mRNA _overlap	Lnc-AntiOverlap-mRNA	NONHSAT054035. 2	MTCONS_0007785 3	chr17	43296717	43299589	chr17	43299292	43324686
tran	NA	NONHSAT054036.	MTCONS_0003070	chr17	43297102	43297699	chr11	67155110	67165883
cis_mRNA overlap	Lnc-Overlap-mRNA	NONHSAT054039. 2	MTCONS_0007785 4	chr17	43299292	43324681	chr17	43299292	43324686
cis_mRNA dw20k	NA	NONHSAT054213. 2	MTCONS_0007795 5	chr17	44636196	44640161	chr17	44450164	44633014
cis_mRNA dw20k	NA	NONHSAT054213. 2	NM_001006607	chr17	44636196	44640161	chr17	44590076	44633014
cis_mRNA dw20k	NA	NONHSAT054716. 2	MTCONS_0007830 4	chr17	48830417	48833574	chr17	48796926	48830072
cis_mRNA dw20k	NA	NONHSAT054716. 2	NM_016424	chr17	48830417	48833574	chr17	48796926	48830072
cis_mRNA overlap	Lnc-CompleteIn-	NONHSAT054895. 2	NM_138962	chr17	55731047	55732651	chr17	55333931	55757299

cis_mRNA dw20k	NA	NONHSAT054916.2	NM_001078166	chr17	56066405	56072211	chr17	56078280	56084707
cis_mRNA up10k	NA	NONHSAT054952.2	MTCONS_00082934	chr17	56414563	56431088	chr17	56378588	56406152
cis_mRNA dw20k	NA	NONHSAT055218.2	NM_001003788	chr17	61777698	61780045	chr17	61780192	61819330
cis_mRNA up10k	NA	NONHSAT055218.2	MTCONS_00083142	chr17	61777698	61780045	chr17	61773249	61777519
cis_mRNA dw20k	NA	NONHSAT055267.2	NM_001433	chr17	62116510	62119203	chr17	62120390	62207502
cis_mRNA overlap	Lnc-Overlap-mRNA	NONHSAT055313.2	MTCONS_00083237	chr17	62774935	62777801	chr17	62745641	62811106
cis_mRNA overlap	Lnc-Overlap-mRNA	NONHSAT055313.2	MTCONS_00083246	chr17	62774935	62777801	chr17	62771867	62793418
cis_mRNA overlap	Lnc-Overlap-mRNA	NONHSAT055313.2	MTCONS_00083248	chr17	62774935	62777801	chr17	62771867	62811106
tran	NA	NONHSAT055523.	MTCONS_0017572	chr17	66203737	66209123	chr7	4815262	4834026
cis_mRNA dw20k	NA	NONHSAT055585.2	NM_002758	chr17	67547499	67550002	chr17	67410838	67538470
cis_mRNA up10k	NA	NONHSAT055616.2	NM_000891	chr17	68163102	68165543	chr17	68165676	68176183
cis_mRNA dw20k	NA	NONHSAT055859.2	NM_024585	chr17	73126978	73129138	chr17	73106047	73126360
cis_mRNA overlap	Lnc-CompleteIn-	NONHSAT055909.2	NM_001031803	chr17	73561174	73563545	chr17	73521783	73571290
cis_mRNA overlap	Lnc-CompleteIn-	NONHSAT055909.2	NM_004524	chr17	73561174	73563545	chr17	73521783	73571290
cis_mRNA overlap	Lnc-Overlap-mRNA	NONHSAT055916.2	MTCONS_00079197	chr17	73598382	73599142	chr17	73583535	73622960
cis_mRNA up10k	NA	NONHSAT056017.2	NM_001005498	chr17	74492862	74494931	chr17	74466975	74483991
cis_mRNA dw20k	NA	NONHSAT056077.2	NM_001206983	chr17	74730205	74732546	chr17	74722912	74729963
cis_mRNA overlap	Lnc-Overlap-mRNA	NONHSAT056077.2	MTCONS_00083847	chr17	74730205	74732546	chr17	74730197	74733493

cis_mRNA overlap	Lnc-Overlap-mRNA	NONHSAT056077.2	MTCONS_00083848	chr17	74730205	74732546	chr17	74730197	74733493
cis_mRNA up10k	NA	NONHSAT056077.2	NM_001242533	chr17	74730205	74732546	chr17	74733583	74775336
cis_mRNA up10k	NA	NONHSAT056153.2	NM_001113495	chr17	75465150	75467127	chr17	75471325	75496678
cis_mRNA overlap	Lnc-Overlap-mRNA	NONHSAT056177.2	MTCONS_00079447	chr17	75975719	75978584	chr17	75973265	76104916
cis_mRNA overlap	Lnc-Overlap-mRNA	NONHSAT056184.2	MTCONS_00083912	chr17	76103479	76106416	chr17	76103479	76124366
cis_mRNA overlap	Lnc-Overlap-mRNA	NONHSAT056184.2	MTCONS_00083913	chr17	76103479	76106416	chr17	76103479	76126834
cis_mRNA overlap	Lnc-Overlap-mRNA	NONHSAT056184.2	MTCONS_00083915	chr17	76103479	76106416	chr17	76103479	76128488
cis_mRNA dw20k	NA	NONHSAT056185.2	NM_007267	chr17	76103479	76107880	chr17	76108999	76128488
cis_mRNA up10k	NA	NONHSAT056248.2	MTCONS_00083952	chr17	76837262	76841644	chr17	76792965	76837260
cis_mRNA overlap	Lnc-Overlap-mRNA	NONHSAT056339.2	NM_001079803	chr17	78086183	78087070	chr17	78075325	78093681
cis_mRNA dw20k	NA	NONHSAT056435.2	NM_001080395	chr17	79084283	79086760	chr17	79091096	79139872
cis_mRNA overlap	Lnc-Overlap-mRNA	NONHSAT056720.2	MTCONS_00079801	chr17	80439974	80441264	chr17	80439974	80446143
cis_mRNA dw20k	NA	NONHSAT056773.2	NM_001004431	chr17	81061205	81063860	chr17	81037567	81052871
cis_mRNA up10k	NA	NONHSAT056834.2	NM_005131	chr18	268148	270278	chr18	214520	268059
cis_mRNA overlap	Lnc-CompleteIn-	NONHSAT056864.2	NM_005433	chr18	738058	739662	chr18	721592	812327
tran	NA	NONHSAT056926.	MTCONS_0015806	chr18	2507852	2510776	chr5	74319296	74348668
tran	NA	NONHSAT056959.	NM_012124	chr18	2943213	2946621	chr11	89933598	89956532
cis_mRNA dw20k	NA	NONHSAT058560.2	NM_005406	chr18	18526867	18528428	chr18	18529703	18691812

cis_mRNA dw20k	NA	NONHSAT058912.2	MTCONS_00085816	chr18	32848504	32850202	chr18	32819995	32831020
cis_mRNA_overlap	Lnc-AntiOverlap-mRNA	NONHSAT058952.2	NM_001201475	chr18	33552114	33553879	chr18	33552588	33559250
cis_mRNA dw20k	NA	NONHSAT058969.2	NM_018255	chr18	33755224	33757890	chr18	33709837	33754689
cis_mRNA dw20k	NA	NONHSAT059059.2	NM_002647	chr18	39661838	39663348	chr18	39535163	39661446
cis_mRNA up10k	NA	NONHSAT059142.2	NM_001307987	chr18	44339687	44344371	chr18	44259081	44337132
cis_mRNA dw20k	NA	NONHSAT059144.2	MTCONS_00088010	chr18	44383187	44383857	chr18	44390023	44485531
cis_mRNA dw20k	NA	NONHSAT059173.2	NM_001003652	chr18	45346666	45356490	chr18	45359466	45457517
tran	NA	NONHSAT059374.	MTCONS_0009079	chr18	53388294	53426353	chr19	20946830	20993757
tran	NA	NONHSAT060114.	MTCONS_0003295	chr19	197016	202209	chr11	126987	139612
cis_mRNA_overlap	Lnc-AntiOverlap-mRNA	NONHSAT060209.2	NM_138690	chr19	999795	1002756	chr19	1000418	1009731
cis_mRNA dw20k	NA	NONHSAT060210.2	NM_001033026	chr19	999795	1002756	chr19	1009650	1021141
tran	NA	NONHSAT060210.	MTCONS_0004045	chr19	999795	1002756	chr12	56915609	56989980
cis_mRNA overlap	Lnc-Overlap-mRNA	NONHSAT060255.2	NM_001300829	chr19	1271166	1272056	chr19	1269265	1273171
cis_mRNA dw20k	NA	NONHSAT060307.2	NM_152482	chr19	1457664	1458590	chr19	1473200	1479228
cis_mRNA dw20k	NA	NONHSAT060307.2	NM_001308226	chr19	1457664	1458590	chr19	1438424	1440496
tran	NA	NONHSAT060550.	MTCONS_0006648	chr19	3753838	3756515	chr15	72491337	72526033
tran	NA	NONHSAT060550.	MTCONS_0017304	chr19	3753838	3756515	chr6	150117711	150185480
cis_mRNA overlap	Lnc-Overlap-mRNA	NONHSAT060780.2	NM_003807	chr19	6661264	6663469	chr19	6663148	6670599
cis_mRNA dw20k	NA	NONHSAT060790.2	NM_005490	chr19	6748304	6751478	chr19	6752173	6767523

tran	NA	NONHSAT060870.	MTCONS_0011279	chr19	8073613	8080909	chr2	208470297	208490028
cis_mRNA dw20k	NA	NONHSAT060973. 2	NM_001008727	chr19	9671029	9673435	chr19	9676404	9695209
tran	NA	NONHSAT060996.	MTCONS_0009682	chr19	9894275	9895950	chr19	52370214	52408305
cis_mRNA dw20k	NA	NONHSAT061012. 2	NM_001304348	chr19	9946122	9960357	chr19	9964394	9968844
cis_mRNA _overlap	Lnc- AntiOverlap- mRNA	NONHSAT062781. 2	NM_016270	chr19	16435645	16438303	chr19	16435651	16438339
cis_mRNA _overlap	Lnc- AntiOverlap- mRNA	NONHSAT063012. 2	MTCONS_0009489 3	chr19	19101702	19104419	chr19	19101697	19144669
cis_mRNA _overlap	Lnc- AntiOverlap- mRNA	NONHSAT063012. 2	NM_001017392	chr19	19101702	19104419	chr19	19103177	19144380
cis_mRNA dw20k	NA	NONHSAT063580. 2	NM_001145404	chr19	19867183	19887222	chr19	19903520	19932560
tran	NA	NONHSAT063615.	NM_001130022	chr19	20011787	20043805	chr7	63985034	64023505
tran	NA	NONHSAT063635.	MTCONS_0014000	chr19	20286909	20349269	chr3	197328847	197354756
cis_mRNA overlap	Lnc- CompleteIn-	NONHSAT063690. 2	MTCONS_0009081 5	chr19	21371401	21371756	chr19	21324840	21373267
tran	NA	NONHSAT064044.	MTCONS_0007393	chr19	29000145	29009635	chr16	67263292	67281425
cis_mRNA up10k	NA	NONHSAT064104. 2	NM_001146339	chr19	30016347	30017286	chr19	30017491	30055226
cis_mRNA overlap	Lnc- CompleteIn-	NONHSAT064200. 2	NM_001172774	chr19	32962847	32967434	chr19	32896655	32976799
cis_mRNA overlap	Lnc- CompleteIn-	NONHSAT064226. 2	MTCONS_0009539 5	chr19	33440310	33441930	chr19	33366831	33462935
tran	NA	NONHSAT064308.	MTCONS_0009079	chr19	35113839	35117228	chr19	20946830	20993757
cis_mRNA _overlap	Lnc- AntiOverlap- mRNA	NONHSAT064321. 2	MTCONS_0009127 9	chr19	35224208	35225092	chr19	35225091	35233774
cis_mRNA overlap	Lnc-Overlap- mRNA	NONHSAT065861. 2	NM_001126056	chr19	35994207	36001412	chr19	35988119	36004560

cis_mRNA dw20k	NA	NONHSAT065931.2	NM_139239	chr19	36370792	36376739	chr19	36379143	36391552
cis_mRNA up10k	NA	NONHSAT066066.2	MTCONS_00091597	chr19	37567724	37569336	chr19	37569382	37622419
cis_mRNA overlap	Lnc-Overlap-mRNA	NONHSAT066139.2	NM_152606	chr19	38042273	38078249	chr19	38042273	38105079
cis_mRNA dw20k	NA	NONHSAT066205.2	NM_004823	chr19	38822274	38822716	chr19	38810484	38819649
cis_mRNA up10k	NA	NONHSAT066331.2	MTCONS_00091813	chr19	40497983	40502942	chr19	40502943	40528186
cis_mRNA overlap	Lnc-CompleteIn-	NONHSAT066361.2	NM_001626	chr19	40779396	40781386	chr19	40736224	40791302
cis_mRNA dw20k	NA	NONHSAT066476.2	MTCONS_00091935	chr19	42108598	42112339	chr19	42082270	42093220
cis_mRNA _overlap	Lnc-AntiOverlap-mRNA	NONHSAT066552.2	NM_001184813	chr19	42928421	43030021	chr19	43011458	43032661
cis_mRNA dw20k	NA	NONHSAT066614.2	NM_001145347	chr19	44124016	44126521	chr19	44100738	44104587
cis_mRNA dw20k	NA	NONHSAT066662.2	MTCONS_00092071	chr19	44609492	44617336	chr19	44580170	44593933
cis_mRNA dw20k	NA	NONHSAT066721.2	MTCONS_00092129	chr19	45265341	45265782	chr19	45252565	45264504
cis_mRNA dw20k	NA	NONHSAT066721.2	MTCONS_00092130	chr19	45265341	45265782	chr19	45252565	45264504
cis_mRNA _overlap	Lnc-AntiOverlap-mRNA	NONHSAT066811.2	NM_001081563	chr19	46271645	46273064	chr19	46272967	46283861
cis_mRNA _overlap	Lnc-AntiOverlap-mRNA	NONHSAT066812.2	NM_001288765	chr19	46272072	46273449	chr19	46272967	46282877
cis_mRNA _overlap	Lnc-AntiOverlap-mRNA	NONHSAT067264.2	NM_001305105	chr19	50358224	50362810	chr19	50354377	50364001

cis_mRNA overlap	Lnc-Overlap-mRNA	NONHSAT067294.2	MTCONS_00092599	chr19	50472912	50479075	chr19	50472482	50475577
tran	NA	NONHSAT067319.	MTCONS_0009665	chr19	50837073	50837861	chr19	50860029	50872584
cis_mRNA dw20k	NA	NONHSAT067336.2	NM_001270639	chr19	50988164	50990120	chr19	51009254	51014612
cis_mRNA dw20k	NA	NONHSAT067435.2	NM_152353	chr19	51864966	51868798	chr19	51870352	51872257
cis_mRNA overlap	Lnc-Overlap-mRNA	NONHSAT067646.2	MTCONS_00092872	chr19	53700364	53714268	chr19	53700364	53719355
tran	NA	NONHSAT067889.	MTCONS_0009437	chr19	54753289	54753907	chr19	11487649	11495018
tran	NA	NONHSAT067889.	MTCONS_0012760	chr19	54753289	54753907	chr22	31835345	31889009
cis_mRNA overlap	Lnc-CompleteIn-	NONHSAT067901.2	MTCONS_00097085	chr19	54862991	54864894	chr19	54739078	55142756
cis_mRNA up10k	NA	NONHSAT067917.2	MTCONS_00093012	chr19	54948912	54960064	chr19	54960340	54973226
cis_mRNA up10k	NA	NONHSAT067917.2	NM_052925	chr19	54948912	54960064	chr19	54960340	54973226
cis_mRNA dw20k	NA	NONHSAT068218.2	NM_006635	chr19	57808920	57811732	chr19	57791853	57805436
cis_mRNA dw20k	NA	NONHSAT068223.2	NM_006635	chr19	57815673	57819930	chr19	57791853	57805436
tran	NA	NONHSAT068268.	NM_001290318	chr19	58212908	58214160	chr19	57862642	57871265
cis_mRNA up10k	NA	NONHSAT068318.2	NM_024620	chr19	58664228	58666447	chr19	58637695	58662148
cis_mRNA_overlap	Lnc-AntiOverlap-mRNA	NONHSAT068374.2	NM_130786	chr19	58863336	58866549	chr19	58858172	58864865
cis_mRNA dw20k	NA	NONHSAT068377.2	NM_181846	chr19	58869365	58870969	chr19	58838385	58853712
cis_mRNA dw20k	NA	NONHSAT068377.2	MTCONS_00097434	chr19	58869365	58870969	chr19	58878990	58892389
cis_mRNA up10k	NA	NONHSAT068377.2	NM_130786	chr19	58869365	58870969	chr19	58858172	58864865
cis_mRNA dw20k	NA	NONHSAT068403.2	MTCONS_00093518	chr19	58939999	58942515	chr19	58920041	58929692

cis_mRNA dw20k	NA	NONHSAT068403.2	NM_003433	chr19	58939999	58942515	chr19	58944181	58951589
tran	NA	NONHSAT068742.	NM_001127395	chr2	3616113	3622828	chr2	208473839	208490055
cis_mRNA dw20k	NA	NONHSAT068916.2	NM_001256478	chr2	6960200	6980496	chr2	6980684	7005950
cis_mRNA dw20k	NA	NONHSAT069046.2	MTCONS_00106760	chr2	9613229	9614664	chr2	9628615	9695949
cis_mRNA overlap	Lnc-CompleteIn-	NONHSAT069179.2	NM_014668	chr2	11724899	11725176	chr2	11674242	11782912
cis_mRNA dw20k	NA	NONHSAT069465.2	NM_182828	chr2	20877569	20879005	chr2	20866424	20871250
cis_mRNA overlap	Lnc-Overlap-mRNA	NONHSAT069584.2	MTCONS_00107195	chr2	24516130	24518580	chr2	24461805	24583397
cis_mRNA up10k	NA	NONHSAT069792.2	NM_004891	chr2	27928653	27989051	chr2	27994584	28002608
cis_mRNA up10k	NA	NONHSAT069792.2	NM_145330	chr2	27928653	27989051	chr2	27994584	28002608
cis_mRNA dw20k	NA	NONHSAT069972.2	NM_032312	chr2	32534100	32538803	chr2	32502958	32531658
cis_mRNA dw20k	NA	NONHSAT070189.2	MTCONS_00107662	chr2	38787215	38789656	chr2	38790328	38830178
cis_mRNA overlap	Lnc-CompleteIn-	NONHSAT070218.2	MTCONS_00107696	chr2	39242995	39244034	chr2	39208690	39348983
tran	NA	NONHSAT070320.	MTCONS_0001411	chr2	42487062	42487735	chr1	156440956	156470634
cis_mRNA overlap	Lnc-Overlap-mRNA	NONHSAT070518.2	MTCONS_00099582	chr2	46865336	46897728	chr2	46844311	46886111
tran	NA	NONHSAT070692.	MTCONS_0009079	chr2	53889616	53891963	chr19	20946830	20993757
cis_mRNA overlap	Lnc-Overlap-mRNA	NONHSAT070708.2	MTCONS_00108089	chr2	54159193	54160306	chr2	54102550	54197977
cis_mRNA _overlap	Lnc-AntiOverlap-mRNA	NONHSAT070777.2	NM_001254943	chr2	55535628	55541797	chr2	55514978	55647057
tran	NA	NONHSAT070821.	MTCONS_0011100	chr2	56400669	56412905	chr2	148687966	148778463
cis_mRNA overlap	Lnc-Overlap-mRNA	NONHSAT070917.2	MTCONS_00099968	chr2	61159963	61163467	chr2	61108630	61167156

cis_mRNA_overlap	Lnc-AntiOverlap-mRNA	NONHSAT070941.2	NM_014709	chr2	61404584	61416060	chr2	61414590	61697849
cis_mRNA_overlap	Lnc-Overlap-mRNA	NONHSAT070941.2	NM_152392	chr2	61404584	61416060	chr2	61404555	61414686
cis_mRNA_overlap	Lnc-AntiOverlap-mRNA	NONHSAT070970.2	NM_003400	chr2	61710508	61711076	chr2	61705069	61765418
cis_mRNA_overlap	Lnc-CompleteIn-	NONHSAT071060.2	NM_015910	chr2	63779650	63781836	chr2	63348535	63815867
tran	NA	NONHSAT071221.	MTCONS_0009079	chr2	66610438	66619582	chr19	20946830	20993757
cis_mRNA_overlap	Lnc-Overlap-mRNA	NONHSAT071372.2	NM_152792	chr2	70189395	70314147	chr2	70187223	70189397
cis_mRNA_up10k	NA	NONHSAT071649.2	NM_001287491	chr2	74212259	74213470	chr2	74213531	74335302
cis_mRNA_overlap	Lnc-AntiOverlap-mRNA	NONHSAT072113.2	NM_001193517	chr2	86789198	86844564	chr2	86730553	86790620
cis_mRNA_overlap	Lnc-AntiOverlap-mRNA	NONHSAT072201.2	MTCONS_00109344	chr2	88927057	88931337	chr2	88856258	88927094
tran	NA	NONHSAT072238.	MTCONS_0010092	chr2	89160084	89545062	chr2	89975678	89976487
cis_mRNA_overlap	Lnc-Overlap-mRNA	NONHSAT072240.2	MTCONS_00109360	chr2	89160375	89442344	chr2	89156608	89340219
tran	NA	NONHSAT072299.	MTCONS_0010093	chr2	89533655	89534393	chr2	89986240	89987074
tran	NA	NONHSAT072508.	MTCONS_0010959	chr2	96455402	96463594	chr2	96687694	96700727
cis_mRNA_dw20k	NA	NONHSAT072590.2	NM_020184	chr2	97493065	97500048	chr2	97426639	97477628
cis_mRNA_overlap	Lnc-Overlap-mRNA	NONHSAT072590.2	MTCONS_00109637	chr2	97493065	97500048	chr2	97492718	97523756
cis_mRNA_up10k	NA	NONHSAT072592.2	NM_017789	chr2	97535853	97539075	chr2	97525473	97535735
cis_mRNA_overlap	Lnc-CompleteIn-	NONHSAT072704.2	NM_001134225	chr2	99089196	99090054	chr2	99061321	99207496

cis_mRNA dw20k	NA	NONHSAT072743.2	NM_145199	chr2	99797542	99816020	chr2	99771418	99779613
cis_mRNA overlap	Lnc-CompleteIn-	NONHSAT072819.2	NM_017546	chr2	101885764	101886759	chr2	101869345	101886778
cis_mRNA dw20k	NA	NONHSAT072844.2	MTCONS_00101390	chr2	102661007	102677543	chr2	102607381	102644884
cis_mRNA dw20k	NA	NONHSAT072844.2	NM_004633	chr2	102661007	102677543	chr2	102608306	102644884
cis_mRNA _overlap	Lnc-AntiOverlap-mRNA	NONHSAT072984.2	MTCONS_00101522	chr2	105950230	105953903	chr2	105953816	105967842
cis_mRNA _overlap	Lnc-AntiOverlap-mRNA	NONHSAT072984.2	NM_024093	chr2	105950230	105953903	chr2	105953816	105965271
cis_mRNA up10k	NA	NONHSAT073731.2	MTCONS_00110239	chr2	113597905	113601772	chr2	113587337	113594356
cis_mRNA up10k	NA	NONHSAT073731.2	MTCONS_00110242	chr2	113597905	113601772	chr2	113587337	113594356
cis_mRNA dw20k	NA	NONHSAT073752.2	MTCONS_00101851	chr2	113963503	113966973	chr2	113931560	113960677
cis_mRNA dw20k	NA	NONHSAT073752.2	MTCONS_00101853	chr2	113963503	113966973	chr2	113931560	113960677
cis_mRNA dw20k	NA	NONHSAT073831.2	NM_025181	chr2	114464370	114467816	chr2	114470369	114514400
cis_mRNA dw20k	NA	NONHSAT074141.2	NM_139350	chr2	127782787	127787262	chr2	127805599	127864903
cis_mRNA dw20k	NA	NONHSAT074145.2	MTCONS_00110558	chr2	128004834	128007658	chr2	128014866	128051752
cis_mRNA dw20k	NA	NONHSAT074145.2	MTCONS_00110559	chr2	128004834	128007658	chr2	128014866	128051752
cis_mRNA dw20k	NA	NONHSAT074145.2	NM_001303418	chr2	128004834	128007658	chr2	128014866	128051670
cis_mRNA dw20k	NA	NONHSAT074192.2	NM_001199140	chr2	128601127	128603261	chr2	128619205	128643514
tran	NA	NONHSAT074498.	NM_024718	chr2	132591356	132594105	chr9	139702374	139735639

cis_mRNA_overlap	Lnc-CompleteIn-	NONHSAT074953.2	MTCONS_00103168	chr2	152228517	152228798	chr2	152214106	152236562
cis_mRNA_up10k	NA	NONHSAT075047.2	MTCONS_00111174	chr2	157193084	157198632	chr2	157180944	157190970
cis_mRNA_up10k	NA	NONHSAT075047.2	MTCONS_00111177	chr2	157193084	157198632	chr2	157183980	157189287
cis_mRNA_dw20k	NA	NONHSAT075123.2	NM_022826	chr2	160626484	160629028	chr2	160568968	160625094
cis_mRNA_dw20k	NA	NONHSAT075265.2	NM_024753	chr2	166713986	166728451	chr2	166729872	166810348
cis_mRNA_dw20k	NA	NONHSAT075351.2	NM_152384	chr2	170382967	170383354	chr2	170336006	170363165
cis_mRNA_overlap	Lnc-AntiOverlap-mRNA	NONHSAT075507.2	MTCONS_00103672	chr2	173345568	173421257	chr2	173420697	173480770
cis_mRNA_overlap	Lnc-AntiOverlap-mRNA	NONHSAT075507.2	MTCONS_00103675	chr2	173345568	173421257	chr2	173420697	173486231
cis_mRNA_dw20k	NA	NONHSAT075698.2	NM_001145412	chr2	178072885	178077421	chr2	178095031	178128859
cis_mRNA_overlap	Lnc-AntiOverlap-mRNA	NONHSAT075802.2	NM_133378	chr2	179449300	179456025	chr2	179390718	179672150
cis_mRNA_up10k	NA	NONHSAT075887.2	NM_001287505	chr2	182752578	182756390	chr2	182756443	182759424
cis_mRNA_overlap	mRNA-CompleteIn-LncExon	NONHSAT075914.2	NM_018981	chr2	183580768	183644750	chr2	183580768	183644750
cis_mRNA_overlap	Lnc-Overlap-mRNA	NONHSAT075940.2	MTCONS_00104094	chr2	184043954	184080015	chr2	183989083	184053108
cis_mRNA_overlap	Lnc-AntiOverlap-mRNA	NONHSAT075952.2	NM_194250	chr2	185460944	185463775	chr2	185463093	185804214
cis_mRNA_dw20k	NA	NONHSAT076128.2	MTCONS_00104294	chr2	192556795	192583257	chr2	192542798	192553248

cis_mRNA dw20k	NA	NONHSAT076204.2	MTCONS_00112406	chr2	197851390	197853327	chr2	197855046	198175521
cis_mRNA overlap	Lnc-CompleteIn-	NONHSAT076209.2	MTCONS_00112406	chr2	197961283	197961876	chr2	197855046	198175521
cis_mRNA overlap	Lnc-CompleteIn-	NONHSAT076212.2	MTCONS_00112406	chr2	198115582	198167243	chr2	197855046	198175521
cis_mRNA dw20k	NA	NONHSAT076328.2	NM_001207069	chr2	201689396	201692061	chr2	201676908	201688569
tran	NA	NONHSAT076353.	MTCONS_0020982	chr2	201828022	201843705	chrY	2559266	2614373
cis_mRNA _overlap	Lnc-AntiCompleteIn-mRNAIntron	NONHSAT076380.2	NM_003879	chr2	202014579	202015883	chr2	201980877	202037411
cis_mRNA overlap	Lnc-CompleteIn-	NONHSAT076663.2	MTCONS_00104846	chr2	210887131	210889246	chr2	210867289	210901940
cis_mRNA up10k	NA	NONHSAT076705.2	MTCONS_00112858	chr2	214016667	214019086	chr2	213864410	214016333
cis_mRNA dw20k	NA	NONHSAT077129.2	NM_005687	chr2	223431624	223434438	chr2	223436162	223521074
cis_mRNA dw20k	NA	NONHSAT077878.2	NM_015148	chr2	242014096	242034916	chr2	242045514	242088919
cis_mRNA dw20k	NA	NONHSAT077932.2	MTCONS_00113778	chr2	242428943	242432702	chr2	242434122	242448110
cis_mRNA dw20k	NA	NONHSAT078117.2	NM_001122962	chr20	1453434	1454044	chr20	1455236	1472233
cis_mRNA dw20k	NA	NONHSAT078117.2	NM_001134836	chr20	1453434	1454044	chr20	1455236	1472233
cis_mRNA up10k	NA	NONHSAT078117.2	NM_016143	chr20	1453434	1454044	chr20	1422807	1448337
cis_mRNA dw20k	NA	NONHSAT078253.2	NM_020746	chr20	3867415	3869485	chr20	3827446	3856770
cis_mRNA overlap	Lnc-CompleteIn-	NONHSAT078261.2	MTCONS_00118334	chr20	3907958	3911562	chr20	3904499	3996216
cis_mRNA up10k	NA	NONHSAT078302.2	NM_001009924	chr20	5100232	5100615	chr20	5080484	5093733
tran	NA	NONHSAT078335.	MTCONS_0018041	chr20	5451842	5457780	chr7	155437203	155480457

tran	NA	NONHSAT078687.	NM_020813	chr20	13360431	13365292	chr19	57019212	57040269
cis_mRNA overlap	Lnc-CompleteIn-	NONHSAT078900. 2	NM_020343	chr20	20389471	20390912	chr20	20370272	20693266
cis_mRNA overlap	Lnc-Overlap-mRNA	NONHSAT078932. 2	NM_018474	chr20	21225026	21227196	chr20	21106624	21227258
cis_mRNA up10k	NA	NONHSAT079029. 2	NM_000361	chr20	23030380	23035547	chr20	23026270	23030301
cis_mRNA _overlap	Lnc-AntiOverlap-mRNA	NONHSAT079137. 2	MTCONS_0011631 5	chr20	25165310	25177508	chr20	25176306	25207365
tran	NA	NONHSAT079137.	MTCONS_0009079	chr20	25165310	25177508	chr19	20946830	20993757
tran	NA	NONHSAT079277. 2	MTCONS_0020375 9	chr20	29611879	29634007	chrUn_gl0002 41	12657	36886
cis_mRNA dw20k	NA	NONHSAT079374. 2	NM_012325	chr20	31442380	31446532	chr20	31407699	31438211
cis_mRNA _overlap	Lnc-AntiOverlap-mRNA	NONHSAT079464. 2	NM_020884	chr20	33576778	33578801	chr20	33543638	33590240
cis_mRNA overlap	Lnc-CompleteIn-	NONHSAT080186. 2	NM_006420	chr20	47551778	47552227	chr20	47538275	47653230
cis_mRNA overlap	Lnc-Overlap-mRNA	NONHSAT080611. 2	NM_006886	chr20	57603733	57617901	chr20	57603733	57607422
cis_mRNA overlap	Lnc-Overlap-mRNA	NONHSAT080611. 2	NM_016045	chr20	57603733	57617901	chr20	57608200	57617901
cis_mRNA _overlap	mRNA-CompleteIn-LncExon	NONHSAT080910. 2	NM_015894	chr20	62271058	62284963	chr20	62271058	62284963
cis_mRNA overlap	Lnc-Overlap-mRNA	NONHSAT080922. 2	NM_001305655	chr20	62367812	62369485	chr20	62367812	62370460
cis_mRNA overlap	Lnc-Overlap-mRNA	NONHSAT080922. 2	NM_017806	chr20	62367812	62369485	chr20	62367812	62370460
cis_mRNA up10k	NA	NONHSAT080922. 2	MTCONS_0011804 1	chr20	62367812	62369485	chr20	62371211	62378647

cis_mRNA up10k	NA	NONHSAT080922.2	NM_020062	chr20	62367812	62369485	chr20	62371211	62375403
cis_mRNA _overlap	Lnc-AntiCompleteIn-mRNAIntron	NONHSAT081304.2	MTCONS_00121456	chr21	19301408	19303493	chr21	19165778	19639688
cis_mRNA overlap	Lnc-CompleteIn-	NONHSAT081549.2	NM_006988	chr21	28217221	28217728	chr21	28208606	28217728
cis_mRNA dw20k	NA	NONHSAT081780.2	NM_000629	chr21	34734436	34735415	chr21	34697214	34732128
cis_mRNA overlap	Lnc-CompleteIn-	NONHSAT082035.2	NM_130436	chr21	38846871	38847759	chr21	38791207	38887679
cis_mRNA overlap	Lnc-CompleteIn-	NONHSAT082085.2	MTCONS_00122110	chr21	39645398	39647441	chr21	39601837	39673746
cis_mRNA overlap	Lnc-CompleteIn-	NONHSAT082085.2	NM_001276438	chr21	39645398	39647441	chr21	39601837	39673746
cis_mRNA dw20k	NA	NONHSAT082186.2	NM_001146218	chr21	40778283	40781266	chr21	40759692	40769815
cis_mRNA dw20k	NA	NONHSAT082260.2	NM_002462	chr21	42835538	42836237	chr21	42797958	42831141
cis_mRNA dw20k	NA	NONHSAT082319.2	NM_004915	chr21	43719104	43720919	chr21	43639267	43717354
cis_mRNA _overlap	Lnc-AntiOverlap-mRNA	NONHSAT083036.2	MTCONS_00124131	chr21	47649131	47679304	chr21	47655039	47674047
cis_mRNA _overlap	Lnc-AntiOverlap-mRNA	NONHSAT083041.2	MTCONS_00124131	chr21	47649158	47671615	chr21	47655039	47674047
cis_mRNA dw20k	NA	NONHSAT083070.2	MTCONS_00122683	chr21	47871407	47878519	chr21	47744036	47865682
cis_mRNA _overlap	Lnc-AntiCompleteIn-mRNAIntron	NONHSAT083201.2	NM_001282228	chr22	17690773	17691374	chr22	17659680	17700475
cis_mRNA overlap	Lnc-Overlap-mRNA	NONHSAT083218.2	NM_001288707	chr22	18062923	18071958	chr22	18062923	18071958

cis_mRNA dw20k	NA	NONHSAT083226.2	NM_015367	chr22	18214961	18217980	chr22	18121350	18213621
cis_mRNA_overlap	Lnc-AntiOverlap-mRNA	NONHSAT083226.2	NM_197967	chr22	18214961	18217980	chr22	18216906	18257261
cis_mRNA up10k	NA	NONHSAT083544.2	NM_032775	chr22	20850177	20856171	chr22	20795806	20850170
cis_mRNA_overlap	Lnc-AntiOverlap-mRNA	NONHSAT083615.2	NM_030573	chr22	21356175	21364414	chr22	21354061	21356404
cis_mRNA dw20k	NA	NONHSAT083616.2	NM_006767	chr22	21356211	21364663	chr22	21336558	21353326
tran	NA	NONHSAT083616.	NM_006001	chr22	21356211	21364663	chr13	19747910	19755992
tran	NA	NONHSAT083681.	MTCONS_0012471	chr22	21822387	21824214	chr22	20377669	20381779
cis_mRNA overlap	Lnc-CompleteIn-	NONHSAT083695.2	MTCONS_00127032	chr22	21982379	21982768	chr22	21982379	21984340
cis_mRNA overlap	Lnc-CompleteIn-	NONHSAT083695.2	NM_001017964	chr22	21982379	21982768	chr22	21982379	21984340
cis_mRNA up10k	NA	NONHSAT083704.2	NM_148176	chr22	22011822	22016651	chr22	22020273	22052202
cis_mRNA_overlap	mRNA-AntiCompleteIn-LncIntron	NONHSAT083925.2	MTCONS_00125082	chr22	23980675	24059610	chr22	24037976	24041363
cis_mRNA up10k	NA	NONHSAT083931.2	MTCONS_00125082	chr22	23980679	24034768	chr22	24037976	24041363
cis_mRNA dw20k	NA	NONHSAT084323.2	MTCONS_00125117	chr22	24379874	24384271	chr22	24373095	24376211
cis_mRNA overlap	Lnc-Overlap-mRNA	NONHSAT084323.2	NM_000853	chr22	24379874	24384271	chr22	24376133	24384311
cis_mRNA dw20k	NA	NONHSAT084887.2	MTCONS_00127603	chr22	31831223	31834552	chr22	31835345	31889009
cis_mRNA dw20k	NA	NONHSAT084887.2	NM_019843	chr22	31831223	31834552	chr22	31835345	31885874
tran	NA	NONHSAT085076.	NM_001317948	chr22	36426576	36434888	chr5	175792471	175796959

cis_mRNA overlap	Lnc-CompleteIn-	NONHSAT085104.2	NM_002473	chr22	36730924	36732334	chr22	36677323	36784112
cis_mRNA dw20k	NA	NONHSAT086789.2	NM_006116	chr22	39828165	39833133	chr22	39795759	39827887
tran	NA	NONHSAT086823.	NM_015933	chr22	40356401	40360687	chr3	48481686	48485537
tran	NA	NONHSAT086890.	MTCONS_0007205	chr22	41581221	41593505	chr16	1413206	1464721
cis_mRNA dw20k	NA	NONHSAT087035.2	MTCONS_00128124	chr22	42539289	42540501	chr22	42556019	42666676
cis_mRNA up10k	NA	NONHSAT087520.2	MTCONS_00128511	chr22	51024687	51025408	chr22	51017387	51021428
cis_mRNA up10k	NA	NONHSAT087526.2	MTCONS_00128511	chr22	51030723	51035836	chr22	51017387	51021428
tran	NA	NONHSAT087541.	MTCONS_0009079	chr22	51195376	51227614	chr19	20946830	20993757
cis_mRNA up10k	NA	NONHSAT087647.2	MTCONS_00128880	chr3	5018474	5020327	chr3	5021097	5044868
cis_mRNA up10k	NA	NONHSAT087770.2	NM_001292043	chr3	9429771	9432288	chr3	9439384	9519838
tran	NA	NONHSAT087785.	MTCONS_0010176	chr3	9432996	9439188	chr2	113032498	113077605
tran	NA	NONHSAT087788.	MTCONS_0015300	chr3	9433065	9438308	chr5	79783800	79842982
cis_mRNA_overlap	Lnc-AntiOverlap-mRNA	NONHSAT088390.2	NM_001291694	chr3	15083911	15086844	chr3	14989091	15090786
cis_mRNA overlap	Lnc-Overlap-mRNA	NONHSAT088390.2	MTCONS_00135213	chr3	15083911	15086844	chr3	15083911	15106844
cis_mRNA_overlap	Lnc-AntiOverlap-mRNA	NONHSAT088391.2	NM_001291694	chr3	15083911	15086844	chr3	14989091	15090786
cis_mRNA overlap	Lnc-Overlap-mRNA	NONHSAT088391.2	MTCONS_00135213	chr3	15083911	15086844	chr3	15083911	15106844
cis_mRNA_overlap	Lnc-AntiCompleteIn-mRNAExon	NONHSAT088393.2	NM_001291694	chr3	15084898	15090004	chr3	14989091	15090786
cis_mRNA overlap	Lnc-CompleteIn-	NONHSAT088393.2	MTCONS_00135213	chr3	15084898	15090004	chr3	15083911	15106844
tran	NA	NONHSAT088803.	MTCONS_0006354	chr3	29657466	29684300	chr15	76196200	76229582

tran	NA	NONHSAT088803.	MTCONS_0012201	chr3	29657466	29684300	chr21	37529006	37668070
cis_mRNA dw20k	NA	NONHSAT088828. 2	NM_003242	chr3	30739153	30741068	chr3	30647994	30735633
tran	NA	NONHSAT088878.	MTCONS_0009079	chr3	32303234	32305396	chr19	20946830	20993757
cis_mRNA _overlap	Lnc-AntiOverlap-mRNA	NONHSAT088941. 2	MTCONS_0012976 2	chr3	33838680	33840555	chr3	33840063	33911199
cis_mRNA overlap	Lnc-Overlap-mRNA	NONHSAT088946. 2	MTCONS_0012976 2	chr3	33896110	33906809	chr3	33840063	33911199
cis_mRNA up10k	NA	NONHSAT089024. 2	NM_005808	chr3	37832356	37903265	chr3	37903669	38025960
cis_mRNA up10k	NA	NONHSAT089031. 2	NM_001008392	chr3	37859768	37903256	chr3	37903669	38025960
cis_mRNA dw20k	NA	NONHSAT089127. 2	NM_005201	chr3	39376470	39377430	chr3	39371197	39375171
cis_mRNA _overlap	Lnc-AntiCompleteIn -mRNAIntron	NONHSAT089391. 2	NM_015340	chr3	45524187	45551037	chr3	45430075	45590328
cis_mRNA overlap	Lnc-Overlap-mRNA	NONHSAT089601. 2	NM_001190317	chr3	48730884	48754711	chr3	48730884	48754711
cis_mRNA _overlap	Lnc-AntiOverlap-mRNA	NONHSAT089734. 2	NM_021971	chr3	49760258	49762050	chr3	49758909	49761407
cis_mRNA _overlap	Lnc-AntiOverlap-mRNA	NONHSAT089734. 2	NM_153273	chr3	49760258	49762050	chr3	49761728	49823973
cis_mRNA dw20k	NA	NONHSAT089837. 2	MTCONS_0013045 2	chr3	50396941	50400230	chr3	50387450	50391496
cis_mRNA up10k	NA	NONHSAT089837. 2	NM_006545	chr3	50396941	50400230	chr3	50384919	50388486
cis_mRNA dw20k	NA	NONHSAT089842. 2	MTCONS_0013045 2	chr3	50403476	50406151	chr3	50387450	50391496
cis_mRNA dw20k	NA	NONHSAT089900. 2	NM_017442	chr3	52251032	52253028	chr3	52255096	52260179

cis_mRNA dw20k	NA	NONHSAT089900.2	NM_199166	chr3	52251032	52253028	chr3	52232099	52248343
cis_mRNA dw20k	NA	NONHSAT090006.2	NM_022899	chr3	53893020	53895603	chr3	53901093	53916229
cis_mRNA up10k	NA	NONHSAT090127.2	NM_001128214	chr3	58457376	58470480	chr3	58477823	58488087
tran	NA	NONHSAT090683.	MTCONS_0009079	chr3	87138430	87147851	chr19	20946830	20993757
cis_mRNA dw20k	NA	NONHSAT090702.2	NM_018293	chr3	88210584	88216245	chr3	88188262	88193814
cis_mRNA dw20k	NA	NONHSAT090702.2	NM_173824	chr3	88210584	88216245	chr3	88198893	88207115
tran	NA	NONHSAT090756.	NM_001190470	chr3	96336029	96336495	chr5	79945819	79946854
cis_mRNA dw20k	NA	NONHSAT090915.2	MTCONS_00131556	chr3	101582412	101582933	chr3	101568358	101579869
cis_mRNA dw20k	NA	NONHSAT090915.2	MTCONS_00131557	chr3	101582412	101582933	chr3	101568358	101579869
cis_mRNA dw20k	NA	NONHSAT090915.2	MTCONS_00131559	chr3	101582412	101582933	chr3	101568358	101579869
cis_mRNA dw20k	NA	NONHSAT090916.2	MTCONS_00131556	chr3	101583057	101583644	chr3	101568358	101579869
cis_mRNA dw20k	NA	NONHSAT090916.2	MTCONS_00131559	chr3	101583057	101583644	chr3	101568358	101579869
cis_mRNA dw20k	NA	NONHSAT090970.2	MTCONS_00137185	chr3	105565135	105569968	chr3	105571556	105587887
cis_mRNA overlap	Lnc-CompleteIn-	NONHSAT090970.2	MTCONS_00137180	chr3	105565135	105569968	chr3	105418985	105587891
cis_mRNA overlap	Lnc-CompleteIn-	NONHSAT090970.2	MTCONS_00137181	chr3	105565135	105569968	chr3	105435579	105587893
cis_mRNA overlap	Lnc-CompleteIn-	NONHSAT090970.2	MTCONS_00137183	chr3	105565135	105569968	chr3	105475127	105587892
cis_mRNA dw20k	NA	NONHSAT091074.2	NM_198793	chr3	107732834	107757940	chr3	107761941	107809935
cis_mRNA dw20k	NA	NONHSAT091085.2	NM_007072	chr3	108106443	108108128	chr3	108021332	108097131

cis_mRNA overlap	Lnc-CompleteIn-	NONHSAT091152.2	NM_145753	chr3	111615806	111619675	chr3	111578027	111695364
cis_mRNA up10k	NA	NONHSAT091161.2	MTCONS_00137426	chr3	111852270	111854206	chr3	111839688	111852152
cis_mRNA dw20k	NA	NONHSAT091178.2	NM_181780	chr3	112130091	112179627	chr3	112182813	112218408
cis_mRNA dw20k	NA	NONHSAT091524.2	NM_198402	chr3	123210404	123211680	chr3	123213363	123303924
cis_mRNA overlap	Lnc-CompleteIn-	NONHSAT091691.2	MTCONS_00137845	chr3	126169968	126171749	chr3	126156444	126194762
cis_mRNA dw20k	NA	NONHSAT092318.2	NM_173543	chr3	137764168	137771903	chr3	137780827	137834451
cis_mRNA dw20k	NA	NONHSAT092521.2	NM_004267	chr3	142849528	142851286	chr3	142838618	142842856
cis_mRNA _overlap	Lnc-AntiOverlap-mRNA	NONHSAT092686.2	NM_001303264	chr3	150125889	150127466	chr3	150126122	150177905
tran	NA	NONHSAT092902.	NM_199187	chr3	156880452	156881721	chr12	53342655	53346685
cis_mRNA dw20k	NA	NONHSAT093012.2	MTCONS_00133295	chr3	160980940	161015792	chr3	160938102	160969795
cis_mRNA up10k	NA	NONHSAT093153.2	NM_018657	chr3	169482251	169482848	chr3	169490853	169507504
cis_mRNA up10k	NA	NONHSAT093200.2	NM_005602	chr3	170128300	170136403	chr3	170136653	170152479
cis_mRNA dw20k	NA	NONHSAT093388.2	NM_006218	chr3	178953578	178955289	chr3	178866311	178952497
tran	NA	NONHSAT093398.	MTCONS_0002866	chr3	179115496	179116471	chr11	10477481	10529126
cis_mRNA overlap	Lnc-Overlap-mRNA	NONHSAT093857.2	NM_001967	chr3	186504280	186505311	chr3	186501361	186507685
tran	NA	NONHSAT093868.	MTCONS_0003154	chr3	186528669	186543310	chr11	89585943	89596997
cis_mRNA overlap	Lnc-CompleteIn-	NONHSAT093953.2	MTCONS_00134257	chr3	190342384	190342958	chr3	190231840	190350769
cis_mRNA overlap	Lnc-CompleteIn-	NONHSAT094064.2	MTCONS_00134375	chr3	193853937	193854176	chr3	193853931	193856401

cis_mRNA overlap	Lnc-CompleteIn-	NONHSAT094150.2	NM_012287	chr3	195001452	195003470	chr3	194995465	195163817
cis_mRNA up10k	NA	NONHSAT094150.2	NM_152531	chr3	195001452	195003470	chr3	194789013	194991895
cis_mRNA overlap	Lnc-Overlap-mRNA	NONHSAT094280.2	MTCONS_00139903	chr3	195685078	195690326	chr3	195684922	195717189
cis_mRNA overlap	Lnc-Overlap-mRNA	NONHSAT094312.2	NM_001312673	chr3	195961240	195964785	chr3	195964616	196014623
cis_mRNA up10k	NA	NONHSAT094353.2	NM_198565	chr3	196363400	196366498	chr3	196366567	196388874
cis_mRNA up10k	NA	NONHSAT094430.2	MTCONS_00140000	chr3	197363338	197374168	chr3	197328847	197354756
cis_mRNA overlap	Lnc-Overlap-mRNA	NONHSAT094493.2	NM_001145248	chr3	197880121	197925886	chr3	197879237	197907728
cis_mRNA dw20k	NA	NONHSAT094547.2	NM_133474	chr4	419562	421671	chr4	433773	492960
cis_mRNA up10k	NA	NONHSAT094688.2	NM_001017405	chr4	1243239	1282079	chr4	1283639	1333936
cis_mRNA up10k	NA	NONHSAT094688.2	NM_001328	chr4	1243239	1282079	chr4	1205228	1242908
cis_mRNA dw20k	NA	NONHSAT094698.2	NM_001017405	chr4	1336582	1340665	chr4	1283639	1333936
cis_mRNA up10k	NA	NONHSAT094698.2	MTCONS_00141295	chr4	1336582	1340665	chr4	1340844	1384065
cis_mRNA overlap	Lnc-CompleteIn-	NONHSAT094882.2	NM_002337	chr4	3514297	3520638	chr4	3505324	3534224
cis_mRNA up10k	NA	NONHSAT095005.2	MTCONS_00141580	chr4	6669919	6675170	chr4	6675678	6677814
cis_mRNA overlap	Lnc-CompleteIn-	NONHSAT095146.2	NM_020041	chr4	9870401	9874487	chr4	9827848	10023114
cis_mRNA overlap	Lnc-CompleteIn-	NONHSAT095695.2	NM_001130834	chr4	16876091	16879230	chr4	16503157	16900432
cis_mRNA dw20k	NA	NONHSAT095883.2	MTCONS_00142087	chr4	26780029	26786640	chr4	26585546	26767937

cis_mRNA overlap	Lnc-CompleteIn-	NONHSAT096254.2	MTCONS_00142508	chr4	47552735	47556650	chr4	47487197	47559948
cis_mRNA _overlap	Lnc-AntiCompleteIn-mRNAIntron	NONHSAT096259.2	NM_001278585	chr4	47708389	47709004	chr4	47596015	47840123
cis_mRNA dw20k	NA	NONHSAT096530.2	NM_005612	chr4	57815289	57816376	chr4	57774042	57802010
cis_mRNA _overlap	Lnc-AntiOverlap-mRNA	NONHSAT096734.2	NM_001076	chr4	69403133	69513135	chr4	69512315	69536494
cis_mRNA overlap	Lnc-CompleteIn-	NONHSAT097124.2	NM_058172	chr4	80826725	80992808	chr4	80822771	80994477
cis_mRNA overlap	Lnc-Overlap-mRNA	NONHSAT097124.2	NM_001145794	chr4	80826725	80992808	chr4	80898662	80994477
cis_mRNA overlap	Lnc-Overlap-mRNA	NONHSAT097372.2	MTCONS_00143432	chr4	89630847	89633484	chr4	89513574	89651254
cis_mRNA dw20k	NA	NONHSAT097386.2	NM_198281	chr4	90162421	90164636	chr4	90165429	90229161
tran	NA	NONHSAT097413.	NM_021109	chr4	91759652	91760263	chrX	12993226	12995346
cis_mRNA overlap	Lnc-Overlap-mRNA	NONHSAT097487.2	MTCONS_00143552	chr4	99313102	99317812	chr4	99182527	99317812
cis_mRNA overlap	Lnc-CompleteIn-	NONHSAT097488.2	NM_001100430	chr4	99330441	99330914	chr4	99182527	99365012
cis_mRNA overlap	Lnc-Overlap-mRNA	NONHSAT097602.2	NM_181891	chr4	103717134	103749307	chr4	103715540	103749105
cis_mRNA _overlap	Lnc-AntiOverlap-mRNA	NONHSAT097610.2	NM_181891	chr4	103746198	103764918	chr4	103715540	103749105
tran	NA	NONHSAT097644.	MTCONS_0009079	chr4	104882773	104958080	chr19	20946830	20993757
cis_mRNA overlap	Lnc-CompleteIn-	NONHSAT097701.2	MTCONS_00147989	chr4	106962789	106964536	chr4	106962789	107237423
cis_mRNA up10k	NA	NONHSAT097745.2	NM_016269	chr4	109093276	109097586	chr4	108968701	109090112
cis_mRNA up10k	NA	NONHSAT097748.2	NM_016269	chr4	109093865	109097584	chr4	108968701	109090112

cis_mRNA_overlap	Lnc-AntiOverlap-mRNA	NONHSAT098098.2	NM_001083	chr4	120548945	120551908	chr4	120415550	120549981
cis_mRNA_overlap	Lnc-AntiOverlap-mRNA	NONHSAT098098.2	NM_033437	chr4	120548945	120551908	chr4	120415550	120549239
cis_mRNA_up10k	NA	NONHSAT098098.2	NM_033430	chr4	120548945	120551908	chr4	120415550	120548442
cis_mRNA_overlap	Lnc-CompleteIn-	NONHSAT098487.2	NM_018717	chr4	140897780	140899933	chr4	140637545	141075233
cis_mRNA_up10k	NA	NONHSAT098512.2	NM_015130	chr4	141677473	141678878	chr4	141541936	141677471
cis_mRNA_dw20k	NA	NONHSAT098798.2	NM_018315	chr4	153241696	153242196	chr4	153242410	153274110
cis_mRNA_overlap	Lnc-AntiOverlap-mRNA	NONHSAT098805.2	MTCONS_00148929	chr4	153456345	153460395	chr4	153242410	153456393
cis_mRNA_up10k	NA	NONHSAT099470.2	NM_001111319	chr4	184245123	184254636	chr4	184239220	184241927
cis_mRNA_up10k	NA	NONHSAT099472.2	NM_001185149	chr4	184251303	184253742	chr4	184242917	184243579
cis_mRNA_up10k	NA	NONHSAT099472.2	NM_001111319	chr4	184251303	184253742	chr4	184239220	184241927
tran	NA	NONHSAT099830.2	NM_001286559	chr4_gl000194_random	53589	115071	chr2	95537178	95542574
cis_mRNA_dw20k	NA	NONHSAT099909.2	NM_018140	chr5	667579	668841	chr5	612405	653666
tran	NA	NONHSAT100031.	MTCONS_0013446	chr5	1568637	1579695	chr3	195384964	195460424
cis_mRNA_dw20k	NA	NONHSAT100468.2	NM_001291963	chr5	10675434	10675687	chr5	10679342	10761387
cis_mRNA_overlap	Lnc-AntiOverlap-mRNA	NONHSAT100564.2	NM_054027	chr5	14712803	14716638	chr5	14704909	14871887

cis_mRNA dw20k	NA	NONHSAT101022.2	NM_001007527	chr5	36098658	36102483	chr5	36103414	36152015
tran	NA	NONHSAT101272.	MTCONS_0009079	chr5	43054831	43055407	chr19	20946830	20993757
cis_mRNA dw20k	NA	NONHSAT101419.2	NM_181501	chr5	52251187	52255037	chr5	52084136	52249485
cis_mRNA dw20k	NA	NONHSAT101419.2	MTCONS_00152119	chr5	52251187	52255037	chr5	52217985	52250638
cis_mRNA overlap	Lnc-CompleteIn-	NONHSAT101516.2	NM_001190981	chr5	55232481	55235321	chr5	55230925	55290821
cis_mRNA overlap	Lnc-CompleteIn-	NONHSAT101522.2	MTCONS_00157520	chr5	55286238	55286707	chr5	55251341	55290821
cis_mRNA overlap	Lnc-CompleteIn-	NONHSAT101522.2	NM_002184	chr5	55286238	55286707	chr5	55230925	55290821
cis_mRNA overlap	Lnc-Overlap-mRNA	NONHSAT101974.2	MTCONS_00152633	chr5	69716075	69720986	chr5	69710723	69746189
cis_mRNA overlap	Lnc-CompleteIn-	NONHSAT102009.2	MTCONS_00157890	chr5	70525720	70525924	chr5	70264310	70585523
cis_mRNA up10k	NA	NONHSAT102030.2	NM_022132	chr5	70868036	70874285	chr5	70883115	70954530
cis_mRNA overlap	Lnc-CompleteIn-	NONHSAT102195.2	MTCONS_00158065	chr5	74343722	74349594	chr5	74319296	74349594
cis_mRNA dw20k	NA	NONHSAT102289.2	NM_018268	chr5	76723252	76725632	chr5	76726758	76788365
cis_mRNA_overlap	Lnc-AntiCompleteIn-mRNAExon	NONHSAT102414.2	NM_001190470	chr5	79946322	79946796	chr5	79945819	79946854
cis_mRNA overlap	Lnc-CompleteIn-	NONHSAT102608.2	MTCONS_00158370	chr5	87553773	87555310	chr5	87487853	87564696
cis_mRNA up10k	NA	NONHSAT102732.2	NM_004365	chr5	89705933	89712311	chr5	89689152	89705603
cis_mRNA_overlap	Lnc-AntiCompleteIn-mRNAIntron	NONHSAT102826.2	NM_173665	chr5	93928476	93930496	chr5	93854918	93954309
cis_mRNA dw20k	NA	NONHSAT102852.2	NM_198150	chr5	94946081	94955781	chr5	94890825	94940806

cis_mRNA dw20k	NA	NONHSAT102920.2	MTCONS_00153428	chr5	96371586	96373214	chr5	96270128	96365384
cis_mRNA overlap	Lnc-Overlap-mRNA	NONHSAT102964.2	MTCONS_00158639	chr5	98218838	98221935	chr5	98203525	98264675
cis_mRNA overlap	Lnc-Overlap-mRNA	NONHSAT102964.2	NM_001270	chr5	98218838	98221935	chr5	98190908	98262238
cis_mRNA up10k	NA	NONHSAT102971.2	MTCONS_00158639	chr5	98264925	98297248	chr5	98203525	98264675
cis_mRNA up10k	NA	NONHSAT102971.2	NM_001270	chr5	98264925	98297248	chr5	98190908	98262238
cis_mRNA dw20k	NA	NONHSAT103012.2	NM_175052	chr5	100206203	100207715	chr5	100221015	100238989
cis_mRNA up10k	NA	NONHSAT103433.2	NM_001286813	chr5	118677271	118680524	chr5	118690468	118730299
cis_mRNA up10k	NA	NONHSAT103508.2	NM_001031812	chr5	122844430	122844881	chr5	122847793	122952738
cis_mRNA up10k	NA	NONHSAT104153.2	NM_001007189	chr5	139496843	139501752	chr5	139505521	139508391
cis_mRNA dw20k	NA	NONHSAT104271.2	MTCONS_00159677	chr5	141018875	141021179	chr5	141032965	141061800
cis_mRNA dw20k	NA	NONHSAT104464.2	MTCONS_00154964	chr5	148250760	148257457	chr5	148206156	148244494
cis_mRNA up10k	NA	NONHSAT104475.2	MTCONS_00159865	chr5	148443066	148446548	chr5	148361713	148441974
cis_mRNA overlap	Lnc-Overlap-mRNA	NONHSAT104512.2	MTCONS_00159891	chr5	148873877	148884233	chr5	148873877	148929894
tran	NA	NONHSAT104546.	MTCONS_0011279	chr5	149719783	149720699	chr2	208470297	208490055
cis_mRNA dw20k	NA	NONHSAT104639.2	NM_198395	chr5	151186316	151190911	chr5	151151476	151184915
cis_mRNA dw20k	NA	NONHSAT104836.2	NM_052927	chr5	159488878	159492670	chr5	159502892	159546452
cis_mRNA dw20k	NA	NONHSAT104904.2	NM_199246	chr5	162875116	162877423	chr5	162864577	162872022
cis_mRNA overlap	Lnc-CompleteIn-	NONHSAT104991.2	MTCONS_00160311	chr5	167975500	167979974	chr5	167975500	168006614

cis_mRNA dw20k	NA	NONHSAT105043.2	MTCONS_00160343	chr5	169673241	169673861	chr5	169675088	169724822
cis_mRNA dw20k	NA	NONHSAT105298.2	MTCONS_00155801	chr5	175551160	175553058	chr5	175490605	175542692
cis_mRNA overlap	Lnc-Overlap-mRNA	NONHSAT105543.2	MTCONS_00160715	chr5	178048810	178050340	chr5	178029665	178054054
cis_mRNA overlap	Lnc-Overlap-mRNA	NONHSAT105595.2	MTCONS_00160755	chr5	179041180	179050670	chr5	179041179	179050722
cis_mRNA dw20k	NA	NONHSAT105621.2	NM_014275	chr5	179215129	179220536	chr5	179224598	179233952
cis_mRNA _overlap	Lnc-AntiCompleteIn-mRNAIntron	NONHSAT105819.2	MTCONS_00160829	chr5	180237297	180238375	chr5	180217541	180242621
cis_mRNA dw20k	NA	NONHSAT105837.2	MTCONS_00160855	chr5	180256959	180262725	chr5	180274611	180288286
cis_mRNA dw20k	NA	NONHSAT105936.2	NM_033549	chr5	180672876	180679438	chr5	180650263	180662808
cis_mRNA _overlap	Lnc-AntiCompleteIn-mRNAIntron	NONHSAT106545.2	NM_001500	chr6	1739435	1746752	chr6	1624035	2245868
cis_mRNA overlap	Lnc-CompleteIn-	NONHSAT106573.2	MTCONS_00168413	chr6	1971122	1973057	chr6	1548086	2245868
cis_mRNA overlap	Lnc-Overlap-mRNA	NONHSAT106882.2	NM_001271825	chr6	2940298	2956422	chr6	2948393	2962404
cis_mRNA dw20k	NA	NONHSAT106935.2	MTCONS_00162549	chr6	3025025	3027659	chr6	3000050	3023034
cis_mRNA dw20k	NA	NONHSAT106937.2	NM_000904	chr6	3026411	3027751	chr6	3000050	3020110
cis_mRNA overlap	Lnc-Overlap-mRNA	NONHSAT107038.2	NM_001135750	chr6	3258996	3262269	chr6	3259162	3264097
cis_mRNA dw20k	NA	NONHSAT107042.2	NM_015482	chr6	3266750	3267543	chr6	3269208	3456793
tran	NA	NONHSAT107042.	MTCONS_0016852	chr6	3266750	3267543	chr6	2887500	2903546
tran	NA	NONHSAT107042.	MTCONS_0003277	chr6	3266750	3267543	chr11	129685741	129733498

cis_mRNA up10k	NA	NONHSAT107775.2	MTCONS_00163106	chr6	12007817	12009073	chr6	12012900	12166086
cis_mRNA dw20k	NA	NONHSAT107804.2	NM_018988	chr6	13358067	13361194	chr6	13363587	13487869
cis_mRNA overlap	Lnc-Overlap-mRNA	NONHSAT108136.2	MTCONS_00169392	chr6	25081640	25085013	chr6	25079705	25138587
cis_mRNA up10k	NA	NONHSAT108251.2	NM_003525	chr6	26272386	26272940	chr6	26273204	26273640
cis_mRNA up10k	NA	NONHSAT108614.2	NM_172016	chr6	30255174	30294933	chr6	30295008	30311506
cis_mRNA up10k	NA	NONHSAT108666.2	NM_133471	chr6	30655827	30656316	chr6	30644166	30655672
cis_mRNA dw20k	NA	NONHSAT108676.2	NM_178014	chr6	30710700	30711393	chr6	30687978	30693203
cis_mRNA overlap	Lnc-Overlap-mRNA	NONHSAT108748.2	NM_001243042	chr6	31238876	31239867	chr6	31236526	31239913
cis_mRNA overlap	Lnc-Overlap-mRNA	NONHSAT108748.2	NM_002117	chr6	31238876	31239867	chr6	31236526	31239913
cis_mRNA dw20k	NA	NONHSAT108925.2	MTCONS_00164266	chr6	32632577	32632859	chr6	32600537	32614759
cis_mRNA overlap	Lnc-Overlap-mRNA	NONHSAT108925.2	NM_002123	chr6	32632577	32632859	chr6	32627241	32634466
cis_mRNA dw20k	NA	NONHSAT112124.2	NM_006703	chr6	34247457	34249969	chr6	34254973	34360457
cis_mRNA dw20k	NA	NONHSAT112201.2	NM_001315	chr6	36084167	36091301	chr6	35995454	36079013
cis_mRNA dw20k	NA	NONHSAT112300.2	NM_021943	chr6	38129773	38132113	chr6	37787307	38122399
cis_mRNA overlap	Lnc-Overlap-mRNA	NONHSAT112719.2	MTCONS_00164716	chr6	41106339	41107767	chr6	41040707	41108573
cis_mRNA up10k	NA	NONHSAT112955.2	MTCONS_00164960	chr6	44204713	44208319	chr6	44214695	44221625
cis_mRNA up10k	NA	NONHSAT113132.2	NM_012288	chr6	52442852	52448389	chr6	52362200	52441862

cis_mRNA up10k	NA	NONHSAT113411.2	MTCONS_00165318	chr6	69342555	69344919	chr6	69345228	69352537
cis_mRNA up10k	NA	NONHSAT113411.2	NM_001704	chr6	69342555	69344919	chr6	69345632	70099403
cis_mRNA overlap	Lnc-CompleteIn-	NONHSAT113568.2	MTCONS_00171302	chr6	74227241	74230755	chr6	74225473	74230755
tran	NA	NONHSAT113568.	MTCONS_0015731	chr6	74227241	74230755	chr5	43494871	43496989
cis_mRNA dw20k	NA	NONHSAT113570.2	NM_012123	chr6	74229103	74229803	chr6	74171454	74211179
cis_mRNA overlap	Lnc-CompleteIn-	NONHSAT113843.2	NM_001297614	chr6	86270033	86270928	chr6	86215215	86303874
cis_mRNA overlap	Lnc-CompleteIn-	NONHSAT113843.2	NM_001304479	chr6	86270033	86270928	chr6	86215215	86303874
cis_mRNA up10k	NA	NONHSAT114047.2	NM_024641	chr6	96023059	96025326	chr6	96025373	96057328
cis_mRNA overlap	Lnc-CompleteIn-	NONHSAT114109.2	NM_012160	chr6	99321601	99395882	chr6	99321601	99395882
cis_mRNA dw20k	NA	NONHSAT114311.2	NM_198081	chr6	108018503	108021422	chr6	108023361	108145521
cis_mRNA up10k	NA	NONHSAT114423.2	NM_033125	chr6	110799110	110800639	chr6	110745893	110797844
cis_mRNA dw20k	NA	NONHSAT114451.2	NM_153369	chr6	111597657	111599246	chr6	111580482	111590261
cis_mRNA overlap	Lnc-CompleteIn-	NONHSAT114451.2	MTCONS_00166355	chr6	111597657	111599246	chr6	111580482	111613986
cis_mRNA overlap	Lnc-CompleteIn-	NONHSAT114866.2	NM_002844	chr6	128768543	128770446	chr6	128289924	128841819
cis_mRNA dw20k	NA	NONHSAT115059.2	MTCONS_00172526	chr6	135353366	135354885	chr6	135356995	135376036
cis_mRNA dw20k	NA	NONHSAT115059.2	NM_001145207	chr6	135353366	135354885	chr6	135356995	135376036
cis_mRNA up10k	NA	NONHSAT115380.2	NM_007124	chr6	144606291	144609320	chr6	144612873	145174170
cis_mRNA up10k	NA	NONHSAT115544.2	MTCONS_00167545	chr6	150945813	150947222	chr6	150951359	151165589

cis_mRNA dw20k	NA	NONHSAT115652.2	NM_001286194	chr6	155161312	155163251	chr6	155054512	155155378
cis_mRNA overlap	Lnc-CompleteIn-	NONHSAT115937.2	MTCONS_00173331	chr6	161581164	161583014	chr6	161551057	161695107
cis_mRNA_overlap	Lnc-AntiOverlap-mRNA	NONHSAT116042.2	NM_145169	chr6	166756030	166756617	chr6	166733216	166756094
tran	NA	NONHSAT116053.	NM_031452	chr6	166999612	166999965	chr15	83654955	83659809
cis_mRNA overlap	Lnc-CompleteIn-	NONHSAT116227.2	MTCONS_00168184	chr6	170170509	170172138	chr6	170151718	170181680
cis_mRNA up10k	NA	NONHSAT116374.2	NM_172016	chr6	30255174	30294933	chr6	30295008	30311506
tran	NA	NONHSAT116383.2	MTCONS_00175378	chr6	30312919	30314634	chr6_ssto_hap	1642643	1644615
cis_mRNA dw20k	NA	NONHSAT116385.2	NM_172016	chr6	30312932	30314634	chr6	30295008	30311506
cis_mRNA dw20k	NA	NONHSAT116462.2	NM_002586	chr6	32148745	32150708	chr6	32152510	32157963
cis_mRNA up10k	NA	NONHSAT116485.2	NM_002118	chr6	32916390	32917448	chr6	32902406	32908847
cis_mRNA up10k	NA	NONHSAT116589.2	NM_172016	chr6	30255174	30294933	chr6	30295008	30311506
tran	NA	NONHSAT116598.2	MTCONS_00175378	chr6	30312919	30314634	chr6_ssto_hap	1642643	1644615
cis_mRNA dw20k	NA	NONHSAT116600.2	NM_172016	chr6	30312932	30314634	chr6	30295008	30311506
tran	NA	NONHSAT116614.	MTCONS_0009310	chr6	30466133	30466723	chr19	55417508	55445875
cis_mRNA overlap	Lnc-Overlap-mRNA	NONHSAT116629.2	NM_002714	chr6	30577724	30585021	chr6	30568177	30585084
cis_mRNA up10k	NA	NONHSAT116629.2	NM_014046	chr6	30577724	30585021	chr6	30585486	30594174
cis_mRNA overlap	Lnc-Overlap-mRNA	NONHSAT116694.2	NM_001243042	chr6	31238876	31239867	chr6	31236526	31239913
cis_mRNA overlap	Lnc-Overlap-mRNA	NONHSAT116694.2	NM_002117	chr6	31238876	31239867	chr6	31236526	31239913

cis_mRNA overlap	Lnc-CompleteIn-	NONHSAT116704.2	NM_001289152	chr6	31368424	31378574	chr6	31367561	31383093
cis_mRNA overlap	Lnc-Overlap-mRNA	NONHSAT116724.2	NM_080686	chr6	31601731	31602533	chr6	31588450	31605554
cis_mRNA dw20k	NA	NONHSAT116732.2	NM_080686	chr6	31608602	31609922	chr6	31588450	31605554
cis_mRNA dw20k	NA	NONHSAT116733.2	NM_004638	chr6	31610461	31611977	chr6	31588450	31605554
cis_mRNA dw20k	NA	NONHSAT116735.2	NM_001199237	chr6	31626106	31628496	chr6	31629006	31634060
cis_mRNA overlap	Lnc-CompleteIn-	NONHSAT116767.2	MTCONS_00164185	chr6	31919192	31919861	chr6	31828012	32009447
cis_mRNA dw20k	NA	NONHSAT116798.2	NM_002586	chr6	32148745	32150708	chr6	32152510	32157963
cis_mRNA up10k	NA	NONHSAT116830.2	NM_002118	chr6	32916390	32917448	chr6	32902406	32908847
cis_mRNA overlap	Lnc-Overlap-mRNA	NONHSAT116852.2	NM_022553	chr6	33235243	33235930	chr6	33218049	33239742
cis_mRNA up10k	NA	NONHSAT116942.2	NM_172016	chr6	30255174	30294933	chr6	30295008	30311506
tran	NA	NONHSAT116949.2	MTCONS_00175378	chr6	30312919	30314634	chr6_ssto_hap	1642643	1644615
cis_mRNA dw20k	NA	NONHSAT116951.2	NM_172016	chr6	30312932	30314634	chr6	30295008	30311506
tran	NA	NONHSAT116965.	MTCONS_0009310	chr6	30466133	30466723	chr19	55417508	55445875
cis_mRNA overlap	Lnc-Overlap-mRNA	NONHSAT116979.2	NM_002714	chr6	30577724	30585021	chr6	30568177	30585084
cis_mRNA up10k	NA	NONHSAT116979.2	NM_014046	chr6	30577724	30585021	chr6	30585486	30594174
tran	NA	NONHSAT117046.	MTCONS_0007555	chr6	31274808	31275022	chr17	1933431	1946725
cis_mRNA overlap	Lnc-Overlap-mRNA	NONHSAT117064.2	NM_080686	chr6	31601731	31602533	chr6	31588450	31605554
cis_mRNA dw20k	NA	NONHSAT117072.2	NM_080686	chr6	31608602	31609922	chr6	31588450	31605554

cis_mRNA dw20k	NA	NONHSAT117073.2	NM_004638	chr6	31610461	31611977	chr6	31588450	31605554
cis_mRNA overlap	Lnc-CompleteIn-	NONHSAT117110.2	MTCONS_00164185	chr6	31919192	31919861	chr6	31828012	32009447
cis_mRNA up10k	NA	NONHSAT117178.2	NM_002118	chr6	32916390	32917448	chr6	32902406	32908847
cis_mRNA overlap	Lnc-Overlap-mRNA	NONHSAT117187.2	NM_002121	chr6	33048163	33051789	chr6	33043703	33057473
cis_mRNA overlap	Lnc-Overlap-mRNA	NONHSAT117200.2	NM_022553	chr6	33235243	33235930	chr6	33218049	33239742
cis_mRNA up10k	NA	NONHSAT117290.2	NM_172016	chr6	30255174	30294933	chr6	30295008	30311506
cis_mRNA overlap	Lnc-Overlap-mRNA	NONHSAT117330.2	NM_002714	chr6	30571350	30571989	chr6	30568177	30585084
cis_mRNA overlap	Lnc-Overlap-mRNA	NONHSAT117331.2	NM_002714	chr6	30577724	30585021	chr6	30568177	30585084
cis_mRNA up10k	NA	NONHSAT117331.2	NM_014046	chr6	30577724	30585021	chr6	30585486	30594174
cis_mRNA dw20k	NA	NONHSAT117416.2	NM_001145467	chr6	31553971	31556686	chr6	31557051	31560762
cis_mRNA overlap	Lnc-Overlap-mRNA	NONHSAT117421.2	NM_080686	chr6	31601731	31602533	chr6	31588450	31605554
cis_mRNA dw20k	NA	NONHSAT117429.2	NM_080686	chr6	31608602	31609922	chr6	31588450	31605554
cis_mRNA dw20k	NA	NONHSAT117430.2	NM_004638	chr6	31610461	31611977	chr6	31588450	31605554
cis_mRNA dw20k	NA	NONHSAT117465.2	NM_002586	chr6	32148745	32150708	chr6	32152510	32157963
cis_mRNA up10k	NA	NONHSAT117528.2	NM_002118	chr6	32916390	32917448	chr6	32902406	32908847
cis_mRNA overlap	Lnc-Overlap-mRNA	NONHSAT117632.2	NM_002714	chr6	30571350	30571989	chr6	30568177	30585084
cis_mRNA overlap	Lnc-Overlap-mRNA	NONHSAT117633.2	NM_002714	chr6	30577724	30585021	chr6	30568177	30585084

cis_mRNA up10k	NA	NONHSAT117633.2	NM_014046	chr6	30577724	30585021	chr6	30585486	30594174
cis_mRNA overlap	Lnc-Overlap-mRNA	NONHSAT117721.2	NM_080686	chr6	31601731	31602533	chr6	31588450	31605554
cis_mRNA dw20k	NA	NONHSAT117729.2	NM_080686	chr6	31608602	31609922	chr6	31588450	31605554
cis_mRNA dw20k	NA	NONHSAT117730.2	NM_004638	chr6	31610461	31611977	chr6	31588450	31605554
cis_mRNA overlap	Lnc-CompleteIn-	NONHSAT117765.2	MTCONS_00164185	chr6	31919192	31919861	chr6	31828012	32009447
cis_mRNA dw20k	NA	NONHSAT117800.2	NM_002586	chr6	32148745	32150708	chr6	32152510	32157963
cis_mRNA up10k	NA	NONHSAT117833.2	NM_002118	chr6	32916390	32917448	chr6	32902406	32908847
cis_mRNA overlap	Lnc-Overlap-mRNA	NONHSAT117853.2	NM_022553	chr6	33235243	33235930	chr6	33218049	33239742
cis_mRNA up10k	NA	NONHSAT117953.2	NM_172016	chr6	30255174	30294933	chr6	30295008	30311506
tran	NA	NONHSAT117959.2	MTCONS_00175378	chr6	30312919	30314634	chr6_ssto_hap	1642643	1644615
cis_mRNA dw20k	NA	NONHSAT117961.2	NM_172016	chr6	30312932	30314634	chr6	30295008	30311506
tran	NA	NONHSAT117975.	MTCONS_0009310	chr6	30466133	30466723	chr19	55417508	55445875
cis_mRNA overlap	Lnc-Overlap-mRNA	NONHSAT117989.2	NM_002714	chr6	30577724	30585021	chr6	30568177	30585084
cis_mRNA up10k	NA	NONHSAT117989.2	NM_014046	chr6	30577724	30585021	chr6	30585486	30594174
cis_mRNA overlap	Lnc-Overlap-mRNA	NONHSAT118079.2	NM_080686	chr6	31601731	31602533	chr6	31588450	31605554
cis_mRNA dw20k	NA	NONHSAT118088.2	NM_080686	chr6	31608602	31609922	chr6	31588450	31605554
cis_mRNA dw20k	NA	NONHSAT118089.2	NM_004638	chr6	31610461	31611977	chr6	31588450	31605554
cis_mRNA overlap	Lnc-CompleteIn-	NONHSAT118118.2	MTCONS_00164185	chr6	31919192	31919861	chr6	31828012	32009447

cis_mRNA_overlap	Lnc-AntiOverlap-mRNA	NONHSAT118126.2	NM_004197	chr6	31937587	31940015	chr6	31939646	31949223
cis_mRNA_dw20k	NA	NONHSAT118149.2	NM_002586	chr6	32148745	32150708	chr6	32152510	32157963
cis_mRNA_up10k	NA	NONHSAT118182.2	NM_002118	chr6	32916390	32917448	chr6	32902406	32908847
cis_mRNA_overlap	Lnc-Overlap-mRNA	NONHSAT118206.2	NM_022553	chr6	33235243	33235930	chr6	33218049	33239742
cis_mRNA_up10k	NA	NONHSAT118303.2	NM_172016	chr6	30255174	30294933	chr6	30295008	30311506
tran	NA	NONHSAT118312.2	MTCONS_00175378	chr6	30312919	30314634	chr6_ssto_hap	1642643	1644615
cis_mRNA_dw20k	NA	NONHSAT118314.2	NM_172016	chr6	30312932	30314634	chr6	30295008	30311506
tran	NA	NONHSAT118329.	MTCONS_0009310	chr6	30466133	30466723	chr19	55417508	55445875
cis_mRNA_overlap	Lnc-Overlap-mRNA	NONHSAT118344.2	NM_002714	chr6	30577724	30585021	chr6	30568177	30585084
cis_mRNA_up10k	NA	NONHSAT118344.2	NM_014046	chr6	30577724	30585021	chr6	30585486	30594174
cis_mRNA_overlap	Lnc-CompleteIn-	NONHSAT118394.2	NM_001289152	chr6	31368424	31378574	chr6	31367561	31383093
cis_mRNA_overlap	Lnc-Overlap-mRNA	NONHSAT118408.2	NM_205839	chr6	31553971	31556686	chr6	31553956	31556686
cis_mRNA_overlap	Lnc-Overlap-mRNA	NONHSAT118414.2	NM_080686	chr6	31601731	31602533	chr6	31588450	31605554
cis_mRNA_dw20k	NA	NONHSAT118422.2	NM_080686	chr6	31608602	31609922	chr6	31588450	31605554
cis_mRNA_dw20k	NA	NONHSAT118423.2	NM_004638	chr6	31610461	31611977	chr6	31588450	31605554
cis_mRNA_dw20k	NA	NONHSAT118425.2	NM_001199237	chr6	31626106	31628496	chr6	31629006	31634060
cis_mRNA_overlap	Lnc-CompleteIn-	NONHSAT118455.2	MTCONS_00164185	chr6	31919192	31919861	chr6	31828012	32009447

cis_mRNA _overlap	Lnc-AntiOverlap-mRNA	NONHSAT118463.2	NM_004197	chr6	31937587	31940015	chr6	31939646	31949223
cis_mRNA dw20k	NA	NONHSAT118485.2	NM_002586	chr6	32148745	32150708	chr6	32152510	32157963
cis_mRNA up10k	NA	NONHSAT118524.2	NM_002118	chr6	32916390	32917448	chr6	32902406	32908847
cis_mRNA overlap	Lnc-Overlap-mRNA	NONHSAT118534.2	NM_002121	chr6	33048163	33051789	chr6	33043703	33057473
cis_mRNA overlap	Lnc-Overlap-mRNA	NONHSAT118547.2	NM_022553	chr6	33235243	33235930	chr6	33218049	33239742
cis_mRNA overlap	Lnc-Overlap-mRNA	NONHSAT118559.2	NM_001014838	chr6	33384338	33385885	chr6	33384319	33386065
cis_mRNA overlap	Lnc-CompleteIn-	NONHSAT119053.2	MTCONS_00175851	chr7	6694495	6696063	chr7	6676953	6697910
cis_mRNA up10k	NA	NONHSAT119622.2	NM_002137	chr7	26245656	26248096	chr7	26229556	26240413
cis_mRNA dw20k	NA	NONHSAT119666.2	NM_030661	chr7	27135852	27139585	chr7	27145809	27159214
tran	NA	NONHSAT119833.	NM_147128	chr7	29724388	29725437	chr7	30323923	30407308
cis_mRNA _overlap	Lnc-AntiOverlap-mRNA	NONHSAT119844.2	NM_017946	chr7	30028216	30065216	chr7	30050199	30066417
cis_mRNA _overlap	Lnc-AntiOverlap-mRNA	NONHSAT119987.2	MTCONS_00181587	chr7	32797898	32802536	chr7	32620553	32801399
cis_mRNA dw20k	NA	NONHSAT120258.2	NM_002192	chr7	41724713	41726643	chr7	41728601	41742706
cis_mRNA overlap	Lnc-CompleteIn-	NONHSAT120330.2	MTCONS_00181869	chr7	44043645	44047465	chr7	44025239	44054104
cis_mRNA dw20k	NA	NONHSAT120336.2	MTCONS_00181880	chr7	44078648	44083895	chr7	44101312	44122139
cis_mRNA _overlap	Lnc-AntiOverlap-mRNA	NONHSAT120341.2	MTCONS_00181876	chr7	44079122	44081905	chr7	44065964	44081529

cis_mRNA dw20k	NA	NONHSAT120346.2	NM_014063	chr7	44104507	44105678	chr7	44084239	44101315
tran	NA	NONHSAT121385.	MTCONS_0018265	chr7	72634674	72649979	chr7	74572392	74587785
cis_mRNA up10k	NA	NONHSAT121450.2	NM_022170	chr7	73586631	73588688	chr7	73588706	73611429
cis_mRNA _overlap	Lnc-AntiCompleteIn-mRNAExon	NONHSAT121497.2	NM_001145064	chr7	74440309	74445630	chr7	74422160	74445635
cis_mRNA dw20k	NA	NONHSAT121601.2	NM_001540	chr7	75935609	75937653	chr7	75931875	75933614
tran	NA	NONHSAT121614.	MTCONS_0017878	chr7	76178628	76180902	chr7	102004308	102067129
cis_mRNA _overlap	Lnc-AntiOverlap-mRNA	NONHSAT121617.2	NM_152992	chr7	76178728	76250705	chr7	76239303	76256620
cis_mRNA up10k	NA	NONHSAT121828.2	NM_001243745	chr7	86954638	86974883	chr7	86974951	86989425
cis_mRNA dw20k	NA	NONHSAT121869.2	NM_024636	chr7	87900207	87903065	chr7	87905744	87936228
cis_mRNA overlap	Lnc-CompleteIn-	NONHSAT121999.2	NM_152703	chr7	92774919	92777682	chr7	92759367	92777701
cis_mRNA overlap	Lnc-Overlap-mRNA	NONHSAT122167.2	MTCONS_00183170	chr7	97599649	97601638	chr7	97481429	97601656
cis_mRNA overlap	Lnc-CompleteIn-	NONHSAT122234.2	NM_003910	chr7	99006601	99017238	chr7	99006601	99017239
cis_mRNA dw20k	NA	NONHSAT122246.2	MTCONS_00178548	chr7	99074503	99078842	chr7	99036563	99055000
cis_mRNA up10k	NA	NONHSAT122356.2	NM_178238	chr7	99933702	99949523	chr7	99955626	99965454
cis_mRNA dw20k	NA	NONHSAT122386.2	NM_006076	chr7	100169855	100176323	chr7	100136834	100165843
cis_mRNA overlap	Lnc-Overlap-mRNA	NONHSAT122386.2	NM_001168682	chr7	100169855	100176323	chr7	100169853	100171270
cis_mRNA overlap	Lnc-Overlap-mRNA	NONHSAT122386.2	NM_001289934	chr7	100169855	100176323	chr7	100171634	100183811

cis_mRNA up10k	NA	NONHSAT122392.2	NM_001040099	chr7	100200537	100201829	chr7	100210114	100213000
cis_mRNA dw20k	NA	NONHSAT122447.2	NM_030961	chr7	100737328	100741248	chr7	100728720	100735019
cis_mRNA overlap	Lnc-Overlap-mRNA	NONHSAT122605.2	MTCONS_00178857	chr7	104717563	104718255	chr7	104653665	104743284
cis_mRNA overlap	Lnc-Overlap-mRNA	NONHSAT122605.2	MTCONS_00178858	chr7	104717563	104718255	chr7	104653665	104754532
cis_mRNA dw20k	NA	NONHSAT122606.2	NM_182692	chr7	104748534	104749020	chr7	104756821	105029377
cis_mRNA overlap	Lnc-CompleteIn-	NONHSAT122683.2	NM_181581	chr7	107204402	107218968	chr7	107204402	107218968
cis_mRNA overlap	Lnc-Overlap-mRNA	NONHSAT122683.2	NM_001270419	chr7	107204402	107218968	chr7	107204402	107218968
cis_mRNA dw20k	NA	NONHSAT123178.2	NM_018077	chr7	127937738	127947816	chr7	127950436	127983962
tran	NA	NONHSAT123200.	MTCONS_0007861	chr7	128171426	128173824	chr17	60556139	60692841
tran	NA	NONHSAT123200.	MTCONS_0007861	chr7	128171426	128173824	chr17	60556139	60692841
cis_mRNA _overlap	Lnc-AntiCompleteIn-mRNAIntron	NONHSAT123292.2	NM_001130720	chr7	129007871	129011389	chr7	128864855	129070052
cis_mRNA dw20k	NA	NONHSAT123513.2	MTCONS_00184291	chr7	135069766	135071580	chr7	135071822	135194875
cis_mRNA dw20k	NA	NONHSAT123658.2	NM_030984	chr7	139723548	139724022	chr7	139528952	139720125
cis_mRNA _overlap	mRNA-CompleteIn-LncIntron	NONHSAT123872.2	NM_177437	chr7	143104906	143220542	chr7	143140546	143141502
cis_mRNA _overlap	mRNA-CompleteIn-LncIntron	NONHSAT123908.2	NM_001005328	chr7	143948357	144052231	chr7	143955789	143956721
cis_mRNA up10k	NA	NONHSAT123920.2	NM_005435	chr7	144015766	144052244	chr7	144052489	144077725
tran	NA	NONHSAT123924.	MTCONS_0011121	chr7	144040335	144052494	chr2	158592839	158675709

cis_mRNA dw20k	NA	NONHSAT124021.2	NM_001195220	chr7	148991257	148994379	chr7	148959262	148982085
cis_mRNA dw20k	NA	NONHSAT124073.2	MTCONS_00180095	chr7	149564783	149570951	chr7	149535509	149564627
cis_mRNA dw20k	NA	NONHSAT124073.2	MTCONS_00180096	chr7	149564783	149570951	chr7	149535509	149564627
cis_mRNA dw20k	NA	NONHSAT124073.2	NM_001099220	chr7	149564783	149570951	chr7	149535509	149564568
cis_mRNA_overlap	Lnc-AntiOverlap-mRNA	NONHSAT124073.2	NM_145230	chr7	149564783	149570951	chr7	149570057	149577801
cis_mRNA dw20k	NA	NONHSAT124141.2	NM_175571	chr7	150181149	150182223	chr7	150147718	150176483
cis_mRNA dw20k	NA	NONHSAT124154.2	NM_018384	chr7	150446824	150447182	chr7	150434436	150440737
cis_mRNA dw20k	NA	NONHSAT124156.2	NM_001101311	chr7	150474296	150478201	chr7	150488376	150497621
cis_mRNA dw20k	NA	NONHSAT124156.2	NM_001101312	chr7	150474296	150478201	chr7	150488376	150497621
cis_mRNA dw20k	NA	NONHSAT124156.2	NM_001101314	chr7	150474296	150478201	chr7	150488376	150497621
cis_mRNA overlap	Lnc-Overlap-mRNA	NONHSAT124614.2	NM_001303100	chr8	563151	594400	chr8	564737	681239
cis_mRNA overlap	Lnc-CompleteIn-	NONHSAT124690.2	MTCONS_00186064	chr8	1822679	1823827	chr8	1806809	1906807
cis_mRNA up10k	NA	NONHSAT125150.2	NM_001137610	chr8	12294662	12302466	chr8	12283124	12293852
cis_mRNA up10k	NA	NONHSAT125181.2	MTCONS_00190505	chr8	12623571	12645965	chr8	12579406	12616049
cis_mRNA dw20k	NA	NONHSAT125265.2	NM_013354	chr8	17081051	17083288	chr8	17086740	17104387
cis_mRNA dw20k	NA	NONHSAT125265.2	NM_054026	chr8	17081051	17083288	chr8	17089022	17104387
cis_mRNA dw20k	NA	NONHSAT125265.2	NM_016353	chr8	17081051	17083288	chr8	17013836	17080241

tran	NA	NONHSAT125545.	NM_182552	chr8	23082733	23087616	chr6	169857303	170102159
cis_mRNA dw20k	NA	NONHSAT125552. 2	NM_152272	chr8	23120444	23121615	chr8	23101150	23119513
cis_mRNA dw20k	NA	NONHSAT125622. 2	NM_024940	chr8	25272426	25273231	chr8	25042287	25270619
cis_mRNA overlap	Lnc- CompleteIn-	NONHSAT125803. 2	MTCONS_0018698 7	chr8	28921224	28922322	chr8	28747911	28923208
cis_mRNA _overlap	Lnc- AntiOverlap- mRNA	NONHSAT126247. 2	MTCONS_0019126 0	chr8	41518509	41523084	chr8	41510744	41574862
tran	NA	NONHSAT127230.	MTCONS_0018041	chr8	73973659	73975440	chr7	155437203	155480457
tran	NA	NONHSAT127625.	MTCONS_0020881	chr8	90768463	90769592	chrX	148558521	148586884
cis_mRNA _overlap	Lnc- AntiOverlap- mRNA	NONHSAT127670. 2	NM_016023	chr8	92080190	92082694	chr8	92082424	92099323
cis_mRNA dw20k	NA	NONHSAT128024. 2	NM_003406	chr8	101928753	101930297	chr8	101930804	101965623
cis_mRNA _overlap	Lnc- AntiCompleteIn -mRNAIntron	NONHSAT128253. 2	NM_001198533	chr8	107532702	107669776	chr8	107282406	107764921
cis_mRNA overlap	Lnc- CompleteIn-	NONHSAT128406. 2	NM_001101676	chr8	119271563	119274046	chr8	119201695	119634184
cis_mRNA overlap	Lnc-Overlap- mRNA	NONHSAT128531. 2	NM_007222	chr8	124260690	124286727	chr8	124260690	124286727
cis_mRNA overlap	Lnc- CompleteIn-	NONHSAT128557. 2	NM_058229	chr8	124536040	124537708	chr8	124510127	124553493
cis_mRNA dw20k	NA	NONHSAT129423. 2	NM_005293	chr8	142363503	142365465	chr8	142366586	142377365
cis_mRNA up10k	NA	NONHSAT129516. 2	NM_002346	chr8	144097834	144099804	chr8	144099902	144103827
cis_mRNA dw20k	NA	NONHSAT129636. 2	NM_001288807	chr8	144795188	144796371	chr8	144766622	144777555
cis_mRNA overlap	Lnc-Overlap- mRNA	NONHSAT129733. 2	MTCONS_0019001 4	chr8	145582255	145583559	chr8	145582217	145588184

cis_mRNA up10k	NA	NONHSAT129733.2	MTCONS_00193819	chr8	145582255	145583559	chr8	145576886	145582183
cis_mRNA up10k	NA	NONHSAT129733.2	NM_012162	chr8	145582255	145583559	chr8	145579088	145582183
cis_mRNA dw20k	NA	NONHSAT129986.2	MTCONS_00195038	chr9	2042994	2046019	chr9	2016890	2041764
cis_mRNA dw20k	NA	NONHSAT130170.2	NM_015061	chr9	7177450	7178549	chr9	6757641	7175648
cis_mRNA overlap	Lnc-CompleteIn-	NONHSAT130288.2	NM_017637	chr9	16424469	16426173	chr9	16409501	16870786
cis_mRNA dw20k	NA	NONHSAT130346.2	NM_004529	chr9	20330163	20332553	chr9	20341663	20622542
cis_mRNA dw20k	NA	NONHSAT130622.2	MTCONS_00199669	chr9	27937615	27944495	chr9	27948076	29214176
cis_mRNA overlap	Lnc-CompleteIn-	NONHSAT130627.2	MTCONS_00199669	chr9	28598666	28599290	chr9	27948076	29214176
cis_mRNA overlap	Lnc-Overlap-mRNA	NONHSAT130630.2	NM_001258282	chr9	29185844	29214176	chr9	27948084	29212998
cis_mRNA up10k	NA	NONHSAT130632.2	MTCONS_00199669	chr9	29214319	29220139	chr9	27948076	29214176
cis_mRNA _overlap	Lnc-AntiOverlap-mRNA	NONHSAT130669.2	NM_001195622	chr9	32551142	32553015	chr9	32540542	32552626
cis_mRNA dw20k	NA	NONHSAT130671.2	NM_002493	chr9	32551715	32553015	chr9	32553524	32573182
cis_mRNA _overlap	Lnc-AntiOverlap-mRNA	NONHSAT131029.2	NM_016734	chr9	37002694	37008037	chr9	36833272	37034476
cis_mRNA dw20k	NA	NONHSAT131072.2	NM_022490	chr9	37505587	37510377	chr9	37485932	37503694
cis_mRNA overlap	Lnc-Overlap-mRNA	NONHSAT131104.2	NM_033412	chr9	37877572	37904350	chr9	37887594	37904350
cis_mRNA dw20k	NA	NONHSAT131173.2	MTCONS_00200090	chr9	38566257	38568006	chr9	38573089	38620360

cis_mRNA overlap	Lnc-CompleteIn-	NONHSAT131209.2	NM_033655	chr9	39173791	39174510	chr9	39072764	39288300
tran	NA	NONHSAT131432.	NM_201433	chr21	10475148	10476058	chr17	9813926	10101868
cis_mRNA overlap	Lnc-CompleteIn-	NONHSAT131812.2	MTCONS_00196428	chr9	72935053	72937759	chr9	72873858	72969789
cis_mRNA dw20k	NA	NONHSAT133133.2	MTCONS_00200898	chr9	94035854	94039649	chr9	94040057	94124251
cis_mRNA _overlap	Lnc-AntiOverlap-mRNA	NONHSAT133135.2	NM_001289999	chr9	94186604	94189429	chr9	94171327	94186908
cis_mRNA dw20k	NA	NONHSAT133202.2	NM_001003800	chr9	95452063	95458422	chr9	95473645	95527083
tran	NA	NONHSAT133440.	MTCONS_0003070	chr9	99449359	99536191	chr11	67155110	67165883
tran	NA	NONHSAT133440.	NM_004488	chr9	99449359	99536191	chr3	194115550	194119995
cis_mRNA _overlap	Lnc-AntiOverlap-mRNA	NONHSAT133445.2	NM_001314059	chr9	99540340	99542614	chr9	99516831	99540348
tran	NA	NONHSAT133488.	NM_024639	chr9	99957620	99958989	chr6	26634611	26659980
cis_mRNA dw20k	NA	NONHSAT133730.2	NM_001278231	chr9	104181299	104185086	chr9	104161136	104172942
cis_mRNA overlap	Lnc-CompleteIn-	NONHSAT133813.2	NM_080546	chr9	108006929	108153680	chr9	108006894	108159628
cis_mRNA dw20k	NA	NONHSAT134020.2	MTCONS_00197705	chr9	115390750	115391983	chr9	115249170	115382591
cis_mRNA overlap	Lnc-CompleteIn-	NONHSAT134020.2	NM_133465	chr9	115390750	115391983	chr9	115249248	115427591
cis_mRNA dw20k	NA	NONHSAT134322.2	NM_015258	chr9	115923286	115924232	chr9	115927800	115983641
cis_mRNA overlap	Lnc-CompleteIn-	NONHSAT134580.2	MTCONS_00197993	chr9	125134884	125135104	chr9	125133284	125157982
cis_mRNA dw20k	NA	NONHSAT134595.2	NM_001100588	chr9	125606835	125610773	chr9	125611732	125667562
cis_mRNA dw20k	NA	NONHSAT134684.2	NM_014397	chr9	127115752	127121463	chr9	127020196	127114719

cis_mRNA dw20k	NA	NONHSAT134715.2	NM_001144877	chr9	127703476	127704887	chr9	127704888	127905838
cis_mRNA dw20k	NA	NONHSAT134898.2	NM_001006641	chr9	130875116	130876997	chr9	130830479	130871537
cis_mRNA dw20k	NA	NONHSAT134898.2	NM_052901	chr9	130875116	130876997	chr9	130860761	130871537
cis_mRNA dw20k	NA	NONHSAT134898.2	NM_198938	chr9	130875116	130876997	chr9	130882972	130890741
cis_mRNA dw20k	NA	NONHSAT134905.2	NM_001006641	chr9	130890808	130892913	chr9	130830479	130871537
cis_mRNA up10k	NA	NONHSAT134905.2	NM_198938	chr9	130890808	130892913	chr9	130882972	130890741
tran	NA	NONHSAT134945.	NM_021109	chr9	131104432	131105049	chrX	12993226	12995346
cis_mRNA dw20k	NA	NONHSAT135002.2	NM_020145	chr9	131766781	131767277	chr9	131770072	131790632
cis_mRNA _overlap	Lnc-AntiCompleteIn-mRNAIntron	NONHSAT135002.2	NM_015354	chr9	131766781	131767277	chr9	131709972	131769375
tran	NA	NONHSAT135002.	MTCONS_0003277	chr9	131766781	131767277	chr11	129685741	129733498
cis_mRNA dw20k	NA	NONHSAT135203.2	NM_001318325	chr9	134128684	134130092	chr9	134065502	134110057
cis_mRNA overlap	Lnc-CompleteIn-	NONHSAT135236.2	MTCONS_00202317	chr9	134600556	134600854	chr9	134452155	134615331
cis_mRNA dw20k	NA	NONHSAT135290.2	MTCONS_00202386	chr9	135968861	135969455	chr9	135973107	135981110
cis_mRNA dw20k	NA	NONHSAT135290.2	MTCONS_00202391	chr9	135968861	135969455	chr9	135973107	136006544
cis_mRNA dw20k	NA	NONHSAT135323.2	NM_020385	chr9	136264533	136267775	chr9	136271182	136283216
cis_mRNA dw20k	NA	NONHSAT135642.2	MTCONS_00198971	chr9	139886915	139887850	chr9	139869303	139876194
cis_mRNA overlap	Lnc-Overlap-mRNA	NONHSAT135642.2	MTCONS_00198979	chr9	139886915	139887850	chr9	139886870	139888439
cis_mRNA overlap	Lnc-Overlap-mRNA	NONHSAT135642.2	NM_183241	chr9	139886915	139887850	chr9	139886870	139888428

cis_mRNA dw20k	NA	NONHSAT135646.2	MTCONS_00198971	chr9	139887309	139888436	chr9	139869303	139876194
cis_mRNA dw20k	NA	NONHSAT135654.2	NM_207511	chr9	139931492	139932407	chr9	139921916	139931234
cis_mRNA dw20k	NA	NONHSAT135673.2	MTCONS_00202724	chr9	139996134	139998657	chr9	140004992	140009195
cis_mRNA overlap	Lnc-CompleteIn-	NONHSAT135673.2	NM_016219	chr9	139996134	139998657	chr9	139981379	140003639
cis_mRNA dw20k	NA	NONHSAT135683.2	MTCONS_00202724	chr9	139996357	139998038	chr9	140004992	140009195
cis_mRNA up10k	NA	NONHSAT135743.2	NM_138778	chr9	140477241	140479475	chr9	140449359	140473387
tran	NA	NONHSAT135796.	NM_001190470	chrM	1709	4262	chr5	79945819	79946854
cis_mRNA overlap	Lnc-Overlap-mRNA	NONHSAT136141.2	MTCONS_00206690	chrX	3809479	3820041	chrX	3739722	3820041
cis_mRNA dw20k	NA	NONHSAT136263.2	NM_078628	chrX	11786432	11790337	chrX	11776278	11786096
cis_mRNA overlap	Lnc-CompleteIn-	NONHSAT136340.2	NM_002641	chrX	15345759	15347078	chrX	15337573	15353676
cis_mRNA overlap	Lnc-Overlap-mRNA	NONHSAT136729.2	NM_001193416	chrX	41192651	41197822	chrX	41193415	41209540
cis_mRNA overlap	Lnc-Overlap-mRNA	NONHSAT136729.2	NM_001193417	chrX	41192651	41197822	chrX	41193415	41209540
tran	NA	NONHSAT136875.	MTCONS_0008799	chrX	47157251	47158120	chr18	44179670	44236996
tran	NA	NONHSAT136875.	MTCONS_0011279	chrX	47157251	47158120	chr2	208470297	208490028
cis_mRNA dw20k	NA	NONHSAT136986.2	NM_007075	chrX	48916231	48927504	chrX	48932092	48958059
cis_mRNA overlap	Lnc-CompleteIn-	NONHSAT137696.2	NM_001866	chrX	77154961	77160881	chrX	77154961	77160881
cis_mRNA dw20k	NA	NONHSAT137697.2	NM_001866	chrX	77162205	77163540	chrX	77154961	77160881
cis_mRNA dw20k	NA	NONHSAT137908.2	NM_001167971	chrX	100258821	100262962	chrX	100264334	100307105
tran	NA	NONHSAT138158.	MTCONS_0017303	chrX	109085564	109158741	chr6	150045452	150067708

cis_mRNA overlap	Lnc-CompleteIn-	NONHSAT138174.2	NM_002578	chrX	110367493	110372431	chrX	110339375	110464173
cis_mRNA overlap	Lnc-CompleteIn-	NONHSAT138914.2	MTCONS_00206311	chrX	147028461	147029103	chrX	147003682	147032647
tran	NA	NONHSAT139133.	MTCONS_0003099	chrX	153297825	153402578	chr11	71927819	71933102
cis_mRNA overlap	Lnc-Overlap-mRNA	NONHSAT139135.2	NM_004992	chrX	153297884	153353509	chrX	153287264	153363188
cis_mRNA dw20k	NA	NONHSAT139157.2	MTCONS_00206526	chrX	153625219	153627157	chrX	153607597	153620180
cis_mRNA _overlap	Lnc-AntiOverlap-mRNA	NONHSAT139157.2	NM_006013	chrX	153625219	153627157	chrX	153626406	153630680
cis_mRNA _overlap	Lnc-AntiOverlap-mRNA	NONHSAT139162.2	MTCONS_00206538	chrX	153638305	153640417	chrX	153639854	153650063
tran	NA	NONHSAT139252.	MTCONS_0003816	chrX	155250993	155255328	chr12	86468	92199
cis_mRNA up10k	NA	NONHSAT139296.2	NM_001145275	chrY	2797042	2799161	chrY	2803518	2850547
cis_mRNA up10k	NA	NONHSAT139297.2	MTCONS_00209834	chrY	2799749	2800571	chrY	2803022	2850547
cis_mRNA overlap	Lnc-CompleteIn-	NONHSAT139355.2	MTCONS_00209873	chrY	7201071	7224264	chrY	7142004	7250290
cis_mRNA overlap	Lnc-Overlap-mRNA	NONHSAT139355.2	MTCONS_00209872	chrY	7201071	7224264	chrY	7142001	7250290
cis_mRNA overlap	Lnc-Overlap-mRNA	NONHSAT139355.2	MTCONS_00209874	chrY	7201071	7224264	chrY	7142019	7250290
cis_mRNA up10k	NA	NONHSAT139486.2	MTCONS_00209955	chrY	14773021	14773995	chrY	14774265	14972768
cis_mRNA overlap	Lnc-Overlap-mRNA	NONHSAT139487.2	MTCONS_00209955	chrY	14774265	14804162	chrY	14774265	14972768
cis_mRNA overlap	Lnc-Overlap-mRNA	NONHSAT139487.2	MTCONS_00209956	chrY	14774265	14804162	chrY	14774265	14972768
cis_mRNA up10k	NA	NONHSAT139487.2	MTCONS_00209961	chrY	14774265	14804162	chrY	14813160	14972768

cis_mRNA up10k	NA	NONHSAT139487.2	NM_004654	chrY	14774265	14804162	chrY	14813160	14972768
cis_mRNA overlap	Lnc-Overlap-mRNA	NONHSAT139488.2	MTCONS_00209955	chrY	14774265	14802370	chrY	14774265	14972768
cis_mRNA overlap	Lnc-Overlap-mRNA	NONHSAT139488.2	MTCONS_00209956	chrY	14774265	14802370	chrY	14774265	14972768
cis_mRNA overlap	Lnc-Overlap-mRNA	NONHSAT139490.2	MTCONS_00209955	chrY	14774292	14777742	chrY	14774265	14972768
cis_mRNA overlap	Lnc-Overlap-mRNA	NONHSAT139490.2	MTCONS_00209956	chrY	14774292	14777742	chrY	14774265	14972768
cis_mRNA overlap	Lnc-Overlap-mRNA	NONHSAT139504.2	MTCONS_00210296	chrY	15360259	15592550	chrY	15343342	15592550
cis_mRNA overlap	Lnc-Overlap-mRNA	NONHSAT139504.2	MTCONS_00210299	chrY	15360259	15592550	chrY	15356737	15592550
cis_mRNA overlap	Lnc-Overlap-mRNA	NONHSAT139504.2	NM_001258260	chrY	15360259	15592550	chrY	15360259	15592550
cis_mRNA overlap	Lnc-Overlap-mRNA	NONHSAT139504.2	NM_182659	chrY	15360259	15592550	chrY	15409389	15592550
cis_mRNA overlap	Lnc-Overlap-mRNA	NONHSAT139621.2	MTCONS_00210024	chrY	21729236	21752304	chrY	21729199	21756123
cis_mRNA up10k	NA	NONHSAT139621.2	MTCONS_00210038	chrY	21729236	21752304	chrY	21757066	21767704
cis_mRNA overlap	Lnc-Overlap-mRNA	NONHSAT139622.2	MTCONS_00210024	chrY	21729673	21751735	chrY	21729199	21756123
cis_mRNA up10k	NA	NONHSAT139622.2	MTCONS_00210038	chrY	21729673	21751735	chrY	21757066	21767704
cis_mRNA overlap	Lnc-Overlap-mRNA	NONHSAT139623.2	MTCONS_00210024	chrY	21729715	21751733	chrY	21729199	21756123
cis_mRNA overlap	Lnc-Overlap-mRNA	NONHSAT139623.2	MTCONS_00210028	chrY	21729715	21751733	chrY	21729199	21756123
cis_mRNA up10k	NA	NONHSAT139623.2	MTCONS_00210038	chrY	21729715	21751733	chrY	21757066	21767704
cis_mRNA overlap	Lnc-CompleteIn-	NONHSAT139625.2	MTCONS_00210028	chrY	21750495	21755488	chrY	21729199	21756123

cis_mRNA dw20k	NA	NONHSAT139627.2	MTCONS_00210024	chrY	21760074	21760643	chrY	21729199	21756123
cis_mRNA dw20k	NA	NONHSAT139627.2	MTCONS_00210028	chrY	21760074	21760643	chrY	21729199	21756123
cis_mRNA overlap	Lnc-CompleteIn-	NONHSAT139627.2	MTCONS_00210038	chrY	21760074	21760643	chrY	21757066	21767704
cis_mRNA dw20k	NA	NONHSAT139628.2	MTCONS_00210406	chrY	21853827	21856492	chrY	21867301	21906825
cis_mRNA dw20k	NA	NONHSAT139628.2	NM_004653	chrY	21853827	21856492	chrY	21867301	21906825
cis_mRNA up10k	NA	NONHSAT139672.2	NM_001002758	chrY	24247839	24293631	chrY	24217903	24242154
cis_mRNA up10k	NA	NONHSAT139672.2	NM_004676	chrY	24247839	24293631	chrY	24217903	24242154
cis_mRNA overlap	Lnc-CompleteIn-	NONHSAT140187.2	MTCONS_00064806	chr15	23282490	23285566	chr15	23281071	23378259
tran	NA	NONHSAT140230.	MTCONS_0006516	chr15	34697171	34729667	chr15	34817305	34875034
cis_mRNA_overlap	Lnc-AntiOverlap-mRNA	NONHSAT140417.2	NM_001278264	chr16	8941962	8954274	chr16	8946799	8961285
cis_mRNA overlap	Lnc-Overlap-mRNA	NONHSAT140431.2	NM_003470	chr16	8988941	8992401	chr16	8985951	9057341
cis_mRNA dw20k	NA	NONHSAT140540.2	NM_000246	chr16	11027760	11030137	chr16	10971055	11018840
cis_mRNA overlap	Lnc-CompleteIn-	NONHSAT140540.2	MTCONS_00068923	chr16	11027760	11030137	chr16	10971055	11036878
cis_mRNA overlap	Lnc-Overlap-mRNA	NONHSAT140540.2	MTCONS_00068920	chr16	11027760	11030137	chr16	10971055	11030137
cis_mRNA_overlap	Lnc-AntiOverlap-mRNA	NONHSAT140542.2	MTCONS_00068920	chr16	11029866	11031993	chr16	10971055	11030137
tran	NA	NONHSAT140659.	MTCONS_0003070	chr16	14047012	14048682	chr11	67155110	67165883
cis_mRNA_overlap	Lnc-AntiOverlap-mRNA	NONHSAT140731.2	NM_001285449	chr16	15111963	15114345	chr16	15068820	15233206

tran	NA	NONHSAT140737.	NM_006985	chr16	15204359	15213071	chr16	15031300	15045931
tran	NA	NONHSAT140978.	MTCONS_0007305	chr16	21413548	21847993	chr16	29392034	29405986
tran	NA	NONHSAT141020.	MTCONS_0007305	chr16	21845985	21849091	chr16	29392034	29405986
cis_mRNA overlap	Lnc-Overlap-mRNA	NONHSAT141034.2	MTCONS_00072804	chr16	21892076	21893548	chr16	21890304	21901180
cis_mRNA up10k	NA	NONHSAT141479.2	NM_001024401	chr16	28296206	28303385	chr16	28303840	28335170
cis_mRNA up10k	NA	NONHSAT141527.2	NM_148415	chr16	28831891	28833354	chr16	28834369	28848558
cis_mRNA up10k	NA	NONHSAT141599.2	NM_001015000	chr16	29454226	29465435	chr16	29465822	29469545
cis_mRNA up10k	NA	NONHSAT141599.2	NM_178044	chr16	29454226	29465435	chr16	29465822	29469545
tran	NA	NONHSAT141601.	NM_007074	chr16	29460666	29461236	chr16	30194731	30200397
cis_mRNA overlap	Lnc-Overlap-mRNA	NONHSAT141613.2	MTCONS_00073067	chr16	29535926	29542213	chr16	29535926	29625053
cis_mRNA dw20k	NA	NONHSAT141684.2	NM_016151	chr16	30013305	30015877	chr16	29985188	29999726
cis_mRNA dw20k	NA	NONHSAT141684.2	MTCONS_00069581	chr16	30013305	30015877	chr16	29985188	30003582
cis_mRNA overlap	Lnc-CompleteIn-	NONHSAT141684.2	NM_173618	chr16	30013305	30015877	chr16	30007530	30017111
cis_mRNA _overlap	Lnc-AntiOverlap-mRNA	NONHSAT141712.2	NM_001193333	chr16	30195383	30196278	chr16	30194731	30200397
cis_mRNA overlap	Lnc-Overlap-mRNA	NONHSAT141716.2	NM_007074	chr16	30200005	30200575	chr16	30194731	30200397
cis_mRNA _overlap	Lnc-AntiOverlap-mRNA	NONHSAT141763.2	NM_001305019	chr16	30546073	30548465	chr16	30541688	30546291
cis_mRNA up10k	NA	NONHSAT141763.2	NM_024671	chr16	30546073	30548465	chr16	30535322	30537910
tran	NA	NONHSAT141888.	MTCONS_0007428	chr16	31207452	31208284	chr16	74357188	74402046
tran	NA	NONHSAT142142.	MTCONS_0000596	chr16	34232106	34232469	chr1	160663577	160668201

cis_mRNA dw20k	NA	NONHSAT142582.2	NM_022476	chr16	53521519	53523856	chr16	53524952	53537216
cis_mRNA overlap	Lnc-CompleteIn-	NONHSAT142638.2	NM_017839	chr16	55572112	55575939	chr16	55542913	55620582
cis_mRNA dw20k	NA	NONHSAT142706.2	NM_005951	chr16	56716382	56721719	chr16	56703726	56705041
cis_mRNA overlap	Lnc-Overlap-mRNA	NONHSAT142706.2	NM_005952	chr16	56716382	56721719	chr16	56716382	56718108
tran	NA	NONHSAT142706.	MTCONS_0007040	chr16	56716382	56721719	chr16	56666534	56667898
cis_mRNA dw20k	NA	NONHSAT142708.2	NM_005951	chr16	56720763	56721964	chr16	56703726	56705041
cis_mRNA overlap	Lnc-Overlap-mRNA	NONHSAT142717.2	NM_001242796	chr16	56878629	56880792	chr16	56817413	56878861
cis_mRNA overlap	Lnc-CompleteIn-	NONHSAT142722.2	NM_014685	chr16	56974190	56975254	chr16	56966002	56977793
cis_mRNA overlap	Lnc-CompleteIn-	NONHSAT142723.2	NM_014685	chr16	56974940	56975638	chr16	56966002	56977793
tran	NA	NONHSAT142909.	NM_002954	chr16	61089303	61089868	chr2	55459541	55462989
cis_mRNA _overlap	Lnc-AntiOverlap-mRNA	NONHSAT143105.2	MTCONS_00073936	chr16	67270763	67272418	chr16	67263292	67281425
tran	NA	NONHSAT143382.	NM_001285448	chr16	70051820	70057521	chr16	15068660	15132187
cis_mRNA overlap	Lnc-Overlap-mRNA	NONHSAT143410.2	MTCONS_00074097	chr16	70253768	70258002	chr16	70253149	70285833
cis_mRNA dw20k	NA	NONHSAT143669.2	MTCONS_00074283	chr16	74339100	74344205	chr16	74357188	74402046
cis_mRNA dw20k	NA	NONHSAT143669.2	MTCONS_00074285	chr16	74339100	74344205	chr16	74363859	74402046
cis_mRNA overlap	Lnc-Overlap-mRNA	NONHSAT143672.2	MTCONS_00074283	chr16	74343998	74369244	chr16	74357188	74402046
cis_mRNA dw20k	NA	NONHSAT143673.2	MTCONS_00074285	chr16	74346507	74347288	chr16	74363859	74402046
cis_mRNA dw20k	NA	NONHSAT143673.2	MTCONS_00074283	chr16	74346507	74347288	chr16	74357188	74402046

cis_mRNA dw20k	NA	NONHSAT143674.2	MTCONS_00074285	chr16	74347204	74360683	chr16	74363859	74402046
cis_mRNA overlap	Lnc-Overlap-mRNA	NONHSAT143674.2	MTCONS_00074283	chr16	74347204	74360683	chr16	74357188	74402046
cis_mRNA overlap	Lnc-Overlap-mRNA	NONHSAT143681.2	MTCONS_00074270	chr16	74400933	74402046	chr16	74339007	74402046
cis_mRNA overlap	Lnc-Overlap-mRNA	NONHSAT143681.2	MTCONS_00074283	chr16	74400933	74402046	chr16	74357188	74402046
cis_mRNA overlap	Lnc-Overlap-mRNA	NONHSAT143681.2	MTCONS_00074285	chr16	74400933	74402046	chr16	74363859	74402046
tran	NA	NONHSAT143681.	MTCONS_0003070	chr16	74400933	74402046	chr11	67155110	67165883
tran	NA	NONHSAT143681.	NM_001145320	chr16	74400933	74402046	chr9	136397286	136440641
cis_mRNA_overlap	Lnc-AntiOverlap-mRNA	NONHSAT143739.2	NM_021615	chr16	75507023	75529305	chr16	75507022	75528926
cis_mRNA dw20k	NA	NONHSAT143903.2	NM_152342	chr16	80631803	80636416	chr16	80637676	80838175
cis_mRNA dw20k	NA	NONHSAT143944.2	NM_022041	chr16	81416874	81424489	chr16	81348571	81413803
tran	NA	NONHSAT144155.	MTCONS_0014000	chr16	85968887	85969829	chr3	197337808	197354755
cis_mRNA up10k	NA	NONHSAT144274.2	NM_001256917	chr16	87351111	87351640	chr16	87336404	87351026
cis_mRNA dw20k	NA	NONHSAT144315.2	MTCONS_00074794	chr16	87731754	87739290	chr16	87741418	87799598
cis_mRNA dw20k	NA	NONHSAT144545.2	NM_003119	chr16	89627330	89629512	chr16	89574796	89624176
tran	NA	NONHSAT144977.	NM_001013649	chr17	2574352	2577029	chr2	85832376	85839179
cis_mRNA dw20k	NA	NONHSAT145054.2	NM_016376	chr17	4064050	4065910	chr17	4066665	4167274
cis_mRNA_overlap	Lnc-AntiOverlap-mRNA	NONHSAT145409.2	NM_004870	chr17	7485282	7487390	chr17	7486965	7491527
cis_mRNA dw20k	NA	NONHSAT145411.2	NM_004860	chr17	7485431	7486964	chr17	7494548	7518215

cis_mRNA_overlap	Lnc-AntiCompleteIn-mRNAIntron	NONHSAT145609.2	NM_201433	chr17	10048831	10050277	chr17	9813926	10101868
cis_mRNA_overlap	Lnc-AntiOverlap-mRNA	NONHSAT145752.2	NM_001303	chr17	13932609	13972775	chr17	13972719	14111996
cis_mRNA_overlap	Lnc-CompleteIn-	NONHSAT145765.2	NM_006041	chr17	14207057	14209062	chr17	14204367	14252721
cis_mRNA_dw20k	NA	NONHSAT145966.2	MTCONS_00076288	chr17	16342659	16345056	chr17	16318856	16340326
cis_mRNA_dw20k	NA	NONHSAT146130.2	MTCONS_00080967	chr17	17906236	17909643	chr17	17920192	17942519
cis_mRNA_overlap	Lnc-AntiOverlap-mRNA	NONHSAT146155.2	NM_017758	chr17	18079879	18088067	chr17	18086867	18113267
tran	NA	NONHSAT146385.	NM_001031	chr17	18476045	18476415	chr19	8386384	8387280
cis_mRNA_dw20k	NA	NONHSAT146436.2	NM_001129778	chr17	19064102	19065587	chr17	19030782	19062148
cis_mRNA_dw20k	NA	NONHSAT146952.2	NM_024100	chr19	1005772	1009305	chr19	984328	994569
cis_mRNA_dw20k	NA	NONHSAT146952.2	NM_001033026	chr19	1005772	1009305	chr19	1009650	1021141
tran	NA	NONHSAT147573.	NM_001099415	chr7	72416689	72419500	chr7	75046060	75115565
cis_mRNA_up10k	NA	NONHSAT147590.2	NM_178238	chr7	99933746	99949834	chr7	99955626	99965454
cis_mRNA_overlap	Lnc-AntiOverlap-mRNA	NONHSAT147786.2	NM_002613	chr16	2611472	2615097	chr16	2587965	2653191
cis_mRNA_up10k	NA	NONHSAT147816.2	NM_018992	chr16	2708390	2723440	chr16	2732495	2759032
cis_mRNA_overlap	Lnc-AntiOverlap-mRNA	NONHSAT147841.2	NM_016333	chr16	2799787	2802519	chr16	2802330	2821413
cis_mRNA_dw20k	NA	NONHSAT147909.2	NM_001288665	chr16	3056586	3057374	chr16	3072621	3074287

cis_mRNA_up10k	NA	NONHSAT147927.2	NM_001012634	chr16	3101996	3109336	chr16	3115639	3119668
cis_mRNA_up10k	NA	NONHSAT147928.2	MTCONS_00068622	chr16	3102157	3106362	chr16	3106366	3110871
cis_mRNA_dw20k	NA	NONHSAT148065.2	NM_016292	chr16	3700637	3701704	chr16	3708038	3767598
cis_mRNA_up10k	NA	NONHSAT148065.2	NM_005223	chr16	3700637	3701704	chr16	3702940	3708096
cis_mRNA_dw20k	NA	NONHSAT148075.2	NM_005223	chr16	3710743	3715473	chr16	3702940	3708096
cis_mRNA_overlap	Lnc-AntiOverlap-mRNA	NONHSAT148075.2	NM_016292	chr16	3710743	3715473	chr16	3708038	3767598
cis_mRNA_dw20k	NA	NONHSAT148099.2	MTCONS_00072387	chr16	4294031	4303750	chr16	4307187	4323050
cis_mRNA_dw20k	NA	NONHSAT148099.2	NM_003223	chr16	4294031	4303750	chr16	4307187	4323001
cis_mRNA_up10k	NA	NONHSAT148102.2	NM_001098814	chr16	4295826	4303790	chr16	4239375	4292081
cis_mRNA_dw20k	NA	NONHSAT148160.2	NM_133450	chr16	4735831	4740970	chr16	4746511	4784163
cis_mRNA_dw20k	NA	NONHSAT148238.1	NM_007033	chr1	2346289	2353236	chr1	2323214	2336885
cis_mRNA_up10k	NA	NONHSAT148241.1	MTCONS_00000303	chr1	2477904	2478899	chr1	2487804	2490838
cis_mRNA_overlap	Lnc-AntiCompleteIn-mRNAIntron	NONHSAT148346.1	NM_014944	chr1	9830964	9832799	chr1	9789079	9884584
cis_mRNA_overlap	Lnc-AntiOverlap-mRNA	NONHSAT148376.1	NM_002685	chr1	11159733	11162158	chr1	11126670	11159967
cis_mRNA_overlap	Lnc-Overlap-mRNA	NONHSAT148392.1	MTCONS_00000736	chr1	11875632	11888976	chr1	11866153	11903201
tran	NA	NONHSAT148480.	MTCONS_0009682	chr1	20356772	20359646	chr19	52370214	52408305

cis_mRNA up10k	NA	NONHSAT148521.1	NM_030634	chr1	23695464	23698330	chr1	23685941	23694879
cis_mRNA overlap	Lnc-Overlap-mRNA	NONHSAT148554.1	MTCONS_00001332	chr1	25664995	25687477	chr1	25598893	25688852
cis_mRNA overlap	Lnc-CompleteIn-	NONHSAT148569.1	NM_002953	chr1	26897205	26897794	chr1	26856249	26901520
tran	NA	NONHSAT148601.	MTCONS_0019514	chr1	29295240	29295515	chr9	4792834	4890193
cis_mRNA overlap	Lnc-CompleteIn-	NONHSAT148662.1	NM_012316	chr1	32589442	32590610	chr1	32573644	32642168
cis_mRNA _overlap	Lnc-AntiOverlap-mRNA	NONHSAT148674.1	NM_018207	chr1	33628452	33631899	chr1	33611003	33647671
cis_mRNA _overlap	Lnc-AntiCompleteIn-mRNAIntron	NONHSAT148703.1	NM_024874	chr1	35977580	35981764	chr1	35899091	36023037
cis_mRNA overlap	Lnc-Overlap-mRNA	NONHSAT148739.1	NM_001142726	chr1	38273993	38275118	chr1	38273473	38275126
cis_mRNA overlap	Lnc-Overlap-mRNA	NONHSAT148739.1	NM_198446	chr1	38273993	38275118	chr1	38273473	38275126
cis_mRNA overlap	Lnc-CompleteIn-	NONHSAT148740.1	NM_198446	chr1	38273996	38275126	chr1	38273473	38275126
tran	NA	NONHSAT148768.	MTCONS_0018414	chr1	39442921	39443375	chr7	130146080	130372268
cis_mRNA overlap	Lnc-CompleteIn-	NONHSAT148876.1	NM_002482	chr1	46079180	46079500	chr1	46049660	46084578
cis_mRNA _overlap	Lnc-AntiOverlap-mRNA	NONHSAT148882.1	NM_005897	chr1	46162730	46165067	chr1	46164407	46216485
cis_mRNA dw20k	NA	NONHSAT149087.1	MTCONS_00003119	chr1	64012753	64016307	chr1	63988958	64012428
cis_mRNA overlap	Lnc-Overlap-mRNA	NONHSAT149103.1	NM_001003679	chr1	65886342	65898249	chr1	65886335	66101111
cis_mRNA overlap	Lnc-Overlap-mRNA	NONHSAT149103.1	NM_017526	chr1	65886342	65898249	chr1	65886131	65901690
cis_mRNA overlap	Lnc-Overlap-mRNA	NONHSAT149139.1	NM_001190987	chr1	70687153	70703140	chr1	70671365	70717701

cis_mRNA overlap	Lnc-Overlap-mRNA	NONHSAT149141.1	MTCONS_00003317	chr1	70710362	70717699	chr1	70671365	70717701
cis_mRNA overlap	Lnc-Overlap-mRNA	NONHSAT149141.1	MTCONS_00003319	chr1	70710362	70717699	chr1	70671365	70717701
cis_mRNA overlap	Lnc-Overlap-mRNA	NONHSAT149141.1	NM_001190987	chr1	70710362	70717699	chr1	70671365	70717701
cis_mRNA overlap	Lnc-Overlap-mRNA	NONHSAT149217.1	MTCONS_00003540	chr1	76255139	76260765	chr1	76251867	76261735
cis_mRNA _overlap	Lnc-AntiOverlap-mRNA	NONHSAT149231.1	MTCONS_00012067	chr1	78444878	78482938	chr1	78409743	78444976
cis_mRNA dw20k	NA	NONHSAT149273.1	NM_005274	chr1	84948646	84963359	chr1	84964006	84972262
cis_mRNA overlap	Lnc-CompleteIn-NA	NONHSAT149273.1	NM_025065	chr1	84948646	84963359	chr1	84944920	84964033
tran	NA	NONHSAT149274.	MTCONS_0003099	chr1	84995286	85252944	chr11	71927819	71933102
cis_mRNA up10k	NA	NONHSAT149275.1	NM_004388	chr1	85040233	85050574	chr1	85018804	85040163
cis_mRNA overlap	Lnc-Overlap-mRNA	NONHSAT149314.1	MTCONS_00003835	chr1	89273370	89280015	chr1	89149922	89303064
cis_mRNA up10k	NA	NONHSAT149322.1	MTCONS_00003870	chr1	90001692	90008050	chr1	90012164	90063601
cis_mRNA dw20k	NA	NONHSAT149356.1	NM_016040	chr1	93544873	93599279	chr1	93615299	93646246
cis_mRNA overlap	Lnc-CompleteIn-	NONHSAT149448.1	NM_017734	chr1	100111502	100152712	chr1	100111431	100160097
tran	NA	NONHSAT149450.	MTCONS_0011100	chr1	100262039	100262315	chr2	148687966	148778463
cis_mRNA dw20k	NA	NONHSAT149547.1	MTCONS_00004562	chr1	110912776	110915625	chr1	110881945	110903905
cis_mRNA _overlap	Lnc-AntiOverlap-mRNA	NONHSAT149589.1	NM_018372	chr1	111486090	111495471	chr1	111489812	111506566
cis_mRNA _overlap	Lnc-AntiOverlap-mRNA	NONHSAT149590.1	NM_001006945	chr1	111505924	111507271	chr1	111489812	111506566

cis_mRNA overlap	Lnc-Overlap-mRNA	NONHSAT149597.1	MTCONS_00004678	chr1	112251763	112255661	chr1	112162405	112259317
cis_mRNA overlap	Lnc-Overlap-mRNA	NONHSAT149597.1	MTCONS_00004681	chr1	112251763	112255661	chr1	112162657	112259317
tran	NA	NONHSAT149749.	NM_001317918	chr1	147893933	147908049	chr8	124780679	124822901
cis_mRNA dw20k	NA	NONHSAT149773.1	MTCONS_00013505	chr1	149817383	149818053	chr1	149828245	149832807
cis_mRNA up10k	NA	NONHSAT149773.1	NM_001040874	chr1	149817383	149818053	chr1	149813785	149814318
cis_mRNA up10k	NA	NONHSAT149773.1	NM_003516	chr1	149817383	149818053	chr1	149822628	149823161
cis_mRNA dw20k	NA	NONHSAT149774.1	NM_003516	chr1	149832837	149834070	chr1	149822628	149823161
cis_mRNA up10k	NA	NONHSAT149774.1	MTCONS_00013505	chr1	149832837	149834070	chr1	149828245	149832807
cis_mRNA dw20k	NA	NONHSAT149788.1	NM_025008	chr1	150531459	150533401	chr1	150521845	150531224
tran	NA	NONHSAT149802.	NM_001060	chr1	151289974	151291462	chr19	3594504	3606831
tran	NA	NONHSAT149802.	NM_001242547	chr1	151289974	151291462	chr10	73975758	73995618
tran	NA	NONHSAT149802.	NM_052859	chr1	151289974	151291462	chr3	53122501	53164470
tran	NA	NONHSAT149802.	NM_145298	chr1	151289974	151291462	chr22	39436673	39451975
cis_mRNA overlap	Lnc-Overlap-mRNA	NONHSAT149850.1	NM_001030	chr1	153963256	153964619	chr1	153963239	153964631
cis_mRNA up10k	NA	NONHSAT149874.1	NM_001162383	chr1	155948590	155952777	chr1	155916630	155948336
cis_mRNA_overlap	Lnc-AntiOverlap-mRNA	NONHSAT149883.1	MTCONS_00014113	chr1	156470302	156472268	chr1	156432660	156471490
cis_mRNA dw20k	NA	NONHSAT149961.1	MTCONS_00014423	chr1	161496264	161497073	chr1	161511551	161601252
cis_mRNA overlap	Lnc-Overlap-mRNA	NONHSAT150041.1	NM_015172	chr1	171454691	171484934	chr1	171454666	171562650
tran	NA	NONHSAT150052.	MTCONS_0018041	chr1	172136224	172136458	chr7	155437203	155480457
cis_mRNA overlap	Lnc-CompleteIn-	NONHSAT150104.1	MTCONS_00006547	chr1	178794733	178802277	chr1	178694282	178890977

cis_mRNA overlap	Lnc-CompleteIn-	NONHSAT150104.1	NM_152663	chr1	178794733	178802277	chr1	178694282	178890977
cis_mRNA overlap	Lnc-Overlap-mRNA	NONHSAT150105.1	NM_152663	chr1	178862891	178867965	chr1	178694282	178890977
cis_mRNA overlap	Lnc-CompleteIn-	NONHSAT150159.1	NM_030806	chr1	184559874	184589459	chr1	184356150	184598155
cis_mRNA up10k	NA	NONHSAT150184.1	MTCONS_00015157	chr1	186649754	186650578	chr1	186640944	186649559
cis_mRNA up10k	NA	NONHSAT150184.1	MTCONS_00015158	chr1	186649754	186650578	chr1	186640944	186649559
cis_mRNA up10k	NA	NONHSAT150184.1	MTCONS_00015160	chr1	186649754	186650578	chr1	186640944	186649559
cis_mRNA up10k	NA	NONHSAT150184.1	MTCONS_00015161	chr1	186649754	186650578	chr1	186640944	186649559
cis_mRNA overlap	Lnc-CompleteIn-	NONHSAT150320.1	NM_006335	chr1	201926639	201939477	chr1	201924619	201939789
cis_mRNA dw20k	NA	NONHSAT150335.1	NM_002023	chr1	203289528	203302911	chr1	203309749	203320557
cis_mRNA overlap	Lnc-Overlap-mRNA	NONHSAT150467.1	NM_001199756	chr1	212458821	212506895	chr1	212475148	212535205
cis_mRNA overlap	Lnc-Overlap-mRNA	NONHSAT150467.1	NM_006243	chr1	212458821	212506895	chr1	212458879	212535205
cis_mRNA dw20k	NA	NONHSAT150699.1	NM_014777	chr1	229801273	229805818	chr1	229761963	229795947
cis_mRNA up10k	NA	NONHSAT150724.1	NM_001164537	chr1	231753699	231753933	chr1	231762561	232177019
cis_mRNA dw20k	NA	NONHSAT150805.1	MTCONS_00008489	chr1	235512716	235516213	chr1	235491870	235507844
cis_mRNA overlap	Lnc-Overlap-mRNA	NONHSAT150859.1	NM_006642	chr1	243542012	243548698	chr1	243419307	243663393
cis_mRNA _overlap	mRNA-CompleteIn-LncExon	NONHSAT150910.1	MTCONS_00008890	chr1	14407	29370	chr1	14407	29370
cis_mRNA overlap	Lnc-Overlap-mRNA	NONHSAT150949.1	NM_021170	chr1	934346	935465	chr1	934342	935552

cis_mRNA dw20k	NA	NONHSAT150967.1	NM_001039577	chr1	1321884	1325635	chr1	1327668	1334718
cis_mRNA_overlap	Lnc-AntiOverlap-mRNA	NONHSAT150995.1	MTCONS_00000303	chr1	2481360	2488471	chr1	2487804	2490838
cis_mRNA overlap	Lnc-CompleteIn-	NONHSAT151018.1	NM_014704	chr1	3756274	3773782	chr1	3728645	3773797
cis_mRNA overlap	Lnc-Overlap-mRNA	NONHSAT151071.1	NM_018948	chr1	8073654	8086323	chr1	8071779	8086393
cis_mRNA_overlap	Lnc-AntiCompleteIn-mRNAIntron	NONHSAT151095.1	MTCONS_00000630	chr1	10293790	10295931	chr1	10270604	10441759
cis_mRNA dw20k	NA	NONHSAT151162.1	NM_052929	chr1	15729475	15735929	chr1	15573768	15724622
cis_mRNA dw20k	NA	NONHSAT151209.1	NM_020765	chr1	19378932	19386621	chr1	19401000	19536746
cis_mRNA dw20k	NA	NONHSAT151211.1	NM_020765	chr1	19378932	19386621	chr1	19401000	19536746
cis_mRNA overlap	Lnc-CompleteIn-	NONHSAT151255.1	NM_001142546	chr1	23480328	23489611	chr1	23410516	23495351
cis_mRNA overlap	Lnc-Overlap-mRNA	NONHSAT151376.1	MTCONS_00010602	chr1	32373849	32403919	chr1	32372022	32398349
cis_mRNA overlap	Lnc-Overlap-mRNA	NONHSAT151378.1	NM_080391	chr1	32374277	32403811	chr1	32372022	32403988
cis_mRNA_overlap	Lnc-AntiCompleteIn-mRNAIntron	NONHSAT151383.1	NM_012316	chr1	32587382	32588851	chr1	32573644	32642168
tran	NA	NONHSAT151482.	MTCONS_0009437	chr1	40319056	40354242	chr19	11487649	11495018
tran	NA	NONHSAT151539.	MTCONS_0011279	chr1	46515722	46516517	chr2	208470297	208490028
tran	NA	NONHSAT151550.	MTCONS_0009079	chr1	47632037	47633802	chr19	20946830	20993757
cis_mRNA dw20k	NA	NONHSAT151558.1	NM_001290403	chr1	47670392	47672900	chr1	47681962	47698007
cis_mRNA overlap	Lnc-CompleteIn-	NONHSAT151622.1	NM_001305043	chr1	54497356	54518889	chr1	54492354	54519111

cis_mRNA overlap	Lnc-Overlap-mRNA	NONHSAT151622.1	MTCONS_00011586	chr1	54497356	54518889	chr1	54492354	54519246
cis_mRNA overlap	Lnc-Overlap-mRNA	NONHSAT151803.1	MTCONS_00012067	chr1	78409760	78430656	chr1	78409743	78444976
cis_mRNA overlap	Lnc-CompleteIn-	NONHSAT151804.1	MTCONS_00012067	chr1	78414301	78444706	chr1	78409743	78444976
cis_mRNA overlap	Lnc-CompleteIn-	NONHSAT151869.1	NM_004261	chr1	87328409	87379782	chr1	87328128	87380107
cis_mRNA up10k	NA	NONHSAT151869.1	NM_012262	chr1	87328409	87379782	chr1	87380335	87575681
cis_mRNA overlap	Lnc-CompleteIn-	NONHSAT151941.1	NM_016040	chr1	93625711	93646209	chr1	93615299	93646246
cis_mRNA overlap	Lnc-CompleteIn-	NONHSAT152012.1	NM_001261441	chr1	101355644	101355904	chr1	101337928	101360735
cis_mRNA dw20k	NA	NONHSAT152082.1	NM_002232	chr1	111201609	111214145	chr1	111215074	111217655
cis_mRNA overlap	Lnc-Overlap-mRNA	NONHSAT152085.1	NM_178454	chr1	111660541	111682693	chr1	111659954	111682838
cis_mRNA overlap	Lnc-Overlap-mRNA	NONHSAT152086.1	NM_178454	chr1	111660541	111682744	chr1	111659954	111682838
cis_mRNA dw20k	NA	NONHSAT152088.1	NM_024901	chr1	111727037	111727683	chr1	111728591	111743325
cis_mRNA dw20k	NA	NONHSAT152089.1	NM_006090	chr1	111729796	111730925	chr1	111682833	111727724
cis_mRNA dw20k	NA	NONHSAT152089.1	MTCONS_00012994	chr1	111729796	111730925	chr1	111738227	111747160
cis_mRNA overlap	Lnc-CompleteIn-	NONHSAT152089.1	NM_024901	chr1	111729796	111730925	chr1	111728591	111743325
cis_mRNA overlap	Lnc-Overlap-mRNA	NONHSAT152110.1	NM_001308264	chr1	113153098	113161721	chr1	113066141	113161761
cis_mRNA up10k	NA	NONHSAT152175.1	NM_152380	chr1	119536212	119540066	chr1	119425666	119532179
cis_mRNA dw20k	NA	NONHSAT152224.1	NM_006468	chr1	145611052	145688338	chr1	145590666	145611043

cis_mRNA_overlap	Lnc-AntiCompleteIn-mRNAIntron	NONHSAT152234.1	NM_001037675	chr1	145209136	145282083	chr1	144823812	148346929
cis_mRNA_up10k	NA	NONHSAT152280.1	NM_005850	chr1	149900550	149901797	chr1	149895209	149900144
cis_mRNA_overlap	mRNA-CompleteIn-LncExon	NONHSAT152289.1	MTCONS_00013533	chr1	150237799	150241609	chr1	150237799	150241609
cis_mRNA_overlap	Lnc-Overlap-mRNA	NONHSAT152297.1	MTCONS_00013581	chr1	150704861	150720356	chr1	150687526	150738433
cis_mRNA_dw20k	NA	NONHSAT152298.1	NM_001040217	chr1	150965686	150967682	chr1	150969301	150979385
cis_mRNA_overlap	Lnc-AntiOverlap-mRNA	NONHSAT152305.1	NM_030918	chr1	151584514	151585839	chr1	151584662	151671559
cis_mRNA_overlap	Lnc-CompleteIn-	NONHSAT152334.1	NM_006694	chr1	153946779	153950123	chr1	153946745	153950451
cis_mRNA_overlap	Lnc-Overlap-mRNA	NONHSAT152335.1	NM_006694	chr1	153946986	153950112	chr1	153946745	153950451
cis_mRNA_overlap	Lnc-Overlap-mRNA	NONHSAT152340.1	NM_006694	chr1	153946993	153950142	chr1	153946745	153950451
cis_mRNA_overlap	Lnc-Overlap-mRNA	NONHSAT152402.1	NM_001303095	chr1	156697062	156698529	chr1	156691683	156698231
tran	NA	NONHSAT152488.	MTCONS_0013211	chr1	166636691	166658518	chr3	122296449	122357894
tran	NA	NONHSAT152488.	MTCONS_0018041	chr1	166636691	166658518	chr7	155437203	155480457
cis_mRNA_dw20k	NA	NONHSAT152562.1	NM_018122	chr1	173833172	173836867	chr1	173793797	173827682
cis_mRNA_overlap	Lnc-Overlap-mRNA	NONHSAT152606.1	MTCONS_00014978	chr1	180238789	180244398	chr1	180237858	180472022
cis_mRNA_dw20k	NA	NONHSAT152617.1	NM_016545	chr1	181064285	181074485	chr1	181057638	181059979
cis_mRNA_dw20k	NA	NONHSAT152692.1	NM_001173524	chr1	193059996	193060686	chr1	193028552	193055115

cis_mRNA_overlap	Lnc-AntiOverlap-mRNA	NONHSAT152692.1	MTCONS_00006928	chr1	193059996	193060686	chr1	193028552	193059999
tran	NA	NONHSAT152771.	MTCONS_0003070	chr1	203849519	203849882	chr11	67155110	67165883
cis_mRNA_overlap	Lnc-Overlap-mRNA	NONHSAT152882.1	MTCONS_00015904	chr1	213181773	213188838	chr1	213165524	213188851
cis_mRNA_overlap	Lnc-Overlap-mRNA	NONHSAT153023.1	NM_001031685	chr1	223993751	224033597	chr1	223967595	224033674
cis_mRNA_overlap	Lnc-Overlap-mRNA	NONHSAT153043.1	MTCONS_00016306	chr1	226028596	226036698	chr1	226033233	226070420
cis_mRNA_dw20k	NA	NONHSAT153064.1	NM_001024227	chr1	228288440	228289840	chr1	228270851	228286913
cis_mRNA_overlap	Lnc-CompleteIn-	NONHSAT153064.1	NM_024319	chr1	228288440	228289840	chr1	228288428	228291163
cis_mRNA_dw20k	NA	NONHSAT153065.1	NM_001024227	chr1	228295369	228296206	chr1	228270851	228286913
cis_mRNA_up10k	NA	NONHSAT153065.1	NM_024319	chr1	228295369	228296206	chr1	228288428	228291163
cis_mRNA_overlap	Lnc-Overlap-mRNA	NONHSAT153105.1	MTCONS_00016522	chr1	231055046	231059781	chr1	231041987	231114618
tran	NA	NONHSAT153254.	MTCONS_0016852	chr1	243077153	243077520	chr6	2877747	2903546
cis_mRNA_dw20k	NA	NONHSAT153306.1	NM_030645	chr1	249095502	249103104	chr1	249104651	249120154
cis_mRNA_dw20k	NA	NONHSAT153653.1	MTCONS_00011348	chr1	47665014	47667225	chr1	47681962	47697456
cis_mRNA_up10k	NA	NONHSAT153924.1	NM_001006605	chr1	93427459	93428659	chr1	93307717	93427079
cis_mRNA_up10k	NA	NONHSAT154027.1	NM_000757	chr1	110448449	110450008	chr1	110453233	110473616
cis_mRNA_dw20k	NA	NONHSAT154125.1	MTCONS_00005222	chr1	150451671	150452360	chr1	150336587	150433447
cis_mRNA_up10k	NA	NONHSAT154148.1	NM_012437	chr1	153621431	153623500	chr1	153631130	153634328
cis_mRNA_dw20k	NA	NONHSAT154163.1	MTCONS_00014113	chr1	156427898	156428731	chr1	156432660	156471490

cis_mRNA dw20k	NA	NONHSAT154304.1	NM_016545	chr1	181063036	181063722	chr1	181057638	181059979
cis_mRNA dw20k	NA	NONHSAT154883.1	NM_014023	chr10	1192594	1199051	chr10	1102776	1178237
cis_mRNA up10k	NA	NONHSAT155010.1	NM_001171864	chr10	5454670	5473587	chr10	5435061	5446793
cis_mRNA overlap	Lnc-CompleteIn-	NONHSAT155186.1	NM_032812	chr10	20506487	20569114	chr10	20105372	20578784
cis_mRNA overlap	Lnc-Overlap-mRNA	NONHSAT155313.1	MTCONS_00019642	chr10	30726147	30726487	chr10	30722950	30750762
cis_mRNA _overlap	Lnc-AntiCompleteIn-mRNAIntron	NONHSAT155341.1	NM_001282391	chr10	32635428	32636108	chr10	32556644	32667726
cis_mRNA overlap	Lnc-Overlap-mRNA	NONHSAT155481.1	MTCONS_00020217	chr10	49999890	50029596	chr10	49892848	50191007
cis_mRNA _overlap	Lnc-AntiCompleteIn-mRNAIntron	NONHSAT155594.1	MTCONS_00025037	chr10	69762266	69763205	chr10	69681656	69835103
cis_mRNA overlap	Lnc-Overlap-mRNA	NONHSAT155864.1	MTCONS_00021518	chr10	92631774	92665404	chr10	92631709	92661947
cis_mRNA dw20k	NA	NONHSAT155925.1	NM_021732	chr10	99425508	99433845	chr10	99437181	99447015
cis_mRNA overlap	Lnc-Overlap-mRNA	NONHSAT155925.1	NM_018425	chr10	99425508	99433845	chr10	99400443	99436189
cis_mRNA overlap	Lnc-Overlap-mRNA	NONHSAT156099.1	NM_014456	chr10	112631720	112636837	chr10	112631553	112659764
cis_mRNA overlap	Lnc-Overlap-mRNA	NONHSAT156099.1	NM_145341	chr10	112631720	112636837	chr10	112631553	112659764
cis_mRNA _overlap	Lnc-AntiOverlap-mRNA	NONHSAT156154.1	MTCONS_00026519	chr10	116215590	116225594	chr10	116190429	116286685
cis_mRNA _overlap	Lnc-AntiOverlap-mRNA	NONHSAT156307.1	NM_022802	chr10	126684633	126690060	chr10	126676418	126716453

cis_mRNA overlap	Lnc-CompleteIn-	NONHSAT156388.1	MTCONS_00022942	chr10	132011023	132050308	chr10	131934639	132082436
cis_mRNA overlap	Lnc-Overlap-mRNA	NONHSAT156441.1	NM_138499	chr10	134219389	134231365	chr10	134210702	134231358
tran	NA	NONHSAT156566.	MTCONS_0011100	chr10	5186655	5192149	chr2	148687966	148778463
cis_mRNA overlap	Lnc-Overlap-mRNA	NONHSAT156722.1	NM_001081	chr10	17053529	17061966	chr10	16865965	17171816
cis_mRNA _overlap	Lnc-AntiOverlap-mRNA	NONHSAT156726.1	NM_003380	chr10	17256238	17271984	chr10	17270258	17279592
tran	NA	NONHSAT156862.	MTCONS_0009079	chr10	32742331	32775044	chr19	20946830	20993757
cis_mRNA overlap	Lnc-CompleteIn-	NONHSAT156969.1	MTCONS_00020264	chr10	51585540	51590733	chr10	51565201	51590733
cis_mRNA overlap	Lnc-Overlap-mRNA	NONHSAT157099.1	MTCONS_00025037	chr10	69747118	69750213	chr10	69681656	69835103
cis_mRNA overlap	Lnc-CompleteIn-	NONHSAT157100.1	MTCONS_00025037	chr10	69769704	69770640	chr10	69681656	69835103
cis_mRNA overlap	Lnc-CompleteIn-	NONHSAT157108.1	MTCONS_00025070	chr10	70237756	70240522	chr10	70236960	70287280
cis_mRNA overlap	Lnc-CompleteIn-	NONHSAT157133.1	NM_022153	chr10	73521943	73525455	chr10	73507313	73533337
cis_mRNA overlap	Lnc-Overlap-mRNA	NONHSAT157164.1	NM_144589	chr10	76993730	76995682	chr10	76993729	76995770
cis_mRNA dw20k	NA	NONHSAT157165.1	NM_001184823	chr10	76993737	76995705	chr10	76969912	76991206
cis_mRNA overlap	Lnc-Overlap-mRNA	NONHSAT157428.1	NM_012215	chr10	103572369	103578172	chr10	103544200	103578222
cis_mRNA overlap	Lnc-CompleteIn-	NONHSAT157445.1	NM_000494	chr10	105791267	105793119	chr10	105791046	105845638
cis_mRNA _overlap	Lnc-AntiOverlap-mRNA	NONHSAT157517.1	MTCONS_00022336	chr10	114710406	114711635	chr10	114710009	114915765
cis_mRNA up10k	NA	NONHSAT157766.1	NM_138499	chr10	134208072	134210489	chr10	134210702	134231358

cis_mRNA dw20k	NA	NONHSAT157774.1	NM_005539	chr10	134607461	134628918	chr10	134351353	134596984
cis_mRNA up10k	NA	NONHSAT157829.1	MTCONS_00023292	chr10	3827633	3830102	chr10	3818188	3827473
cis_mRNA up10k	NA	NONHSAT157829.1	MTCONS_00023293	chr10	3827633	3830102	chr10	3818188	3827473
cis_mRNA up10k	NA	NONHSAT158131.1	NM_174890	chr10	46173126	46173758	chr10	46110949	46168180
cis_mRNA dw20k	NA	NONHSAT158685.1	NM_006435	chr11	314056	315262	chr11	308107	309410
cis_mRNA overlap	Lnc-CompleteIn-	NONHSAT158685.1	NM_003641	chr11	314056	315262	chr11	313991	315272
cis_mRNA dw20k	NA	NONHSAT158707.1	NM_019009	chr11	1287575	1288512	chr11	1295598	1330892
cis_mRNA overlap	Lnc-CompleteIn-	NONHSAT159015.1	NM_001202557	chr11	35222407	35222647	chr11	35160417	35244867
tran	NA	NONHSAT159022.	MTCONS_0018041	chr11	35792458	35796132	chr7	155437203	155480457
cis_mRNA overlap	Lnc-Overlap-mRNA	NONHSAT159107.1	MTCONS_00029632	chr11	46365506	46392640	chr11	46366817	46402104
cis_mRNA dw20k	NA	NONHSAT159115.1	NM_001143984	chr11	47599277	47599829	chr11	47586982	47595013
cis_mRNA dw20k	NA	NONHSAT159169.1	NM_015533	chr11	61119716	61121949	chr11	61100654	61116231
cis_mRNA dw20k	NA	NONHSAT159178.1	MTCONS_00030122	chr11	61735266	61736962	chr11	61717302	61732844
cis_mRNA dw20k	NA	NONHSAT159178.1	MTCONS_00030129	chr11	61735266	61736962	chr11	61722760	61732844
cis_mRNA up10k	NA	NONHSAT159185.1	NM_001085372	chr11	62432905	62433425	chr11	62439126	62441162
cis_mRNA up10k	NA	NONHSAT159207.1	NM_001135208	chr11	63998097	64001752	chr11	64008604	64011607
cis_mRNA dw20k	NA	NONHSAT159210.1	NM_031471	chr11	64003359	64006261	chr11	63974152	63991363
cis_mRNA dw20k	NA	NONHSAT159210.1	NM_005528	chr11	64003359	64006261	chr11	63997753	64001753

cis_mRNA up10k	NA	NONHSAT159210.1	MTCONS_00035026	chr11	64003359	64006261	chr11	63991271	63993726
cis_mRNA overlap	Lnc-CompleteIn-	NONHSAT159226.1	MTCONS_00030414	chr11	64966921	64969518	chr11	64949150	64981116
cis_mRNA up10k	NA	NONHSAT159258.1	NM_001135635	chr11	65686933	65689015	chr11	65684283	65686531
cis_mRNA up10k	NA	NONHSAT159290.1	NM_012308	chr11	66884134	66886262	chr11	66886740	67025550
cis_mRNA overlap	Lnc-CompleteIn-	NONHSAT159480.1	MTCONS_00031411	chr11	82885614	82887878	chr11	82868137	82896835
tran	NA	NONHSAT159480.	MTCONS_0018041	chr11	82885614	82887878	chr7	155437203	155480457
cis_mRNA overlap	Lnc-Overlap-mRNA	NONHSAT159497.1	NM_007173	chr11	86511661	86662873	chr11	86511282	86522273
cis_mRNA _overlap	Lnc-AntiOverlap-mRNA	NONHSAT159521.1	NM_012124	chr11	89956321	89959706	chr11	89933598	89956532
tran	NA	NONHSAT159570.	NM_024884	chr11	96774842	96912016	chr14	50709152	50778947
cis_mRNA overlap	Lnc-Overlap-mRNA	NONHSAT159592.1	MTCONS_00031796	chr11	102206765	102208451	chr11	102188181	102215003
cis_mRNA up10k	NA	NONHSAT159592.1	NM_001166	chr11	102206765	102208451	chr11	102217913	102249401
cis_mRNA overlap	Lnc-CompleteIn-	NONHSAT159677.1	NM_006006	chr11	114083320	114084251	chr11	113930431	114121397
cis_mRNA overlap	Lnc-Overlap-mRNA	NONHSAT159680.1	NM_001286045	chr11	114272424	114279633	chr11	114271251	114281332
cis_mRNA _overlap	Lnc-AntiOverlap-mRNA	NONHSAT159734.1	NM_012104	chr11	117160214	117164162	chr11	117156402	117186972
cis_mRNA _overlap	Lnc-AntiOverlap-mRNA	NONHSAT159736.1	NM_012104	chr11	117163514	117164287	chr11	117156402	117186972
cis_mRNA dw20k	NA	NONHSAT159749.1	MTCONS_00032307	chr11	118881129	118885790	chr11	118868843	118873672
cis_mRNA overlap	Lnc-CompleteIn-	NONHSAT159896.1	NM_001243299	chr11	130013492	130014699	chr11	129940463	130014706

cis_mRNA overlap	Lnc-Overlap-mRNA	NONHSAT159977.1	NM_173573	chr11	555660	558420	chr11	554850	560779
cis_mRNA overlap	Lnc-Overlap-mRNA	NONHSAT159980.1	NM_004031	chr11	612556	615193	chr11	612555	615728
cis_mRNA overlap	Lnc-Overlap-mRNA	NONHSAT160022.1	NM_001122630	chr11	2904871	2905470	chr11	2904448	2906995
cis_mRNA dw20k	NA	NONHSAT160046.1	NM_000391	chr11	6624873	6625651	chr11	6633997	6640692
tran	NA	NONHSAT160102.	MTCONS_0009079	chr11	14866730	14867081	chr19	20946830	20993757
cis_mRNA dw20k	NA	NONHSAT160210.1	NM_001033506	chr11	33161864	33162104	chr11	33162977	33183037
tran	NA	NONHSAT160229.	NM_020702	chr11	35069118	35075290	chr9	34366664	34376894
tran	NA	NONHSAT160348.	MTCONS_0009079	chr11	59084351	59089182	chr19	20946830	20993757
cis_mRNA dw20k	NA	NONHSAT160364.1	NM_001254750	chr11	60798969	60810737	chr11	60739113	60787848
cis_mRNA dw20k	NA	NONHSAT160367.1	NM_015533	chr11	61116282	61118552	chr11	61100654	61116231
cis_mRNA _overlap	Lnc-AntiCompleteIn-mRNAIntron	NONHSAT160419.1	MTCONS_00030399	chr11	64869274	64874222	chr11	64863587	64883707
cis_mRNA up10k	NA	NONHSAT160422.1	MTCONS_00035153	chr11	64947492	64949305	chr11	64941577	64943013
cis_mRNA overlap	Lnc-Overlap-mRNA	NONHSAT160431.1	NM_001130144	chr11	65317065	65318936	chr11	65306030	65325699
cis_mRNA overlap	Lnc-Overlap-mRNA	NONHSAT160436.1	NM_005507	chr11	65622288	65627637	chr11	65622285	65625804
cis_mRNA overlap	Lnc-Overlap-mRNA	NONHSAT160438.1	NM_001242486	chr11	65768900	65772094	chr11	65764016	65769637
cis_mRNA overlap	Lnc-CompleteIn-	NONHSAT160572.1	NM_005040	chr11	82535413	82561442	chr11	82533983	82611645
cis_mRNA overlap	Lnc-CompleteIn-	NONHSAT160586.1	MTCONS_00035987	chr11	85455444	85456066	chr11	85405264	85522202
cis_mRNA overlap	Lnc-Overlap-mRNA	NONHSAT160589.1	MTCONS_00035998	chr11	85722160	85780133	chr11	85668214	85780139

cis_mRNA overlap	Lnc-CompleteIn-	NONHSAT160605.1	NM_148170	chr11	88059260	88068125	chr11	88053979	88070955
cis_mRNA overlap	Lnc-Overlap-mRNA	NONHSAT160605.1	NM_001814	chr11	88059260	88068125	chr11	88026760	88070955
cis_mRNA dw20k	NA	NONHSAT160633.1	NM_024116	chr11	93467906	93468826	chr11	93469097	93474703
cis_mRNA overlap	Lnc-CompleteIn-	NONHSAT160633.1	MTCONS_00036160	chr11	93467906	93468826	chr11	93463369	93474703
tran	NA	NONHSAT160695.	MTCONS_0018041	chr11	106049963	106054336	chr7	155437203	155480457
cis_mRNA overlap	Lnc-CompleteIn-	NONHSAT160760.1	NM_001098517	chr11	115133379	115136555	chr11	115044345	115375241
cis_mRNA _overlap	Lnc-AntiCompleteIn-mRNAIntron	NONHSAT160834.1	NM_003105	chr11	121471818	121474271	chr11	121322912	121504471
cis_mRNA up10k	NA	NONHSAT160853.1	MTCONS_00032505	chr11	123292533	123299872	chr11	123299955	123498479
cis_mRNA up10k	NA	NONHSAT160988.1	MTCONS_00033242	chr11	3083056	3089526	chr11	3022152	3078681
cis_mRNA dw20k	NA	NONHSAT161246.1	MTCONS_00030672	chr11	67031466	67033845	chr11	67007506	67025550
cis_mRNA up10k	NA	NONHSAT161251.1	MTCONS_00035417	chr11	67981452	67983462	chr11	67922330	67981326
cis_mRNA overlap	Lnc-Overlap-mRNA	NONHSAT161726.1	NM_024551	chr12	1863423	1879573	chr12	1800247	1897845
cis_mRNA dw20k	NA	NONHSAT161799.1	NM_001135217	chr12	7037123	7043722	chr12	7013897	7023406
cis_mRNA up10k	NA	NONHSAT161836.1	MTCONS_00038790	chr12	9811164	9814682	chr12	9822304	9852151
cis_mRNA overlap	Lnc-Overlap-mRNA	NONHSAT161837.1	MTCONS_00038790	chr12	9816426	9833562	chr12	9822304	9852151
cis_mRNA overlap	Lnc-Overlap-mRNA	NONHSAT161846.1	NM_002262	chr12	10460489	10467600	chr12	10460417	10469850
cis_mRNA overlap	Lnc-Overlap-mRNA	NONHSAT161846.1	NM_007334	chr12	10460489	10467600	chr12	10460417	10469850

cis_mRNA overlap	Lnc-CompleteIn-	NONHSAT161865.1	NM_004064	chr12	12871907	12872455	chr12	12870204	12875316
cis_mRNA up10k	NA	NONHSAT161866.1	NM_016355	chr12	12959718	12962132	chr12	12966280	12982915
cis_mRNA overlap	Lnc-CompleteIn-	NONHSAT161934.1	NM_153207	chr12	19592841	19646880	chr12	19592608	19675173
cis_mRNA overlap	Lnc-Overlap-mRNA	NONHSAT162254.1	NM_021104	chr12	56510431	56511613	chr12	56510374	56511616
cis_mRNA up10k	NA	NONHSAT162268.1	NM_003708	chr12	57359550	57368635	chr12	57345215	57353158
tran	NA	NONHSAT162268.	MTCONS_0009079	chr12	57359550	57368635	chr19	20946830	20993757
tran	NA	NONHSAT162268.	NM_001164436	chr12	57359550	57368635	chr3	171561139	171577108
cis_mRNA up10k	NA	NONHSAT162282.1	NM_001195057	chr12	57921735	57923931	chr12	57910371	57914300
cis_mRNA dw20k	NA	NONHSAT162362.1	NM_018448	chr12	67708478	67713730	chr12	67663061	67708472
tran	NA	NONHSAT162370.	MTCONS_0001226	chr12	67944589	67945076	chr1	86815777	86862025
cis_mRNA overlap	Lnc-Overlap-mRNA	NONHSAT162423.1	NM_014999	chr12	72163440	72165755	chr12	72148643	72187256
cis_mRNA dw20k	NA	NONHSAT162918.1	NM_001304833	chr12	123465270	123467454	chr12	123459750	123464588
cis_mRNA overlap	Lnc-Overlap-mRNA	NONHSAT162918.1	NM_001278379	chr12	123465270	123467454	chr12	123464607	123467460
cis_mRNA overlap	Lnc-Overlap-mRNA	NONHSAT162918.1	NM_001278380	chr12	123465270	123467454	chr12	123464607	123467460
cis_mRNA overlap	Lnc-Overlap-mRNA	NONHSAT162918.1	NM_016638	chr12	123465270	123467454	chr12	123464607	123467460
cis_mRNA up10k	NA	NONHSAT162946.1	MTCONS_00047258	chr12	125052322	125053226	chr12	124903786	125052010
cis_mRNA overlap	Lnc-Overlap-mRNA	NONHSAT163238.1	MTCONS_00043401	chr12	6628992	6663237	chr12	6648694	6665249
cis_mRNA dw20k	NA	NONHSAT163279.1	NM_004426	chr12	9099453	9101926	chr12	9067316	9094060
tran	NA	NONHSAT163307.	MTCONS_0003070	chr12	10998482	11049352	chr11	67155110	67165883

cis_mRNA up10k	NA	NONHSAT163320.1	NM_058169	chr12	12508340	12510001	chr12	12510013	12619838
cis_mRNA overlap	Lnc-Overlap-mRNA	NONHSAT163493.1	NM_001278390	chr12	46623370	46662773	chr12	46589508	46661770
cis_mRNA overlap	Lnc-Overlap-mRNA	NONHSAT163497.1	MTCONS_00044522	chr12	46764157	46766546	chr12	46751971	46766645
cis_mRNA overlap	Lnc-Overlap-mRNA	NONHSAT163497.1	MTCONS_00044524	chr12	46764157	46766546	chr12	46751971	46766645
cis_mRNA overlap	Lnc-Overlap-mRNA	NONHSAT163497.1	MTCONS_00044525	chr12	46764157	46766546	chr12	46751971	46766645
cis_mRNA overlap	Lnc-Overlap-mRNA	NONHSAT163497.1	NM_018976	chr12	46764157	46766546	chr12	46751971	46766645
tran	NA	NONHSAT163594.	NM_001080391	chr12	55824671	55827669	chr2	231280871	231410317
cis_mRNA up10k	NA	NONHSAT163680.1	NM_032338	chr12	66531621	66563762	chr12	66516849	66524533
cis_mRNA dw20k	NA	NONHSAT163910.1	NM_201613	chr12	98997629	99000150	chr12	99007182	99038829
cis_mRNA overlap	Lnc-Overlap-mRNA	NONHSAT163938.1	MTCONS_00046400	chr12	104513103	104531637	chr12	104510858	104532040
cis_mRNA overlap	Lnc-CompleteIn-	NONHSAT163953.1	MTCONS_00046458	chr12	107385148	107393725	chr12	107385143	107487635
cis_mRNA overlap	Lnc-CompleteIn-	NONHSAT163953.1	NM_004075	chr12	107385148	107393725	chr12	107385143	107487635
cis_mRNA overlap	Lnc-CompleteIn-	NONHSAT163956.1	MTCONS_00046466	chr12	108132207	108134942	chr12	108132207	108154914
cis_mRNA overlap	Lnc-Overlap-mRNA	NONHSAT164013.1	NM_024072	chr12	113596297	113600823	chr12	113594978	113623284
cis_mRNA dw20k	NA	NONHSAT164018.1	NM_016196	chr12	114245533	114246817	chr12	114259859	114404176
cis_mRNA overlap	Lnc-CompleteIn-	NONHSAT164043.1	NM_015335	chr12	116446619	116457185	chr12	116396381	116714991
cis_mRNA overlap	Lnc-CompleteIn-	NONHSAT164069.1	MTCONS_00046875	chr12	118633213	118633590	chr12	118587606	118797486
cis_mRNA overlap	Lnc-CompleteIn-	NONHSAT164153.1	MTCONS_00047245	chr12	125009188	125030947	chr12	124808689	125052045

cis_mRNA dw20k	NA	NONHSAT164410.1	NM_016355	chr12	12998944	13000383	chr12	12966280	12982915
cis_mRNA dw20k	NA	NONHSAT164410.1	NM_201224	chr12	12998944	13000383	chr12	12966280	12982915
cis_mRNA overlap	Lnc-Overlap-mRNA	NONHSAT165297.1	MTCONS_00048587	chr13	20655603	20657810	chr13	20530917	20666550
cis_mRNA overlap	Lnc-Overlap-mRNA	NONHSAT165297.1	MTCONS_00048591	chr13	20655603	20657810	chr13	20532810	20666550
cis_mRNA overlap	Lnc-CompleteIn-	NONHSAT165298.1	MTCONS_00048587	chr13	20657889	20660888	chr13	20530917	20666550
tran	NA	NONHSAT165346.	MTCONS_0009079	chr13	23208100	23215721	chr19	20946830	20993757
tran	NA	NONHSAT165423.	MTCONS_0018041	chr13	29344750	29360542	chr7	155437203	155480457
cis_mRNA overlap	Lnc-Overlap-mRNA	NONHSAT165475.1	MTCONS_00049027	chr13	33245963	33250712	chr13	33160564	33362316
cis_mRNA overlap	Lnc-CompleteIn-	NONHSAT165515.1	MTCONS_00049128	chr13	34420327	34424962	chr13	34392206	34424962
cis_mRNA overlap	Lnc-Overlap-mRNA	NONHSAT165741.1	MTCONS_00049834	chr13	53206553	53213829	chr13	53191605	53217919
cis_mRNA overlap	Lnc-Overlap-mRNA	NONHSAT166343.1	MTCONS_00051066	chr13	111857456	111862406	chr13	111806006	111938268
tran	NA	NONHSAT166635.	MTCONS_0011100	chr13	40533204	40533589	chr2	148687966	148778463
cis_mRNA overlap	Lnc-CompleteIn-	NONHSAT166700.1	NM_006022	chr13	45008254	45010975	chr13	45006279	45011060
cis_mRNA overlap	Lnc-Overlap-mRNA	NONHSAT166701.1	NM_183422	chr13	45008959	45151258	chr13	45006279	45150701
cis_mRNA overlap	Lnc-Overlap-mRNA	NONHSAT166914.1	MTCONS_00052818	chr13	73333197	73336118	chr13	73329540	73356266
cis_mRNA dw20k	NA	NONHSAT166937.1	MTCONS_00052890	chr13	77575706	77601277	chr13	77618790	77901177
cis_mRNA overlap	Lnc-Overlap-mRNA	NONHSAT166940.1	MTCONS_00052890	chr13	77768288	77818087	chr13	77618790	77901177
cis_mRNA overlap	Lnc-Overlap-mRNA	NONHSAT166940.1	NM_015057	chr13	77768288	77818087	chr13	77618792	77901177
cis_mRNA up10k	NA	NONHSAT167438.1	MTCONS_00051785	chr13	31736701	31737692	chr13	31709111	31736525

cis_mRNA up10k	NA	NONHSAT167438.1	MTCONS_00051786	chr13	31736701	31737692	chr13	31709111	31736525
cis_mRNA up10k	NA	NONHSAT167438.1	MTCONS_00051790	chr13	31736701	31737692	chr13	31716655	31736525
cis_mRNA up10k	NA	NONHSAT167438.1	MTCONS_00051780	chr13	31736701	31737692	chr13	31709111	31726774
cis_mRNA _overlap	Lnc-AntiOverlap-mRNA	NONHSAT168412.1	NM_018139	chr14	50100819	50117188	chr14	50091892	50101948
cis_mRNA overlap	Lnc-CompleteIn-	NONHSAT168413.1	MTCONS_00055425	chr14	50244653	50252607	chr14	50234787	50252607
cis_mRNA overlap	Lnc-CompleteIn-	NONHSAT168460.1	NM_144578	chr14	55521645	55523944	chr14	55518362	55536912
cis_mRNA overlap	Lnc-CompleteIn-	NONHSAT168486.1	NM_021255	chr14	56692000	56693653	chr14	56585093	56768031
cis_mRNA overlap	Lnc-CompleteIn-	NONHSAT168525.1	NM_021003	chr14	60752363	60760067	chr14	60715966	60765805
cis_mRNA overlap	Lnc-Overlap-mRNA	NONHSAT168525.1	MTCONS_00055809	chr14	60752363	60760067	chr14	60715966	60755272
cis_mRNA overlap	Lnc-Overlap-mRNA	NONHSAT168542.1	MTCONS_00055854	chr14	62207532	62214976	chr14	62162119	62214977
cis_mRNA overlap	Lnc-Overlap-mRNA	NONHSAT168542.1	NM_001530	chr14	62207532	62214976	chr14	62162119	62214977
cis_mRNA up10k	NA	NONHSAT168562.1	NM_001258272	chr14	64108861	64109414	chr14	64063757	64108641
cis_mRNA overlap	Lnc-CompleteIn-	NONHSAT168565.1	MTCONS_00055899	chr14	64523701	64525743	chr14	64320140	64693169
cis_mRNA overlap	Lnc-CompleteIn-	NONHSAT168565.1	NM_015180	chr14	64523701	64525743	chr14	64319683	64693169
tran	NA	NONHSAT168565.	MTCONS_0001310	chr14	64523701	64525743	chr1	114265504	114302077
tran	NA	NONHSAT168565.	MTCONS_0018731	chr14	64523701	64525743	chr8	38770940	38831459
cis_mRNA dw20k	NA	NONHSAT168568.1	NM_001308147	chr14	65214369	65217048	chr14	65171126	65211064
cis_mRNA overlap	Lnc-CompleteIn-	NONHSAT168604.1	MTCONS_00056072	chr14	70233852	70238721	chr14	70233804	70238722

cis_mRNA overlap	Lnc-Overlap-mRNA	NONHSAT168604.1	NM_006925	chr14	70233852	70238721	chr14	70233804	70238722
cis_mRNA overlap	Lnc-Overlap-mRNA	NONHSAT168605.1	MTCONS_00056071	chr14	70233873	70237776	chr14	70233804	70238722
cis_mRNA overlap	Lnc-Overlap-mRNA	NONHSAT168605.1	NM_001039465	chr14	70233873	70237776	chr14	70233829	70238722
cis_mRNA overlap	Lnc-Overlap-mRNA	NONHSAT168606.1	NM_001039465	chr14	70233874	70237003	chr14	70233829	70238722
tran	NA	NONHSAT168641.	MTCONS_0011279	chr14	74830970	74832862	chr2	208470297	208490028
cis_mRNA up10k	NA	NONHSAT168651.1	MTCONS_00056319	chr14	75743713	75744858	chr14	75745481	75748937
cis_mRNA up10k	NA	NONHSAT168651.1	NM_005252	chr14	75743713	75744858	chr14	75745481	75748937
cis_mRNA overlap	Lnc-CompleteIn-	NONHSAT168765.1	NM_207660	chr14	89041164	89078219	chr14	89029253	89079853
cis_mRNA _overlap	Lnc-AntiCompleteIn-mRNAIntron	NONHSAT168777.1	MTCONS_00059815	chr14	89991327	89994963	chr14	89622516	90085494
cis_mRNA _overlap	Lnc-AntiOverlap-mRNA	NONHSAT168836.1	NM_001127701	chr14	94856404	94871146	chr14	94843084	94857029
cis_mRNA overlap	Lnc-Overlap-mRNA	NONHSAT168916.1	MTCONS_00057106	chr14	99947776	99961369	chr14	99947739	99977852
cis_mRNA overlap	Lnc-CompleteIn-	NONHSAT168994.1	NM_001969	chr14	103800374	103804689	chr14	103800339	103811361
cis_mRNA overlap	Lnc-CompleteIn-	NONHSAT169047.1	NM_144568	chr14	20926018	20928829	chr14	20926012	20929771
cis_mRNA overlap	Lnc-Overlap-mRNA	NONHSAT169351.1	MTCONS_00058848	chr14	55861795	55864146	chr14	55832232	55878576
cis_mRNA up10k	NA	NONHSAT169469.1	MTCONS_00056123	chr14	71373374	71373828	chr14	71374122	71582099
cis_mRNA _overlap	Lnc-AntiCompleteIn-mRNAIntron	NONHSAT169475.1	NM_001284246	chr14	72145495	72149556	chr14	72052998	72207946
tran	NA	NONHSAT169475.	MTCONS_0009079	chr14	72145495	72149556	chr19	20946830	20993757

cis_mRNA overlap	Lnc-CompleteIn-	NONHSAT169628.1	MTCONS_00059889	chr14	91968239	91970505	chr14	91923825	91976824
cis_mRNA overlap	Lnc-CompleteIn-	NONHSAT169629.1	MTCONS_00059883	chr14	91972866	91973066	chr14	91923825	91976824
cis_mRNA overlap	Lnc-CompleteIn-	NONHSAT169639.1	NM_014216	chr14	93445685	93454419	chr14	93406068	93582263
cis_mRNA overlap	Lnc-Overlap-mRNA	NONHSAT169666.1	MTCONS_00060082	chr14	96807848	96829681	chr14	96746183	96829742
tran	NA	NONHSAT169786.	MTCONS_0006055	chr14	106068003	106452964	chr14	106538820	106539768
cis_mRNA overlap	Lnc-Overlap-mRNA	NONHSAT169787.1	MTCONS_00060509	chr14	106110263	106725718	chr14	106109411	106518961
cis_mRNA overlap	Lnc-Overlap-mRNA	NONHSAT169787.1	MTCONS_00060524	chr14	106110263	106725718	chr14	106235440	106878313
cis_mRNA overlap	Lnc-Overlap-mRNA	NONHSAT169790.1	MTCONS_00060514	chr14	106235440	106237745	chr14	106134592	106878313
tran	NA	NONHSAT169790.	MTCONS_0006051	chr14	106235440	106237745	chr14	106134592	106209253
cis_mRNA overlap	Lnc-CompleteIn-	NONHSAT169799.1	MTCONS_00060524	chr14	106405189	106406092	chr14	106235440	106878313
cis_mRNA overlap	Lnc-CompleteIn-	NONHSAT169800.1	MTCONS_00060514	chr14	106725203	106725721	chr14	106134592	106878313
cis_mRNA overlap	Lnc-CompleteIn-	NONHSAT169800.1	MTCONS_00060524	chr14	106725203	106725721	chr14	106235440	106878313
tran	NA	NONHSAT169800.	MTCONS_0006056	chr14	106725203	106725721	chr14	106573192	106573951
tran	NA	NONHSAT169800.	MTCONS_0006061	chr14	106725203	106725721	chr14	107048460	107049348
cis_mRNA dw20k	NA	NONHSAT170103.1	MTCONS_00055600	chr14	55263401	55264695	chr14	55076321	55260326
cis_mRNA dw20k	NA	NONHSAT170161.1	MTCONS_00055956	chr14	66215897	66217566	chr14	65879541	66213294
cis_mRNA dw20k	NA	NONHSAT170418.1	NM_002719	chr14	102399072	102401961	chr14	102276140	102394328
cis_mRNA overlap	Lnc-CompleteIn-	NONHSAT170674.1	NM_003246	chr15	39878762	39879494	chr15	39873280	39891122
cis_mRNA _overlap	Lnc-AntiCompleteIn-mRNAIntron	NONHSAT170699.1	NM_152596	chr15	41485541	41487747	chr15	41474929	41522955

cis_mRNA overlap	Lnc-Overlap-mRNA	NONHSAT170711.1	MTCONS_00062330	chr15	42602619	42607005	chr15	42565856	42704515
cis_mRNA dw20k	NA	NONHSAT170715.1	MTCONS_00065474	chr15	42819883	42822944	chr15	42834720	42841033
cis_mRNA overlap	Lnc-Overlap-mRNA	NONHSAT170732.1	MTCONS_00062416	chr15	44852441	44854287	chr15	44829266	44855221
cis_mRNA dw20k	NA	NONHSAT170786.1	NM_014547	chr15	52205351	52207919	chr15	52121825	52204331
tran	NA	NONHSAT170907.	MTCONS_0003277	chr15	63876373	63895291	chr11	129685741	129733498
cis_mRNA overlap	Lnc-CompleteIn-	NONHSAT170925.1	NM_002755	chr15	66779589	66783856	chr15	66679211	66783882
cis_mRNA overlap	Lnc-Overlap-mRNA	NONHSAT170941.1	MTCONS_00063151	chr15	68346669	68379936	chr15	68346572	68484505
cis_mRNA overlap	Lnc-Overlap-mRNA	NONHSAT170941.1	NM_016166	chr15	68346669	68379936	chr15	68346572	68480404
cis_mRNA _overlap	Lnc-AntiOverlap-mRNA	NONHSAT171032.1	NM_018285	chr15	75932101	75933317	chr15	75931426	75932664
cis_mRNA overlap	Lnc-Overlap-mRNA	NONHSAT171153.1	NM_002201	chr15	89178868	89199575	chr15	89181974	89199575
cis_mRNA overlap	Lnc-CompleteIn-	NONHSAT171171.1	NM_198925	chr15	90757889	90760073	chr15	90744562	90772892
cis_mRNA overlap	Lnc-CompleteIn-	NONHSAT171376.1	NM_024490	chr15	25953617	25956016	chr15	25923860	26108349
cis_mRNA overlap	Lnc-CompleteIn-	NONHSAT171394.1	NM_175610	chr15	30064291	30114677	chr15	29991571	30114706
tran	NA	NONHSAT171397.	MTCONS_0009079	chr15	30636510	30640439	chr19	20946830	20993757
cis_mRNA overlap	Lnc-Overlap-mRNA	NONHSAT171429.1	NM_153613	chr15	34651111	34653287	chr15	34651089	34659395
cis_mRNA overlap	Lnc-Overlap-mRNA	NONHSAT171431.1	NM_181077	chr15	34677946	34679791	chr15	34671270	34682001
cis_mRNA overlap	Lnc-CompleteIn-	NONHSAT171578.1	NM_001143887	chr15	49419671	49422981	chr15	49417471	49447854
cis_mRNA dw20k	NA	NONHSAT171603.1	MTCONS_00065894	chr15	55638545	55638950	chr15	55640209	55700708

cis_mRNA up10k	NA	NONHSAT171758.1	MTCONS_00066488	chr15	72533529	72535430	chr15	72491337	72526033
cis_mRNA dw20k	NA	NONHSAT171807.1	NM_144572	chr15	78278255	78283125	chr15	78287327	78369994
tran	NA	NONHSAT171807.	NM_014272	chr15	78278255	78283125	chr15	79051545	79103805
cis_mRNA_overlap	Lnc-AntiOverlap-mRNA	NONHSAT171903.1	MTCONS_00064010	chr15	89182681	89183205	chr15	89178868	89199575
cis_mRNA_overlap	Lnc-AntiOverlap-mRNA	NONHSAT171903.1	NM_001303236	chr15	89182681	89183205	chr15	89181974	89199575
tran	NA	NONHSAT172269.	MTCONS_0008008	chr15	65120082	65121813	chr17	3468740	3539616
cis_mRNA up10k	NA	NONHSAT172351.1	NM_000745	chr15	78850780	78853113	chr15	78857862	78887611
cis_mRNA dw20k	NA	NONHSAT172525.1	MTCONS_00071991	chr16	884611	893530	chr16	903513	1020989
cis_mRNA dw20k	NA	NONHSAT172542.1	MTCONS_00068414	chr16	1833005	1839186	chr16	1756184	1820412
cis_mRNA dw20k	NA	NONHSAT172542.1	MTCONS_00068423	chr16	1833005	1839186	chr16	1823229	1826708
cis_mRNA dw20k	NA	NONHSAT172542.1	NM_001257370	chr16	1833005	1839186	chr16	1823229	1826239
cis_mRNA overlap	Lnc-Overlap-mRNA	NONHSAT172542.1	NM_012225	chr16	1833005	1839186	chr16	1832924	1839192
cis_mRNA up10k	NA	NONHSAT172542.1	NM_080861	chr16	1833005	1839186	chr16	1826713	1832581
tran	NA	NONHSAT172551.	MTCONS_0003253	chr16	2265911	2266572	chr11	123986111	124017618
cis_mRNA dw20k	NA	NONHSAT172552.1	NM_001919	chr16	2282808	2285742	chr16	2289873	2301602
cis_mRNA up10k	NA	NONHSAT172552.1	NM_001301680	chr16	2282808	2285742	chr16	2286468	2288712
cis_mRNA overlap	Lnc-CompleteIn-	NONHSAT172556.1	NM_001694	chr16	2566659	2568000	chr16	2563871	2570224
cis_mRNA overlap	Lnc-CompleteIn-	NONHSAT172568.1	MTCONS_00068581	chr16	2988386	2999808	chr16	2961980	3001209

cis_mRNA overlap	Lnc-Overlap-mRNA	NONHSAT172568.1	NM_020912	chr16	2988386	2999808	chr16	2961980	2990158
cis_mRNA overlap	Lnc-Overlap-mRNA	NONHSAT172577.1	NM_001012631	chr16	3115665	3119009	chr16	3115313	3119668
cis_mRNA overlap	Lnc-CompleteIn-	NONHSAT172661.1	MTCONS_00068898	chr16	10480029	10481585	chr16	10479870	10577495
cis_mRNA overlap	Lnc-Overlap-mRNA	NONHSAT172661.1	MTCONS_00068901	chr16	10480029	10481585	chr16	10479912	10577495
cis_mRNA overlap	Lnc-Overlap-mRNA	NONHSAT172765.1	NM_016025	chr16	21611269	21667438	chr16	21610797	21668794
cis_mRNA dw20k	NA	NONHSAT172766.1	NM_005849	chr16	21638063	21638890	chr16	21652605	21663987
cis_mRNA dw20k	NA	NONHSAT172766.1	MTCONS_00072783	chr16	21638063	21638890	chr16	21638907	21663987
cis_mRNA overlap	Lnc-Overlap-mRNA	NONHSAT172835.1	MTCONS_00069510	chr16	29466426	29476094	chr16	29465822	29476301
cis_mRNA overlap	Lnc-Overlap-mRNA	NONHSAT172835.1	NM_001014999	chr16	29466426	29476094	chr16	29465822	29469545
cis_mRNA overlap	Lnc-Overlap-mRNA	NONHSAT172835.1	NM_001017390	chr16	29466426	29476094	chr16	29471207	29476301
cis_mRNA overlap	Lnc-Overlap-mRNA	NONHSAT172835.1	NM_024044	chr16	29466426	29476094	chr16	29465822	29469545
tran	NA	NONHSAT172835.	NM_177530	chr16	29466426	29476094	chr16	28616908	28620649
cis_mRNA dw20k	NA	NONHSAT172850.1	MTCONS_00069581	chr16	30015272	30022988	chr16	29985188	30003582
cis_mRNA dw20k	NA	NONHSAT172850.1	NM_016151	chr16	30015272	30022988	chr16	29985188	29999726
cis_mRNA overlap	Lnc-Overlap-mRNA	NONHSAT172850.1	NM_173618	chr16	30015272	30022988	chr16	30007530	30017111
cis_mRNA up10k	NA	NONHSAT172850.1	NM_003609	chr16	30015272	30022988	chr16	30003642	30007417
tran	NA	NONHSAT172853.	MTCONS_0003070	chr16	30122216	30123276	chr11	67155110	67165883
cis_mRNA overlap	Lnc-CompleteIn-	NONHSAT172864.1	MTCONS_00069661	chr16	30662224	30664101	chr16	30662188	30667761

cis_mRNA dw20k	NA	NONHSAT172954.1	MTCONS_00070036	chr16	48390711	48392738	chr16	48278078	48390218
cis_mRNA overlap	Lnc-Overlap-mRNA	NONHSAT172986.1	NM_182922	chr16	50136328	50140291	chr16	50099881	50139375
cis_mRNA overlap	Lnc-CompleteIn-	NONHSAT172989.1	MTCONS_00070113	chr16	50259189	50263408	chr16	50186829	50269368
cis_mRNA_overlap	Lnc-AntiOverlap-mRNA	NONHSAT173121.1	MTCONS_00073778	chr16	58556874	58557746	chr16	58553850	58663790
cis_mRNA dw20k	NA	NONHSAT173213.1	NM_022341	chr16	69345177	69350218	chr16	69362524	69364498
cis_mRNA dw20k	NA	NONHSAT173213.1	NM_032382	chr16	69345177	69350218	chr16	69362524	69373526
cis_mRNA dw20k	NA	NONHSAT173213.1	NM_006750	chr16	69345177	69350218	chr16	69221050	69342955
cis_mRNA dw20k	NA	NONHSAT173221.1	NM_001605	chr16	70283058	70285793	chr16	70286297	70323412
cis_mRNA dw20k	NA	NONHSAT173224.1	NM_001172772	chr16	70695570	70699739	chr16	70657895	70694585
tran	NA	NONHSAT173449.	MTCONS_0003253	chr16	88634561	88635563	chr11	123986111	124017618
cis_mRNA dw20k	NA	NONHSAT173451.1	MTCONS_00074858	chr16	88857281	88860815	chr16	88875877	88923374
cis_mRNA overlap	Lnc-Overlap-mRNA	NONHSAT173490.1	MTCONS_00071952	chr16	684431	686316	chr16	684427	686366
cis_mRNA_overlap	Lnc-AntiOverlap-mRNA	NONHSAT173527.1	MTCONS_00068555	chr16	2787077	2802601	chr16	2802330	2821428
tran	NA	NONHSAT173605.	MTCONS_0003070	chr16	11931213	11933662	chr11	67155110	67165883
cis_mRNA overlap	Lnc-CompleteIn-	NONHSAT173659.1	NM_001304499	chr16	15959582	15977878	chr16	15959576	15982506
cis_mRNA overlap	Lnc-CompleteIn-	NONHSAT173663.1	NM_022166	chr16	17343531	17345252	chr16	17196181	17564738
cis_mRNA_overlap	Lnc-AntiOverlap-mRNA	NONHSAT173738.1	NM_007245	chr16	28833752	28835290	chr16	28834369	28848558

cis_mRNA overlap	Lnc-Overlap-mRNA	NONHSAT173741.1	NM_024816	chr16	28926058	28937010	chr16	28915742	28936532
cis_mRNA overlap	Lnc-Overlap-mRNA	NONHSAT173745.1	MTCONS_00073057	chr16	29454572	29461235	chr16	29454226	29466285
cis_mRNA dw20k	NA	NONHSAT173747.1	MTCONS_00073066	chr16	29528103	29528460	chr16	29535926	29625053
cis_mRNA dw20k	NA	NONHSAT173830.1	NM_001006610	chr16	48380842	48387407	chr16	48394447	48399784
cis_mRNA overlap	Lnc-Overlap-mRNA	NONHSAT173917.1	NM_007006	chr16	56466537	56469045	chr16	56463048	56485261
cis_mRNA _overlap	Lnc-AntiOverlap-mRNA	NONHSAT173918.1	NM_018233	chr16	56499554	56500074	chr16	56485424	56511407
cis_mRNA overlap	Lnc-Overlap-mRNA	NONHSAT173934.1	NM_001130100	chr16	57824690	57831807	chr16	57792129	57836439
cis_mRNA up10k	NA	NONHSAT173987.1	NM_006565	chr16	67584718	67586838	chr16	67596310	67673088
cis_mRNA dw20k	NA	NONHSAT174001.1	NM_032382	chr16	69360394	69361625	chr16	69362524	69373526
cis_mRNA dw20k	NA	NONHSAT174009.1	NM_006927	chr16	70305131	70411976	chr16	70413338	70472991
cis_mRNA overlap	Lnc-CompleteIn-	NONHSAT174012.1	MTCONS_00074128	chr16	70747635	70750911	chr16	70721342	70835061
cis_mRNA dw20k	NA	NONHSAT174049.1	MTCONS_00074283	chr16	74339637	74341668	chr16	74357188	74402046
cis_mRNA dw20k	NA	NONHSAT174284.1	NM_014117	chr16	9218688	9220997	chr16	9185537	9213555
cis_mRNA overlap	Lnc-Overlap-mRNA	NONHSAT174765.1	MTCONS_00075575	chr17	2274559	2276259	chr17	2240806	2284348
cis_mRNA overlap	Lnc-Overlap-mRNA	NONHSAT174813.1	MTCONS_00075817	chr17	6659397	6663664	chr17	6659156	6678964
cis_mRNA overlap	Lnc-Overlap-mRNA	NONHSAT174813.1	NM_199139	chr17	6659397	6663664	chr17	6659156	6678964
cis_mRNA overlap	Lnc-Overlap-mRNA	NONHSAT174822.1	NM_014716	chr17	7248918	7252186	chr17	7239848	7254793

cis_mRNA dw20k	NA	NONHSAT174943.1	NM_020653	chr17	16439866	16440268	chr17	16453631	16472520
cis_mRNA overlap	Lnc-Overlap-mRNA	NONHSAT175029.1	NM_015626	chr17	25621169	25635231	chr17	25621106	25640645
tran	NA	NONHSAT175057.	NM_016281	chr17	27717431	27822731	chr12	118587606	118810750
cis_mRNA overlap	Lnc-CompleteIn-	NONHSAT175096.1	NM_001270482	chr17	30775930	30778126	chr17	30771481	30810337
cis_mRNA overlap	Lnc-CompleteIn-	NONHSAT175134.1	NM_001291470	chr17	34431220	34433014	chr17	34431220	34433014
cis_mRNA _overlap	Lnc-AntiCompleteIn-mRNAIntron	NONHSAT175137.1	MTCONS_00081678	chr17	34538310	34540280	chr17	34522269	34625731
cis_mRNA overlap	Lnc-CompleteIn-	NONHSAT175192.1	MTCONS_00077509	chr17	38282507	38286194	chr17	38278790	38293044
cis_mRNA overlap	Lnc-Overlap-mRNA	NONHSAT175208.1	NM_005801	chr17	39845153	39847893	chr17	39845127	39847898
cis_mRNA dw20k	NA	NONHSAT175222.1	NM_025233	chr17	40721532	40724725	chr17	40714092	40718299
cis_mRNA _overlap	Lnc-AntiCompleteIn-mRNAIntron	NONHSAT175226.1	MTCONS_00082182	chr17	41277699	41305675	chr17	41196240	41328675
cis_mRNA overlap	Lnc-Overlap-mRNA	NONHSAT175227.1	MTCONS_00077710	chr17	41329190	41331653	chr17	41329190	41363708
cis_mRNA dw20k	NA	NONHSAT175241.1	NM_004160	chr17	42004999	42011882	chr17	42030101	42081837
tran	NA	NONHSAT175258.	NM_025136	chr17	43117272	43117828	chr19	46049539	46088122
cis_mRNA overlap	Lnc-CompleteIn-	NONHSAT175278.1	MTCONS_00077954	chr17	44680600	44688810	chr17	44450147	44834828
cis_mRNA overlap	Lnc-Overlap-mRNA	NONHSAT175343.1	NM_001242791	chr17	47923273	47926200	chr17	47923272	47926199
cis_mRNA overlap	Lnc-CompleteIn-	NONHSAT175356.1	NM_016424	chr17	48799435	48801585	chr17	48796926	48830072
cis_mRNA _overlap	Lnc-AntiOverlap-mRNA	NONHSAT175465.1	MTCONS_00083151	chr17	61780887	61791225	chr17	61780192	61820272

cis_mRNA overlap	Lnc-CompleteIn-	NONHSAT175516.1	MTCONS_00078821	chr17	65951430	65953407	chr17	65821780	65980494
cis_mRNA overlap	Lnc-CompleteIn-	NONHSAT175516.1	MTCONS_00078824	chr17	65951430	65953407	chr17	65821780	65980494
tran	NA	NONHSAT175522.	MTCONS_0008163	chr17	66148862	66153975	chr17	34231224	34257780
cis_mRNA dw20k	NA	NONHSAT175547.1	NM_002758	chr17	67538552	67547417	chr17	67410838	67538470
cis_mRNA _overlap	Lnc-AntiOverlap-mRNA	NONHSAT175595.1	NM_001129885	chr17	71228761	71245095	chr17	71244588	71258019
cis_mRNA dw20k	NA	NONHSAT175631.1	MTCONS_00083847	chr17	74722933	74729961	chr17	74730197	74733493
cis_mRNA dw20k	NA	NONHSAT175631.1	MTCONS_00083848	chr17	74722933	74729961	chr17	74730197	74733493
cis_mRNA overlap	Lnc-CompleteIn-	NONHSAT175631.1	NM_001080510	chr17	74722933	74729961	chr17	74722912	74729963
cis_mRNA up10k	NA	NONHSAT175631.1	NM_001242533	chr17	74722933	74729961	chr17	74733583	74775336
cis_mRNA up10k	NA	NONHSAT175631.1	NM_015167	chr17	74722933	74729961	chr17	74714525	74722881
cis_mRNA up10k	NA	NONHSAT175721.1	MTCONS_00084003	chr17	78121049	78122395	chr17	78109013	78115394
cis_mRNA up10k	NA	NONHSAT175721.1	NM_014740	chr17	78121049	78122395	chr17	78109013	78120982
cis_mRNA overlap	Lnc-CompleteIn-	NONHSAT175722.1	MTCONS_00079596	chr17	78234879	78261625	chr17	78234879	78372581
cis_mRNA overlap	Lnc-Overlap-mRNA	NONHSAT175746.1	NM_001291324	chr17	79369271	79376190	chr17	79373521	79433358
cis_mRNA overlap	Lnc-Overlap-mRNA	NONHSAT175865.1	NM_005022	chr17	4848962	4851615	chr17	4848945	4852381
cis_mRNA overlap	Lnc-CompleteIn-	NONHSAT175871.1	NM_002532	chr17	5293548	5303824	chr17	5288153	5323059
cis_mRNA up10k	NA	NONHSAT175904.1	NM_172087	chr17	7461137	7461562	chr17	7461609	7464925
tran	NA	NONHSAT175907.	MTCONS_0015603	chr17	7588574	7590782	chr5	179247842	179267871

cis_mRNA up10k	NA	NONHSAT175913.1	MTCONS_00080523	chr17	8056769	8059642	chr17	8043788	8056490
cis_mRNA up10k	NA	NONHSAT175913.1	MTCONS_00080525	chr17	8056769	8059642	chr17	8043788	8056490
cis_mRNA up10k	NA	NONHSAT175913.1	MTCONS_00080520	chr17	8056769	8059642	chr17	8043788	8056487
cis_mRNA overlap	Lnc-Overlap-mRNA	NONHSAT175916.1	MTCONS_00080557	chr17	8138234	8140866	chr17	8123948	8151413
cis_mRNA_overlap	Lnc-AntiOverlap-mRNA	NONHSAT175925.1	NM_004822	chr17	9140911	9144186	chr17	8924859	9147317
cis_mRNA overlap	Lnc-CompleteIn-	NONHSAT175976.1	NM_001135036	chr17	15440510	15466837	chr17	15440294	15466945
cis_mRNA overlap	Lnc-Overlap-mRNA	NONHSAT175976.1	MTCONS_00080795	chr17	15440510	15466837	chr17	15337417	15466945
cis_mRNA overlap	Lnc-Overlap-mRNA	NONHSAT175977.1	MTCONS_00080795	chr17	15448368	15466908	chr17	15337417	15466945
cis_mRNA overlap	Lnc-Overlap-mRNA	NONHSAT175977.1	MTCONS_00080810	chr17	15448368	15466908	chr17	15440294	15466945
cis_mRNA up10k	NA	NONHSAT175986.1	NM_001271420	chr17	15881058	15897260	chr17	15902694	15932723
cis_mRNA dw20k	NA	NONHSAT176002.1	MTCONS_00081022	chr17	18420696	18432298	chr17	18434738	18528903
cis_mRNA overlap	Lnc-Overlap-mRNA	NONHSAT176002.1	MTCONS_00081000	chr17	18420696	18432298	chr17	18413966	18528903
cis_mRNA overlap	Lnc-Overlap-mRNA	NONHSAT176002.1	MTCONS_00081001	chr17	18420696	18432298	chr17	18413966	18528903
cis_mRNA overlap	Lnc-Overlap-mRNA	NONHSAT176136.1	NM_002983	chr17	34415603	34417489	chr17	34415602	34417506
tran	NA	NONHSAT176136.	MTCONS_0008167	chr17	34415603	34417489	chr17	34522269	34625731
cis_mRNA overlap	Lnc-CompleteIn-	NONHSAT176167.1	MTCONS_00081838	chr17	37259283	37259593	chr17	37219556	37307902
cis_mRNA overlap	Lnc-CompleteIn-	NONHSAT176210.1	MTCONS_00082091	chr17	40265132	40266412	chr17	40265129	40273382

cis_mRNA overlap	Lnc-Overlap-mRNA	NONHSAT176228.1	NM_006373	chr17	41166624	41170220	chr17	41166622	41174459
cis_mRNA dw20k	NA	NONHSAT176249.1	NM_001098833	chr17	42264343	42267550	chr17	42269173	42275529
cis_mRNA dw20k	NA	NONHSAT176281.1	NM_001113738	chr17	44396628	44401871	chr17	44412268	44439163
cis_mRNA overlap	Lnc-CompleteIn-	NONHSAT176309.1	NM_001075099	chr17	46408279	46408522	chr17	46210802	46507594
cis_mRNA overlap	Lnc-Overlap-mRNA	NONHSAT176352.1	MTCONS_00082801	chr17	49118474	49125023	chr17	49038497	49198226
cis_mRNA overlap	Lnc-Overlap-mRNA	NONHSAT176352.1	NM_003971	chr17	49118474	49125023	chr17	49039535	49198226
tran	NA	NONHSAT176441.	NM_015500	chr17	61528357	61530474	chr21	43305219	43373999
tran	NA	NONHSAT176445.	MTCONS_0007436	chr17	61788894	61790998	chr16	75632247	75658035
cis_mRNA up10k	NA	NONHSAT176458.1	NM_000515	chr17	62006106	62007178	chr17	61994553	61996212
cis_mRNA overlap	Lnc-Overlap-mRNA	NONHSAT176462.1	MTCONS_00083224	chr17	62499057	62502209	chr17	62494372	62502639
cis_mRNA up10k	NA	NONHSAT176462.1	MTCONS_00083219	chr17	62499057	62502209	chr17	62473902	62493184
cis_mRNA overlap	Lnc-Overlap-mRNA	NONHSAT176463.1	MTCONS_00083237	chr17	62745641	62747358	chr17	62745641	62811106
cis_mRNA dw20k	NA	NONHSAT176592.1	NM_001080510	chr17	74730205	74732551	chr17	74722912	74729963
cis_mRNA overlap	Lnc-Overlap-mRNA	NONHSAT176592.1	MTCONS_00083847	chr17	74730205	74732551	chr17	74730197	74733493
cis_mRNA overlap	Lnc-Overlap-mRNA	NONHSAT176592.1	MTCONS_00083848	chr17	74730205	74732551	chr17	74730197	74733493
cis_mRNA _overlap	Lnc-AntiOverlap-mRNA	NONHSAT176618.1	MTCONS_00079445	chr17	75940228	76003563	chr17	75954756	76104916
cis_mRNA overlap	Lnc-CompleteIn-	NONHSAT176625.1	MTCONS_00083941	chr17	76687348	76688061	chr17	76670129	76732947
cis_mRNA dw20k	NA	NONHSAT176679.1	MTCONS_00084221	chr17	80059346	80092031	chr17	80111450	80170744

cis_mRNA up10k	NA	NONHSAT176679.1	NM_004104	chr17	80059346	80092031	chr17	80036214	80056106
cis_mRNA dw20k	NA	NONHSAT176881.1	NM_015626	chr17	25655748	25656483	chr17	25621106	25640645
cis_mRNA up10k	NA	NONHSAT177072.1	NM_005450	chr17	54670076	54670672	chr17	54671060	54672951
cis_mRNA up10k	NA	NONHSAT177195.1	NM_052916	chr17	74236828	74237701	chr17	74138534	74236390
tran	NA	NONHSAT177199.	MTCONS_0014142	chr17	74519332	74520270	chr4	2965230	2972037
cis_mRNA overlap	Lnc-Overlap-mRNA	NONHSAT177282.1	NM_001291470	chr17	34431066	34433014	chr17	34431220	34433014
cis_mRNA overlap	Lnc-Overlap-mRNA	NONHSAT177282.1	NM_002984	chr17	34431066	34433014	chr17	34431220	34433014
cis_mRNA overlap	Lnc-CompleteIn-	NONHSAT177283.1	NM_001291469	chr17	34431220	34433014	chr17	34431220	34433014
cis_mRNA _overlap	Lnc-AntiCompleteIn-mRNAIntron	NONHSAT177284.1	MTCONS_00081678	chr17	34538310	34540280	chr17	34522269	34625731
cis_mRNA overlap	Lnc-Overlap-mRNA	NONHSAT177296.1	NM_002983	chr17	34415603	34417489	chr17	34415602	34417506
tran	NA	NONHSAT177296.	MTCONS_0008167	chr17	34415603	34417489	chr17	34522269	34625731
cis_mRNA _overlap	Lnc-AntiCompleteIn-mRNAIntron	NONHSAT177310.1	MTCONS_00081678	chr17	34538310	34540280	chr17	34522269	34625731
tran	NA	NONHSAT177310.	NM_002984	chr17	34538310	34540280	chr17	34431220	34433014
tran	NA	NONHSAT177312.	NM_001291470	chr17	34639876	34641846	chr17	34431220	34433014
cis_mRNA overlap	Lnc-Overlap-mRNA	NONHSAT177314.1	MTCONS_00081678	chr17	34623843	34625731	chr17	34522269	34625731
tran	NA	NONHSAT177314.	NM_002983	chr17	34623843	34625731	chr17	34415602	34417506
tran	NA	NONHSAT177330.	MTCONS_0009079	chr18	976591	1174805	chr19	20946830	20993757
cis_mRNA overlap	Lnc-CompleteIn-	NONHSAT177356.1	NM_001303047	chr18	3248027	3248607	chr18	3247528	3256235
cis_mRNA overlap	Lnc-CompleteIn-	NONHSAT177422.1	NM_194434	chr18	9914279	9954811	chr18	9913955	9960018

cis_mRNA_overlap	Lnc-AntiOverlap-mRNA	NONHSAT177457.1	MTCONS_00087312	chr18	11908711	11909222	chr18	11883470	11908796
tran	NA	NONHSAT177476.	MTCONS_0011279	chr18	14010053	14010916	chr2	208470297	208490028
cis_mRNA_overlap	Lnc-CompleteIn-	NONHSAT177823.1	MTCONS_00086242	chr18	51809370	51821092	chr18	51796306	51824604
cis_mRNA_overlap	Lnc-CompleteIn-	NONHSAT177900.1	NM_017742	chr18	60218436	60219738	chr18	60190658	60245818
tran	NA	NONHSAT178051.	MTCONS_0013211	chr18	74240874	74274170	chr3	122296449	122357894
cis_mRNA_overlap	Lnc-CompleteIn-	NONHSAT178122.1	MTCONS_00086932	chr18	77496503	77514501	chr18	77441430	77514510
cis_mRNA_overlap	Lnc-Overlap-mRNA	NONHSAT178169.1	MTCONS_00087157	chr18	5392394	5395142	chr18	5392380	5628990
cis_mRNA_up10k	NA	NONHSAT178194.1	NM_015208	chr18	9135711	9136489	chr18	9136751	9285983
tran	NA	NONHSAT178369.	MTCONS_0009079	chr18	33818930	33819140	chr19	20946830	20993757
cis_mRNA_dw20k	NA	NONHSAT178474.1	NM_000985	chr18	47008046	47010105	chr18	47014851	47018935
cis_mRNA_overlap	Lnc-CompleteIn-	NONHSAT178474.1	NM_001199346	chr18	47008046	47010105	chr18	47007548	47013644
tran	NA	NONHSAT179272.	MTCONS_0009079	chr18	75289297	75291956	chr19	20946830	20993757
cis_mRNA_overlap	Lnc-Overlap-mRNA	NONHSAT179324.1	NM_001300815	chr19	1273610	1274806	chr19	1269265	1274808
cis_mRNA_overlap	Lnc-CompleteIn-	NONHSAT179325.1	NM_032853	chr19	1373883	1375161	chr19	1354976	1378430
cis_mRNA_dw20k	NA	NONHSAT179332.1	NM_001308226	chr19	1440838	1441937	chr19	1438424	1440496
cis_mRNA_overlap	Lnc-CompleteIn-	NONHSAT179350.1	MTCONS_00089467	chr19	2345251	2353926	chr19	2328629	2355100
cis_mRNA_overlap	Lnc-CompleteIn-	NONHSAT179366.1	NM_006339	chr19	3578008	3579079	chr19	3572943	3579081
cis_mRNA_overlap	Lnc-CompleteIn-	NONHSAT179416.1	NM_031203	chr19	8521218	8521638	chr19	8509803	8554002
cis_mRNA_overlap	Lnc-Overlap-mRNA	NONHSAT179421.1	NM_001202406	chr19	9435369	9438826	chr19	9434448	9454521

tran	NA	NONHSAT179433.	MTCONS_0007428	chr19	9732223	9756165	chr16	74357188	74402046
cis_mRNA _overlap	mRNA- AntiCompleteIn -LncIntron	NONHSAT179441. 1	NM_203500	chr19	10496482	11044209	chr19	10596796	10614054
cis_mRNA overlap	Lnc-Overlap- mRNA	NONHSAT179450. 1	MTCONS_0009004 9	chr19	10897369	10917305	chr19	10828729	10942586
cis_mRNA overlap	Lnc- CompleteIn-	NONHSAT179458. 1	MTCONS_0009014 0	chr19	11966847	11969117	chr19	11959535	11980306
tran	NA	NONHSAT179459.	MTCONS_0004000	chr19	12035960	12058123	chr12	51318534	51353202
cis_mRNA dw20k	NA	NONHSAT179468. 1	MTCONS_0009023 3	chr19	13278337	13281649	chr19	13261245	13276986
cis_mRNA overlap	Lnc-Overlap- mRNA	NONHSAT179468. 1	MTCONS_0009023 6	chr19	13278337	13281649	chr19	13262328	13281649
cis_mRNA dw20k	NA	NONHSAT179469. 1	MTCONS_0009023 5	chr19	13278337	13316340	chr19	13261282	13276986
cis_mRNA overlap	Lnc-Overlap- mRNA	NONHSAT179505. 1	MTCONS_0009043 3	chr19	16264111	16266999	chr19	16244654	16269384
cis_mRNA overlap	Lnc- CompleteIn-	NONHSAT179557. 1	NM_145326	chr19	21580734	21583049	chr19	21579921	21591601
tran	NA	NONHSAT179670.	MTCONS_0009079	chr19	35196416	35196747	chr19	20946830	20993757
cis_mRNA overlap	Lnc-Overlap- mRNA	NONHSAT179700. 1	NM_001007469	chr19	36393442	36395173	chr19	36393382	36395173
cis_mRNA overlap	Lnc-Overlap- mRNA	NONHSAT179700. 1	NM_014266	chr19	36393442	36395173	chr19	36393382	36395173
cis_mRNA dw20k	NA	NONHSAT179786. 1	NM_013362	chr19	44645745	44653430	chr19	44617548	44637255
cis_mRNA overlap	Lnc-Overlap- mRNA	NONHSAT179800. 1	MTCONS_0009217 7	chr19	45570601	45574213	chr19	45542298	45574214
cis_mRNA overlap	Lnc-Overlap- mRNA	NONHSAT179800. 1	NM_007056	chr19	45570601	45574213	chr19	45542298	45574214
cis_mRNA overlap	Lnc-Overlap- mRNA	NONHSAT179822. 1	NM_005184	chr19	47109074	47112559	chr19	47104512	47114039
cis_mRNA dw20k	NA	NONHSAT179855. 1	NM_000554	chr19	48349271	48356854	chr19	48325099	48346586

cis_mRNA overlap	Lnc-CompleteIn-	NONHSAT179872.1	MTCONS_00092495	chr19	49607654	49611859	chr19	49588397	49611870
cis_mRNA overlap	Lnc-Overlap-mRNA	NONHSAT179872.1	MTCONS_00092497	chr19	49607654	49611859	chr19	49588397	49611870
cis_mRNA overlap	Lnc-Overlap-mRNA	NONHSAT179872.1	NM_001301069	chr19	49607654	49611859	chr19	49588397	49611870
cis_mRNA overlap	Lnc-Overlap-mRNA	NONHSAT179873.1	MTCONS_00092495	chr19	49609835	49611857	chr19	49588397	49611870
cis_mRNA up10k	NA	NONHSAT179873.1	NM_022165	chr19	49609835	49611857	chr19	49617618	49621717
cis_mRNA overlap	Lnc-Overlap-mRNA	NONHSAT179889.1	NM_001193646	chr19	50432037	50434379	chr19	50432400	50437193
cis_mRNA overlap	Lnc-CompleteIn-	NONHSAT179895.1	MTCONS_00092629	chr19	50983871	51013154	chr19	50979734	51013537
cis_mRNA overlap	Lnc-Overlap-mRNA	NONHSAT179960.1	NM_001136134	chr19	55898014	55899739	chr19	55897300	55903451
cis_mRNA overlap	Lnc-Overlap-mRNA	NONHSAT179960.1	NM_001136135	chr19	55898014	55899739	chr19	55897300	55903451
cis_mRNA dw20k	NA	NONHSAT179987.1	MTCONS_00093348	chr19	57961057	57964379	chr19	57946693	57960887
cis_mRNA dw20k	NA	NONHSAT180001.1	NM_021089	chr19	58808733	58813217	chr19	58790318	58807254
cis_mRNA dw20k	NA	NONHSAT180033.1	NM_012292	chr19	1088168	1090967	chr19	1067165	1087830
cis_mRNA overlap	Lnc-CompleteIn-	NONHSAT180088.1	NM_174983	chr19	3544198	3546109	chr19	3544197	3557582
cis_mRNA overlap	Lnc-CompleteIn-	NONHSAT180135.1	NM_001079817	chr19	7114921	7116555	chr19	7112266	7294313
tran	NA	NONHSAT180150.	MTCONS_0007915	chr19	8415456	8416481	chr17	73083822	73104313
cis_mRNA overlap	Lnc-Overlap-mRNA	NONHSAT180159.1	NM_001130823	chr19	10257480	10260695	chr19	10244020	10305811
cis_mRNA overlap	Lnc-Overlap-mRNA	NONHSAT180159.1	NM_001379	chr19	10257480	10260695	chr19	10244020	10305811
cis_mRNA up10k	NA	NONHSAT180178.1	MTCONS_00090236	chr19	13254877	13255688	chr19	13262328	13281649

cis_mRNA up10k	NA	NONHSAT180227.1	NM_001286968	chr19	18400253	18403814	chr19	18390504	18392466
cis_mRNA up10k	NA	NONHSAT180227.1	NM_005354	chr19	18400253	18403814	chr19	18390504	18392466
cis_mRNA overlap	Lnc-CompleteIn-	NONHSAT180284.1	NM_138286	chr19	23927706	23941654	chr19	23921997	23941693
cis_mRNA dw20k	NA	NONHSAT180367.1	MTCONS_00095558	chr19	36230148	36233329	chr19	36233428	36236343
cis_mRNA up10k	NA	NONHSAT180367.1	NM_172341	chr19	36230148	36233329	chr19	36236478	36238056
cis_mRNA overlap	Lnc-Overlap-mRNA	NONHSAT180405.1	NM_001020	chr19	39923871	39926571	chr19	39923847	39926618
tran	NA	NONHSAT180415.	NM_030650	chr19	40859820	40882276	chr2	176788620	176867073
cis_mRNA overlap	Lnc-Overlap-mRNA	NONHSAT180488.1	NM_014417	chr19	47724081	47734474	chr19	47724079	47734451
tran	NA	NONHSAT180495.	MTCONS_0003253	chr19	48379974	48391430	chr11	123986111	124017618
cis_mRNA dw20k	NA	NONHSAT180545.1	NM_001098612	chr19	52142731	52143184	chr19	52145806	52150132
cis_mRNA _overlap	Lnc-AntiCompleteIn-mRNAExon	NONHSAT180561.1	NM_152473	chr19	53517584	53519833	chr19	53517344	53519833
cis_mRNA dw20k	NA	NONHSAT180596.1	NM_001136136	chr19	55913290	55914350	chr19	55897300	55898408
cis_mRNA dw20k	NA	NONHSAT180596.1	NM_000991	chr19	55913290	55914350	chr19	55897300	55903451
cis_mRNA _overlap	Lnc-AntiCompleteIn-mRNAExon	NONHSAT180611.1	NM_003417	chr19	57730238	57732870	chr19	57702868	57734214
cis_mRNA dw20k	NA	NONHSAT180628.1	NM_003433	chr19	58938883	58941763	chr19	58944181	58951589
cis_mRNA up10k	NA	NONHSAT180653.1	NM_003200	chr19	1652764	1653474	chr19	1609289	1652328
cis_mRNA dw20k	NA	NONHSAT180664.1	NM_015675	chr19	2495004	2495602	chr19	2476123	2478257

cis_mRNA up10k	NA	NONHSAT180668.1	MTCONS_00089513	chr19	3166987	3170538	chr19	3178736	3209573
cis_mRNA dw20k	NA	NONHSAT180846.1	MTCONS_00091935	chr19	42104901	42105767	chr19	42082270	42093220
cis_mRNA overlap	Lnc-Overlap-mRNA	NONHSAT180944.1	NM_001289027	chr19	54865233	54876721	chr19	54865233	54882163
cis_mRNA overlap	Lnc-Overlap-mRNA	NONHSAT181070.1	NM_080921	chr1	198608098	198662334	chr1	198608098	198726605
cis_mRNA dw20k	NA	NONHSAT181266.1	MTCONS_00106846	chr2	11304330	11318984	chr2	11321778	11428624
cis_mRNA dw20k	NA	NONHSAT181266.1	NM_004850	chr2	11304330	11318984	chr2	11321778	11484711
cis_mRNA overlap	Lnc-CompleteIn-	NONHSAT181266.1	NM_152391	chr2	11304330	11318984	chr2	11295498	11318998
cis_mRNA _overlap	Lnc-AntiCompleteIn-mRNAIntron	NONHSAT181271.1	NM_004850	chr2	11456746	11459838	chr2	11321778	11484711
tran	NA	NONHSAT181271.	MTCONS_0011100	chr2	11456746	11459838	chr2	148687966	148778463
cis_mRNA dw20k	NA	NONHSAT181396.1	NM_004040	chr2	20650297	20652994	chr2	20646832	20649204
cis_mRNA overlap	Lnc-Overlap-mRNA	NONHSAT181485.1	NM_002709	chr2	28974742	29022765	chr2	28974626	29025806
cis_mRNA overlap	Lnc-Overlap-mRNA	NONHSAT181486.1	NM_002709	chr2	29001442	29005691	chr2	28974626	29025806
cis_mRNA overlap	Lnc-Overlap-mRNA	NONHSAT181486.1	NM_206876	chr2	29001442	29005691	chr2	28974614	29025806
cis_mRNA dw20k	NA	NONHSAT181722.1	MTCONS_00099582	chr2	46888187	46902927	chr2	46844311	46886111
cis_mRNA dw20k	NA	NONHSAT181876.1	NM_014709	chr2	61405226	61414047	chr2	61414590	61697849
cis_mRNA overlap	Lnc-CompleteIn-	NONHSAT181981.1	MTCONS_00100309	chr2	70128283	70129687	chr2	70121075	70142037
tran	NA	NONHSAT182041.	MTCONS_0011100	chr2	75020893	75023305	chr2	148687966	148778463
cis_mRNA overlap	Lnc-Overlap-mRNA	NONHSAT182120.1	NM_001302758	chr2	85923476	85925870	chr2	85921414	85925974

cis_mRNA overlap	Lnc-Overlap-mRNA	NONHSAT182120.1	NM_006433	chr2	85923476	85925870	chr2	85921414	85925974
cis_mRNA overlap	Lnc-Overlap-mRNA	NONHSAT182120.1	NM_012483	chr2	85923476	85925870	chr2	85921414	85925974
cis_mRNA up10k	NA	NONHSAT182132.1	NM_016622	chr2	86422713	86423172	chr2	86426556	86440477
tran	NA	NONHSAT182198.	MTCONS_0009437	chr2	97333063	97335485	chr19	11487649	11495018
tran	NA	NONHSAT182198.	MTCONS_0012201	chr2	97333063	97335485	chr21	37529006	37668070
cis_mRNA _overlap	mRNA-AntiCompleteIn-LncIntron	NONHSAT182202.1	NM_144994	chr2	97498800	97512467	chr2	97503651	97509758
tran	NA	NONHSAT182206.	MTCONS_0010957	chr2	97865325	97868099	chr2	96501067	96658068
cis_mRNA dw20k	NA	NONHSAT182215.1	MTCONS_00109706	chr2	98262554	98264656	chr2	98272402	98280561
cis_mRNA up10k	NA	NONHSAT182414.1	MTCONS_00110239	chr2	113596459	113599069	chr2	113587337	113594356
cis_mRNA up10k	NA	NONHSAT182414.1	MTCONS_00110242	chr2	113596459	113599069	chr2	113587337	113594356
tran	NA	NONHSAT182427.	MTCONS_0014142	chr2	113986510	113989532	chr4	2965230	2972037
cis_mRNA overlap	Lnc-Overlap-mRNA	NONHSAT182705.1	NM_001282798	chr2	136389488	136392048	chr2	136343893	136482840
tran	NA	NONHSAT182903.	MTCONS_0003070	chr2	149851714	149854931	chr11	67155110	67165883
cis_mRNA up10k	NA	NONHSAT182954.1	MTCONS_00111174	chr2	157193084	157198632	chr2	157180944	157190970
cis_mRNA up10k	NA	NONHSAT182955.1	MTCONS_00111174	chr2	157193085	157198633	chr2	157180944	157190970
cis_mRNA _overlap	Lnc-AntiOverlap-mRNA	NONHSAT183033.1	MTCONS_00111414	chr2	166646288	166866540	chr2	166604313	166651180
cis_mRNA _overlap	mRNA-AntiCompleteIn-LncExon	NONHSAT183033.1	NM_024753	chr2	166646288	166866540	chr2	166729872	166810348
cis_mRNA overlap	Lnc-Overlap-mRNA	NONHSAT183090.1	MTCONS_00103674	chr2	173450754	173457995	chr2	173420697	173480770

cis_mRNA_overlap	Lnc-Overlap-mRNA	NONHSAT183160.1	NM_194247	chr2	178083764	178087368	chr2	178077422	178088685
cis_mRNA_overlap	Lnc-AntiOverlap-mRNA	NONHSAT183199.1	NM_133378	chr2	179501288	179578966	chr2	179390718	179672150
cis_mRNA_overlap	Lnc-CompleteIn-	NONHSAT183232.1	MTCONS_00104051	chr2	182358072	182361161	chr2	182321619	182361161
cis_mRNA_overlap	Lnc-Overlap-mRNA	NONHSAT183311.1	MTCONS_00104265	chr2	191784256	191786246	chr2	191764923	191800015
tran	NA	NONHSAT183360.	MTCONS_0009079	chr2	194768526	194858393	chr19	20946830	20993757
cis_mRNA_up10k	NA	NONHSAT183385.1	MTCONS_00112427	chr2	198365336	198368089	chr2	198351308	198364640
cis_mRNA_up10k	NA	NONHSAT183385.1	MTCONS_00112431	chr2	198365336	198368089	chr2	198357761	198364640
cis_mRNA_up10k	NA	NONHSAT183385.1	MTCONS_00112426	chr2	198365336	198368089	chr2	198351308	198364347
cis_mRNA_overlap	Lnc-CompleteIn-	NONHSAT183935.1	MTCONS_00106016	chr2	241532849	241533049	chr2	241526133	241538526
cis_mRNA_dw20k	NA	NONHSAT184130.1	NM_001256477	chr2	6972134	6976264	chr2	6990781	7005950
cis_mRNA_up10k	NA	NONHSAT184183.1	NM_002166	chr2	8819939	8821991	chr2	8822113	8824583
cis_mRNA_overlap	Lnc-CompleteIn-	NONHSAT184234.1	MTCONS_00106846	chr2	11361392	11375878	chr2	11321778	11428624
tran	NA	NONHSAT184341.	MTCONS_0009079	chr2	23451114	23453536	chr19	20946830	20993757
cis_mRNA_up10k	NA	NONHSAT184439.1	NM_017964	chr2	32390115	32390826	chr2	32390910	32449181
tran	NA	NONHSAT184463.	MTCONS_0000153	chr2	36925545	36925959	chr1	28696093	28826881
tran	NA	NONHSAT184508.	MTCONS_0003277	chr2	39039935	39040989	chr11	129685741	129733498
cis_mRNA_overlap	Lnc-AntiOverlap-mRNA	NONHSAT184551.1	NM_177968	chr2	44394764	44395998	chr2	44395942	44461742
tran	NA	NONHSAT184658.	MTCONS_0001971	chr2	56043830	56127965	chr10	32735010	32774488
tran	NA	NONHSAT184690.	MTCONS_0009079	chr2	60431234	60433655	chr19	20946830	20993757

cis_mRNA_overlap	Lnc-CompleteIn-	NONHSAT184743.1	MTCONS_00108418	chr2	64119279	64139723	chr2	64119279	64246593
cis_mRNA_overlap	Lnc-Overlap-mRNA	NONHSAT184743.1	NM_001005739	chr2	64119279	64139723	chr2	64119667	64246214
cis_mRNA_up10k	NA	NONHSAT184874.1	NM_152792	chr2	70190592	70243473	chr2	70187223	70189397
tran	NA	NONHSAT184930.	MTCONS_0003253	chr2	70283324	70313352	chr11	123986111	124017618
tran	NA	NONHSAT184930.	MTCONS_0007994	chr2	70283324	70313352	chr17	1472548	1531669
cis_mRNA_overlap	Lnc-Overlap-mRNA	NONHSAT185078.1	MTCONS_00109361	chr2	89156675	89266254	chr2	89156608	89385208
cis_mRNA_overlap	Lnc-Overlap-mRNA	NONHSAT185078.1	MTCONS_00109362	chr2	89156675	89266254	chr2	89156608	89442627
cis_mRNA_overlap	Lnc-Overlap-mRNA	NONHSAT185078.1	MTCONS_00109364	chr2	89156675	89266254	chr2	89156608	89442642
cis_mRNA_overlap	mRNA-AntiCompleteIn-LncIntron	NONHSAT185078.1	MTCONS_00100910	chr2	89156675	89266254	chr2	89184517	89185662
cis_mRNA_overlap	Lnc-Overlap-mRNA	NONHSAT185080.1	MTCONS_00109361	chr2	89156675	89417317	chr2	89156608	89385208
cis_mRNA_overlap	Lnc-Overlap-mRNA	NONHSAT185080.1	MTCONS_00109362	chr2	89156675	89417317	chr2	89156608	89442627
cis_mRNA_overlap	mRNA-AntiCompleteIn-LncIntron	NONHSAT185080.1	MTCONS_00100910	chr2	89156675	89417317	chr2	89184517	89185662
cis_mRNA_overlap	Lnc-Overlap-mRNA	NONHSAT185082.1	MTCONS_00109363	chr2	89156676	89385204	chr2	89156608	89442642
cis_mRNA_overlap	Lnc-Overlap-mRNA	NONHSAT185082.1	MTCONS_00109364	chr2	89156676	89385204	chr2	89156608	89442642
cis_mRNA_overlap	Lnc-Overlap-mRNA	NONHSAT185082.1	MTCONS_00109366	chr2	89156676	89385204	chr2	89156608	89442664
cis_mRNA_overlap	Lnc-Overlap-mRNA	NONHSAT185084.1	MTCONS_00109359	chr2	89156678	89385199	chr2	89156608	89247335
cis_mRNA_overlap	Lnc-Overlap-mRNA	NONHSAT185084.1	MTCONS_00109361	chr2	89156678	89385199	chr2	89156608	89385208

cis_mRNA_overlap	Lnc-Overlap-mRNA	NONHSAT185084.1	MTCONS_00109362	chr2	89156678	89385199	chr2	89156608	89442627
cis_mRNA_overlap	Lnc-Overlap-mRNA	NONHSAT185084.1	MTCONS_00109363	chr2	89156678	89385199	chr2	89156608	89442642
cis_mRNA_overlap	Lnc-Overlap-mRNA	NONHSAT185084.1	MTCONS_00109364	chr2	89156678	89385199	chr2	89156608	89442642
cis_mRNA_overlap	Lnc-Overlap-mRNA	NONHSAT185084.1	MTCONS_00109365	chr2	89156678	89385199	chr2	89156608	89442664
cis_mRNA_overlap	Lnc-Overlap-mRNA	NONHSAT185084.1	MTCONS_00109366	chr2	89156678	89385199	chr2	89156608	89442664
cis_mRNA_overlap	mRNA-AntiCompleteIn-LncIntron	NONHSAT185084.1	MTCONS_00100910	chr2	89156678	89385199	chr2	89184517	89185662
cis_mRNA_overlap	Lnc-Overlap-mRNA	NONHSAT185087.1	MTCONS_00109361	chr2	89156680	89266272	chr2	89156608	89385208
cis_mRNA_overlap	Lnc-Overlap-mRNA	NONHSAT185087.1	MTCONS_00109362	chr2	89156680	89266272	chr2	89156608	89442627
cis_mRNA_overlap	Lnc-Overlap-mRNA	NONHSAT185087.1	MTCONS_00109363	chr2	89156680	89266272	chr2	89156608	89442642
cis_mRNA_overlap	Lnc-Overlap-mRNA	NONHSAT185087.1	MTCONS_00109364	chr2	89156680	89266272	chr2	89156608	89442642
cis_mRNA_overlap	Lnc-Overlap-mRNA	NONHSAT185087.1	MTCONS_00109366	chr2	89156680	89266272	chr2	89156608	89442664
cis_mRNA_overlap	mRNA-AntiCompleteIn-LncIntron	NONHSAT185087.1	MTCONS_00100910	chr2	89156680	89266272	chr2	89184517	89185662
cis_mRNA_overlap	Lnc-Overlap-mRNA	NONHSAT185088.1	MTCONS_00109361	chr2	89156680	89327201	chr2	89156608	89385208
cis_mRNA_overlap	Lnc-Overlap-mRNA	NONHSAT185088.1	MTCONS_00109362	chr2	89156680	89327201	chr2	89156608	89442627
cis_mRNA_overlap	Lnc-Overlap-mRNA	NONHSAT185088.1	MTCONS_00109363	chr2	89156680	89327201	chr2	89156608	89442642
cis_mRNA_overlap	Lnc-Overlap-mRNA	NONHSAT185088.1	MTCONS_00109364	chr2	89156680	89327201	chr2	89156608	89442642

cis_mRNA_overlap	mRNA-AntiCompleteIn-LncIntron	NONHSAT185088.1	MTCONS_00100910	chr2	89156680	89327201	chr2	89184517	89185662
cis_mRNA_overlap	Lnc-Overlap-mRNA	NONHSAT185094.1	MTCONS_00109361	chr2	89156680	89476611	chr2	89156608	89385208
cis_mRNA_overlap	Lnc-Overlap-mRNA	NONHSAT185094.1	MTCONS_00109362	chr2	89156680	89476611	chr2	89156608	89442627
cis_mRNA_overlap	Lnc-Overlap-mRNA	NONHSAT185094.1	MTCONS_00109364	chr2	89156680	89476611	chr2	89156608	89442642
cis_mRNA_overlap	mRNA-AntiCompleteIn-LncIntron	NONHSAT185094.1	MTCONS_00100910	chr2	89156680	89476611	chr2	89184517	89185662
cis_mRNA_overlap	Lnc-Overlap-mRNA	NONHSAT185095.1	MTCONS_00109359	chr2	89156680	89513381	chr2	89156608	89247335
cis_mRNA_overlap	Lnc-Overlap-mRNA	NONHSAT185095.1	MTCONS_00109360	chr2	89156680	89513381	chr2	89156608	89340219
cis_mRNA_overlap	mRNA-AntiCompleteIn-LncIntron	NONHSAT185095.1	MTCONS_00100910	chr2	89156680	89513381	chr2	89184517	89185662
cis_mRNA_overlap	Lnc-Overlap-mRNA	NONHSAT185096.1	MTCONS_00109359	chr2	89156680	89568244	chr2	89156608	89247335
cis_mRNA_overlap	Lnc-Overlap-mRNA	NONHSAT185096.1	MTCONS_00109360	chr2	89156680	89568244	chr2	89156608	89340219
cis_mRNA_overlap	Lnc-Overlap-mRNA	NONHSAT185096.1	MTCONS_00109361	chr2	89156680	89568244	chr2	89156608	89385208
cis_mRNA_overlap	Lnc-Overlap-mRNA	NONHSAT185096.1	MTCONS_00109362	chr2	89156680	89568244	chr2	89156608	89442627
cis_mRNA_overlap	Lnc-Overlap-mRNA	NONHSAT185096.1	MTCONS_00109363	chr2	89156680	89568244	chr2	89156608	89442642
cis_mRNA_overlap	Lnc-Overlap-mRNA	NONHSAT185096.1	MTCONS_00109364	chr2	89156680	89568244	chr2	89156608	89442642
cis_mRNA_overlap	Lnc-Overlap-mRNA	NONHSAT185096.1	MTCONS_00109366	chr2	89156680	89568244	chr2	89156608	89442664
cis_mRNA_overlap	Lnc-Overlap-mRNA	NONHSAT185097.1	MTCONS_00109359	chr2	89156680	89619876	chr2	89156608	89247335

cis_mRNA_overlap	Lnc-Overlap-mRNA	NONHSAT185097.1	MTCONS_00109361	chr2	89156680	89619876	chr2	89156608	89385208
cis_mRNA_overlap	Lnc-Overlap-mRNA	NONHSAT185097.1	MTCONS_00109362	chr2	89156680	89619876	chr2	89156608	89442627
cis_mRNA_overlap	Lnc-Overlap-mRNA	NONHSAT185097.1	MTCONS_00109363	chr2	89156680	89619876	chr2	89156608	89442642
cis_mRNA_overlap	Lnc-Overlap-mRNA	NONHSAT185097.1	MTCONS_00109364	chr2	89156680	89619876	chr2	89156608	89442642
cis_mRNA_overlap	Lnc-Overlap-mRNA	NONHSAT185097.1	MTCONS_00109366	chr2	89156680	89619876	chr2	89156608	89442664
cis_mRNA_overlap	mRNA-AntiCompleteIn-LncIntron	NONHSAT185097.1	MTCONS_00100910	chr2	89156680	89619876	chr2	89184517	89185662
cis_mRNA_overlap	Lnc-Overlap-mRNA	NONHSAT185102.1	MTCONS_00109361	chr2	89156682	89327186	chr2	89156608	89385208
cis_mRNA_overlap	Lnc-Overlap-mRNA	NONHSAT185102.1	MTCONS_00109362	chr2	89156682	89327186	chr2	89156608	89442627
cis_mRNA_overlap	mRNA-AntiCompleteIn-LncIntron	NONHSAT185102.1	MTCONS_00100910	chr2	89156682	89327186	chr2	89184517	89185662
cis_mRNA_overlap	Lnc-Overlap-mRNA	NONHSAT185107.1	MTCONS_00109363	chr2	89156683	89399830	chr2	89156608	89442642
cis_mRNA_overlap	Lnc-Overlap-mRNA	NONHSAT185108.1	MTCONS_00109359	chr2	89156688	89327205	chr2	89156608	89247335
cis_mRNA_overlap	Lnc-Overlap-mRNA	NONHSAT185108.1	MTCONS_00109361	chr2	89156688	89327205	chr2	89156608	89385208
cis_mRNA_overlap	Lnc-Overlap-mRNA	NONHSAT185108.1	MTCONS_00109362	chr2	89156688	89327205	chr2	89156608	89442627
cis_mRNA_overlap	Lnc-Overlap-mRNA	NONHSAT185108.1	MTCONS_00109363	chr2	89156688	89327205	chr2	89156608	89442642
cis_mRNA_overlap	Lnc-Overlap-mRNA	NONHSAT185108.1	MTCONS_00109364	chr2	89156688	89327205	chr2	89156608	89442642
cis_mRNA_overlap	Lnc-Overlap-mRNA	NONHSAT185108.1	MTCONS_00109366	chr2	89156688	89327205	chr2	89156608	89442664

cis_mRNA overlap	Lnc-Overlap-mRNA	NONHSAT185131.1	MTCONS_00109555	chr2	96557405	96561770	chr2	96501067	96571748
cis_mRNA overlap	Lnc-CompleteIn-	NONHSAT185133.1	MTCONS_00109555	chr2	96561181	96568469	chr2	96501067	96571748
cis_mRNA overlap	Lnc-Overlap-mRNA	NONHSAT185133.1	MTCONS_00109578	chr2	96561181	96568469	chr2	96501067	96658068
cis_mRNA dw20k	NA	NONHSAT185202.1	MTCONS_00109834	chr2	101887694	101890620	chr2	101892063	101925178
cis_mRNA dw20k	NA	NONHSAT185203.1	NM_017546	chr2	101892085	101905517	chr2	101869345	101886778
cis_mRNA overlap	Lnc-Overlap-mRNA	NONHSAT185204.1	MTCONS_00109834	chr2	101893001	101899277	chr2	101892063	101925178
cis_mRNA _overlap	Lnc-AntiOverlap-mRNA	NONHSAT185329.1	MTCONS_00101657	chr2	110595035	110615987	chr2	110550335	110615282
cis_mRNA overlap	Lnc-CompleteIn-	NONHSAT185540.1	MTCONS_00110676	chr2	130914268	130914588	chr2	130914268	130939330
cis_mRNA overlap	Lnc-Overlap-mRNA	NONHSAT185616.1	MTCONS_00110934	chr2	145162450	145164222	chr2	145141942	145277958
cis_mRNA overlap	Lnc-CompleteIn-	NONHSAT185717.1	MTCONS_00111174	chr2	157188322	157188942	chr2	157180944	157190970
cis_mRNA overlap	Lnc-CompleteIn-	NONHSAT185717.1	MTCONS_00111177	chr2	157188322	157188942	chr2	157183980	157189287
cis_mRNA overlap	Lnc-CompleteIn-	NONHSAT185926.1	NM_001256092	chr2	175976303	176032897	chr2	175936978	176032934
cis_mRNA overlap	Lnc-Overlap-mRNA	NONHSAT186058.1	NM_001032281	chr2	188393413	188419157	chr2	188343306	188419219
cis_mRNA overlap	Lnc-Overlap-mRNA	NONHSAT186070.1	MTCONS_00112248	chr2	190616847	190619106	chr2	190611386	190627924
cis_mRNA up10k	NA	NONHSAT186073.1	NM_000534	chr2	190636494	190648700	chr2	190648811	190742355
cis_mRNA up10k	NA	NONHSAT186104.1	MTCONS_00104296	chr2	192541521	192542559	chr2	192542798	192553248
cis_mRNA overlap	Lnc-CompleteIn-	NONHSAT186120.1	NM_004226	chr2	197001635	197008379	chr2	196998307	197036336

cis_mRNA dw20k	NA	NONHSAT186127.1	MTCONS_00112431	chr2	198353205	198355410	chr2	198357761	198364640
cis_mRNA overlap	Lnc-Overlap-mRNA	NONHSAT186127.1	MTCONS_00112426	chr2	198353205	198355410	chr2	198351308	198364347
cis_mRNA overlap	Lnc-Overlap-mRNA	NONHSAT186127.1	MTCONS_00112427	chr2	198353205	198355410	chr2	198351308	198364640
cis_mRNA up10k	NA	NONHSAT186127.1	NM_002157	chr2	198353205	198355410	chr2	198364721	198368187
cis_mRNA dw20k	NA	NONHSAT186437.1	NM_024080	chr2	234934080	234936343	chr2	234826043	234928166
cis_mRNA up10k	NA	NONHSAT186675.1	NM_182626	chr2	10273758	10276016	chr2	10281509	10351856
cis_mRNA dw20k	NA	NONHSAT186779.1	NM_001127401	chr2	30393845	30405956	chr2	30369750	30383399
cis_mRNA dw20k	NA	NONHSAT186801.1	NM_016252	chr2	32846858	32848838	chr2	32582096	32843965
tran	NA	NONHSAT186893.	MTCONS_0014142	chr2	48143654	48144367	chr4	2965230	2972037
cis_mRNA dw20k	NA	NONHSAT187247.1	MTCONS_00101390	chr2	102658334	102658880	chr2	102607381	102644884
tran	NA	NONHSAT187338.	MTCONS_0018041	chr2	113616274	113622751	chr7	155437203	155480457
tran	NA	NONHSAT187381.	MTCONS_0009079	chr2	118282357	118283372	chr19	20946830	20993757
tran	NA	NONHSAT187381.	MTCONS_0019546	chr2	118282357	118283372	chr9	20658308	20953758
cis_mRNA dw20k	NA	NONHSAT188134.1	MTCONS_00105432	chr2	228439262	228440366	chr2	228336888	228425938
cis_mRNA dw20k	NA	NONHSAT188134.1	NM_004504	chr2	228439262	228440366	chr2	228336888	228425938
cis_mRNA dw20k	NA	NONHSAT188357.1	MTCONS_00118200	chr20	1559583	1577118	chr20	1577986	1600689
cis_mRNA dw20k	NA	NONHSAT188357.1	NM_001135844	chr20	1559583	1577118	chr20	1577986	1600689
cis_mRNA _overlap	Lnc-AntiOverlap-mRNA	NONHSAT188357.1	MTCONS_00118191	chr20	1559583	1577118	chr20	1542360	1569380
cis_mRNA overlap	Lnc-Overlap-mRNA	NONHSAT188425.1	MTCONS_00115781	chr20	8113023	8352998	chr20	8112912	8375809

cis_mRNA overlap	Lnc-Overlap-mRNA	NONHSAT188425.1	MTCONS_00115782	chr20	8113023	8352998	chr20	8112912	8634200
cis_mRNA dw20k	NA	NONHSAT188618.1	MTCONS_00116315	chr20	25209786	25212569	chr20	25176306	25207365
tran	NA	NONHSAT188633.1	MTCONS_00203761	chr20	29611898	29632648	chrUn_gl000241	16574	36886
cis_mRNA overlap	Lnc-Overlap-mRNA	NONHSAT188753.1	MTCONS_00116811	chr20	39708718	39713737	chr20	39657462	39753126
cis_mRNA overlap	Lnc-CompleteIn-	NONHSAT188874.1	NM_006420	chr20	47538280	47570225	chr20	47538275	47653230
cis_mRNA dw20k	NA	NONHSAT189171.1	MTCONS_00118015	chr20	62328024	62330029	chr20	62289163	62327610
cis_mRNA dw20k	NA	NONHSAT189173.1	NM_001305655	chr20	62371652	62374856	chr20	62367812	62370460
cis_mRNA overlap	Lnc-Overlap-mRNA	NONHSAT189173.1	MTCONS_00118041	chr20	62371652	62374856	chr20	62371211	62378647
cis_mRNA overlap	Lnc-Overlap-mRNA	NONHSAT189173.1	NM_020062	chr20	62371652	62374856	chr20	62371211	62375403
cis_mRNA _overlap	Lnc-AntiOverlap-mRNA	NONHSAT189438.1	MTCONS_00116127	chr20	21283369	21284342	chr20	21283922	21370463
cis_mRNA _overlap	Lnc-AntiOverlap-mRNA	NONHSAT189438.1	MTCONS_00116128	chr20	21283369	21284342	chr20	21283922	21370463
cis_mRNA overlap	Lnc-Overlap-mRNA	NONHSAT189609.1	NM_004902	chr20	34328138	34330159	chr20	34291531	34330258
cis_mRNA overlap	Lnc-Overlap-mRNA	NONHSAT189609.1	NM_184234	chr20	34328138	34330159	chr20	34291531	34330258
cis_mRNA overlap	Lnc-CompleteIn-	NONHSAT189704.1	NM_016470	chr20	42825548	42839376	chr20	42824581	42839546
cis_mRNA dw20k	NA	NONHSAT189734.1	NM_033550	chr20	45310315	45311014	chr20	45313004	45318276
cis_mRNA up10k	NA	NONHSAT189972.1	MTCONS_00120593	chr20	61559973	61560649	chr20	61509090	61557903

tran	NA	NONHSAT190254.	MTCONS_0003154	chr20	32287508	32288316	chr11	89585943	89596997
cis_mRNA overlap	Lnc-Overlap-mRNA	NONHSAT190700.1	MTCONS_00121368	chr21	17163815	17178122	chr21	17102344	17178122
cis_mRNA overlap	Lnc-CompleteIn-	NONHSAT190829.1	NM_006447	chr21	30419523	30426804	chr21	30396938	30426807
tran	NA	NONHSAT190846.	MTCONS_0003277	chr21	32687310	32688140	chr11	129685741	129733498
cis_mRNA overlap	Lnc-CompleteIn-	NONHSAT190964.1	MTCONS_00122110	chr21	39607686	39623237	chr21	39601837	39673746
cis_mRNA overlap	Lnc-CompleteIn-	NONHSAT190964.1	NM_001276438	chr21	39607686	39623237	chr21	39601837	39673746
cis_mRNA overlap	Lnc-CompleteIn-	NONHSAT190964.1	NM_001276439	chr21	39607686	39623237	chr21	39601837	39673746
cis_mRNA up10k	NA	NONHSAT190964.1	NM_001276436	chr21	39607686	39623237	chr21	39628620	39673746
cis_mRNA overlap	Lnc-CompleteIn-	NONHSAT190965.1	MTCONS_00122110	chr21	39616429	39616741	chr21	39601837	39673746
cis_mRNA dw20k	NA	NONHSAT191118.1	MTCONS_00122705	chr21	48001264	48004500	chr21	47878862	47989926
cis_mRNA dw20k	NA	NONHSAT191118.1	MTCONS_00122713	chr21	48001264	48004500	chr21	47948346	47989926
cis_mRNA overlap	Lnc-CompleteIn-	NONHSAT191119.1	NM_001242866	chr21	48069495	48079434	chr21	48055507	48081190
cis_mRNA overlap	Lnc-Overlap-mRNA	NONHSAT191119.1	NM_001242865	chr21	48069495	48079434	chr21	48055507	48075009
cis_mRNA overlap	Lnc-Overlap-mRNA	NONHSAT191119.1	NM_206962	chr21	48069495	48079434	chr21	48055507	48085036
tran	NA	NONHSAT191252.	MTCONS_0001971	chr21	18129204	18179796	chr10	32735010	32774488
cis_mRNA overlap	Lnc-Overlap-mRNA	NONHSAT191260.1	MTCONS_00122959	chr21	19165124	19191646	chr21	19149721	19191703
cis_mRNA dw20k	NA	NONHSAT191353.1	NM_015565	chr21	30291159	30296988	chr21	30300466	30365277
cis_mRNA overlap	Lnc-Overlap-mRNA	NONHSAT191354.1	MTCONS_00123203	chr21	30329101	30332367	chr21	30316687	30365277
tran	NA	NONHSAT191362.	MTCONS_0009723	chr21	30742380	30748659	chr19	55823934	55836708

cis_mRNA up10k	NA	NONHSAT191588.1	NM_001316984	chr21	46349568	46350027	chr21	46359912	46396904
cis_mRNA dw20k	NA	NONHSAT191613.1	MTCONS_00122683	chr21	47874901	47878594	chr21	47744036	47865682
cis_mRNA dw20k	NA	NONHSAT191613.1	MTCONS_00122696	chr21	47874901	47878594	chr21	47844767	47865682
cis_mRNA overlap	Lnc-CompleteIn-	NONHSAT192002.1	NM_003776	chr22	19420057	19423593	chr22	19419425	19423601
cis_mRNA overlap	Lnc-Overlap-mRNA	NONHSAT192002.1	NM_001318152	chr22	19420057	19423593	chr22	19420462	19423601
cis_mRNA overlap	Lnc-CompleteIn-	NONHSAT192037.1	MTCONS_00124760	chr22	21305083	21308034	chr22	21271714	21308037
cis_mRNA overlap	Lnc-Overlap-mRNA	NONHSAT192065.1	MTCONS_00124978	chr22	22676840	23243612	chr22	23054878	23248991
cis_mRNA overlap	Lnc-Overlap-mRNA	NONHSAT192068.1	MTCONS_00124919	chr22	22712120	23248959	chr22	22712030	22713114
cis_mRNA overlap	Lnc-Overlap-mRNA	NONHSAT192070.1	MTCONS_00124910	chr22	22712124	23243614	chr22	22676774	23248991
cis_mRNA overlap	Lnc-Overlap-mRNA	NONHSAT192070.1	MTCONS_00124925	chr22	22712124	23243614	chr22	22723978	23262165
cis_mRNA overlap	Lnc-Overlap-mRNA	NONHSAT192070.1	MTCONS_00124997	chr22	22712124	23243614	chr22	23101176	23248991
cis_mRNA overlap	Lnc-Overlap-mRNA	NONHSAT192072.1	MTCONS_00124978	chr22	22735221	23248968	chr22	23054878	23248991
cis_mRNA overlap	Lnc-CompleteIn-	NONHSAT192073.1	MTCONS_00124925	chr22	22735224	23243610	chr22	22723978	23262165
cis_mRNA overlap	Lnc-Overlap-mRNA	NONHSAT192078.1	MTCONS_00124910	chr22	22749369	23248968	chr22	22676774	23248991
cis_mRNA overlap	Lnc-Overlap-mRNA	NONHSAT192078.1	MTCONS_00124925	chr22	22749369	23248968	chr22	22723978	23262165
cis_mRNA overlap	Lnc-Overlap-mRNA	NONHSAT192078.1	MTCONS_00124975	chr22	22749369	23248968	chr22	23054765	23248991
cis_mRNA overlap	Lnc-Overlap-mRNA	NONHSAT192080.1	MTCONS_00124910	chr22	22749377	23248969	chr22	22676774	23248991

cis_mRNA overlap	Lnc-Overlap-mRNA	NONHSAT192080.1	MTCONS_00124925	chr22	22749377	23248969	chr22	22723978	23262165
cis_mRNA overlap	Lnc-Overlap-mRNA	NONHSAT192081.1	MTCONS_00124910	chr22	22764115	23248970	chr22	22676774	23248991
cis_mRNA overlap	Lnc-Overlap-mRNA	NONHSAT192081.1	MTCONS_00124981	chr22	22764115	23248970	chr22	23063080	23248991
cis_mRNA overlap	Lnc-Overlap-mRNA	NONHSAT192081.1	MTCONS_00124997	chr22	22764115	23248970	chr22	23101176	23248991
cis_mRNA overlap	Lnc-Overlap-mRNA	NONHSAT192081.1	MTCONS_00125020	chr22	22764115	23248970	chr22	23222869	23248991
cis_mRNA _overlap	mRNA-CompleteIn-LncIntron	NONHSAT192084.1	NM_001256296	chr22	22764146	23248970	chr22	23229960	23238013
cis_mRNA overlap	Lnc-CompleteIn-	NONHSAT192088.1	MTCONS_00124925	chr22	23029181	23243614	chr22	22723978	23262165
cis_mRNA overlap	Lnc-Overlap-mRNA	NONHSAT192088.1	MTCONS_00124910	chr22	23029181	23243614	chr22	22676774	23248991
cis_mRNA overlap	Lnc-Overlap-mRNA	NONHSAT192088.1	MTCONS_00124975	chr22	23029181	23243614	chr22	23054765	23248991
cis_mRNA overlap	Lnc-Overlap-mRNA	NONHSAT192088.1	MTCONS_00124982	chr22	23029181	23243614	chr22	23063114	23248991
cis_mRNA overlap	Lnc-Overlap-mRNA	NONHSAT192088.1	MTCONS_00124992	chr22	23029181	23243614	chr22	23101100	23248991
cis_mRNA overlap	Lnc-Overlap-mRNA	NONHSAT192088.1	MTCONS_00124997	chr22	23029181	23243614	chr22	23101176	23248991
cis_mRNA overlap	Lnc-Overlap-mRNA	NONHSAT192088.1	MTCONS_00125020	chr22	23029181	23243614	chr22	23222869	23248991
cis_mRNA _overlap	mRNA-CompleteIn-LncIntron	NONHSAT192091.1	MTCONS_00124996	chr22	23029211	23248968	chr22	23101176	23238013
cis_mRNA overlap	Lnc-Overlap-mRNA	NONHSAT192098.1	MTCONS_00124982	chr22	23054881	23248972	chr22	23063114	23248991
cis_mRNA overlap	Lnc-Overlap-mRNA	NONHSAT192098.1	MTCONS_00125020	chr22	23054881	23248972	chr22	23222869	23248991

cis_mRNA _overlap	mRNA-CompleteIn-LncIntron	NONHSAT192098.1	NM_001256296	chr22	23054881	23248972	chr22	23229960	23238013
cis_mRNA _overlap	mRNA-CompleteIn-LncIntron	NONHSAT192100.1	MTCONS_00124995	chr22	23063122	23248970	chr22	23101176	23238013
cis_mRNA overlap	Lnc-Overlap-mRNA	NONHSAT192102.1	MTCONS_00125020	chr22	23063128	23238006	chr22	23222869	23248991
cis_mRNA overlap	Lnc-Overlap-mRNA	NONHSAT192105.1	MTCONS_00124975	chr22	23089885	23248952	chr22	23054765	23248991
cis_mRNA overlap	Lnc-Overlap-mRNA	NONHSAT192113.1	MTCONS_00125011	chr22	23134993	23248967	chr22	23165261	23248991
cis_mRNA overlap	Lnc-CompleteIn-	NONHSAT192114.1	MTCONS_00124992	chr22	23134997	23238009	chr22	23101100	23248991
cis_mRNA overlap	Lnc-Overlap-mRNA	NONHSAT192116.1	MTCONS_00124996	chr22	23161599	23248966	chr22	23101176	23238013
cis_mRNA overlap	Lnc-Overlap-mRNA	NONHSAT192155.1	NM_001145206	chr22	25584597	25591749	chr22	25423941	25593415
cis_mRNA overlap	Lnc-CompleteIn-	NONHSAT192176.1	NM_001184774	chr22	26645389	26650752	chr22	26565440	26779563
cis_mRNA overlap	Lnc-CompleteIn-	NONHSAT192229.1	NM_001163285	chr22	29682947	29696510	chr22	29663998	29696515
cis_mRNA up10k	NA	NONHSAT192229.1	NM_001278730	chr22	29682947	29696510	chr22	29702985	29708778
cis_mRNA up10k	NA	NONHSAT192256.1	NM_032050	chr22	31742745	31747141	chr22	31721790	31742249
cis_mRNA dw20k	NA	NONHSAT192350.1	NM_033386	chr22	38339057	38340905	chr22	38302155	38338465
cis_mRNA dw20k	NA	NONHSAT192351.1	NM_033386	chr22	38344359	38398896	chr22	38302155	38338465
cis_mRNA _overlap	Lnc-AntiOverlap-mRNA	NONHSAT192354.1	MTCONS_00127899	chr22	38526223	38546619	chr22	38507140	38577841
cis_mRNA dw20k	NA	NONHSAT192383.1	NM_001291824	chr22	40378071	40380750	chr22	40322595	40369346

cis_mRNA overlap	Lnc-CompleteIn-	NONHSAT192387.1	MTCONS_00126080	chr22	40804959	40806104	chr22	40766566	41188085
cis_mRNA overlap	Lnc-Overlap-mRNA	NONHSAT192387.1	NM_015705	chr22	40804959	40806104	chr22	40766566	40806293
cis_mRNA overlap	Lnc-Overlap-mRNA	NONHSAT192405.1	NM_014248	chr22	41347400	41368666	chr22	41347351	41369019
cis_mRNA dw20k	NA	NONHSAT192406.1	NM_022098	chr22	41347412	41368666	chr22	41253085	41328823
cis_mRNA dw20k	NA	NONHSAT192411.1	NM_016272	chr22	41809657	41816621	chr22	41829492	41843027
cis_mRNA overlap	Lnc-Overlap-mRNA	NONHSAT192460.1	NM_001137605	chr22	44577216	44583247	chr22	44568836	44604349
cis_mRNA up10k	NA	NONHSAT192576.1	MTCONS_00128455	chr22	50683502	50685441	chr22	50656118	50683400
cis_mRNA overlap	Lnc-Overlap-mRNA	NONHSAT192633.1	NM_001166242	chr22	19428417	19431789	chr22	19428410	19435755
tran	NA	NONHSAT192675.	NM_001130111	chr22	22469043	22472752	chr19	1876975	1885518
cis_mRNA overlap	Lnc-CompleteIn-	NONHSAT192929.1	MTCONS_00128023	chr22	40931376	40933932	chr22	40806285	41032723
tran	NA	NONHSAT192929.	MTCONS_0011279	chr22	40931376	40933932	chr2	208470297	208490028
cis_mRNA _overlap	Lnc-AntiOverlap-mRNA	NONHSAT193048.1	MTCONS_00126588	chr22	50637528	50639286	chr22	50624341	50638028
cis_mRNA up10k	NA	NONHSAT193048.1	MTCONS_00126593	chr22	50637528	50639286	chr22	50639408	50656128
cis_mRNA overlap	Lnc-Overlap-mRNA	NONHSAT193052.1	NM_001113756	chr22	50964185	50964817	chr22	50964182	50968514
cis_mRNA overlap	Lnc-CompleteIn-	NONHSAT193363.1	NM_213720	chr22	24108021	24110159	chr22	24108021	24110159
tran	NA	NONHSAT193410.	MTCONS_0009310	chr3	5154933	5284661	chr19	55417508	55445875
cis_mRNA overlap	Lnc-CompleteIn-	NONHSAT193516.1	MTCONS_00129263	chr3	15273083	15273692	chr3	15247733	15306005
tran	NA	NONHSAT193516.	MTCONS_0003277	chr3	15273083	15273692	chr11	129685741	129733498
cis_mRNA dw20k	NA	NONHSAT193528.1	MTCONS_00129311	chr3	16380815	16381378	chr3	16306667	16364867

cis_mRNA dw20k	NA	NONHSAT193530.1	MTCONS_00129314	chr3	16387694	16391035	chr3	16306667	16378866
cis_mRNA overlap	Lnc-CompleteIn-	NONHSAT193614.1	NM_004162	chr3	19988691	20017636	chr3	19988572	20026667
cis_mRNA overlap	Lnc-Overlap-mRNA	NONHSAT193676.1	NM_182523	chr3	28283127	28361261	chr3	28283124	28361263
cis_mRNA overlap	Lnc-Overlap-mRNA	NONHSAT193698.1	NM_003242	chr3	30711945	30715793	chr3	30647994	30735633
cis_mRNA up10k	NA	NONHSAT193717.1	NM_001128160	chr3	33488084	33497653	chr3	33429828	33481870
cis_mRNA overlap	Lnc-Overlap-mRNA	NONHSAT193738.1	MTCONS_00129841	chr3	37278088	37295877	chr3	37284682	37408370
cis_mRNA overlap	Lnc-CompleteIn-	NONHSAT193791.1	MTCONS_00130049	chr3	42642178	42661905	chr3	42642147	42690233
cis_mRNA overlap	Lnc-CompleteIn-	NONHSAT193791.1	MTCONS_00130052	chr3	42642178	42661905	chr3	42642147	42690233
cis_mRNA overlap	Lnc-Overlap-mRNA	NONHSAT193791.1	MTCONS_00130051	chr3	42642178	42661905	chr3	42642147	42690233
cis_mRNA up10k	NA	NONHSAT193840.1	NM_003965	chr3	46434250	46448624	chr3	46448721	46451014
cis_mRNA_overlap	Lnc-AntiCompleteIn-mRNAIntron	NONHSAT193854.1	NM_004157	chr3	48860310	48868978	chr3	48788093	48885270
cis_mRNA up10k	NA	NONHSAT193899.1	NM_015512	chr3	52338543	52346694	chr3	52350335	52434513
cis_mRNA overlap	Lnc-Overlap-mRNA	NONHSAT193964.1	NM_001291941	chr3	62304742	62319316	chr3	62304648	62321888
cis_mRNA overlap	Lnc-Overlap-mRNA	NONHSAT193972.1	MTCONS_00130829	chr3	63898054	63973806	chr3	63850233	63993755
cis_mRNA overlap	Lnc-Overlap-mRNA	NONHSAT193974.1	MTCONS_00130825	chr3	63964738	63972864	chr3	63850233	63993755
cis_mRNA overlap	Lnc-Overlap-mRNA	NONHSAT193974.1	MTCONS_00130841	chr3	63964738	63972864	chr3	63953420	63993755
cis_mRNA overlap	Lnc-Overlap-mRNA	NONHSAT193974.1	MTCONS_00130842	chr3	63964738	63972864	chr3	63953420	63993755

tran	NA	NONHSAT194173.	MTCONS_0009079	chr3	87138431	87147852	chr19	20946830	20993757
cis_mRNA up10k	NA	NONHSAT194237. 1	MTCONS_0013155 7	chr3	101557620	101559164	chr3	101568358	101579869
cis_mRNA up10k	NA	NONHSAT194237. 1	MTCONS_0013155 8	chr3	101557620	101559164	chr3	101568358	101579869
cis_mRNA up10k	NA	NONHSAT194237. 1	MTCONS_0013156 1	chr3	101557620	101559164	chr3	101568862	101579869
tran	NA	NONHSAT194250.	MTCONS_0011100	chr3	103142393	103199460	chr2	148687966	148778463
cis_mRNA _overlap	Lnc-AntiOverlap-mRNA	NONHSAT194332. 1	NM_001164346	chr3	114070685	114107817	chr3	114033347	114343792
cis_mRNA dw20k	NA	NONHSAT194388. 1	NM_021082	chr3	121671834	121688928	chr3	121613171	121663034
tran	NA	NONHSAT194493.	MTCONS_0009079	chr3	132704550	132722386	chr19	20946830	20993757
cis_mRNA overlap	Lnc-CompleteIn-	NONHSAT194496. 1	NM_001134422	chr3	133305468	133307138	chr3	133292434	133309118
cis_mRNA overlap	Lnc-Overlap-mRNA	NONHSAT194496. 1	NM_001282763	chr3	133305468	133307138	chr3	133293278	133309118
tran	NA	NONHSAT194655.	MTCONS_0006921	chr3	151171806	151178685	chr16	22217592	22300066
cis_mRNA overlap	Lnc-Overlap-mRNA	NONHSAT194668. 1	NM_207295	chr3	152018104	152020586	chr3	152017194	152183569
cis_mRNA overlap	Lnc-Overlap-mRNA	NONHSAT194668. 1	NM_207297	chr3	152018104	152020586	chr3	152017194	152183569
cis_mRNA overlap	Lnc-Overlap-mRNA	NONHSAT194683. 1	MTCONS_0013303 0	chr3	152923369	152928502	chr3	152880001	152942579
tran	NA	NONHSAT194695.	MTCONS_0009079	chr3	153515154	153515679	chr19	20946830	20993757
tran	NA	NONHSAT194807.	MTCONS_0001236	chr3	163217660	163218586	chr1	89571816	89593307
tran	NA	NONHSAT194949.	MTCONS_0003277	chr3	179084734	179112714	chr11	129685741	129733498
tran	NA	NONHSAT194949.	MTCONS_0011121	chr3	179084734	179112714	chr2	158592839	158675709
tran	NA	NONHSAT194949.	NM_001267782	chr3	179084734	179112714	chr11	46417962	46615619
tran	NA	NONHSAT194949.	NM_052916	chr3	179084734	179112714	chr17	74138534	74236390
tran	NA	NONHSAT194949.	NM_130807	chr3	179084734	179112714	chr19	2071035	2096269
tran	NA	NONHSAT194949.	NM_198147	chr3	179084734	179112714	chr17	27887689	27894042
tran	NA	NONHSAT194949.	NM_201433	chr3	179084734	179112714	chr17	9813926	10101868

cis_mRNA_overlap	Lnc-CompleteIn-	NONHSAT195056.1	MTCONS_00133950	chr3	182598987	182600295	chr3	182511459	182639421
tran	NA	NONHSAT195096.	MTCONS_0009079	chr3	187131990	187132408	chr19	20946830	20993757
cis_mRNA_overlap	Lnc-Overlap-mRNA	NONHSAT195199.1	MTCONS_00134464	chr3	195402812	195404734	chr3	195384964	195460424
cis_mRNA_overlap	Lnc-AntiOverlap-mRNA	NONHSAT195361.1	MTCONS_00128880	chr3	5033119	5067434	chr3	5021097	5044868
cis_mRNA_overlap	Lnc-AntiOverlap-mRNA	NONHSAT195361.1	MTCONS_00128881	chr3	5033119	5067434	chr3	5021097	5044868
cis_mRNA_dw20k	NA	NONHSAT195362.1	MTCONS_00128880	chr3	5051525	5067470	chr3	5021097	5044868
cis_mRNA_dw20k	NA	NONHSAT195362.1	MTCONS_00128881	chr3	5051525	5067470	chr3	5021097	5044868
cis_mRNA_overlap	Lnc-CompleteIn-	NONHSAT195513.1	MTCONS_00135429	chr3	27427432	27439253	chr3	27414212	27498245
tran	NA	NONHSAT195515.	MTCONS_0013211	chr3	27847383	27850631	chr3	122296449	122357894
cis_mRNA_overlap	Lnc-CompleteIn-	NONHSAT195550.1	NM_017801	chr3	32524231	32544295	chr3	32522804	32544403
cis_mRNA_overlap	Lnc-Overlap-mRNA	NONHSAT195677.1	NM_014056	chr3	42825796	42845943	chr3	42824400	42846027
cis_mRNA_dw20k	NA	NONHSAT195794.1	NM_021237	chr3	53913524	53916119	chr3	53919226	53925989
cis_mRNA_overlap	Lnc-Overlap-mRNA	NONHSAT195795.1	NM_021237	chr3	53919094	53925829	chr3	53919226	53925989
cis_mRNA_dw20k	NA	NONHSAT195831.1	MTCONS_00130842	chr3	63993758	63994368	chr3	63953420	63993755
cis_mRNA_up10k	NA	NONHSAT195865.1	NM_006407	chr3	69105773	69129494	chr3	69134090	69155239
cis_mRNA_overlap	Lnc-Overlap-mRNA	NONHSAT196001.1	MTCONS_00137126	chr3	101085557	101118852	chr3	101043033	101232085
tran	NA	NONHSAT196051.	MTCONS_0012760	chr3	106969884	106971980	chr22	31835345	31889009
cis_mRNA_overlap	Lnc-CompleteIn-	NONHSAT196108.1	NM_138806	chr3	112667128	112679904	chr3	112641532	112693937

tran	NA	NONHSAT196117.	MTCONS_0009079	chr3	114252449	114264980	chr19	20946830	20993757
cis_mRNA overlap	Lnc-Overlap-mRNA	NONHSAT196118.1	MTCONS_00137523	chr3	114795125	114866105	chr3	114033347	114819227
tran	NA	NONHSAT196223.	MTCONS_0007205	chr3	127311242	127317209	chr16	1413206	1464721
cis_mRNA overlap	Lnc-Overlap-mRNA	NONHSAT196245.1	NM_001127195	chr3	128888331	128902737	chr3	128886658	128902810
cis_mRNA overlap	Lnc-Overlap-mRNA	NONHSAT196245.1	NM_001127196	chr3	128888331	128902737	chr3	128886658	128902810
cis_mRNA dw20k	NA	NONHSAT196359.1	NM_001039547	chr3	141692975	141868360	chr3	141876369	141944449
cis_mRNA overlap	Lnc-CompleteIn-	NONHSAT196378.1	NM_021105	chr3	146233582	146239701	chr3	146232967	146262628
cis_mRNA overlap	Lnc-Overlap-mRNA	NONHSAT196482.1	MTCONS_00138700	chr3	156865591	156867186	chr3	156865345	156878549
tran	NA	NONHSAT196554.	MTCONS_0018041	chr3	171154710	171167216	chr7	155437203	155480457
tran	NA	NONHSAT196683.	MTCONS_0003253	chr3	186264133	186272085	chr11	123986111	124017618
tran	NA	NONHSAT196735.	MTCONS_0011100	chr3	187133696	187133958	chr2	148687966	148778463
tran	NA	NONHSAT197267.	MTCONS_0009079	chr3	87394844	87396206	chr19	20946830	20993757
cis_mRNA dw20k	NA	NONHSAT197351.1	MTCONS_00131558	chr3	101586033	101586693	chr3	101568358	101579869
cis_mRNA dw20k	NA	NONHSAT197702.1	NM_007287	chr3	154905118	154906692	chr3	154797705	154901518
cis_mRNA up10k	NA	NONHSAT197872.1	MTCONS_00138956	chr3	169904672	169909597	chr3	169805368	169899537
cis_mRNA up10k	NA	NONHSAT197893.1	NM_001190942	chr3	172242496	172243202	chr3	172223298	172241297
cis_mRNA overlap	Lnc-CompleteIn-	NONHSAT198239.1	MTCONS_00141588	chr4	6784481	6843843	chr4	6784459	6885899
tran	NA	NONHSAT198657.	MTCONS_0009079	chr4	62624479	62626646	chr19	20946830	20993757
tran	NA	NONHSAT198707.	NM_002089	chr4	74735173	74736941	chr4	74962754	74964997
cis_mRNA dw20k	NA	NONHSAT198779.1	MTCONS_00143281	chr4	84389905	84391615	chr4	84377085	84383311
cis_mRNA overlap	Lnc-CompleteIn-	NONHSAT198799.1	MTCONS_00143345	chr4	87939592	87951284	chr4	87856154	87979070
tran	NA	NONHSAT198803.	MTCONS_0009079	chr4	88273499	88273744	chr19	20946830	20993757

cis_mRNA up10k	NA	NONHSAT198878.1	NM_181887	chr4	103749299	103809122	chr4	103715540	103748710
tran	NA	NONHSAT199082.	MTCONS_0009079	chr4	131361837	131365044	chr19	20946830	20993757
tran	NA	NONHSAT199389.	MTCONS_0009079	chr4	171091288	171129930	chr19	20946830	20993757
cis_mRNA dw20k	NA	NONHSAT199711.1	MTCONS_00146211	chr4	8356817	8360065	chr4	8363187	8430399
cis_mRNA overlap	Lnc-CompleteIn-	NONHSAT199896.1	NM_001100399	chr4	39955059	39959114	chr4	39824483	39979576
cis_mRNA overlap	Lnc-Overlap-mRNA	NONHSAT199899.1	MTCONS_00146745	chr4	40625759	40640209	chr4	40425272	40632826
cis_mRNA overlap	Lnc-CompleteIn-	NONHSAT200145.1	NM_016211	chr4	83753291	83761614	chr4	83739662	83812433
cis_mRNA dw20k	NA	NONHSAT200227.1	NM_002106	chr4	100863238	100863679	chr4	100869244	100871512
cis_mRNA overlap	Lnc-CompleteIn-	NONHSAT200227.1	NM_001278310	chr4	100863238	100863679	chr4	100817407	100867883
cis_mRNA overlap	Lnc-Overlap-mRNA	NONHSAT200244.1	NM_003340	chr4	103717136	103749207	chr4	103715540	103749105
cis_mRNA overlap	Lnc-Overlap-mRNA	NONHSAT200244.1	NM_181887	chr4	103717136	103749207	chr4	103715540	103748710
cis_mRNA overlap	Lnc-Overlap-mRNA	NONHSAT200244.1	NM_181892	chr4	103717136	103749207	chr4	103715540	103748340
cis_mRNA overlap	Lnc-Overlap-mRNA	NONHSAT200247.1	NM_003340	chr4	103717980	103748913	chr4	103715540	103749105
cis_mRNA overlap	Lnc-Overlap-mRNA	NONHSAT200247.1	NM_181887	chr4	103717980	103748913	chr4	103715540	103748710
cis_mRNA overlap	Lnc-Overlap-mRNA	NONHSAT200247.1	NM_181892	chr4	103717980	103748913	chr4	103715540	103748340
cis_mRNA dw20k	NA	NONHSAT200265.1	MTCONS_00147989	chr4	106924475	106943636	chr4	106962789	107237423
cis_mRNA overlap	Lnc-CompleteIn-	NONHSAT200267.1	MTCONS_00147999	chr4	107176904	107177924	chr4	107169649	107237423
cis_mRNA overlap	Lnc-Overlap-mRNA	NONHSAT200269.1	MTCONS_00147999	chr4	107215665	107237353	chr4	107169649	107237423

cis_mRNA overlap	Lnc-Overlap-mRNA	NONHSAT200272.1	NM_016269	chr4	108999416	109087899	chr4	108968701	109090112
cis_mRNA up10k	NA	NONHSAT200453.1	NM_031296	chr4	140369597	140374930	chr4	140374961	140397069
cis_mRNA overlap	Lnc-Overlap-mRNA	NONHSAT200462.1	NM_015130	chr4	141592014	141638936	chr4	141541936	141677471
tran	NA	NONHSAT200698.	MTCONS_0009079	chr4	174501046	174528428	chr19	20946830	20993757
cis_mRNA dw20k	NA	NONHSAT200891.1	MTCONS_00141216	chr4	688386	695438	chr4	668129	675822
cis_mRNA up10k	NA	NONHSAT200891.1	NM_006315	chr4	688386	695438	chr4	699530	764768
tran	NA	NONHSAT201710.	MTCONS_0014725	chr4	140099334	140100546	chr4	73920413	73935476
cis_mRNA dw20k	NA	NONHSAT201950.1	NM_017632	chr4	184372671	184378166	chr4	184365744	184370219
cis_mRNA overlap	Lnc-CompleteIn-	NONHSAT202348.1	MTCONS_00151520	chr5	21493545	21494705	chr5	21459589	21504872
cis_mRNA overlap	Lnc-CompleteIn-	NONHSAT202457.1	NM_006713	chr5	32585714	32602059	chr5	32585605	32604185
cis_mRNA dw20k	NA	NONHSAT202541.1	NM_001134848	chr5	42806496	42807099	chr5	42756920	42802539
cis_mRNA overlap	Lnc-CompleteIn-	NONHSAT202613.1	MTCONS_00152237	chr5	56469704	56472134	chr5	56469704	56566600
cis_mRNA overlap	Lnc-Overlap-mRNA	NONHSAT202614.1	NM_001127235	chr5	56469968	56526934	chr5	56471275	56560506
cis_mRNA overlap	Lnc-Overlap-mRNA	NONHSAT202615.1	NM_001127236	chr5	56471816	56542920	chr5	56509901	56560506
cis_mRNA dw20k	NA	NONHSAT202736.1	NM_024754	chr5	71656517	71658805	chr5	71616194	71655180
cis_mRNA overlap	Lnc-Overlap-mRNA	NONHSAT202937.1	NM_001284212	chr5	96038548	96073601	chr5	96038493	96108523
tran	NA	NONHSAT203217.	MTCONS_0018041	chr5	132469745	132469955	chr7	155437203	155480457
cis_mRNA overlap	Lnc-Overlap-mRNA	NONHSAT203225.1	NM_003337	chr5	133710064	133726468	chr5	133706867	133727799
cis_mRNA dw20k	NA	NONHSAT203237.1	NM_021982	chr5	134077045	134087063	chr5	133984475	134063601

tran	NA	NONHSAT203244.	MTCONS_0017304	chr5	134329492	134329803	chr6	150117711	150185480
cis_mRNA overlap	Lnc-CompleteIn-	NONHSAT203283.1	NM_001194956	chr5	138661879	138666127	chr5	138629439	138667366
cis_mRNA overlap	Lnc-CompleteIn-	NONHSAT203283.1	NM_018834	chr5	138661879	138666127	chr5	138629333	138667366
tran	NA	NONHSAT203308.	MTCONS_0009079	chr5	139896169	139896573	chr19	20946830	20993757
cis_mRNA overlap	Lnc-Overlap-mRNA	NONHSAT203340.1	MTCONS_00154774	chr5	141313943	141319716	chr5	141303385	141321612
cis_mRNA overlap	Lnc-Overlap-mRNA	NONHSAT203352.1	MTCONS_00154839	chr5	142292389	142312247	chr5	142150292	142424088
cis_mRNA overlap	Lnc-Overlap-mRNA	NONHSAT203352.1	MTCONS_00154841	chr5	142292389	142312247	chr5	142150292	142484839
cis_mRNA dw20k	NA	NONHSAT203392.1	MTCONS_00154964	chr5	148251988	148255314	chr5	148206156	148244494
cis_mRNA up10k	NA	NONHSAT203498.1	NM_022090	chr5	159827823	159847012	chr5	159820155	159827104
cis_mRNA overlap	Lnc-Overlap-mRNA	NONHSAT203596.1	NM_003945	chr5	172421777	172461862	chr5	172410763	172461900
tran	NA	NONHSAT203676.	NM_032361	chr5	177299276	177319733	chr5	175386534	175395318
cis_mRNA dw20k	NA	NONHSAT203696.1	NM_001257293	chr5	179028891	179037013	chr5	179041179	179050711
cis_mRNA overlap	Lnc-Overlap-mRNA	NONHSAT203696.1	MTCONS_00156015	chr5	179028891	179037013	chr5	178995967	179037027
cis_mRNA _overlap	Lnc-AntiCompleteIn-mRNAIntron	NONHSAT203708.1	MTCONS_00160829	chr5	180229468	180230967	chr5	180217541	180242621
cis_mRNA dw20k	NA	NONHSAT203711.1	NM_001172638	chr5	180257770	180262699	chr5	180274611	180288286
cis_mRNA overlap	Lnc-CompleteIn-	NONHSAT204088.1	MTCONS_00157188	chr5	39109458	39109665	chr5	39105326	39219680
tran	NA	NONHSAT204104.	MTCONS_0009079	chr5	43050280	43054670	chr19	20946830	20993757
cis_mRNA overlap	Lnc-Overlap-mRNA	NONHSAT204186.1	MTCONS_00157512	chr5	55251341	55254032	chr5	55230925	55254032
tran	NA	NONHSAT204365.	MTCONS_0003099	chr5	79778724	79783750	chr11	71927819	71933102
tran	NA	NONHSAT204365.	NM_212550	chr5	79778724	79783750	chr19	45682003	45685059

cis_mRNA dw20k	NA	NONHSAT204415.1	MTCONS_00158352	chr5	86672073	86672997	chr5	86687844	86708850
cis_mRNA overlap	Lnc-CompleteIn-	NONHSAT204416.1	NM_001199189	chr5	86690084	86708693	chr5	86690079	86708721
tran	NA	NONHSAT204447.	MTCONS_0011121	chr5	90634306	90637434	chr2	158592839	158675709
tran	NA	NONHSAT204448.	NM_020702	chr5	90642594	90645975	chr9	34366664	34376894
cis_mRNA overlap	Lnc-Overlap-mRNA	NONHSAT204500.1	MTCONS_00158639	chr5	98215316	98228438	chr5	98203525	98264675
cis_mRNA overlap	Lnc-Overlap-mRNA	NONHSAT204500.1	NM_001270	chr5	98215316	98228438	chr5	98190908	98262238
cis_mRNA overlap	Lnc-Overlap-mRNA	NONHSAT204501.1	MTCONS_00158639	chr5	98232645	98232997	chr5	98203525	98264675
cis_mRNA overlap	Lnc-Overlap-mRNA	NONHSAT204501.1	NM_001270	chr5	98232645	98232997	chr5	98190908	98262238
tran	NA	NONHSAT204507.	MTCONS_0015789	chr5	99397368	99403805	chr5	70264310	70585523
tran	NA	NONHSAT204574.	MTCONS_0009079	chr5	110613216	110638266	chr19	20946830	20993757
tran	NA	NONHSAT204604.	MTCONS_0009079	chr5	117686677	117719092	chr19	20946830	20993757
cis_mRNA overlap	Lnc-Overlap-mRNA	NONHSAT204851.1	MTCONS_00159891	chr5	148884905	148893192	chr5	148873877	148929894
cis_mRNA overlap	Lnc-CompleteIn-	NONHSAT204898.1	MTCONS_00160063	chr5	154287324	154292176	chr5	154266976	154292176
cis_mRNA overlap	Lnc-Overlap-mRNA	NONHSAT204986.1	MTCONS_00160311	chr5	167983371	167988713	chr5	167975500	168006614
cis_mRNA _overlap	Lnc-AntiCompleteIn -mRNAIntron	NONHSAT205081.1	NM_022455	chr5	176672618	176678566	chr5	176560833	176727214
cis_mRNA overlap	Lnc-Overlap-mRNA	NONHSAT205092.1	NM_001190946	chr5	176979894	176981473	chr5	176946790	176981586
cis_mRNA overlap	Lnc-Overlap-mRNA	NONHSAT205100.1	MTCONS_00160714	chr5	178034705	178037290	chr5	178029665	178044667
cis_mRNA overlap	Lnc-CompleteIn-	NONHSAT206649.1	NM_020135	chr6	2771262	2783599	chr6	2765666	2785979
tran	NA	NONHSAT206661.	MTCONS_0020505	chr6	3259238	3303595	chrX	70822199	70833809
cis_mRNA dw20k	NA	NONHSAT206719.1	NM_016588	chr6	5892674	5986354	chr6	5998233	6007633

cis_mRNA up10k	NA	NONHSAT206790.1	NM_145649	chr6	10512750	10512957	chr6	10521568	10629601
cis_mRNA overlap	Lnc-Overlap-mRNA	NONHSAT206983.1	NM_003545	chr6	26204594	26205035	chr6	26204873	26205249
cis_mRNA overlap	Lnc-Overlap-mRNA	NONHSAT206986.1	MTCONS_00163567	chr6	26360288	26370215	chr6	26365387	26378548
cis_mRNA overlap	Lnc-Overlap-mRNA	NONHSAT206986.1	NM_001197249	chr6	26360288	26370215	chr6	26365387	26378548
tran	NA	NONHSAT207026.	MTCONS_0016944	chr6	27861285	27926103	chr6	26113397	26124132
cis_mRNA _overlap	Lnc-AntiOverlap-mRNA	NONHSAT207101.1	NM_203289	chr6	31129963	31135632	chr6	31132114	31134947
cis_mRNA dw20k	NA	NONHSAT207107.1	NM_000594	chr6	31546991	31548157	chr6	31543344	31546112
cis_mRNA overlap	Lnc-Overlap-mRNA	NONHSAT207187.1	NM_022047	chr6	35285587	35289542	chr6	35265595	35289548
cis_mRNA overlap	Lnc-Overlap-mRNA	NONHSAT207199.1	NM_003017	chr6	36564542	36570490	chr6	36562090	36572244
cis_mRNA overlap	Lnc-Overlap-mRNA	NONHSAT207206.1	NM_153370	chr6	36931371	36932611	chr6	36922209	36932613
cis_mRNA dw20k	NA	NONHSAT207296.1	NM_014345	chr6	43297936	43300703	chr6	43303808	43337181
tran	NA	NONHSAT207795.	MTCONS_0009437	chr6	108551412	108584595	chr19	11487649	11495018
cis_mRNA dw20k	NA	NONHSAT207835.1	MTCONS_00166355	chr6	111618329	111619713	chr6	111580482	111613986
cis_mRNA dw20k	NA	NONHSAT207836.1	MTCONS_00166355	chr6	111630406	111634720	chr6	111580482	111613986
cis_mRNA overlap	Lnc-CompleteIn-	NONHSAT208186.1	MTCONS_00167354	chr6	144929604	144929864	chr6	144665239	145174170
tran	NA	NONHSAT208242.	MTCONS_0020982	chr6	149887546	149894440	chrY	2559266	2614373
cis_mRNA overlap	Lnc-Overlap-mRNA	NONHSAT208311.1	NM_003898	chr6	158505148	158508586	chr6	158402888	158520207
cis_mRNA overlap	Lnc-CompleteIn-	NONHSAT208349.1	NM_152857	chr6	160164714	160170446	chr6	160148532	160170453

cis_mRNA overlap	Lnc-Overlap-mRNA	NONHSAT208374.1	NM_005922	chr6	161512504	161515590	chr6	161412759	161538417
cis_mRNA overlap	Lnc-CompleteIn-	NONHSAT208399.1	NM_006775	chr6	163835816	163899919	chr6	163835675	163999628
cis_mRNA overlap	Lnc-CompleteIn-	NONHSAT208401.1	NM_206855	chr6	163984606	163992750	chr6	163835675	163999628
tran	NA	NONHSAT208565.	MTCONS_0003070	chr6	170697560	170700622	chr11	67155110	67165883
cis_mRNA overlap	Lnc-CompleteIn-	NONHSAT208820.1	MTCONS_00169213	chr6	17675506	17707101	chr6	17615266	17707101
cis_mRNA overlap	Lnc-Overlap-mRNA	NONHSAT208826.1	NM_003472	chr6	18249944	18258638	chr6	18224400	18264799
cis_mRNA overlap	Lnc-Overlap-mRNA	NONHSAT208883.1	NM_001286445	chr6	24828250	24829127	chr6	24804509	24936280
cis_mRNA overlap	Lnc-CompleteIn-	NONHSAT208885.1	MTCONS_00169357	chr6	24904924	24907159	chr6	24804509	24936280
cis_mRNA overlap	Lnc-CompleteIn-	NONHSAT208904.1	MTCONS_00169453	chr6	26194498	26196362	chr6	26193184	26199521
cis_mRNA up10k	NA	NONHSAT208904.1	NM_003539	chr6	26194498	26196362	chr6	26188938	26189304
cis_mRNA dw20k	NA	NONHSAT208918.1	NM_001312653	chr6	27068166	27095862	chr6	27114140	27114637
cis_mRNA dw20k	NA	NONHSAT208918.1	NM_021058	chr6	27068166	27095862	chr6	27100095	27100575
cis_mRNA up10k	NA	NONHSAT208918.1	NM_021064	chr6	27068166	27095862	chr6	27100817	27101314
cis_mRNA dw20k	NA	NONHSAT208919.1	NM_080593	chr6	27090436	27091000	chr6	27106072	27114637
cis_mRNA dw20k	NA	NONHSAT208920.1	NM_001312653	chr6	27109873	27113660	chr6	27114140	27114637
cis_mRNA dw20k	NA	NONHSAT208920.1	NM_021064	chr6	27109873	27113660	chr6	27100817	27101314
cis_mRNA up10k	NA	NONHSAT208920.1	NM_021058	chr6	27109873	27113660	chr6	27100095	27100575
tran	NA	NONHSAT208971.1	MTCONS_00175229	chr6	31236531	31238022	chr6_mcf_hap	2617040	2622108

cis_mRNA overlap	Lnc-Overlap-mRNA	NONHSAT208987.1	MTCONS_00169923	chr6	31611915	31612240	chr6	31606805	31620477
cis_mRNA overlap	Lnc-Overlap-mRNA	NONHSAT208987.1	MTCONS_00169924	chr6	31611915	31612240	chr6	31606805	31620477
cis_mRNA dw20k	NA	NONHSAT208989.1	NM_138275	chr6	31695446	31697976	chr6	31691121	31694485
cis_mRNA_overlap	Lnc-AntiCompleteIn-mRNAIntron	NONHSAT208992.1	MTCONS_00164170	chr6	31788494	31789590	chr6	31783291	31798031
cis_mRNA up10k	NA	NONHSAT208992.1	NM_005346	chr6	31788494	31789590	chr6	31795512	31798031
cis_mRNA overlap	Lnc-Overlap-mRNA	NONHSAT209000.1	NM_002124	chr6	32537003	32553978	chr6	32546547	32557613
cis_mRNA up10k	NA	NONHSAT209012.1	NM_152735	chr6	33405140	33422230	chr6	33422356	33425320
cis_mRNA overlap	Lnc-Overlap-mRNA	NONHSAT209028.1	NM_006703	chr6	34253179	34278307	chr6	34254973	34360457
cis_mRNA overlap	Lnc-Overlap-mRNA	NONHSAT209043.1	NM_001145776	chr6	35614325	35656672	chr6	35541362	35656692
cis_mRNA overlap	Lnc-Overlap-mRNA	NONHSAT209055.1	NM_001271641	chr6	36935920	36940529	chr6	36935911	36954327
cis_mRNA dw20k	NA	NONHSAT209313.1	NM_012123	chr6	74229819	74230719	chr6	74171454	74211179
cis_mRNA dw20k	NA	NONHSAT209313.1	NM_133645	chr6	74229819	74230719	chr6	74171454	74211179
cis_mRNA overlap	Lnc-Overlap-mRNA	NONHSAT209325.1	NM_001865	chr6	75947505	75953426	chr6	75947391	75953644
cis_mRNA_overlap	Lnc-AntiCompleteIn-mRNAIntron	NONHSAT209399.1	NM_006416	chr6	88198684	88200326	chr6	88182643	88222057
tran	NA	NONHSAT209407.	MTCONS_0009079	chr6	89235643	89249088	chr19	20946830	20993757
cis_mRNA overlap	Lnc-Overlap-mRNA	NONHSAT209415.1	NM_020466	chr6	90346782	90348159	chr6	90341943	90348474
cis_mRNA overlap	Lnc-Overlap-mRNA	NONHSAT209479.1	MTCONS_00171747	chr6	99859692	99873145	chr6	99846822	99873207

cis_mRNA overlap	Lnc-Overlap-mRNA	NONHSAT209480.1	MTCONS_00171759	chr6	99923214	99930745	chr6	99879250	99963252
cis_mRNA overlap	Lnc-Overlap-mRNA	NONHSAT209672.1	MTCONS_00172356	chr6	130454662	130459611	chr6	130454555	130686570
cis_mRNA overlap	Lnc-CompleteIn-	NONHSAT209874.1	NM_032832	chr6	150139934	150157340	chr6	150139894	150185480
cis_mRNA overlap	Lnc-CompleteIn-	NONHSAT210098.1	NM_182552	chr6	169951339	169952123	chr6	169857303	170102159
tran	NA	NONHSAT210098.	MTCONS_0011279	chr6	169951339	169952123	chr2	208470297	208490028
cis_mRNA dw20k	NA	NONHSAT210353.1	NM_003524	chr6	26254574	26256922	chr6	26251879	26252303
cis_mRNA dw20k	NA	NONHSAT210406.1	NM_005104	chr6	32952020	32954625	chr6	32936437	32949282
tran	NA	NONHSAT210839.	MTCONS_0001507	chr6	95227988	95228771	chr1	183595328	183605076
tran	NA	NONHSAT210839.	NM_005717	chr6	95227988	95228771	chr1	183595328	183605076
tran	NA	NONHSAT210855.	MTCONS_0018041	chr6	98870241	98873393	chr7	155437203	155480457
tran	NA	NONHSAT211099.	MTCONS_0009427	chr6	123996072	124000107	chr19	9920943	9929779
cis_mRNA _overlap	Lnc-AntiCompleteIn-mRNAIntron	NONHSAT211527.1	NM_001164760	chr7	641642	648120	chr7	588834	766978
cis_mRNA overlap	Lnc-CompleteIn-	NONHSAT211530.1	MTCONS_00175524	chr7	912183	914539	chr7	856252	914557
cis_mRNA up10k	NA	NONHSAT211593.1	NM_001037165	chr7	4712307	4717279	chr7	4721930	4811074
cis_mRNA dw20k	NA	NONHSAT211971.1	NM_031267	chr7	40140463	40143507	chr7	39989959	40136733
tran	NA	NONHSAT211972.	MTCONS_0007126	chr7	40577611	40578960	chr16	78133385	78474707
cis_mRNA dw20k	NA	NONHSAT212007.1	MTCONS_00176972	chr7	43670759	43683421	chr7	43622692	43666978
cis_mRNA overlap	Lnc-Overlap-mRNA	NONHSAT212241.1	NM_022170	chr7	73607958	73609859	chr7	73588706	73611429
tran	NA	NONHSAT212304.	NM_007111	chr7	80291320	80294155	chr13	114239056	114295788
tran	NA	NONHSAT212405.	MTCONS_0003070	chr7	97332810	97333299	chr11	67155110	67165883
cis_mRNA dw20k	NA	NONHSAT212429.1	NM_001085367	chr7	99163011	99163934	chr7	99156448	99162328

cis_mRNA_overlap	Lnc-CompleteIn-	NONHSAT212429.1	MTCONS_00178569	chr7	99163011	99163934	chr7	99156045	99174079
cis_mRNA_up10k	NA	NONHSAT212447.1	NM_178238	chr7	99949941	99951559	chr7	99955626	99965454
tran	NA	NONHSAT212447.	NM_024070	chr7	99949941	99951559	chr7	99816871	99819111
cis_mRNA_overlap	Lnc-Overlap-mRNA	NONHSAT212479.1	NM_004279	chr7	102940708	102952838	chr7	102937873	102955133
cis_mRNA_overlap	Lnc-Overlap-mRNA	NONHSAT212507.1	NM_001244262	chr7	106836583	106841366	chr7	106809406	106842974
cis_mRNA_overlap	Lnc-Overlap-mRNA	NONHSAT212509.1	NM_018844	chr7	107220947	107259871	chr7	107220422	107263762
cis_mRNA_overlap	Lnc-Overlap-mRNA	NONHSAT212541.1	NM_001197079	chr7	112090538	112099282	chr7	112092113	112117258
cis_mRNA_overlap	Lnc-Overlap-mRNA	NONHSAT212781.1	MTCONS_00179736	chr7	139090479	139103876	chr7	139052503	139108203
cis_mRNA_overlap	Lnc-AntiCompleteIn-mRNAIntron	NONHSAT212940.1	NM_007349	chr7	154748139	154748817	chr7	154735400	154794682
cis_mRNA_overlap	Lnc-CompleteIn-	NONHSAT213074.1	NM_182491	chr7	1192547	1199817	chr7	1192543	1199855
tran	NA	NONHSAT213366.	MTCONS_0009079	chr7	32473944	32504526	chr19	20946830	20993757
cis_mRNA_dw20k	NA	NONHSAT213394.1	NM_032016	chr7	38279180	38289151	chr7	38217808	38270272
cis_mRNA_overlap	Lnc-CompleteIn-	NONHSAT213396.1	NM_001003799	chr7	38305937	38306406	chr7	38299244	38313248
cis_mRNA_overlap	Lnc-CompleteIn-	NONHSAT213397.1	NM_001003799	chr7	38308874	38310903	chr7	38299244	38313248
cis_mRNA_overlap	Lnc-CompleteIn-	NONHSAT213397.1	NM_001003806	chr7	38308874	38310903	chr7	38299244	38313248
tran	NA	NONHSAT213635.	NM_002737	chr7	76693414	76701962	chr17	64298926	64806862
tran	NA	NONHSAT213635.	NM_003099	chr7	76693414	76701962	chr15	64388083	64436433
tran	NA	NONHSAT213671.	NM_001145204	chr7	81413696	81416913	chr16	12995477	13334273
cis_mRNA_overlap	Lnc-AntiOverlap-mRNA	NONHSAT213687.1	NM_021145	chr7	86792600	86812767	chr7	86781870	86825648

cis_mRNA overlap	Lnc-Overlap-mRNA	NONHSAT213773.1	NM_001185080	chr7	100875377	100877032	chr7	100875373	100882101
cis_mRNA overlap	Lnc-Overlap-mRNA	NONHSAT213781.1	MTCONS_00183454	chr7	102962441	102968201	chr7	102952921	102985320
cis_mRNA dw20k	NA	NONHSAT213798.1	NM_182692	chr7	104743285	104745930	chr7	104756821	105029377
cis_mRNA overlap	Lnc-Overlap-mRNA	NONHSAT213932.1	NM_005000	chr7	123181093	123190640	chr7	123177052	123197958
cis_mRNA dw20k	NA	NONHSAT213950.1	MTCONS_00184038	chr7	126989887	126990347	chr7	127010097	127032778
tran	NA	NONHSAT214073.	MTCONS_0009079	chr7	142786899	142788258	chr19	20946830	20993757
cis_mRNA overlap	Lnc-Overlap-mRNA	NONHSAT214136.1	MTCONS_00184822	chr7	151917610	151945526	chr7	151832010	152133090
cis_mRNA dw20k	NA	NONHSAT214248.1	NM_017802	chr7	836367	845861	chr7	766338	826116
cis_mRNA up10k	NA	NONHSAT214921.1	MTCONS_00183642	chr7	108097214	108099321	chr7	107788071	108096841
cis_mRNA overlap	Lnc-Overlap-mRNA	NONHSAT215538.1	MTCONS_00186715	chr8	21946670	21952390	chr8	21946670	21961891
cis_mRNA dw20k	NA	NONHSAT215646.1	NM_001160008	chr8	32623643	32625477	chr8	32405728	32618507
cis_mRNA dw20k	NA	NONHSAT215813.1	NM_002350	chr8	56926046	56926727	chr8	56792386	56925006
cis_mRNA up10k	NA	NONHSAT215910.1	MTCONS_00191784	chr8	66754987	66755566	chr8	66626569	66754772
tran	NA	NONHSAT215919.	MTCONS_0008052	chr8	67579830	67590528	chr17	8043788	8056490
tran	NA	NONHSAT216086.	MTCONS_0018041	chr8	91727845	91733159	chr7	155437203	155480457
cis_mRNA overlap	Lnc-Overlap-mRNA	NONHSAT216207.1	NM_001695	chr8	104011571	104082629	chr8	104033248	104085285
cis_mRNA _overlap	Lnc-AntiCompleteIn-mRNAIntron	NONHSAT216501.1	NM_001289394	chr8	135679496	135682114	chr8	135490031	135725292
tran	NA	NONHSAT216501.	MTCONS_0009079	chr8	135679496	135682114	chr19	20946830	20993757
cis_mRNA overlap	Lnc-Overlap-mRNA	NONHSAT216629.1	NM_001127213	chr8	144099961	144103825	chr8	144099902	144103827

cis_mRNA dw20k	NA	NONHSAT216645.1	NM_023078	chr8	144683416	144685394	chr8	144686083	144691784
cis_mRNA overlap	Lnc-Overlap-mRNA	NONHSAT216667.1	MTCONS_00190014	chr8	145582285	145583720	chr8	145582217	145588184
cis_mRNA up10k	NA	NONHSAT216667.1	MTCONS_00193819	chr8	145582285	145583720	chr8	145576886	145582183
cis_mRNA dw20k	NA	NONHSAT216669.1	MTCONS_00190021	chr8	145634876	145635360	chr8	145597458	145618489
cis_mRNA overlap	Lnc-CompleteIn-	NONHSAT216691.1	NM_001303100	chr8	567219	595468	chr8	564737	681239
cis_mRNA overlap	Lnc-CompleteIn-	NONHSAT216692.1	NM_001303100	chr8	580408	584400	chr8	564737	681239
tran	NA	NONHSAT216708.	MTCONS_0009079	chr8	1540128	1557990	chr19	20946830	20993757
cis_mRNA overlap	Lnc-Overlap-mRNA	NONHSAT216786.1	NM_001908	chr8	11701829	11703302	chr8	11700034	11725659
cis_mRNA overlap	Lnc-Overlap-mRNA	NONHSAT216816.1	NM_177924	chr8	17924402	17941619	chr8	17913808	17941879
cis_mRNA overlap	Lnc-Overlap-mRNA	NONHSAT216817.1	MTCONS_00190588	chr8	17932523	17941586	chr8	17924402	17942507
cis_mRNA _overlap	Lnc-AntiOverlap-mRNA	NONHSAT216888.1	NM_001440	chr8	28603642	28614824	chr8	28558990	28611207
cis_mRNA up10k	NA	NONHSAT216942.1	NM_025069	chr8	37552203	37553098	chr8	37553269	37557539
cis_mRNA _overlap	Lnc-AntiOverlap-mRNA	NONHSAT216943.1	NM_001003790	chr8	37592279	37594944	chr8	37594197	37604071
cis_mRNA overlap	Lnc-Overlap-mRNA	NONHSAT216945.1	MTCONS_00191132	chr8	38153199	38158074	chr8	38127217	38239790
cis_mRNA overlap	Lnc-CompleteIn-	NONHSAT217026.1	NM_014781	chr8	53586496	53626995	chr8	53535018	53627026
cis_mRNA overlap	Lnc-CompleteIn-	NONHSAT217043.1	NM_001279358	chr8	54959976	54978373	chr8	54958927	55014577
tran	NA	NONHSAT217174.	NM_001145204	chr8	69377026	69379303	chr16	12995477	13334273
tran	NA	NONHSAT217174.	NM_024884	chr8	69377026	69379303	chr14	50709152	50778947

cis_mRNA overlap	Lnc-CompleteIn-	NONHSAT217193.1	NM_001317805	chr8	71486670	71510387	chr8	71485453	71519926
cis_mRNA overlap	Lnc-CompleteIn-	NONHSAT217193.1	NM_014294	chr8	71486670	71510387	chr8	71485453	71520694
cis_mRNA overlap	Lnc-CompleteIn-	NONHSAT217194.1	NM_014294	chr8	71486671	71510236	chr8	71485453	71520694
cis_mRNA overlap	Lnc-CompleteIn-	NONHSAT217244.1	NM_000318	chr8	77895158	77912322	chr8	77892494	77912524
cis_mRNA overlap	Lnc-Overlap-mRNA	NONHSAT217244.1	NM_001172087	chr8	77895158	77912322	chr8	77892494	77913280
cis_mRNA up10k	NA	NONHSAT217342.1	NM_016023	chr8	92072138	92082418	chr8	92082424	92099323
cis_mRNA overlap	Lnc-Overlap-mRNA	NONHSAT217398.1	MTCONS_00192576	chr8	99233789	99248963	chr8	99201661	99306767
cis_mRNA overlap	Lnc-CompleteIn-	NONHSAT217441.1	MTCONS_00192737	chr8	103661013	103663919	chr8	103661005	103668130
cis_mRNA overlap	Lnc-Overlap-mRNA	NONHSAT217557.1	NM_058229	chr8	124543404	124553427	chr8	124510127	124553493
cis_mRNA dw20k	NA	NONHSAT217569.1	NM_194291	chr8	125318430	125320617	chr8	125323159	125384940
cis_mRNA up10k	NA	NONHSAT218744.1	NM_000127	chr8	119124255	119125373	chr8	118811604	119124058
cis_mRNA overlap	Lnc-Overlap-mRNA	NONHSAT219161.1	NM_004972	chr9	5054860	5067785	chr9	4985245	5128183
cis_mRNA overlap	Lnc-Overlap-mRNA	NONHSAT219174.1	MTCONS_00195221	chr9	6709368	6716751	chr9	6702819	7077264
cis_mRNA overlap	Lnc-CompleteIn-	NONHSAT219237.1	MTCONS_00195358	chr9	15864309	15864521	chr9	15723371	15971897
cis_mRNA overlap	Lnc-Overlap-mRNA	NONHSAT219432.1	MTCONS_00195859	chr9	34655188	34672351	chr9	34646586	34661904
cis_mRNA overlap	Lnc-Overlap-mRNA	NONHSAT219432.1	MTCONS_00195863	chr9	34655188	34672351	chr9	34646586	34661904
cis_mRNA overlap	Lnc-Overlap-mRNA	NONHSAT219432.1	MTCONS_00195864	chr9	34655188	34672351	chr9	34646586	34661904
tran	NA	NONHSAT219461.	MTCONS_0008096	chr9	36297039	36305276	chr17	17920192	17942519

cis_mRNA dw20k	NA	NONHSAT219486.1	NM_016042	chr9	37753813	37762481	chr9	37779711	37785089
tran	NA	NONHSAT219619.	MTCONS_0018041	chr9	76617686	76621158	chr7	155437203	155480457
tran	NA	NONHSAT219883.	MTCONS_0003070	chr9	99449359	99536252	chr11	67155110	67165883
tran	NA	NONHSAT219883.	MTCONS_0004045	chr9	99449359	99536252	chr12	56915609	56989980
tran	NA	NONHSAT220022.	MTCONS_0003253	chr9	114688720	114690612	chr11	123986111	124017618
cis_mRNA overlap	Lnc-Overlap-mRNA	NONHSAT220127.1	MTCONS_00197993	chr9	125133324	125141073	chr9	125133284	125157982
cis_mRNA overlap	Lnc-Overlap-mRNA	NONHSAT220127.1	NM_000962	chr9	125133324	125141073	chr9	125133284	125157982
tran	NA	NONHSAT220156.	MTCONS_0011121	chr9	127545744	127548219	chr2	158592839	158675709
tran	NA	NONHSAT220156.	NM_206893	chr9	127545744	127548219	chr11	60552821	60568778
cis_mRNA dw20k	NA	NONHSAT220199.1	NM_001261	chr9	130569573	130570385	chr9	130548305	130553052
cis_mRNA overlap	Lnc-Overlap-mRNA	NONHSAT220204.1	NM_005564	chr9	130914286	130915461	chr9	130911709	130915734
cis_mRNA dw20k	NA	NONHSAT220219.1	NM_005094	chr9	131140317	131153012	chr9	131102839	131123749
tran	NA	NONHSAT220226.	MTCONS_0017832	chr9	131256106	131281157	chr7	92076762	92120925
cis_mRNA overlap	Lnc-CompleteIn-	NONHSAT220236.1	MTCONS_00198375	chr9	131358172	131358912	chr9	131314837	131395944
cis_mRNA overlap	Lnc-CompleteIn-	NONHSAT220236.1	NM_001130438	chr9	131358172	131358912	chr9	131314837	131395944
tran	NA	NONHSAT220236.	MTCONS_0016852	chr9	131358172	131358912	chr6	2877747	2903546
tran	NA	NONHSAT220236.	MTCONS_0016852	chr9	131358172	131358912	chr6	2887500	2903546
cis_mRNA overlap	Lnc-Overlap-mRNA	NONHSAT220395.1	NM_000954	chr9	139874203	139876191	chr9	139871956	139876194
cis_mRNA up10k	NA	NONHSAT220395.1	NM_207510	chr9	139874203	139876191	chr9	139877445	139880210
cis_mRNA dw20k	NA	NONHSAT220408.1	MTCONS_00202781	chr9	140323819	140326867	chr9	140342023	140353786
tran	NA	NONHSAT220623.	NM_003708	chr9	32952699	32959172	chr12	57345215	57353158
cis_mRNA dw20k	NA	NONHSAT220645.1	MTCONS_00199881	chr9	35038622	35044323	chr9	35056065	35072739

cis_mRNA overlap	Lnc-Overlap-mRNA	NONHSAT220651.1	NM_001782	chr9	35609990	35618387	chr9	35609976	35618424
cis_mRNA overlap	Lnc-Overlap-mRNA	NONHSAT220834.1	NM_001102421	chr9	74975036	74980109	chr9	74966341	74980163
cis_mRNA overlap	Lnc-Overlap-mRNA	NONHSAT220834.1	NM_001278243	chr9	74975036	74980109	chr9	74966341	74979508
cis_mRNA overlap	Lnc-Overlap-mRNA	NONHSAT221074.1	MTCONS_00201180	chr9	100671100	100674605	chr9	100666771	100684852
tran	NA	NONHSAT221162.	MTCONS_0003277	chr9	111634209	111640403	chr11	129685741	129733498
cis_mRNA overlap	Lnc-Overlap-mRNA	NONHSAT221180.1	NM_001146108	chr9	114354971	114361918	chr9	114325247	114362135
cis_mRNA dw20k	NA	NONHSAT221353.1	NM_001035254	chr9	130697380	130699615	chr9	130702861	130742812
cis_mRNA overlap	Lnc-Overlap-mRNA	NONHSAT221416.1	NM_020469	chr9	136125788	136130950	chr9	136130563	136150630
cis_mRNA _overlap	Lnc-AntiOverlap-mRNA	NONHSAT221522.1	NM_001317968	chr9	140509784	140513347	chr9	140500092	140509812
cis_mRNA dw20k	NA	NONHSAT221523.1	NM_001317968	chr9	140509819	140513283	chr9	140500092	140509812
cis_mRNA up10k	NA	NONHSAT221523.1	NM_024757	chr9	140509819	140513283	chr9	140513444	140730578
cis_mRNA up10k	NA	NONHSAT222280.1	NM_033387	chr9	134153999	134157308	chr9	134133465	134151906
cis_mRNA overlap	Lnc-Overlap-mRNA	NONHSAT222531.1	NM_005089	chrX	15809055	15823000	chrX	15808574	15841382
cis_mRNA dw20k	NA	NONHSAT222565.1	NM_006406	chrX	23720394	23747906	chrX	23685645	23704514
tran	NA	NONHSAT222603.	MTCONS_0018911	chrX	33654546	33870318	chr8	120879659	121063157
tran	NA	NONHSAT222613.	MTCONS_0018041	chrX	37632920	37643152	chr7	155437203	155480457
cis_mRNA overlap	Lnc-Overlap-mRNA	NONHSAT222650.1	MTCONS_00204406	chrX	40944720	40996182	chrX	40944704	41095832
cis_mRNA overlap	Lnc-CompleteIn-	NONHSAT222700.1	NM_006743	chrX	48433973	48436672	chrX	48432741	48439553
tran	NA	NONHSAT222729.	MTCONS_0015356	chrX	55795311	55795616	chr5	102455958	102541531

cis_mRNA_overlap	Lnc-AntiOverlap-mRNA	NONHSAT223029.1	MTCONS_00208611	chrX	131352447	131564285	chrX	131503343	131623067
cis_mRNA dw20k	NA	NONHSAT223133.1	NM_000117	chrX	153628382	153628742	chrX	153607597	153609883
tran	NA	NONHSAT223133.	MTCONS_0011515	chrX	153628382	153628742	chr2	215711262	215712446
cis_mRNA overlap	Lnc-CompleteIn-	NONHSAT223136.1	NM_023934	chrX	154255336	154283381	chrX	154255064	154285191
cis_mRNA up10k	NA	NONHSAT223159.1	MTCONS_00206690	chrX	3820866	3838787	chrX	3739722	3820041
tran	NA	NONHSAT223188.	MTCONS_0010308	chrX	10891413	11035031	chr2	149066849	149271689
cis_mRNA dw20k	NA	NONHSAT223326.1	NM_002049	chrX	48659460	48659772	chrX	48644982	48652717
tran	NA	NONHSAT223343.	NM_001145204	chrX	52124789	52128633	chr16	12995477	13334273
cis_mRNA dw20k	NA	NONHSAT223537.1	NM_004541	chrX	119028703	119033529	chrX	119005734	119010629
cis_mRNA overlap	Lnc-Overlap-mRNA	NONHSAT223551.1	NM_152692	chrX	119752997	119763943	chrX	119759529	119764005
tran	NA	NONHSAT223617.	MTCONS_0009079	chrX	148416477	148430371	chr19	20946830	20993757
cis_mRNA overlap	Lnc-CompleteIn-	NONHSAT223651.1	NM_001569	chrX	153275968	153278620	chrX	153275957	153285342
cis_mRNA overlap	Lnc-Overlap-mRNA	NONHSAT223653.1	MTCONS_00208996	chrX	153352133	153372705	chrX	153287264	153354096
tran	NA	NONHSAT224107.	MTCONS_0011100	chrX	106612945	106614557	chr2	148687966	148778463
cis_mRNA dw20k	NA	NONHSAT224150.1	NM_144658	chrX	117828658	117829559	chrX	117629872	117820123
cis_mRNA dw20k	NA	NONHSAT224322.1	NM_001171132	chrX	153719800	153720429	chrX	153734490	153744566
cis_mRNA overlap	Lnc-CompleteIn-	NONHSAT224362.1	MTCONS_00209956	chrY	14774338	14821338	chrY	14774265	14972768
cis_mRNA_overlap	Lnc-AntiOverlap-mRNA	NONHSAT224377.1	NM_003411	chrY	2834886	2870668	chrY	2803518	2850547
cis_mRNA overlap	Lnc-CompleteIn-	NONHSAT224405.1	MTCONS_00210296	chrY	15346458	15409629	chrY	15343342	15592550

cis_mRNA overlap	Lnc-Overlap-mRNA	NONHSAT224405.1	MTCONS_00210299	chrY	15346458	15409629	chrY	15356737	15592550
cis_mRNA overlap	Lnc-Overlap-mRNA	NONHSAT224405.1	NM_001258249	chrY	15346458	15409629	chrY	15360259	15592550
cis_mRNA overlap	Lnc-Overlap-mRNA	NONHSAT224405.1	NM_001258258	chrY	15346458	15409629	chrY	15360259	15592550
cis_mRNA overlap	Lnc-Overlap-mRNA	NONHSAT224405.1	NM_001258261	chrY	15346458	15409629	chrY	15360259	15592550
cis_mRNA overlap	Lnc-Overlap-mRNA	NONHSAT224405.1	NM_001258264	chrY	15346458	15409629	chrY	15360259	15592550
cis_mRNA overlap	Lnc-Overlap-mRNA	NONHSAT224405.1	NM_182659	chrY	15346458	15409629	chrY	15409389	15592550
cis_mRNA up10k	NA	NONHSAT224424.1	MTCONS_00210024	chrY	21716365	21729078	chrY	21729199	21756123

Pearson correlation	Spearman correlation
0.7221	0.6784
0.6482	0.6567
0.611	0.7351
0.7059	0.6629
0.7757	0.7042
0.761	0.6709
0.9545	0.771
0.8506	0.8167
0.9683	0.8554
0.9324	0.7052
0.8558	0.835
0.9563	0.8278
0.6213	0.6148
0.7456	0.6478
0.8639	0.8002
0.6578	0.7086

0.6658	0.7266
0.7274	0.8017
0.7033	0.7423
0.8718	0.8645
0.7731	0.6713
0.6199	0.6542
0.6037	0.7244
0.8771	0.845
0.6291	0.6209
0.6268	0.6715
0.7426	0.749
0.6324	0.6186
0.6675	0.6601
0.766	0.6374
0.6278	0.6256

0.7744	0.7112
0.6821	0.6439
0.6676	0.6376
0.7693	0.7871
0.8862	0.7651
0.6014	0.6515
0.6629	0.6376
0.6624	0.6296
0.7311	0.6715
0.6601	0.6206
0.7846	0.7502
0.6797	0.7158
0.7009	0.7986
-0.6562	-0.6323
0.777	0.791

-0.8136	-0.8183
0.6923	0.6758
0.6509	0.6547
0.6356	0.6315
0.9459	0.9396
0.7819	0.6836
0.6239	0.8045
0.7359	0.8006
0.9314	0.6348
0.7171	0.6896
0.8919	0.8496
0.8013	0.7836
0.7473	0.6516
0.8088	0.793
0.7076	0.7567
0.836	0.8608
0.6285	0.6458

0.8632	0.8458
0.6181	0.6585
0.7191	0.634
0.6304	0.6809
0.6708	0.6816
0.8265	0.807
0.6688	0.7191
0.8413	0.6998
0.6225	0.7233
0.72	0.7959
0.7082	0.7168
0.7291	0.6113
0.63	0.6139
0.6023	0.6106
0.6684	0.6468
0.719	0.6355
0.7998	0.8083

0.6264	0.732
0.8019	0.8512
0.722	0.7605
0.7392	0.7127
0.6295	0.6177
0.7447	0.6794
0.9465	0.9157
0.9089	0.7402
0.647	0.6848
0.7344	0.7518
0.6386	0.6371
-0.7308	-0.7656
0.8488	0.8911
0.6132	0.6858
0.628	0.6048
0.7	0.671

0.6825	0.727
0.6273	0.7321
0.7196	0.7962
0.6341	0.66
0.7121	0.713
0.6463	0.6739
0.6396	0.711
0.6789	0.6913
0.6774	0.6655
0.6673	0.7166
0.6457	0.6652
0.8975	0.8485
0.6844	0.6424
0.7246	0.7119
0.9063	0.8382
0.9024	0.8151
0.7178	0.684
0.9382	0.7834
0.7151	0.6196
0.696	0.6908

0.7795	0.7302
0.9108	0.9478
0.7863	0.7557
0.6483	0.6101
0.6437	0.6135
0.693	0.7178
0.653	0.6882
-0.7112	-0.6332
0.6836	0.658
0.664	0.677
0.7528	0.7697
0.8085	0.6124
0.762	0.6219
0.7729	0.699
0.7607	0.7016
0.7921	0.8288

-0.6492	-0.6358
0.6219	0.6336
0.9181	0.8541
0.9646	0.9458
0.767	0.641
0.7515	0.7226
0.7988	0.8693
0.651	0.8557
0.7597	0.7887
0.6907	0.6128
0.8502	0.8604
0.662	0.641
0.6229	0.655
0.8541	0.8727
0.6739	0.7909
0.7589	0.7827

0.7898	0.8104
-0.7179	-0.7522
0.6272	0.6352
0.7095	0.7024
0.7	0.6297
0.6432	0.6843
0.6641	0.6808
-0.6076	-0.6241
0.6218	0.6962
0.612	0.6288
0.6871	0.6559
0.714	0.7009
0.6067	0.6379
0.636	0.6675
0.8668	0.8974
0.7476	0.6948

0.6417	0.6697
0.8033	0.7778
0.7693	0.6394
0.7738	0.7681
0.6709	0.6463
0.6177	0.6061
0.785	0.8954
0.6931	0.7168
0.8067	0.8257
0.9021	0.7244
0.7406	0.6874
0.8847	0.739
0.7216	0.7389
0.7656	0.7253
0.7123	0.6417

0.7746	0.7497
0.7279	0.791
0.8883	0.6086
0.6126	0.6575
0.6225	0.7061
0.7755	0.8122
0.7376	0.7748
0.7956	0.8504
0.6916	0.6548
0.7319	0.7206
0.85	0.8183
0.8268	0.8201
0.6471	0.6907
0.789	0.7384
0.7308	0.6691

0.7113	0.6352
0.7175	0.6817
0.8794	0.7894
0.6273	0.6088
-0.6684	-0.6842
0.6627	0.6178
0.6647	0.6452
0.6918	0.6917
0.7016	0.6249
0.698	0.686
0.8469	0.8632
-0.6132	-0.6088
0.6571	0.6255
0.7597	0.7339

0.7778	0.8034
0.7238	0.7492
0.6661	0.6415
-0.6237	-0.6027
0.6073	0.6142
0.9929	0.8176
0.994	0.8902
0.9942	0.8393
0.9949	0.8718
0.9743	0.7005
0.9853	0.8585
0.9971	0.8303
0.9775	0.7501
0.6929	0.6236
0.6339	0.6172
0.6208	0.6752
0.9621	0.6586

0.9876	0.6595
0.9663	0.7894
0.9563	0.8232
0.6235	0.6925
0.8226	0.673
0.8753	0.7147
0.8203	0.7681
0.7064	0.7215
0.7533	0.7376
0.738	0.6948
0.7318	0.6546
0.6826	0.6172
0.7825	0.7481
0.7665	0.7843
0.631	0.6336
0.6498	0.624
0.6804	0.6307

0.7686	0.8296
0.6974	0.7151
0.7784	0.7312
0.6393	0.679
0.6615	0.6416
0.7294	0.6603
0.8289	0.69
0.708	0.6585
-0.6354	-0.6637
0.722	0.7687
0.7153	0.6444
0.6777	0.613
0.6413	0.6983
0.836	0.7476
0.6251	0.6157
0.8837	0.7916
0.7019	0.6495
0.8127	0.7904
0.6077	0.6638

0.8888	0.7078
0.7491	0.807
0.7275	0.7641
0.6954	0.748
0.7349	0.6549
0.6569	0.7423
0.8828	0.8713
-0.6144	-0.6137
0.8335	0.8308
0.7829	0.7446
0.7392	0.6815
0.8182	0.8015
0.7371	0.6217
0.7222	0.6757
0.6508	0.7069
0.8263	0.8275
0.6129	0.6129

0.6782	0.6744
0.8517	0.8565
0.9103	0.9446
0.7791	0.754
0.6388	0.6823
0.7924	0.7211
0.7118	0.6713
0.7785	0.802
0.8677	0.7129
0.7485	0.6641
0.6016	0.682
0.7612	0.6049
0.6631	0.7221
0.7264	0.6614
0.6126	0.6801
0.7938	0.7029

0.7391	0.6552
0.6255	0.6668
0.7314	0.7286
0.7884	0.6557
0.7336	0.6687
0.6137	0.7421
0.6609	0.7346
0.7507	0.6826
0.6627	0.8124
0.6016	0.7398
0.69	0.6726
0.7257	0.6011
0.6351	0.6716
0.6458	0.6993
0.6159	0.7049
0.6547	0.6048
0.6807	0.7861
0.6564	0.7541
0.7035	0.8254
0.6883	0.8078
0.706	0.7432
0.6614	0.6786
0.7509	0.7901
0.6133	0.7191
0.7368	0.8353
0.6958	0.7838
0.7428	0.6909
0.668	0.7058
0.6125	0.7434

0.636	0.6622
-0.6497	-0.7199
0.6282	0.7969
0.6006	0.6557
0.6638	0.6478
0.689	0.731
0.6495	0.6455
0.7638	0.8483
0.6402	0.6977
0.6287	0.735
0.6706	0.6264
0.7881	0.8425
-0.6372	-0.6742
0.605	0.649
0.6983	0.6975
0.7222	0.6847
0.641	0.7047
0.6487	0.708
0.6308	0.687
0.848	0.8539
0.6741	0.6597
0.7051	0.696
0.7243	0.7261

0.6557	0.6758
0.6456	0.6704
0.7733	0.7823
0.8783	0.8339
0.8118	0.8261
0.8095	0.827
0.675	0.6461
0.9054	0.9383
0.9876	0.9558
0.8514	0.7112
0.7606	0.7453
0.7642	0.8084
0.7866	0.718
0.8688	0.8589
0.8412	0.7983
0.8151	0.7946
0.8951	0.9408

0.7516	0.8681
0.6528	0.6843
0.891	0.7484
0.8245	0.8181
0.663	0.7028
0.835	0.8765
0.7082	0.6996
0.8924	0.8538
0.7418	0.7305
0.8633	0.8494
0.7435	0.8115
0.8663	0.7901
0.8349	0.8194
0.7454	0.8034
0.7059	0.8559
0.9086	0.7372
0.913	0.9188

0.6462	0.6436
0.8199	0.8236
0.7042	0.6848
0.7013	0.633
0.6176	0.7366
0.6233	0.6155
0.6201	0.6047
0.6822	0.7558
-0.6403	-0.6175
0.6958	0.7223
0.6222	0.6216
0.6712	0.6933
0.756	0.7327
0.7253	0.626
0.8532	0.6424
0.6996	0.6786
0.7717	0.837
-0.6527	-0.8267

0.6832	0.6887
0.8429	0.8581
0.7348	0.6274
0.6138	0.6393
0.616	0.6499
0.8048	0.709
0.9884	0.9304
0.8198	0.7944
0.7505	0.6383
0.8994	0.867
0.9776	0.7783
0.7027	0.6129
0.6072	0.6246
0.6816	0.7613
0.6528	0.7452
0.8569	0.8414
0.7707	0.8031
0.6566	0.71
0.6448	0.6585
0.7117	0.6447

0.714	0.7377
0.7104	0.6636
0.7214	0.7087
-0.6208	-0.6789
0.7615	0.7405
0.7128	0.7442
0.6325	0.6145
0.7063	0.751
0.6937	0.668
0.6356	0.6729
0.7056	0.7386
0.6641	0.7421
0.717	0.6984
0.6197	0.6048
0.6684	0.6151
0.7811	0.6961
0.9604	0.6919

0.6156	0.6327
0.7517	0.8335
0.8555	0.7786
0.8185	0.7122
-0.7263	-0.7151
0.6241	0.6136
0.7867	0.6374
0.6217	0.73
0.6578	0.6913
0.8857	0.8417
0.7269	0.707
0.7199	0.8119
0.6087	0.6342
0.6277	0.7106
0.6872	0.6288
0.9066	0.6761
0.9067	0.8538
0.7039	0.6997

0.7592	0.6672
0.7693	0.6934
0.6216	0.646
0.8202	0.7571
-0.6328	-0.6357
0.6073	0.6143
-0.8106	-0.8424
-0.631	-0.708
0.8849	0.8023
0.6402	0.6254
-0.6504	-0.631
0.7695	0.7345
0.8362	0.7224
-0.8052	-0.7715
0.7124	0.6209
0.7163	0.6601
0.6602	0.6498

0.8863	0.8124
0.86	0.7356
0.7735	0.6137
0.8734	0.8453
0.7585	0.7063
0.7225	0.645
0.6559	0.6088
0.6101	0.6293
0.6762	0.7443
0.8289	0.7054
0.8822	0.7362
0.6306	0.6868
0.6787	0.6846
-0.6528	-0.6411
-0.7391	-0.7222
0.6363	0.6744
0.6643	0.6111
0.6314	0.659
-0.6013	-0.6265

0.6261	0.6662
-0.631	-0.6074
0.6869	0.7261
0.8731	0.7087
0.6281	0.6802
0.6397	0.6653
0.8963	0.7769
0.9029	0.6748
0.9415	0.8693
0.714	0.617
0.866	0.7556
0.9221	0.82
0.8935	0.7566
0.7854	0.8017
0.7027	0.6952

0.7901	0.7595
0.7388	0.6973
0.6237	0.6794
0.61	0.6094
-0.6046	-0.6925
0.7846	0.6432
0.6344	0.7299
0.6557	0.6351
0.7221	0.7033
0.6043	0.6393
0.6334	0.6346
0.6399	0.6928
0.7509	0.7176
0.6351	0.7123
0.6478	0.6413
0.6547	0.6394

0.6623	0.6345
0.7495	0.7267
0.7907	0.6665
0.8864	0.8538
0.8954	0.7919
0.7632	0.6948
0.6489	0.6725
0.8593	0.6774
0.7125	0.6852
-0.8978	-0.8792
0.9676	0.7477
0.9129	0.7708
0.6786	0.6583
0.6275	0.663
0.7391	0.7267
0.8077	0.7799
0.7124	0.7129
0.7886	0.7666
0.6571	0.6801
0.6408	0.662

0.6012	0.6179
0.7448	0.6294
0.7075	0.8384
0.9939	0.7557
0.9666	0.7826
0.9884	0.8067
0.6467	0.6511
0.7466	0.7001
0.8724	0.831
0.8799	0.8825
0.8485	0.9008
0.6926	0.7338
0.6184	0.6332
0.69	0.7312
0.8739	0.7296

0.8865	0.6001
0.664	0.6564
0.8643	0.7894
0.8501	0.6057
0.6765	0.6924
0.769	0.678
0.8192	0.8531
0.8495	0.8224
-0.6693	-0.6939
0.7043	0.7155
0.7145	0.6476
-0.7295	-0.661
0.6678	0.653
-0.6835	-0.718
0.6551	0.7685
0.6548	0.6912

0.6698	0.6931
0.7112	0.6631
0.824	0.8167
0.9429	0.9496
-0.8024	-0.831
-0.6458	-0.6511
0.8123	0.8243
0.8125	0.8051
0.6758	0.6617
0.7498	0.7946
0.6847	0.6016
0.6396	0.6279
0.7681	0.7636
0.6551	0.6285
0.6311	0.6683
0.6294	0.618
-0.6497	-0.7212

-0.6235	-0.6419
0.6222	0.6991
0.722	0.6082
0.7035	0.7176
0.7982	0.8356
0.859	0.8299
0.7788	0.6515
0.6521	0.7341
0.7159	0.7342
0.6631	0.6778
0.7428	0.753
0.6388	0.6209
0.7027	0.6449
0.6337	0.6577
0.7788	0.7009
0.6225	0.6347

0.6132	0.61
0.7829	0.7596
0.6014	0.6382
0.7871	0.6027
0.6586	0.6728
0.7367	0.6031
0.6169	0.603
0.6239	0.6127
0.6251	0.6556
0.8226	0.7931
0.7002	0.6217
0.6056	0.6089
0.6312	0.6008
0.8554	0.8576
0.7952	0.8438

-0.6835	-0.6355
0.7833	0.777
0.7025	0.7035
0.6898	0.698
0.8343	0.7725
0.698	0.7261
0.7369	0.6975
0.7102	0.6765
0.7141	0.7623
0.7263	0.6614
0.6572	0.6064
0.6007	0.6301
0.7935	0.8185
0.8638	0.8217
0.6557	0.6643

0.7802	0.7522
0.7541	0.6592
0.8234	0.8215
0.7587	0.6587
-0.7105	-0.7369
0.7225	0.7452
0.7245	0.72
-0.733	-0.844
0.6158	0.6804
0.8105	0.7692
0.7271	0.634
-0.6843	-0.693
0.7623	0.6783
0.9229	0.901
0.6221	0.6498

0.8364	0.8225
0.7777	0.709
0.6922	0.6472
0.6647	0.7084
0.7324	0.8286
0.7543	0.6985
-0.7239	-0.642
0.9907	0.7914
-0.7018	-0.6014
0.6396	0.6605
0.639	0.6304
0.6954	0.7569
0.6979	0.7186
-0.6819	-0.7005
0.6259	0.6467
0.6462	0.679

0.6178	0.6318
0.6834	0.6793
0.7749	0.6095
0.7712	0.6719
0.6059	0.7117
0.6711	0.6273
0.8794	0.8775
0.7398	0.7529
0.713	0.7323
0.675	0.7239
0.6609	0.6862
0.7177	0.6931
-0.7746	-0.8363
-0.7002	-0.6638
0.6812	0.7097
0.7364	0.7005
0.8711	0.8256

0.6214	0.6272
0.7527	0.6645
0.6556	0.7037
0.7771	0.7392
0.6524	0.64
0.7296	0.7012
0.7183	0.7742
0.608	0.6246
0.7039	0.6774
0.6516	0.7505
0.6185	0.613
0.8714	0.8304
0.8765	0.8232
0.6149	0.6528
0.7872	0.7582
0.8529	0.827

0.6325	0.7089
0.7519	0.7173
0.615	0.6233
0.6352	0.6365
0.7984	0.8113
0.7741	0.7306
-0.6299	-0.7108
0.7822	0.773
0.7504	0.8076
0.601	0.6209
0.6976	0.6245
0.7692	0.7498
0.785	0.8122
0.8545	0.8645
0.8275	0.8774
0.8086	0.7894

0.6658	0.6543
0.7624	0.7096
0.7482	0.7623
0.6932	0.7058
0.6963	0.668
0.6261	0.6156
0.8486	0.8636
0.6246	0.7055
-0.8689	-0.8666
0.8808	0.8632
0.7423	0.885
0.6887	0.8454
0.89	0.7689
0.6443	0.6019
-0.6848	-0.6167

0.743	0.7455
0.617	0.6254
-0.6663	-0.6823
0.6904	0.7666
0.6585	0.7059
0.8675	0.8504
0.7135	0.7783
0.7723	0.767
0.6642	0.6173
0.9389	0.7583
0.6695	0.7461
0.6422	0.6423
0.7192	0.6683
0.7019	0.6273
0.6457	0.6708
0.7464	0.7068

0.6149	0.667
0.8555	0.8417
0.6577	0.7522
0.7838	0.6963
0.611	0.6503
0.7082	0.6606
0.624	0.6284
0.6793	0.6217
0.6162	0.6674
-0.6623	-0.6436
0.8367	0.6332
0.8253	0.918
0.7749	0.8141
0.8982	0.8808
0.6398	0.6659
0.6755	0.6938

0.7802	0.6814
0.7802	0.6814
0.7802	0.6814
0.6201	0.6001
0.7039	0.7012
0.668	0.6356
0.6185	0.6671
0.6502	0.7336
0.9216	0.7526
0.8673	0.7083
0.8966	0.78
0.801	0.8156
0.8299	0.6148
0.8289	0.6235
0.8196	0.6304
0.8227	0.794
0.8929	0.913

0.6124	0.6019
0.9255	0.6916
0.7734	0.6658
0.6252	0.633
0.6273	0.7174
0.8223	0.7418
0.6119	0.6115
0.6831	0.6016
0.893	0.8597
0.692	0.641
0.6879	0.618
0.6604	0.6264
0.695	0.6944
0.7766	0.6799
0.8652	0.8514
0.7132	0.6321
0.7247	0.7563
0.6395	0.7457
0.72	0.7281
0.8019	0.803

0.6627	0.7029
0.6792	0.6687
0.9254	0.6066
0.7838	0.763
0.6894	0.6637
0.8619	0.6097
0.8896	0.7167
0.8599	0.6453
0.6675	0.66
0.6299	0.6537
0.9087	0.611
0.8991	0.7361
0.9068	0.8192
0.7671	0.6816
0.705	0.6249

0.6498	0.7354
0.6507	0.7612
0.6077	0.6771
0.7351	0.7764
0.6059	0.6368
0.7609	0.6772
0.6147	0.6675
0.9031	0.7902
0.905	0.8037
0.8287	0.663
0.7754	0.8171
0.9337	0.7514
0.8687	0.7266
0.6186	0.7079
0.8009	0.8104
0.6742	0.6278
0.856	0.6379
0.6517	0.6707
0.7195	0.8002

0.7211	0.7747
0.7118	0.8709
0.6388	0.618
0.8225	0.8433
-0.6258	-0.6993
0.7573	0.7488
0.8476	0.8545
-0.604	-0.7103
0.7818	0.8275
0.8076	0.6047
0.8188	0.7409
0.7912	0.7139
0.721	0.6896
0.7591	0.7058
0.8353	0.6595
0.7681	0.7739
0.7248	0.7165

0.6482	0.7107
0.7359	0.8576
0.6868	0.7087
0.7407	0.6893
0.7222	0.6345
-0.8056	-0.8251
0.7032	0.7169
0.7986	0.6469
0.8567	0.7312
0.6662	0.6555
0.6655	0.641
0.6033	0.7404
0.6991	0.6194
0.6781	0.6715
0.8513	0.7741
0.6025	0.6358

0.6603	0.7067
0.7524	0.7668
0.7416	0.7267
0.6464	0.6306
0.8441	0.7096
0.8319	0.7635
0.9115	0.8772
0.6899	0.6826
0.8055	0.752
0.6478	0.6664
0.6817	0.6452
0.7557	0.747
0.7279	0.6364
0.6285	0.6815
0.7095	0.751
0.7958	0.7687
0.7317	0.6957
0.6136	0.6641
0.8552	0.8267
0.6224	0.6574

0.6368	0.6992
0.6699	0.7417
-0.723	-0.7738
0.926	0.6473
-0.6381	-0.6626
0.7825	0.803
0.7851	0.8128
0.7032	0.8565
0.6878	0.6427
0.7556	0.8097
0.7318	0.672
0.7788	0.8115
0.6253	0.6948
0.6416	0.6346
0.8359	0.8519
0.7507	0.8056
0.6164	0.6612

0.7005	0.746
0.7257	0.7454
0.6897	0.7328
0.7704	0.8428
0.61	0.6748
0.6259	0.6641
0.7672	0.7473
0.7147	0.7708
0.8649	0.7805
0.8648	0.6976
0.8994	0.9974
0.6588	0.6606
0.7794	0.6476
0.7731	0.7927
0.7039	0.664
0.6362	0.7085

0.6823	0.601
0.8645	0.8676
0.7463	0.75
0.6173	0.6349
0.8661	0.8704
0.638	0.6203
0.6495	0.7094
0.6489	0.7699
0.6285	0.6329
0.741	0.6096
0.8471	0.7999
0.6466	0.6096
0.7495	0.828
0.8333	0.8338
0.894	0.6962
0.8232	0.6845
0.6776	0.6055
0.6451	0.6629

0.8042	0.7489
0.7466	0.6048
0.6717	0.6508
0.67	0.7956
0.8553	0.8467
0.6206	0.6133
0.7309	0.7138
0.6995	0.7163
0.6572	0.6674
0.9149	0.9637
0.6451	0.6255
0.7599	0.7841
0.8543	0.8308
0.7846	0.7858
0.6126	0.6726
0.6286	0.6408
0.6715	0.6643

0.6557	0.7049
0.6636	0.7462
0.6535	0.6923
-0.6145	-0.6467
0.8398	0.8252
0.7288	0.6154
0.8828	0.6101
0.759	0.7537
0.8649	0.8725
0.9058	0.9361
0.9391	0.9539
0.75	0.6941
0.7224	0.705
0.6007	0.623
0.6908	0.6609
0.7982	0.8695
0.6373	0.7772
0.9376	0.8202

0.7546	0.8222
0.7174	0.6606
-0.6166	-0.601
-0.8129	-0.7843
0.8328	0.7884
0.7577	0.7404
0.6266	0.6736
0.7299	0.8347
0.7055	0.6474
0.7454	0.7658
0.7646	0.8652
0.8761	0.8969
0.7696	0.843
0.9014	0.8925
0.809	0.8271
0.931	0.8827
0.7332	0.7557
0.6907	0.6441
0.6657	0.6477
0.6498	0.7164

0.8002	0.6743
0.7354	0.6696
0.9192	0.7955
0.6796	0.6915
-0.6809	-0.6573
0.7271	0.6821
0.7074	0.7947
0.8815	0.8564
0.7907	0.767
0.8435	0.7678
0.674	0.6199
0.6117	0.6924
0.8686	0.8575
0.8686	0.8575
0.8901	0.6469
0.8076	0.8087
0.6442	0.6246
0.687	0.7575

0.6711	0.6439
0.6152	0.6652
0.6202	0.6887
0.948	0.7443
0.619	0.6864
0.6805	0.667
0.7347	0.7519
0.6622	0.6071
0.7386	0.7614
-0.6267	-0.6316
0.7176	0.7162
0.6345	0.6968
0.8404	0.7702
0.6478	0.7448
0.7328	0.6111
0.631	0.6052
0.6952	0.6887

0.6329	0.6965
0.7161	0.7243
0.7627	0.7802
0.7625	0.6462
0.7623	0.602
-0.7956	-0.8365
0.909	0.7708
0.7381	0.686
0.7381	0.686
0.9154	0.6026
0.9154	0.6026
0.9546	0.6496
0.9131	0.607
0.9191	0.773
0.7748	0.6235
0.9081	0.7522
0.915	0.6426
0.7872	0.6217
0.8391	0.6445
0.7285	0.6896
0.645	0.6591
0.6946	0.678

0.6178	0.6402
0.6029	0.6872
0.728	0.771
0.7444	0.7126
0.8176	0.8292
0.6217	0.631
0.7478	0.702
0.7676	0.7782
0.6762	0.6758
0.6917	0.6614
0.6925	0.7087
0.6492	0.6645
0.6652	0.7045
0.6065	0.6338
0.782	0.8502
0.6748	0.6733
-0.6515	-0.7429
0.6646	0.6532

0.7655	0.7541
0.8364	0.7235
0.6375	0.619
0.6635	0.6285
0.7383	0.8301
0.6254	0.6207
0.7496	0.6757
0.824	0.8402
0.6684	0.7484
0.6659	0.6226
0.6552	0.6309
0.7197	0.7502
0.7933	0.7112
0.6679	0.7331
0.6178	0.6211
0.6647	0.6876
0.8961	0.821
0.6597	0.6378

0.6583	0.6443
0.6439	0.7084
0.697	0.822
0.6422	0.6388
0.8241	0.6878
0.8812	0.825
0.8441	0.88
0.6127	0.6505
0.6673	0.7038
0.6924	0.6201
0.6691	0.7026
0.7184	0.7375
-0.6368	-0.7026
0.6676	0.7249
0.6958	0.6295
0.7274	0.7039
-0.7161	-0.6938

0.7017	0.7842
0.7276	0.6882
-0.602	-0.6119
0.6027	0.6525
0.6178	0.6714
0.6998	0.7139
0.645	0.6265
0.7797	0.7193
0.6317	0.6139
0.7073	0.6887
0.8065	0.8378
0.6004	0.6019
0.9111	0.7708
0.7777	0.698
0.8811	0.8926
0.6334	0.6555
0.8812	0.8382
0.7247	0.6829
0.6221	0.6039

0.7138	0.631
0.7836	0.8087
0.6543	0.6765
0.6363	0.6588
0.7098	0.8571
0.6861	0.7757
0.7563	0.7549
0.8852	0.8522
0.6383	0.6287
0.7081	0.812
0.6453	0.6923
0.7095	0.7373
0.663	0.6599
0.8198	0.6163
0.6079	0.6031
0.7018	0.6175

-0.6337	-0.6364
0.63	0.724
0.839	0.6423
0.7438	0.6949
0.9427	0.6213
0.7188	0.6287
0.7188	0.6287
0.6486	0.6285
0.7999	0.7402
0.6184	0.6278
0.7936	0.8565
0.793	0.7209
0.6523	0.6118
0.6127	0.7025
0.7793	0.6585
0.6414	0.6556
0.6845	0.7723
0.7346	0.7573

0.7058	0.701
0.6994	0.7148
0.6065	0.6929
0.7097	0.7238
0.786	0.7798
0.7051	0.6732
0.6232	0.6248
0.746	0.7806
0.622	0.6065
-0.6247	-0.6844
0.6959	0.7847
0.7714	0.6475
0.7012	0.6235
0.6989	0.6654
0.6941	0.8433
0.8547	0.7815
0.7663	0.7414

0.6203	0.6325
0.7726	0.7357
0.7951	0.7584
0.752	0.7043
0.7314	0.6325
0.6593	0.6481
0.6198	0.6949
0.777	0.7214
0.7234	0.6163
0.6242	0.708
0.7492	0.734
0.6364	0.7069
-0.6118	-0.7628
0.7751	0.7956
0.7751	0.7956
0.7042	0.7754
0.6098	0.6041

0.6423	0.6174
0.9751	0.7708
0.9508	0.8167
0.7446	0.8067
0.7069	0.7917
-0.6529	-0.6678
0.6082	0.6192
0.6219	0.6177
0.682	0.7338
0.7445	0.6276
0.6204	0.6054
0.7176	0.7291
0.6613	0.7096
0.643	0.6493
0.8275	0.8056
0.8636	0.8293
0.7598	0.7155
0.6707	0.6074
0.6835	0.6921
0.7503	0.6534

0.7269	0.7587
0.7019	0.6886
0.6058	0.6696
0.7697	0.7216
0.7697	0.7216
0.6841	0.6896
0.9199	0.8595
0.712	0.7458
0.712	0.7458
0.6742	0.6587
0.6724	0.6027
0.7618	0.6375
0.7177	0.6336
0.6106	0.6845
0.6585	0.6916
0.718	0.7031

0.6142	0.6633
0.6676	0.611
0.7458	0.633
0.8347	0.7465
0.7822	0.7657
0.6634	0.6505
0.672	0.7215
0.6688	0.697
0.7294	0.6511
-0.6402	-0.6143
0.8309	0.876
0.7412	0.6974
0.66	0.6411
0.7319	0.7157
0.6407	0.6024
0.9185	0.8744
0.6717	0.6506

0.7713	0.7426
0.6228	0.6593
0.7026	0.6977
0.6529	0.6583
0.7429	0.7835
0.7163	0.6148
0.6987	0.6588
0.683	0.6541
0.8294	0.7847
0.7279	0.6255
0.7618	0.7799
0.66	0.6626
0.6623	0.6616
0.6596	0.7411
0.8095	0.7547
0.7871	0.7932
0.7624	0.7355
0.685	0.7644

0.6955	0.7982
0.7078	0.7316
-0.6283	-0.6663
0.7759	0.6435
0.7052	0.6609
0.6322	0.6146
0.7911	0.7809
0.7332	0.6301
0.752	0.6366
0.6109	0.6303
0.8145	0.8388
-0.6073	-0.6541
-0.6798	-0.6784
0.7851	0.6908
0.8991	0.8521
0.7095	0.749
0.6934	0.749
0.6636	0.6451

0.6777	0.6559
0.8179	0.7403
0.9316	0.82
-0.7462	-0.647
0.6826	0.6797
0.6269	0.6685
0.6094	0.7701
0.6749	0.7312
0.7546	0.6822
0.6938	0.6223
0.7572	0.7396
0.6535	0.6638
0.6424	0.7474
0.863	0.7989
0.9459	0.7209
0.8019	0.7217

0.6835	0.6828
0.7331	0.6945
0.7528	0.7306
0.6977	0.7242
0.6772	0.6264
0.635	0.6877
0.6871	0.6336
0.6081	0.6783
0.6364	0.6545
0.6925	0.6113
0.8164	0.7828
0.8428	0.6607
-0.606	-0.655
0.705	0.6862
0.6735	0.6523
0.7778	0.6539
-0.6449	-0.6223

0.7511	0.7514
-0.6018	-0.6566
0.7789	0.646
0.7879	0.792
0.7966	0.8082
0.7308	0.6021
0.6126	0.6467
0.7498	0.7137
0.612	0.6839
0.778	0.7843
0.7585	0.8166
0.7009	0.7442
0.6996	0.6398
0.8079	0.7357
0.6636	0.7898
0.6385	0.6125
-0.6004	-0.6035
0.7256	0.6867

-0.6034	-0.679
0.7308	0.6162
0.7626	0.7328
0.7121	0.6948
0.6631	0.6999
0.6078	0.6658
0.8273	0.704
0.861	0.7444
0.6956	0.7483
0.8204	0.6995
0.6991	0.687
0.7556	0.6192
0.624	0.6167
0.633	0.7062
0.6153	0.6101
0.6252	0.6128
0.8644	0.8893
0.7619	0.7742

0.6948	0.7
0.6039	0.708
0.623	0.6349
0.8312	0.7468
0.8392	0.754
0.7072	0.7198
0.7284	0.662
0.7284	0.662
0.6943	0.6492
0.7612	0.6585
0.6322	0.6149
0.6061	0.6367
0.6897	0.7639
0.8046	0.8265
0.7284	0.6891
0.682	0.6913
0.9122	0.7958

0.6605	0.9087
0.6158	0.7068
0.9229	0.8348
0.9169	0.8284
0.8202	0.8108
0.7425	0.7605
0.8791	0.8537
-0.6753	-0.7537
0.6302	0.692
0.7099	0.7369
0.6021	0.6645
0.6619	0.7026
0.6593	0.7658
0.6122	0.6219
0.7625	0.7454
0.6873	0.7655
0.681	0.6439
0.7077	0.7238
0.8796	0.8345
0.6709	0.6536

0.626	0.6365
0.6721	0.609
0.6298	0.6058
0.7336	0.7052
0.8658	0.8417
0.7518	0.7908
0.7582	0.7544
0.7849	0.8077
0.643	0.6951
0.6062	0.6346
0.9273	0.6313
0.6583	0.6857
0.7574	0.7222
0.6132	0.6214
0.61	0.629
0.6481	0.6047
-0.6061	-0.6338
-0.8334	-0.7521

-0.6912	-0.8139
0.6685	0.7136
0.6231	0.7233
0.8156	0.8162
0.8371	0.8563
0.7775	0.7569
0.6618	0.7325
0.7258	0.7058
0.6983	0.6037
0.733	0.7215
0.6126	0.6706
-0.685	-0.6276
0.6776	0.6933
0.6901	0.742
0.7721	0.72
-0.6713	-0.6638
0.6897	0.6462
0.6642	0.7042

0.732	0.6793
-0.6754	-0.6497
0.8659	0.8374
0.6756	0.6191
0.9261	0.8343
0.6092	0.6475
0.7025	0.682
0.6978	0.6986
0.7376	0.6329
0.6669	0.6521
0.6248	0.6789
0.6096	0.6117
0.7524	0.6945
0.653	0.6487
0.643	0.6554
0.6659	0.7007
0.6875	0.6806
0.7601	0.7335
0.7814	0.7782

0.6677	0.7295
0.7431	0.6983
0.7065	0.7133
0.6574	0.6813
0.6526	0.7053
0.6948	0.6557
0.6815	0.6483
-0.6377	-0.6654
0.6481	0.7994
0.607	0.6159
0.6513	0.6532
0.7871	0.7349
0.8036	0.8047
0.6761	0.6975
0.6057	0.6477
0.8193	0.8241
0.7387	0.7611
0.617	0.6339

0.7827	0.6867
0.7069	0.8252
0.7708	0.6756
0.745	0.6946
0.621	0.671
0.771	0.7427
0.6397	0.7129
0.7107	0.7235
0.6666	0.6748
0.7478	0.7668
0.7527	0.6937
0.769	0.6919
0.6526	0.642
0.6611	0.6158
0.7189	0.7353

0.6334	0.6136
0.8335	0.6086
0.6928	0.66
0.6468	0.6178
0.7224	0.7152
0.6442	0.6591
0.7535	0.8243
0.7104	0.7231
0.7798	0.8064
0.6038	0.6681
0.6665	0.6055
0.755	0.7171
-0.6328	-0.6461
0.699	0.733
0.7087	0.6998
0.6839	0.6124
0.6501	0.6488
0.6083	0.6108
0.6518	0.6605

0.9258	0.909
0.6284	0.6124
0.6164	0.674
-0.6548	-0.7605
0.7593	0.7402
0.7513	0.8362
0.7212	0.7435
-0.6786	-0.6252
-0.7321	-0.6689
0.849	0.8148
0.7141	0.6507
0.736	0.8106
0.6724	0.7018
0.8559	0.8096
0.6434	0.6351
0.7273	0.7003
0.6692	0.6223
0.6299	0.624
0.7935	0.7294

0.6344	0.7304
0.7441	0.7452
0.6923	0.7187
-0.6222	-0.6666
0.9361	0.9311
0.7544	0.7705
0.6603	0.621
0.6931	0.628
-0.7611	-0.6177
0.8914	0.8374
0.8146	0.6253
0.7359	0.6061
0.7617	0.6485
0.7793	0.7577
0.7472	0.7202
0.7588	0.6902
0.612	0.6274

0.6538	0.6868
-0.804	-0.8058
0.6807	0.767
0.6698	0.8258
0.7393	0.6706
0.7964	0.7622
0.9829	0.7778
0.9474	0.8095
0.747	0.8136
0.7561	0.8305
0.7398	0.7789
0.6226	0.6769
0.6681	0.7013
0.7206	0.6789
0.6397	0.7116
0.6564	0.6208
0.6767	0.6618

0.9134	0.6262
0.8681	0.7781
0.8355	0.6224
0.7872	0.7858
0.7346	0.8138
0.6274	0.6348
0.6595	0.7388
0.8593	0.8772
0.6371	0.6443
0.6669	0.6011
0.7012	0.6611
0.6869	0.7087
0.7349	0.7866
0.7357	0.6956
0.6324	0.6863

-0.6944	-0.7075
0.7038	0.608
0.6211	0.6262
0.6669	0.6426
0.633	0.6597
0.6719	0.6137
0.6838	0.7295
0.7162	0.7177
0.6991	0.6574
0.7241	0.7836
0.6887	0.6556
0.8372	0.8101
0.6725	0.6627
0.6465	0.7145
0.6812	0.7395
0.6182	0.6373
0.6753	0.7061
0.694	0.624

0.925	0.9067
0.6771	0.6201
0.6401	0.7711
0.7067	0.7725
0.6263	0.6037
0.8922	0.8832
0.8167	0.7573
0.6833	0.6088
0.636	0.6316
0.7432	0.7383
-0.8108	-0.7656
-0.825	-0.7848
0.7225	0.6703
0.9068	0.8974
0.7639	0.7278
0.7685	0.8099

0.7664	0.7609
0.6822	0.705
0.7616	0.6338
0.6181	0.6021
0.747	0.7962
0.7186	0.6614
0.7475	0.7269
0.6261	0.6046
0.824	0.6774
0.7005	0.6481
0.6106	0.6247
0.6705	0.6422
-0.7113	-0.6763
0.6246	0.71
0.9947	0.9159

0.6882	0.7548
-0.7061	-0.6329
0.6793	0.6865
0.6081	0.6556
0.6011	0.6082
0.6296	0.638
0.6893	0.6971
-0.7386	-0.7502
-0.6196	-0.6337
0.6195	0.6571
0.7222	0.7252
0.6355	0.6966
0.7606	0.7755
0.6072	0.765
0.6882	0.7244
0.6515	0.6218
0.6353	0.6279

0.6117	0.6075
0.8071	0.7196
0.8633	0.9017
0.8658	0.8849
0.6071	0.6254
0.7367	0.6927
0.6646	0.6972
0.6368	0.6543
0.7244	0.718
0.6548	0.6271
-0.7649	-0.7535
0.629	0.6405
0.6064	0.6118
0.8253	0.7654
0.6064	0.6118
0.8253	0.7654
0.6659	0.671
0.7273	0.7232
0.6766	0.6671

0.6236	0.6387
0.7308	0.7661
0.8713	0.8314
0.6592	0.7279
0.6856	0.7421
0.6726	0.7325
0.6697	0.6667
0.8778	0.6878
0.6363	0.7232
0.6149	0.6361
0.6025	0.6851
0.6857	0.6288
0.6466	0.7411
0.6565	0.6816
0.6537	0.6214
0.6676	0.6197

0.648	0.6062
0.7927	0.8074
0.6196	0.6597
0.6712	0.7067
0.7807	0.747
0.8311	0.8452
0.9468	0.9382
0.7232	0.7221
0.8411	0.6725
0.7349	0.7118
0.6684	0.7175
0.7638	0.7395
0.6611	0.6381
0.733	0.6967
0.6236	0.6504
0.772	0.6408
0.6366	0.6121
0.8206	0.6662

0.789	0.699
0.6559	0.6212
0.852	0.8311
0.7066	0.7075
0.6286	0.7124
0.6429	0.6098
0.832	0.7576
0.757	0.6757
0.7214	0.7354
0.6031	0.6358
-0.6789	-0.6919
0.6408	0.6019
0.7981	0.8383
0.7895	0.8302
0.7369	0.7365
0.6434	0.6065
0.6385	0.6224
0.6633	0.6268

0.6692	0.6568
-0.7225	-0.6104
-0.6798	-0.6875
0.8302	0.8054
0.681	0.7571
0.7824	0.8061
0.7063	0.6627
0.6395	0.6614
0.6427	0.627
0.6589	0.6565
0.7181	0.7513
0.7313	0.71
0.7979	0.7988
0.6137	0.6864
0.6924	0.6764
0.7663	0.6521
0.6349	0.6904

0.668	0.7521
0.8141	0.8167
0.7932	0.8274
0.607	0.6833
0.7015	0.7483
0.7167	0.6745
0.6785	0.705
0.9077	0.9034
0.7085	0.9591
-0.6455	-0.6822
0.6103	0.61
0.6485	0.6374
0.6597	0.621
0.9146	0.8898
0.6265	0.6077
0.6868	0.6384
0.7622	0.636

0.7957	0.791
0.8105	0.635
0.8518	0.8507
0.6927	0.6694
0.6778	0.642
0.6835	0.6527
0.6804	0.6009
0.6063	0.7198
0.6511	0.6416
0.6084	0.657
0.7192	0.7016
0.6454	0.6379
0.7436	0.614
0.7105	0.798
0.7314	0.7219

0.6844	0.6271
0.7155	0.7162
0.829	0.7466
0.8057	0.6686
0.6496	0.6215
0.7165	0.734
0.7236	0.6517
0.7254	0.7163
0.6968	0.732
0.6318	0.6226
0.6893	0.6781
0.7886	0.7916
0.9343	0.9506
0.7573	0.6706
0.6795	0.7017
0.6935	0.6315
0.7076	0.6685

0.9005	0.8226
0.8586	0.8767
0.8537	0.9009
-0.7226	-0.6768
-0.7461	-0.7215
0.7993	0.7654
0.67	0.6301
0.6198	0.7586
0.6347	0.6878
0.6471	0.6193
0.8225	0.7088
0.7117	0.7036
0.7068	0.7289
0.7087	0.6037
0.8071	0.7647
0.6661	0.7246
0.6338	0.6401
0.7432	0.7496

0.7973	0.6958
0.6975	0.6812
0.7657	0.72
0.7467	0.7991
0.7312	0.7029
0.6826	0.6206
0.6374	0.6422
0.6365	0.6645
0.6104	0.6261
0.8176	0.675
0.7684	0.6343
0.6997	0.641
0.6373	0.6999
0.6267	0.6848
0.6058	0.686
0.7275	0.6762
0.8208	0.7601

0.9648	0.6872
0.7468	0.6957
0.672	0.7106
0.8122	0.6897
0.7552	0.7334
0.7481	0.6514
0.6452	0.6215
0.6931	0.7535
0.7966	0.8036
0.6945	0.6389
0.654	0.6101
0.749	0.7545
0.7813	0.7906
0.6685	0.6729
0.6207	0.6114
0.61	0.6513
0.6847	0.6802

0.8379	0.8994
0.74	0.7685
0.8729	0.8501
0.7747	0.794
0.6281	0.6269
0.6387	0.6379
0.6953	0.7208
0.6773	0.6916
-0.6065	-0.66
0.7673	0.7728
0.73	0.7036
0.7303	0.8019
0.7011	0.633
0.8637	0.6627
0.795	0.6616
0.7017	0.7345
0.7861	0.8023
0.7573	0.7574

0.6703	0.6288
0.701	0.6378
0.6784	0.7357
-0.6824	-0.6418
0.6402	0.6018
0.7552	0.7334
0.7912	0.7954
0.7149	0.74
0.7089	0.7066
0.686	0.6126
0.7552	0.7334
0.7912	0.7954
0.7149	0.74
0.617	0.6854
0.6749	0.7997
0.6767	0.6027
0.6931	0.7535
0.7966	0.8036

0.6703	0.7107
0.6614	0.6532
0.6516	0.6904
0.6975	0.64
0.6205	0.6201
0.8989	0.6756
0.7089	0.7066
0.686	0.6126
0.7276	0.7959
0.7552	0.7334
0.7912	0.7954
0.7149	0.74
0.617	0.6854
0.6749	0.7997
0.6767	0.6027
0.7081	0.6008
0.6614	0.6532
0.6516	0.6904

0.6975	0.64
0.8989	0.6756
0.686	0.6126
0.7598	0.6854
0.7276	0.7959
0.7552	0.7334
0.6942	0.6221
0.6749	0.7997
0.6767	0.6027
0.601	0.69
0.6614	0.6532
0.6516	0.6904
0.6975	0.64
0.7089	0.7066
0.686	0.6126
0.6942	0.6221
0.6749	0.7997

0.6767	0.6027
0.6614	0.6532
0.6516	0.6904
0.6975	0.64
0.8989	0.6756
0.7089	0.7066
0.686	0.6126
0.7276	0.7959
0.7552	0.7334
0.7912	0.7954
0.7149	0.74
0.617	0.6854
0.6749	0.7997
0.6767	0.6027
0.6614	0.6532
0.6516	0.6904
0.6975	0.64
0.8989	0.6756

0.6344	0.644
0.7089	0.7066
0.686	0.6126
0.7276	0.7959
0.7552	0.7334
0.7912	0.7954
0.7149	0.74
0.617	0.6854
0.6749	0.7997
0.6767	0.6027
0.6703	0.7107
0.6669	0.7621
0.6614	0.6532
0.6516	0.6904
0.6975	0.64
0.6205	0.6201
0.8989	0.6756

0.6344	0.644
0.7089	0.7066
0.686	0.6126
0.7598	0.6854
0.7276	0.7959
0.7339	0.6664
-0.6151	-0.6535
0.666	0.6406
0.6125	0.6032
0.6346	0.6449
0.775	0.6982
0.6325	0.7016
0.677	0.6802
0.7026	0.6913
0.6374	0.6984
0.7493	0.7221

0.6037	0.6319
0.6644	0.6409
-0.8072	-0.761
0.7082	0.7317
0.9849	0.6716
0.743	0.7626
0.6414	0.6725
0.6992	0.7367
0.7335	0.7348
0.657	0.611
0.6744	0.6546
0.7652	0.7795
0.7571	0.6904
0.7615	0.7419
0.8069	0.7852
0.6985	0.8043
0.9303	0.8948

0.6084	0.628
-0.6117	-0.791
0.7884	0.7563
0.6711	0.7044
0.6152	0.656
-0.6032	-0.6963
0.6359	0.6852
0.8748	0.8827
0.727	0.733
0.7694	0.8051
-0.6185	-0.6299
0.6572	0.6465
-0.6364	-0.7202
0.8436	0.7107
0.8287	0.8228
0.6792	0.6517
0.6771	0.7057

0.6943	0.677
0.6131	0.7328
0.7552	0.7054
0.6423	0.8341
0.7255	0.7999
0.675	0.649
0.6217	0.6606
0.7528	0.7935
0.74	0.8026
0.6992	0.6568
0.7054	0.7288
0.898	0.6791
0.6215	0.6004
0.8547	0.6884
0.7563	0.711
0.6468	0.6084
0.7266	0.7245

0.7711	0.6276
0.6029	0.7189
0.8039	0.7462
0.6148	0.6306
0.7838	0.9962
0.7895	0.821
0.6959	0.659
0.8551	0.8435
0.6638	0.6546
0.6169	0.6627
0.6347	0.8251
0.7296	0.6926
0.8043	0.7986
0.6204	0.6072
0.6913	0.6683
0.6826	0.6242
0.6887	0.6227

0.642	0.6655
0.7618	0.7425
0.8993	0.813
0.7437	0.6987
0.777	0.6606
0.6082	0.6534
0.8092	0.8359
0.873	0.6669
0.689	0.6023
0.77	0.6917
-0.6337	-0.6248
0.6893	0.747
0.8256	0.7523
0.8302	0.8132
0.7846	0.7657
0.8259	0.7224

0.6669	0.6337
0.7185	0.7983
0.8287	0.7749
0.7619	0.7299
0.6592	0.6617
0.6605	0.658
0.6287	0.6718
0.7219	0.7013
0.6067	0.6272
0.7796	0.6152
0.6411	0.6114
0.7706	0.7216
0.6406	0.632
0.6123	0.617
0.6273	0.6058
0.7665	0.6098
0.6959	0.7928
0.7116	0.7589

0.6178	0.6691
0.6504	0.6358
-0.6288	-0.7048
0.6023	0.6216
0.6383	0.6527
0.6076	0.6807
0.8908	0.8963
0.7571	0.7564
0.7945	0.8191
0.8412	0.7966
0.6048	0.6915
0.7672	0.7322
0.7186	0.6078
0.743	0.647
0.7024	0.6116
0.6697	0.6017
0.8123	0.845
0.6839	0.6617

0.7217	0.6539
0.8002	0.7477
0.6054	0.6233
0.7448	0.7404
0.7533	0.8238
0.6321	0.643
0.9746	0.9809
0.6248	0.7058
0.7068	0.6815
0.8001	0.7512
0.7194	0.7887
0.6929	0.7296
0.6877	0.6033
0.6151	0.6152
0.6455	0.6632
0.6701	0.6498
0.6985	0.6174
0.6253	0.7393
0.6388	0.6051

0.6898	0.7124
0.7623	0.6777
0.732	0.6576
-0.6516	-0.7459
0.6001	0.6837
0.7133	0.6224
0.623	0.7125
0.6511	0.6675
0.6149	0.8527
0.6127	0.6843
0.702	0.7348
0.7827	0.8217
0.7026	0.7427
0.763	0.8425
0.7421	0.7939
0.8523	0.8396
0.7344	0.8323

0.6607	0.7731
0.7994	0.8679
0.6666	0.6485
0.9212	0.8527
0.6936	0.7475
0.6565	0.7264
0.6513	0.6717
0.6091	0.6762
0.6809	0.6926
0.9441	0.9073
0.9328	0.9102
0.7543	0.8121
0.7155	0.7614
0.8845	0.8558
0.6886	0.734
0.9387	0.9232
0.6674	0.6765

0.8217	0.8428
0.8424	0.8753
0.8755	0.8102
0.6851	0.7731
0.6061	0.743
0.8298	0.944
0.8298	0.944
0.6421	0.6547
0.6948	0.7106
0.6035	0.7375
0.7454	0.7819
0.6571	0.6704
0.8728	0.7736
0.7725	0.6542
0.6836	0.6017
0.6006	0.7152
0.735	0.682

0.6095	0.647
0.7916	0.7777
0.745	0.6081
0.6724	0.7467
0.7265	0.7374
0.6364	0.7231
0.7049	0.6794
0.7049	0.6794
0.7084	0.7302
0.6687	0.6838
0.6579	0.7184
0.757	0.8098
0.6822	0.6342
0.63	0.6331
0.7084	0.7302
0.6762	0.6487
0.7345	0.7233
0.6683	0.6223
0.6848	0.6267

0.6871	0.6776
0.6028	0.6275
0.6446	0.693
0.6068	0.7397
0.9547	0.9617
0.6429	0.7995
0.6003	0.6826
0.7483	0.612
0.809	0.7304
0.6952	0.7187
0.6215	0.6181
0.8176	0.6434
0.7054	0.7209
0.7573	0.7786
0.7556	0.8344
0.6362	0.6166
0.6172	0.7477
0.6174	0.6407

0.883	0.9017
0.9151	0.9534
0.7662	0.7477
0.7461	0.6985
0.7649	0.7308
0.6856	0.7187
0.7966	0.7603
0.611	0.6079
0.7288	0.759
0.8323	0.7018
0.6886	0.6471
0.6444	0.628
0.6168	0.6247
-0.7064	-0.7758
0.6657	0.6919
0.6989	0.7158
0.6948	0.6567
0.6486	0.6461

0.6329	0.6883
0.6195	0.662
0.6271	0.692
0.6059	0.6162
0.7203	0.8708
0.7021	0.6893
0.7592	0.6791
0.7155	0.8245
0.643	0.6758
0.64	0.6636
0.6486	0.6807
0.698	0.8095
0.6174	0.6009
0.6113	0.6096
0.6966	0.6916
0.6261	0.6176

0.7557	0.6991
0.7508	0.6024
0.6343	0.6828
0.6838	0.8039
0.8676	0.8529
0.8644	0.8623
0.7383	0.7287
0.658	0.6022
0.775	0.644
0.6001	0.6883
0.65	0.68
0.6212	0.7705
0.6643	0.6699
0.6323	0.7008
0.8051	0.8127
0.7029	0.7044

0.8274	0.6929
0.8068	0.7249
0.6186	0.6426
0.6783	0.6429
0.6328	0.6609
0.611	0.6023
0.6227	0.6635
0.8207	0.706
0.6157	0.6851
-0.7332	-0.7339
0.6429	0.6574
0.6664	0.6502
0.6287	0.6739
0.634	0.6606
0.7878	0.7678
0.8602	0.7045
0.7476	0.7599

-0.6293	-0.7246
0.8418	0.8772
-0.6087	-0.7231
0.6853	0.666
0.6604	0.7122
0.7329	0.7348
0.6179	0.6643
0.6756	0.6452
0.6535	0.6787
0.6654	0.775
0.748	0.8093
-0.6695	-0.6905
0.7281	0.797
0.7295	0.6156
0.7002	0.6585
-0.6267	-0.6316
0.8117	0.6847

0.7616	0.8141
-0.6719	-0.6806
0.6311	0.7008
0.8555	0.7002
0.8304	0.7441
0.8304	0.7441
0.909	0.7708
0.9244	0.8552
0.6423	0.6795
0.7693	0.777
-0.7094	-0.754
0.6251	0.6576
0.7691	0.7087
0.6118	0.6939
0.615	0.6627
0.6414	0.6357
0.73	0.7357
0.6719	0.7791
0.6065	0.6288
0.6682	0.6625

0.8317	0.7298
0.7381	0.696
0.7489	0.6532
0.9676	0.8144
0.9198	0.7709
0.9227	0.7798
0.9723	0.8353
0.6108	0.6152
0.6761	0.6136
-0.7299	-0.7006
0.6957	0.7666
0.6289	0.6182
0.7015	0.6304
0.6067	0.6116
0.7341	0.7223
0.7389	0.7925
0.6745	0.7425

0.7339	0.7337
0.7449	0.7189
0.6103	0.6167
0.6378	0.6914
0.6353	0.6496
0.633	0.6873
0.6511	0.6504
0.7391	0.8179
0.6023	0.7369
0.7155	0.7039
-0.6047	-0.6306
0.6876	0.6537
0.695	0.6493
0.63	0.6715
0.7291	0.7032
0.7819	0.6788
0.8126	0.748

0.7342	0.6721
0.7506	0.6801
0.8554	0.7459
0.6987	0.6661
0.7419	0.788
-0.7807	-0.8447
0.6447	0.7917
0.6829	0.7304
-0.6947	-0.7115
0.7638	0.7539
0.812	0.7539
0.6786	0.7538
0.7477	0.8032
0.8451	0.9267
0.621	0.6848
0.7387	0.6091
0.6033	0.6045

0.6522	0.613
0.6224	0.6261
-0.6873	-0.654
0.6394	0.6974
0.7153	0.7486
0.6072	0.7257
-0.7876	-0.8017
-0.6473	-0.8241
0.6382	0.7235
0.6202	0.6274
0.6325	0.6431
0.832	0.8313
0.61	0.7906
0.6418	0.6537
0.64	0.6346
0.6031	0.627

0.6094	0.6009
0.693	0.6984
0.6076	0.69
0.662	0.7064
0.6816	0.6771
-0.613	-0.777
-0.6432	-0.682
0.6016	0.6306
0.6079	0.7336
0.6962	0.7247
0.7335	0.685
0.6281	0.6715
0.7045	0.6526
0.7082	0.7168
0.7291	0.6113
0.6692	0.638
0.6684	0.6468
0.719	0.6355

0.7731	0.6713
0.7215	0.6665
0.6468	0.723
0.7287	0.7228
0.8714	0.747
-0.6146	-0.6247
0.6961	0.6626
0.6469	0.6154
-0.6718	-0.6428
0.6279	0.6585
0.7053	0.7464
0.6092	0.6184
0.6534	0.6928
0.83	0.8052
0.7036	0.6667

0.6905	0.659
-0.803	-0.7556
0.7153	0.7022
0.7473	0.6529
0.6784	0.6626
0.8666	0.8482
0.7718	0.6274
0.6084	0.6157
0.7461	0.7864
-0.6199	-0.6279
0.6307	0.6177
0.6572	0.6156
-0.6369	-0.636
0.627	0.6226
0.841	0.6121
0.7914	0.6059
0.6309	0.6657

0.6086	0.6408
0.9063	0.8382
0.9024	0.8151
0.7178	0.684
0.6327	0.6913
0.7698	0.7243
0.686	0.7431
0.8002	0.809
0.6556	0.7064
0.7186	0.6528
0.6435	0.7047
0.6139	0.6686
0.7957	0.6944
0.7098	0.7581
0.6097	0.6138
0.6745	0.6591
0.6831	0.7093
0.735	0.7557

0.6106	0.6119
0.6419	0.6415
-0.6184	-0.6435
0.754	0.7328
0.6064	0.668
0.6438	0.6862
0.7227	0.6847
0.7627	0.6264
0.6878	0.7206
0.8682	0.8522
0.7559	0.8125
0.7772	0.7377
0.795	0.8033
0.6903	0.6858
0.7083	0.7009
0.7843	0.7178
0.6841	0.627

0.7485	0.7237
0.8472	0.8174
0.6669	0.6184
0.8198	0.6163
0.8239	0.7806
0.6761	0.7314
0.7722	0.7102
0.8043	0.6728
0.6905	0.6748
-0.6463	-0.6192
0.682	0.6461
0.9045	0.9504
0.779	0.7294
-0.8061	-0.6252
0.6391	0.603
0.6508	0.6696
0.8672	0.6816
0.625	0.7333

0.7964	0.8014
0.676	0.6713
0.6642	0.6548
0.7278	0.7167
0.7533	0.6954
0.735	0.7395
0.775	0.7441
0.7353	0.7092
0.6286	0.6669
-0.6076	-0.6241
0.6218	0.6962
0.6595	0.6276
-0.683	-0.7425
0.6866	0.6191
0.6742	0.6278
0.9232	0.8391
0.728	0.7652

0.6502	0.618
0.7231	0.6172
-0.81	-0.8251
0.7072	0.7965
0.765	0.822
0.9596	0.9541
0.887	0.8654
0.6302	0.6742
0.7506	0.7727
0.6684	0.6754
0.7053	0.7492
0.6542	0.6329
-0.7728	-0.7
0.8621	0.7513
0.7763	0.6565
-0.7659	-0.7726
0.653	0.6395
0.6963	0.6494
0.7303	0.7964

0.7424	0.6638
0.8141	0.7626
0.7395	0.7007
0.7989	0.8104
0.6762	0.6372
0.6569	0.6946
0.651	0.6896
0.6829	0.6678
-0.7157	-0.7058
-0.6129	-0.6204
0.8308	0.8072
-0.6128	-0.6811
-0.6573	-0.8781
0.7712	0.8158
0.6274	0.736
0.6012	0.6432
0.6987	0.6598
0.6554	0.6157

0.6629	0.6881
-0.6148	-0.6958
0.6067	0.7384
0.7245	0.6943
0.709	0.7253
0.6591	0.6256
0.6927	0.7523
0.796	0.6921
-0.7249	-0.7943
0.7066	0.6295
0.6056	0.6655
0.7144	0.6387
0.7518	0.7235
0.6365	0.6323
0.6868	0.708
0.6186	0.639
0.7301	0.7149
0.8003	0.738
0.9621	0.6586

0.9876	0.6595
0.9663	0.7894
0.9563	0.8232
0.7332	0.7374
-0.7552	-0.7649
0.7582	0.6564
0.6619	0.65
0.8205	0.698
0.6579	0.6183
0.7386	0.7618
0.7687	0.7666
0.8803	0.6333
0.7256	0.7354
0.7264	0.7053
0.6637	0.6562
0.7348	0.7619
0.9231	0.8823
0.7137	0.7583

0.7305	0.7183
0.8063	0.7278
0.7436	0.667
0.867	0.8278
0.6154	0.732
0.8019	0.6237
0.7509	0.8208
-0.6431	-0.6893
0.7652	0.7371
0.7797	0.7334
0.8345	0.619
-0.6741	-0.6729
-0.7	-0.6261
0.7313	0.8702
0.6296	0.6627
0.6062	0.6255
0.7906	0.6915

0.6222	0.7127
0.6838	0.7288
0.6547	0.6318
-0.6568	-0.6219
0.7355	0.6849
0.706	0.6799
0.702	0.7501
0.8322	0.7258
0.9146	0.8338
0.6792	0.6915
0.7889	0.713
0.6875	0.668
0.6481	0.6835
0.6296	0.6249
0.7019	0.6495
0.7825	0.7481
0.6875	0.6899
0.8594	0.7788
0.7245	0.6901

0.7323	0.605
0.6991	0.6072
0.647	0.632
0.744	0.6836
0.7002	0.6108
-0.6194	-0.6815
0.6655	0.6948
-0.7941	-0.7617
0.6224	0.6642
0.6213	0.6141
0.7946	0.7973
0.6329	0.6211
0.7727	0.7064
0.9469	0.9332
0.7233	0.7881
0.6626	0.6991
-0.6803	-0.7025
0.7269	0.7589

0.7206	0.6257
0.6658	0.7346
0.7154	0.6727
0.8617	0.646
0.8236	0.6924
0.6766	0.6709
0.6983	0.6975
0.6061	0.6237
0.7398	0.7564
0.7379	0.7201
0.7126	0.7497
0.6629	0.6606
0.6321	0.6988
0.645	0.7702
0.6355	0.6571
0.6657	0.6219
0.754	0.7084
0.635	0.6497

0.8569	0.808
0.7512	0.7022
-0.6109	-0.6796
0.615	0.668
-0.8045	-0.8893
0.6477	0.633
0.7044	0.6852
0.7468	0.813
0.7856	0.8104
0.6389	0.78
0.7856	0.8104
0.6665	0.6788
0.863	0.9015
0.7474	0.7965
0.7443	0.7241
0.7375	0.6025
0.6576	0.6461
-0.7246	-0.7212

0.6763	0.6452
0.8724	0.8506
0.6961	0.7166
0.6695	0.6312
0.7939	0.8041
0.6751	0.678
0.6794	0.6997
0.6351	0.6053
0.6011	0.668
0.8674	0.7117
0.6341	0.6289
0.8185	0.8114
0.7179	0.644
0.6677	0.7954
0.6416	0.6447
0.6541	0.6648
0.6769	0.6362

0.8385	0.8182
0.669	0.693
0.6221	0.6237
0.7975	0.8034
0.6129	0.6382
0.6329	0.608
0.726	0.6077
0.6269	0.6141
0.6549	0.6319
0.6049	0.6662
0.6782	0.6731
0.7456	0.8103
0.6822	0.7558
0.8005	0.8408
0.9004	0.8202
0.6985	0.6554
0.7172	0.7177

0.7473	0.6179
0.7786	0.8267
0.7464	0.6809
0.6259	0.6649
0.9151	0.6583
0.6683	0.6983
0.6612	0.708
0.6036	0.6635
0.6717	0.6627
0.7674	0.7584
-0.7287	-0.6763
0.697	0.6408
0.6055	0.6443
0.6073	0.6894
1	1
0.6454	0.6341
0.6428	0.6111

0.7322	0.7551
0.6939	0.6681
0.6182	0.6736
0.7078	0.7322
0.837	0.8311
-0.8133	-0.6562
-0.6792	-0.7426
-0.6831	-0.6768
0.6023	0.6332
0.6268	0.7671
0.7515	0.6056
0.6264	0.6784
-0.6675	-0.7041
0.7163	0.8098
-0.6147	-0.6458
0.6424	0.6059
-0.608	-0.6267
0.7526	0.7105

0.8062	0.7669
0.8406	0.8524
0.7363	0.6147
0.7922	0.8366
0.7191	0.6456
0.6082	0.643
0.7072	0.6368
-0.6629	-0.7117
0.6992	0.8216
0.7115	0.6426
0.7548	0.741
0.732	0.7528
0.6631	0.6941
0.7332	0.9157
0.8399	0.7249
0.6303	0.6486
0.6572	0.6806

-0.6141	-0.6208
0.6056	0.6747
0.6803	0.7412
0.6027	0.6188
0.634	0.6594
-0.7113	-0.6541
0.7687	0.6772
-0.6375	-0.6046
0.9347	0.9962
0.7035	0.6957
0.6166	0.6023
0.7955	0.8493
-0.6264	-0.609
-0.8889	-0.7912
-0.6073	-0.7175
0.6109	0.6843
0.6111	0.6296
0.7472	0.7368

0.7598	0.7778
0.6872	0.6288
0.7693	0.6934
0.6216	0.646
0.7043	0.6954
0.9145	0.7884
0.9761	0.8524
0.7893	0.6357
0.8353	0.6166
0.7332	0.9157
0.8399	0.7249
0.9261	0.7918
0.6887	0.6003
0.8275	0.6707
0.9135	0.8093
0.7115	0.8896
0.6778	0.6732
0.6805	0.6391
0.8319	0.8115

-0.6263	-0.6339
0.6322	0.6244
0.7958	0.8161
0.6055	0.6528
0.7197	0.6507
0.8289	0.6459
0.6435	0.6806
-0.6171	-0.7493
0.6838	0.6629
0.6534	0.6327
0.6144	0.7216
0.7482	0.7301
0.6915	0.7489
0.6173	0.6773
0.6564	0.7206
0.6067	0.6387
-0.8621	-0.7619
0.6976	0.64
0.6114	0.6715

0.6401	0.6331
0.6116	0.6374
0.6323	0.701
0.6235	0.6328
0.6861	0.642
0.6364	0.6357
0.6634	0.677
0.622	0.7357
0.8293	0.8099
0.7491	0.7191
0.8305	0.8598
0.6206	0.6583
0.6327	0.6113
0.7486	0.724
0.7408	0.7667
0.6671	0.6314
0.6069	0.727
0.7341	0.6139

0.806	0.7381
0.6562	0.6771
0.7494	0.6815
0.6597	0.6438
0.6473	0.61
0.7094	0.7138
-0.6519	-0.7994
0.731	0.6597
0.6509	0.7183
0.6424	0.7231
0.8809	0.8733
0.6997	0.698
-0.8261	-0.8626
0.6787	0.716
0.6901	0.7193
0.6933	0.7234
0.7234	0.7536
0.6345	0.6619

0.7356	0.6922
0.7356	0.6922
0.7381	0.7505
0.6806	0.6372
0.808	0.7258
0.674	0.6383
-0.7469	-0.7837
0.7774	0.8232
0.7882	0.7202
0.8288	0.9406
0.8498	0.7216
0.7789	0.6293
0.6959	0.6233
0.6107	0.6637
0.7108	0.7799
0.61	0.6094
0.9163	0.6663

0.7092	0.656
0.6344	0.7299
0.6496	0.6126
0.8057	0.7443
0.7351	0.7548
-0.7158	-0.7518
-0.6758	-0.7198
0.7081	0.6948
0.6585	0.6069
0.943	0.7648
-0.7908	-0.9113
0.6171	0.675
0.626	0.7184
0.6985	0.6416
0.6266	0.8096
0.6822	0.721
0.7297	0.701
0.7871	0.66

0.8065	0.6591
0.895	0.8724
0.6139	0.6892
0.8116	0.7459
0.7478	0.7815
-0.7889	-0.6505
0.8132	0.7941
0.622	0.7026
0.9729	0.8387
0.9538	0.8316
0.7833	0.7803
0.6613	0.672
0.6451	0.6383
0.7477	0.6341
0.7477	0.6341
0.6314	0.626
0.7386	0.7568
0.7902	0.7209

0.6506	0.711
0.7665	0.623
-0.6042	-0.6588
0.8713	0.8663
0.8359	0.8191
0.9718	0.6775
0.9873	0.7747
0.9903	0.7271
0.6628	0.7304
0.8854	0.7667
0.6103	0.6519
0.6922	0.6335
0.9109	0.9117
0.6193	0.6077
0.6873	0.6184
0.8446	0.7622
0.6992	0.646
0.7494	0.7791
0.8773	0.834

-0.8734	-0.849
0.6902	0.8255
0.6796	0.7113
0.7806	0.7336
0.6603	0.6635
0.7794	0.7763
0.633	0.7067
0.7107	0.6826
0.6727	0.6591
0.6454	0.6319
0.6199	0.6584
0.6715	0.6513
0.6737	0.7061
0.7429	0.6417
0.6755	0.6235
0.7099	0.7104
0.6492	0.7332

0.6726	0.8202
0.7198	0.7617
0.8441	0.8165
0.6547	0.8209
0.7423	0.7887
0.6955	0.827
0.7539	0.7302
0.8098	0.778
0.6951	0.6678
0.7257	0.807
0.6188	0.8052
0.7555	0.7417
0.8005	0.7828
0.7922	0.7706
0.6311	0.613
0.6301	0.713

0.7122	0.7539
0.7812	0.7619
0.746	0.7415
0.6817	0.8078
0.6542	0.6522
0.7343	0.7897
0.8048	0.7863
0.6678	0.7489
0.7632	0.6887
0.6786	0.7835
0.6804	0.8332
0.6716	0.8328
0.6289	0.687
0.8783	0.8678
0.784	0.7139
0.6634	0.7652

0.7407	0.8511
0.6867	0.8019
0.6214	0.6757
0.8393	0.8626
0.627	0.7017
0.6637	0.8009
0.7315	0.7993
0.7406	0.7523
0.6773	0.7339
0.6733	0.7209
0.6148	0.6617
0.7713	0.7858
0.806	0.8397
0.6703	0.7487
0.7594	0.8374
0.6527	0.7609

0.7025	0.695
0.704	0.7228
0.8091	0.7332
0.7886	0.7389
-0.7083	-0.7082
0.6894	0.6991
0.6652	0.6556
0.764	0.7824
0.8512	0.8043
0.9404	0.7677
0.9499	0.8319
-0.6853	-0.7025
0.8556	0.7195
0.6587	0.6806
0.871	0.8428
0.7338	0.6245
0.8748	0.8609

0.9737	0.8771
0.9819	0.8574
0.9851	0.8713
0.7515	0.7484
0.8701	0.7078
0.6384	0.6867
0.6915	0.7249
0.6396	0.6279
0.7548	0.776
0.6836	0.625
0.7856	0.8052
0.7647	0.7669
0.6263	0.7664
-0.6235	-0.6419
0.6222	0.6991
0.6709	0.6866
0.7467	0.8436
0.8275	0.8063
0.7417	0.752

0.7847	0.7134
0.7728	0.6289
0.7818	0.6614
0.6946	0.6311
0.6168	0.6071
0.9393	0.7389
0.6945	0.6803
0.6848	0.6764
0.642	0.6081
0.6719	0.6543
-0.7674	-0.7437
0.8118	0.7983
0.7547	0.7635
0.7385	0.7183
0.7119	0.7479
0.6142	0.6062

0.7165	0.7101
-0.7123	-0.641
-0.6048	-0.6402
0.6329	0.6067
0.8378	0.7888
0.8337	0.7979
0.6874	0.6542
0.7856	0.7118
0.668	0.7176
0.7719	0.6501
0.7826	0.6645
0.6405	0.6122
0.8338	0.8445
0.8144	0.7957
0.7734	0.7249
0.7995	0.6483
0.6844	0.6736
-0.7164	-0.7227
0.7189	0.6245

0.6474	0.6012
-0.6411	-0.7157
0.7354	0.6285
0.6401	0.6387
-0.672	-0.6781
-0.7273	-0.7549
0.6057	0.6577
0.8729	0.6154
0.6399	0.6456
0.6093	0.6392
0.6293	0.6117
0.7096	0.647
0.6798	0.7415
0.7001	0.7426
0.922	0.7339
0.6873	0.6435
0.6928	0.752

0.6982	0.789
0.712	0.767
0.7367	0.6303
0.7653	0.7852
0.6033	0.7139
0.8569	0.7327
0.6599	0.6896
0.6753	0.7626
0.852	0.7261
0.7614	0.6806
0.665	0.7477
0.6583	0.7896
0.7561	0.7548
0.6074	0.8252
0.8015	0.6636
0.6669	0.7139

0.7784	0.6297
0.8458	0.6152
0.7185	0.6832
0.6122	0.6407
0.7895	0.7232
0.7033	0.6943
0.6677	0.7389
0.7364	0.6355
0.6331	0.6736
-0.8249	-0.8745
-0.6247	-0.6224
0.7743	0.8017
0.6738	0.6949
0.6422	0.6924
0.6402	0.6827
0.8363	0.7253

0.7107	0.6381
0.7843	0.7961
-0.6711	-0.67
0.6223	0.7032
0.7454	0.84
0.7746	0.8023
0.7833	0.7925
-0.7305	-0.7863
0.6016	0.6227
0.8233	0.7829
0.6629	0.6329
0.6559	0.7174
0.7551	0.8082
0.7599	0.657
-0.6879	-0.7012
0.7343	0.7331
0.6691	0.6168
0.6771	0.6612
0.7611	0.7714

0.7031	0.6087
-0.721	-0.7596
0.8226	0.8821
0.6335	0.6041
0.6867	0.7241
0.7277	0.667
0.6072	0.6443
0.6787	0.713
-0.6702	-0.6861
0.9793	0.6353
0.6747	0.649
0.7591	0.7551
0.6448	0.7314
-0.6623	-0.7223
0.7546	0.6662
0.6439	0.6991
0.7463	0.6585

0.6712	0.7067
0.6338	0.6619
0.8855	0.8012
0.8014	0.7311
0.6014	0.6548
0.7964	0.6168
0.6922	0.6719
0.6907	0.7441
-0.6678	-0.6638
0.7069	0.7483
0.6503	0.7996
0.8331	0.8313
0.8317	0.8348
0.7847	0.6558
0.7625	0.7877
0.6252	0.6493
0.8266	0.8482
0.728	0.7447
0.6155	0.6562
0.6465	0.7183
0.7108	0.7583
0.7505	0.7574
0.7197	0.7602

0.749	0.7797
0.9424	0.9289
0.7058	0.7575
0.7207	0.6558
0.705	0.6101
0.8656	0.8043
0.8434	0.7585
-0.6825	-0.796
0.6322	0.6348
0.8532	0.8402
0.7155	0.7461
0.6534	0.6358
0.7323	0.7645
0.6823	0.685
-0.6447	-0.6778
0.82	0.7882
0.6342	0.7145
0.6574	0.6143

0.7959	0.8328
0.8347	0.6823
0.8249	0.8383
0.8732	0.8624
0.8227	0.7852
0.6191	0.6052
-0.6144	-0.669
0.8506	0.7209
0.6067	0.6535
0.7377	0.7514
0.7596	0.8253
0.7015	0.6932
0.7712	0.6719
0.6059	0.7117
0.6286	0.6516
0.6711	0.6273
-0.6662	-0.6098
0.7531	0.6848
0.9137	0.6526
0.6528	0.6777
0.8558	0.7905
0.7086	0.6007

0.7302	0.6368
0.9161	0.9041
0.7069	0.6488
0.7185	0.6765
0.6341	0.6712
0.7669	0.7725
0.6134	0.6342
0.6514	0.7368
0.6787	0.7389
0.6837	0.6915
0.8599	0.867
0.7952	0.8438
-0.6943	-0.7603
-0.8124	-0.7424
-0.6947	-0.7584
0.7037	0.6196
0.7891	0.6713
0.7152	0.6322

0.8071	0.7721
0.7258	0.7653
0.6305	0.6993
0.884	0.8928
0.6153	0.7083
0.6434	0.6592
0.6496	0.6875
0.6182	0.6085
0.6692	0.6104
0.7896	0.8157
0.6056	0.6199
0.682	0.6192
0.6355	0.6835
0.73	0.6542
0.6232	0.644
0.8362	0.8289
0.6375	0.712
0.6644	0.6494
0.6533	0.7435

0.7959	0.6573
0.6095	0.7242
0.7388	0.7681
0.8671	0.8699
0.6127	0.6037
0.8127	0.8652
0.6473	0.6487
0.793	0.7574
0.7617	0.7426
-0.6646	-0.7279
0.778	0.6771
0.6379	0.6361
0.7833	0.6865
0.7091	0.642
0.7164	0.7075
0.7214	0.6054
0.836	0.8242
-0.8462	-0.7875
0.809	0.8031
0.6741	0.7737

0.8582	0.7185
0.6899	0.6397
0.7422	0.7493
0.6483	0.6096
0.7165	0.7675
0.6595	0.7168
0.83	0.8084
0.781	0.7129
0.6502	0.7184
0.6563	0.6527
0.722	0.617
0.6206	0.6053
-0.7199	-0.8224
0.6802	0.6645
0.6303	0.6271
0.6341	0.6097
0.6103	0.6642
0.7135	0.6397
0.6058	0.607
0.8133	0.798

0.9724	0.8343
0.6157	0.6296
0.631	0.6728
0.7412	0.7513
0.6268	0.679
0.678	0.8242
0.9545	0.6951
0.7962	0.7722
-0.672	-0.7643
0.6108	0.6127
0.7921	0.7669
0.702	0.601
0.6578	0.6107
0.8235	0.8342
0.7011	0.6719
0.6073	0.6343
0.7289	0.6195
-0.7757	-0.6923

0.807	0.6737
-0.6716	-0.8062
0.6883	0.7394
0.6154	0.6242
-0.747	-0.7217
-0.6229	-0.7151
0.7494	0.6941
0.7046	0.793
0.6236	0.7864
0.6664	0.6944
0.7267	0.694
0.7785	0.7542
0.7793	0.7006
0.6568	0.6005
0.7792	0.7266
0.8021	0.7914
0.877	0.7148
0.6404	0.6241

0.6121	0.6662
0.7247	0.6614
0.8039	0.7078
0.6006	0.7005
0.8574	0.7847
0.8532	0.6098
0.7291	0.6245
0.835	0.8345
0.8619	0.8407
-0.6876	-0.739
0.7518	0.7203
0.7409	0.6387
-0.6744	-0.6252
0.6964	0.663
0.7259	0.7389
0.6031	0.6518
0.6048	0.6645

0.6028	0.7048
0.6216	0.6446
-0.6058	-0.6638
0.7781	0.6662
0.7684	0.7994
0.705	0.6249
0.6507	0.7612
0.6077	0.6771
0.7351	0.7764
0.7476	0.7404
0.6059	0.6368
0.6775	0.6404
-0.6861	-0.6788
0.6174	0.6255
0.637	0.6709
0.6342	0.6257
0.6949	0.6775
0.6844	0.6704
0.6476	0.6893
0.662	0.6428
0.7435	0.689

0.7534	0.7041
0.877	0.9011
0.6552	0.6784
0.7677	0.7484
0.7478	0.7774
0.6603	0.6749
0.7566	0.6799
0.7277	0.7348
0.6146	0.6762
0.8809	0.604
0.737	0.7553
0.6924	0.7122
0.6649	0.6035
0.785	0.6667
0.7548	0.7519
0.7525	0.6761
0.7468	0.6715
0.8512	0.7429
0.6866	0.6772

0.7455	0.6822
0.6152	0.6868
0.6691	0.6037
0.9056	0.8465
0.6384	0.6136
0.8348	0.8367
0.6898	0.7028
0.7387	0.8094
0.8994	0.9974
0.7047	0.674
0.6185	0.6225
0.7455	0.8067
0.8205	0.6491
0.6682	0.7519
0.7026	0.7292
0.6096	0.6144
0.7651	0.6312
0.7338	0.7231
0.8415	0.6354

0.6685	0.6962
0.7272	0.7869
0.6685	0.6796
0.7304	0.708
0.6015	0.6119
0.7366	0.7336
0.8899	0.8584
0.933	0.9348
0.8254	0.7667
0.6125	0.6439
0.6737	0.6645
0.7862	0.7216
0.6296	0.6743
0.6345	0.7558
-0.6529	-0.707
-0.7081	-0.6796
0.9357	0.9036
0.8358	0.9121

0.7463	0.7348
0.7192	0.7704
0.6116	0.6263
0.7272	0.6652
0.6785	0.6308
0.6819	0.7459
0.7089	0.6305
0.6772	0.7416
0.6675	0.7476
0.7128	0.771
0.8232	0.6845
0.7329	0.6649
0.7094	0.6405
0.7787	0.8311
0.9083	0.9114
0.7975	0.7857
0.7943	0.8197
0.6642	0.7523

0.7191	0.7296
0.7973	0.7738
0.6704	0.6011
0.6919	0.6289
0.7949	0.6322
0.7685	0.7391
0.7407	0.7449
0.7551	0.7343
0.7208	0.7416
0.6547	0.6798
0.7803	0.7072
0.6065	0.6876
0.6364	0.6688
0.7574	0.8032
0.6074	0.8015
0.8025	0.7474
0.7874	0.6394
0.8132	0.7253
0.788	0.694
0.7191	0.7527
0.7242	0.7681
0.643	0.6135

0.8772	0.8753
0.8831	0.8736
-0.8647	-0.7118
0.6291	0.6942
0.6591	0.6475
0.9554	0.8343
0.6498	0.6603
0.8889	0.7421
0.6307	0.6014
0.7026	0.7569
0.6569	0.6352
0.759	0.7537
0.672	0.6014
-0.6526	-0.7539
0.7944	0.6989
0.6223	0.6484
0.72	0.748
0.8107	0.7783
0.6963	0.641

0.6642	0.6173
0.8118	0.7009
0.6711	0.6336
0.6356	0.7
0.674	0.7751
0.7397	0.6845
0.6529	0.6542
0.9181	0.9321
-0.7651	-0.6446
0.6032	0.6304
0.7661	0.7756
-0.602	-0.6391
0.66	0.6522
0.6266	0.6736
0.6554	0.7598
0.7055	0.6474
0.6254	0.7184
0.6702	0.852
0.7613	0.8225

0.7391	0.8229
0.7372	0.8459
0.6116	0.7325
0.7292	0.8524
0.6376	0.7231
0.6615	0.6352
0.7414	0.8084
