# Supplementary material for: RNA Expression Profile and Potential Biomarkers in Patients With Spinocerebellar Ataxia Type 3 From Mainland China
Source: Front Genet. 2019 Jun 13;10:566. doi: 10.3389/fgene.2019.00566 (PMC6584761; doi:10.3389/fgene.2019.00566)
Supplement: Supplementary file 2 [file Table_1.docx]

Table S1. The information about locus, dysregulation, and primers of lncRNAs

| lncRNAs | Locus | Dysregulation | Primers |
| --- | --- | --- | --- |
| LTCONS_00014756 | chr1:173827684 -173837219 | Up | F:ATTGATGGAGCTACTTGGAGAAA  R:CAGATTGCCTTAAACCAGTTGTG |
| LTCONS_00097842 | chr1:505805-  508617 | Down | F: TCCACCCACCCAATTGCTAT  R: TGCTAGGTGAGGGTGGAAAG |
| LTCONS_00114811 | chr2:148384847-148390748 | Up | F:TTGATGCCAAGGAGGAAAGGT  R:CTGCCACTGCTTATCAGCAAGAC |
| LTCONS_00130225 | chr3:46435247 -  46446723 | Down | F:GGGAGTAAGAGGAAGTGTCAGCA  R:GGAGGAGACCAGGCAGGAGTT |
| LTCONS_00150373 | chr4:114705732 -114712542 | Up | F:GGAGAAAAGAAAGATTTATGTTGGTA  R:TCGGACTCTTGTCTGTCTCTGAA |
| LTCONS_00174904 | chr6:3101122-3101734 | Up | F: ACTACAATCCTTAAAGCTCCCG  R: CCAGGCTGGTCTCGAACTCC |
| LTCONS_00175040 | chr6:2839545-2842233 | Up | F: GGCCACAAGCTCTGGATGA  R: GGCGGACAGAAGGACCACT |
| LTCONS_00175021 | chr6:1153053-1207092 | Up | F: CTCAGGCTGCAAGCAGTGAC  R: GGGAAGGGCAGGAACAACTC |
| LTCONS_00166467 | chr6:113961934 -113967791 | Up | F:GGAAACCAGCAAATACAAGATAGG  R:GAAACATCAAAGCTCACCTCCATC |
| LTCONS_00169021 | chr6:11759361 -  11777365 | Down | F: CCTCCTCCTCCTTCAAAGCA  R: AATCCCTGGAACCCGTGAAT |
| LTCONS_00176188 | chr7:20256990 -  20261324 | Down | F: TGCAAATTGTGGTTCCTGGG  R: CCGAAGAGCAACACACCAAA |
| LTCONS_00051791 | chr13:31723572  -31736525 | Up | F:TTGTGAATCTCGAAATGAACTGG  R: AGGCCAACAATGTTAGCAACG |
| NONHSAT007220.2 | chr1:162495162-162499391 | Up | F:CTGAGGGAGGTGGCTACAATAAC  R:AGAGCTGAGCCTCTTGTGGTATA |
| NONHSAT114357.2 | chr6:109002487-109005971 | Up | F:AGTGTCACAGGTCCCTAACAATAA  R:GAGAACAGCAGATCCCAAGAGC |
| NONHSAT022144.2 | chr11:65266530 -65273915 | Down | F:GTCCTGGAGAAATAGTAGATGGCA  R:ACAAACTGCTTACAGATTTGCTGA |
| NONHSAT165686.1 | chr13:48807358-48835996 | Up | F:CTGGCTTCACGTTTCTGAATACC  R:TTAACATCAAGGCTGGAACCTAT |
| NONHSAT146442.2 | chr17:19092978 -19093558 | Up | F: CGTGTAGAGCACCGAAAACC  R:GAGAAGAACGATCATCAATGGC |
| NONHSAT177312.1 | chr17:34639876 -34641846 | Up | F: CCTCTTTGCCACCAATACCAT  R: GAGCAGCTCAGTTCAGTTCCAG |
| NONHSAT180373.1 | chr19:36392463-36394757 | Up | F: GGTGTCTCCGTTACTGCCTTTG  R: ACTCCCATCTTCTCAGCGTTTC |
| NONHSAT192876.1 | chr22:36688269-36697057 | Up | F:TCAAGAACAAGCATGAGGCAATG  R: TGGTCGCTGAGGTCTGTGGAG |
| GAPDH |  |  | F: CCATGGGTGGAATCATATTGGA  R: TCAACGGATTTGGTCGTATTGG |

Novel lncRNAs: using the ‘LTCONS’; known lncRNAs: using the ‘NONHSAT’

F: represents forward primer; R: represents reverse primer；

Location information based on database hg19 (raw data from high-throughput sequencing)
